# Biocatalytic Synthesis of Homochiral 2-Hydroxy-4-butyrolactone Derivatives by Tandem Aldol Addition and Carbonyl Reduction

**DOI:** 10.1021/acscatal.3c00367

**Published:** 2023-04-06

**Authors:** Carlos J. Moreno, Karel Hernández, Samantha Gittings, Michael Bolte, Jesús Joglar, Jordi Bujons, Teodor Parella, Pere Clapés

**Affiliations:** † Dept. of Biological Chemistry, Institute for Advanced Chemistry of Catalonia, IQAC-CSIC, Jordi Girona 18-26, 08034 Barcelona, Spain; ‡ Prozomix Ltd., West End Industrial Estate, Haltwhistle, Northumberland NE49 9HA, United Kingdom; § Institut für Anorganische Chemie, J.-W.-Goethe-Universität, Frankfurt/Main, Max-von-Laue-Str. 7, D-60438 Frankfurt/Main, Germany; ∥ Servei de Ressonància Magnètica Nuclear. Universitat Autònoma de Barcelona, 08193 Bellaterra, Spain

**Keywords:** biocatalysis, 2-oxoacid aldolase, ketoreductases, aldol addition, 2-hydroxy acids, 2-hydroxy-4-butyrolactones

## Abstract

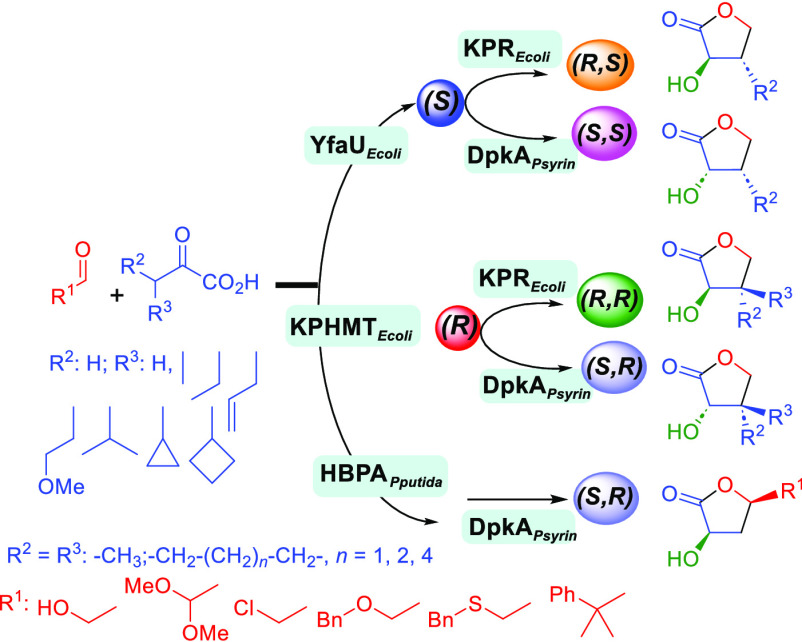

Chiral 2-hydroxy acids and 2-hydroxy-4-butyrolactone derivatives are structural motifs often found in fine and commodity chemicals. Here, we report a tandem biocatalytic stereodivergent route for the preparation of these compounds using three stereoselective aldolases and two stereocomplementary ketoreductases using simple and achiral starting materials. The strategy comprises (i) aldol addition reaction of 2-oxoacids to aldehydes using two aldolases from *E. coli*, 3-methyl-2-oxobutanoate hydroxymethyltransferase (KPHMT_*Ecoli*_), 2-keto-3-deoxy-l-rhamnonate aldolase (YfaU_*Ecoli*_), and *trans*-*o*-hydroxybenzylidene pyruvate hydratase-aldolase from *Pseudomonas putida* (HBPA_*Pputida*_) and (ii) subsequent 2-oxogroup reduction of the aldol adduct by ketopantoate reductase from *E. coli* (KPR_*Ecoli*_) and a Δ^1^-piperidine-2-carboxylate/Δ^1^-pyrroline-2-carboxylate reductase from *Pseudomonas syringae* pv. tomato DSM 50315 (DpkA_*Psyrin*_) with uncovered promiscuous ketoreductase activity. A total of 29 structurally diverse compounds were prepared: both enantiomers of 2-hydroxy-4-butyrolactone (>99% ee), 21 2-hydroxy-3-substituted-4-butyrolactones with the (2*R*,3*S*), (2S,3*S*), (2*R*,3*R*), or (2S,3*R*) configuration (from 60:40 to 98:2 dr), and 6 2-hydroxy-4-substituted-4-butyrolactones with the (2S,4*R*) configuration (from 87:13 to 98:2 dr). Conversions of aldol adducts varied from 32 to 98%, while quantitative conversions were achieved by both ketoreductases, with global isolated yields between 20 and 45% for most of the examples. One-pot one-step cascade reactions were successfully conducted achieving isolated yields from 30 to 57%.

## Introduction

**Figure 1:**
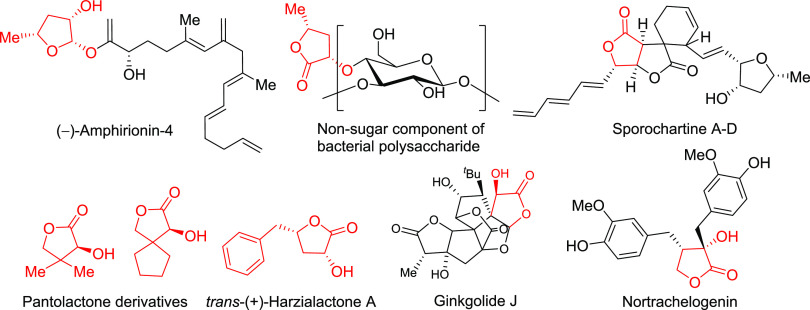
Examples of biologically relevant compounds bearing 2-hydroxy-4-butyrolactone derivatives.

Chiral 2-hydroxy acids and 2-hydroxy-4-butyrolactone derivatives are interesting compounds frequently found in naturally occurring biologically active products, synthetic drugs, and biodegradable polymers (e.g., poly α-hydroxy acids for biomedical and pharmaceutical applications) (Figure 1).^1^ Moreover, they constitute an important class of building blocks and chiral auxiliaries (e.g., (*R*)- or (*S*)-pantolactone derivatives) in asymmetric organic synthesis.^2^

**Scheme 1:**
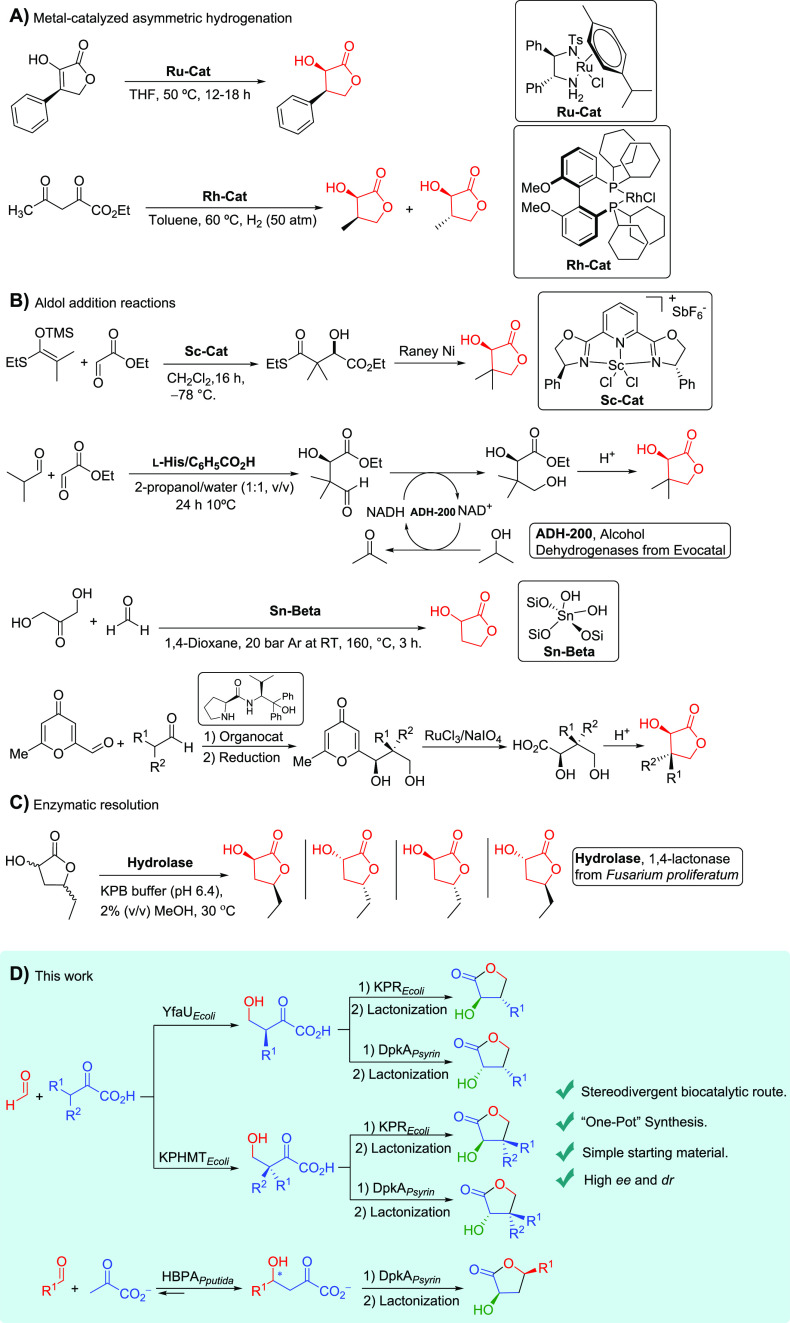
Synthetic Methodologies for the Preparation of 2-Hydroxy-4-butyrolactone Derivatives (A) Metal-catalyzed asymmetric.^1b,4a^ (B) Aldol reaction.^2j,7b,1j,7a,7c^ (C) Enzymatic resolution.^5^ (D) This work: stereodivergent biocatalytic approach. Stereoselective aldol addition of 2-oxoacid derivatives to aldehydes catalyzed by 2-oxoacid aldolases, namely, 3-methyl-2-oxobutanoate hydroxymethyltransferase (KPHMT_*Ecoli*_, EC 2.1.2.11), 2-keto-3-deoxy-l-rhamnonate aldolase (YfaU_*Ecoli*_, EC 4.1.2.53), both from *E. coli*, and *trans*-*o*-hydroxybenzylidene pyruvate hydratase-aldolase from *Pseudomonas putida* (HBPA_*Pputida*_ EC 4.1.2.45) followed by asymmetric enzymatic reduction using ketopantoate reductase from *E. coli* (KPR_*Ecoli*_, EC 1.1.1.169) and an NAD(*P*)H-dependent Δ^1^-piperidine-2-carboxylate/Δ^1^-pyrroline-2-carboxylate reductase from *Pseudomonas syringae* pv. tomato DSM50315 (GenBank: DQ017704.1) (DpkA_*Psyrin*_, EC 1.5.1.21).

Many efforts have been dedicated to the synthesis of 2-hydroxy-4-butyrolactone derivatives.^1g,2e,3^ As examples of methods for their synthesis, it is worth mentioning (Scheme 1): enzymatic or metal-catalyzed asymmetric reduction,^1b,4^ resolution procedures,^5^ oxidation of vicinal diols,^6^ and stereoselective aldol addition using metal- or organocatalysts in combination with enzymatic reduction.^1j,2b,2j,7^ Although organocatalysis and metal-catalyzed asymmetric approaches have reached a high degree of efficiency, in many instances, organocatalysts still suffer from low turnover numbers, and metal catalysis still needs extreme temperatures, sophisticated metal ligands, expensive heavy metals, and high hydrogen pressure. However, although enzymatic carbonyl reduction of 2-oxoacid derivatives has been reported,^8^ the stereoselective reduction of 4-hydroxy-2-oxoacid derivatives to produce 2-hydroxy-4-butyrolactone derivatives remains unexplored.

**Scheme 2:**
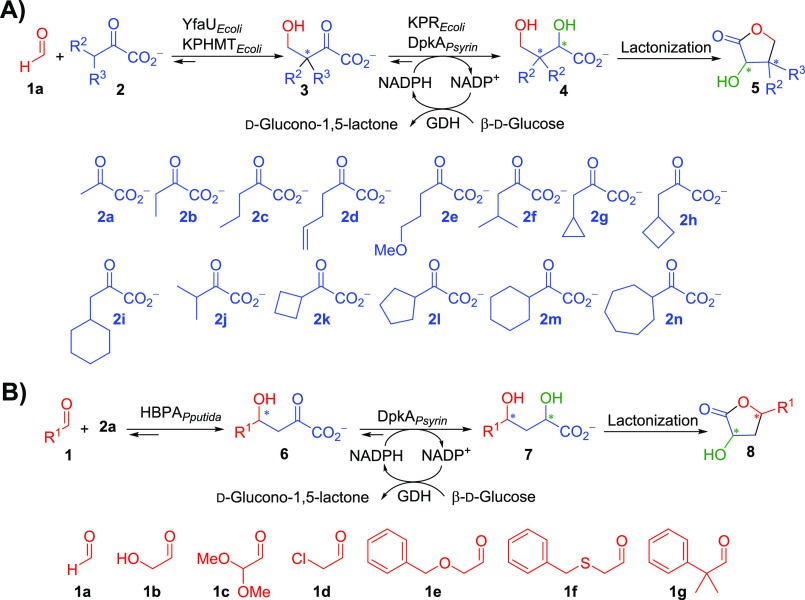
Biocatalytic Synthesis of 2-Hydroxy-4-butyrolactones by Tandem Aldolase and Ketoreductase Starting from Aldehydes **1** and 2-Oxoacids **2** (A) Synthesis of 3-substituted-2-hydroxy-4-butyrolactones: aldolases: YfaU_*Ecoli*_ and KPHMT_*Ecoli*_; ketoreductases: KPR_*Ecoli*_ and DpkA_*Psyrin*_. (B) Synthesis of 4-substituted-2-hydroxy-4-butyrolactones: aldolase HBPA_*Pputida*_ and ketoreductase DpkA_*Psyrin*_. GDH: glucose dehydrogenase from Prozomix.

We have envisioned a straightforward asymmetric construction of 2-hydroxy-4-butyrolactone derivatives by a synthetic route consisting of a stereoselective enzymatic aldol addition of 2-oxoacids to aldehydes and subsequent asymmetric biocatalytic reduction of the 2-carbonyl group (Scheme 1). Herein, we demonstrated the feasibility of this approach by using a selection of 2-oxoacids (**2**) and aldehydes (**1**) to enzymatically generate 3- and 4-substituted 4-hydroxy-2-oxoacids **3** and **6**, respectively, with defined stereochemistry. Then, a stereoselective biocatalytic reduction of the 2-carbonyl group was conducted to produce 2-hydroxyacids **4** and **7**, which, after intramolecular esterification (i.e., lactonization) taking place during the workup and purification steps, rendered 3- and 4-substituted-2-hydroxy-4-butyrolactones **5** and **8**, respectively (Scheme 2).

Three stereoselective 2-oxoacid aldolases were selected as biocatalysts for the aldol reactions, namely, 3-methyl-2-oxobutanoate hydroxymethyltransferase (KPHMT_*Ecoli*_) and 2-keto-3-deoxy-l-rhamnonate aldolase (YfaU_*Ecoli*_) and its W23V variant, both from *E. coli*.^9^ These were employed for the aldol addition of 2-oxoacids **2a**–**2n** to formaldehyde **1a**. The third one, *trans*-*o*-hydroxybenzylidene pyruvate hydratase-aldolase from *Pseudomonas putida* (HBPA_*Pputida*_), and its H205A variant were selected as the catalyst for the stereoselective aldol addition of sodium pyruvate **2a** to aldehydes **1a**–**1g**.^10^ In addition, two reductases were employed to convert the carbonyl group into a hydroxyl group. The ketopantoate reductase from *E. coli* (KPR_*Ecoli*_) in vivo catalyzes the NADPH-dependent reduction of ketopantoate to pantoate as part of the pantothenate biosynthetic pathway.^11^ Ketopantoate bears a *gem*-dimethyl quaternary center, and therefore, we envision that KPR_*Ecoli*_ could be active toward 3-substituted-2-oxo acids **3**. The second one, Δ^1^-piperidine-2-carboxylate/Δ^1^-pyrroline-2-carboxylate reductase from *Pseudomonas syringae* pv. tomato DSM 50315 (DpkA_*Psyrin*_), was described as an imine reductase transforming 3,4,5,6-tetrahydropyridine-2-carboxylic acid and 3,4-dihydro-2*H*-pyrrole-5-carboxylic acid into l-pipecolic acid and l-proline, respectively.^12^ In this case, during our ongoing investigation on iminoreductases, we serendipitously found that DpkA_*Psyrin*_ had a promiscuous ketoreductase activity, and consequently, we consider it interesting to exploit its synthetic capabilities. Moreover, both reductases have been underdeveloped for the synthesis of 2-hydroxy acids.

## Results and Discussion

### Synthesis and Product Characterization

**Scheme 3:**
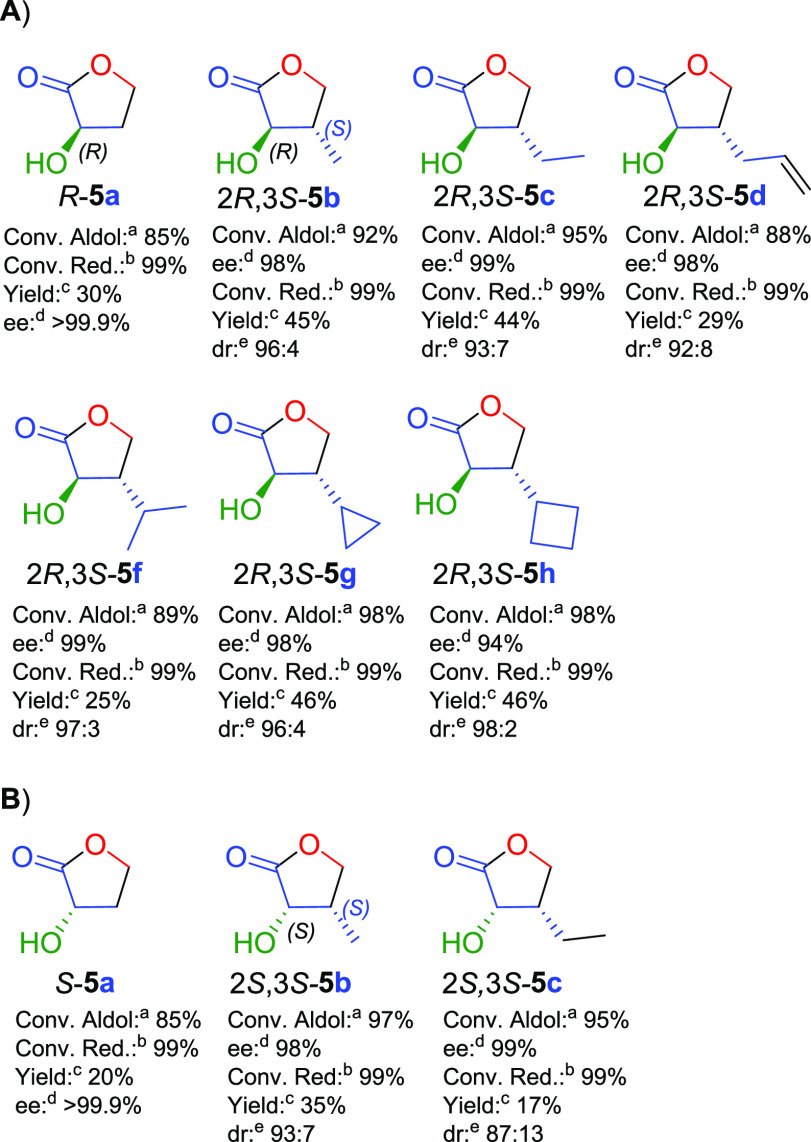
One-Pot Two-Step Synthesis of 3-Substituted-2-hydroxy-4-butyrolactones **5** by Tandem Biocatalytic Aldol-Reduction Reactions Catalyzed by Tandem YfaU_*Ecoli*_ and (A) KPR_*Ecoli*_ and (B) DpkA_*Psyrin*_ Conditions: 1 mmol scale, total volume (10 mL) at 25 °C, and magnetically stirred at 250 rpm; YfaU_*Ecoli*_ wt (3 mg purified protein mL^–1^) in plain water (4 mL), 2-oxoacids (**2a**–**i**) (0.1 M), and NiCl_2_ (1 mM) were added. The reaction was started by adding formaldehyde (**1**, 0.1 M). After 24 h, the reduction reaction (20 mL final volume) was carried out by adding EDTA (5 mM), glucose (0.2 M), GDH (3.4 U mL^–1^), KPR_*Ecoli*_ (4.7 U mL^–1^) or DpkA_*Psyrin*_ (10^–3^ U mL^–1^), and finally NADP^+^ (5 mM). After purification by anion exchange chromatography and eluting with HCO_2_H (1 M), the lactonization occurred during freeze drying the pure fraction pool. The product was then further purified by column chromatography on silica with a step gradient of hexane/EtOAc (see the SI). HPLC monitoring conditions: RP-HPLc XBridge C18, 5 μm, 4.6 × 250 mm column. The solvent system: solvent (A): 0.1% (v/v) trifluoroacetic acid (TFA) in H_2_O and solvent (B): 0.095% (v/v) TFA in CH_3_CN/H_2_O 4:1, flow rate 1 mL min^–1^, detection at 215 nm at 30 °C. Precolumn derivatization with BnONH_2_ elution conditions: gradient from 10 to 100% B over 30 min (reaction with compounds **2a**–**l**) and 10 to 100% B over 60 min (reaction with compounds **2m**,**n**). Conversion of aldol addition. Conversion of the reduction. Isolated yields. Enantiomeric excess of the reduction determined by HPLC on chiral stationary phases. Diastereomeric ratio determined by NMR.

**Scheme 4:**
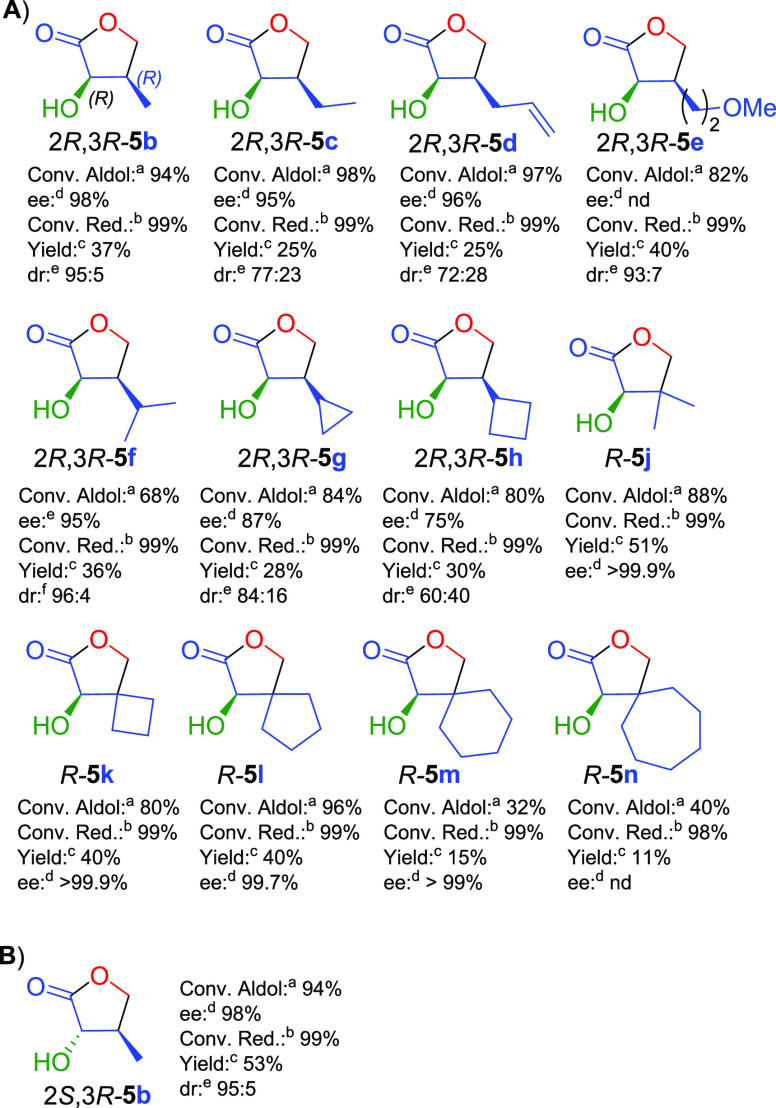
One-Pot Two-Step Synthesis of 3-Substituted-2-hydroxy-4-butyrolactones **5** by Tandem Biocatalytic Aldol-Reduction Reactions Catalyzed by Tandem KPHMT_*Ecoli*_ and (A) KPR_*Ecoli*_ and (B) DpkA_*Psyrin*_ Conditions: 1 mmol scale, total volume (10 mL) at 25 °C, and magnetically stirred at 250 rpm; KPHMT_*Ecoli*_ wild-type (1 mg purified protein mL^–1^) in plain (6.8 mL) water, CoCl_2_ (1 mM), and 2-oxoacids (**2a**–**i**) (0.1 M) were added. The reaction was started by adding formaldehyde (0.1 M). After 24 h, the reduction reaction, workup, lactonization, purification, and HPLC reaction monitoring were conducted as described in Scheme 3 (see also the SI). Conversion of aldol addition. Conversion of the reduction. Isolated yields. Enantiomeric excess of the reduction determined by HPLC on chiral stationary phases. Diastereomeric ratio determined by NMR.

**Scheme 5:**
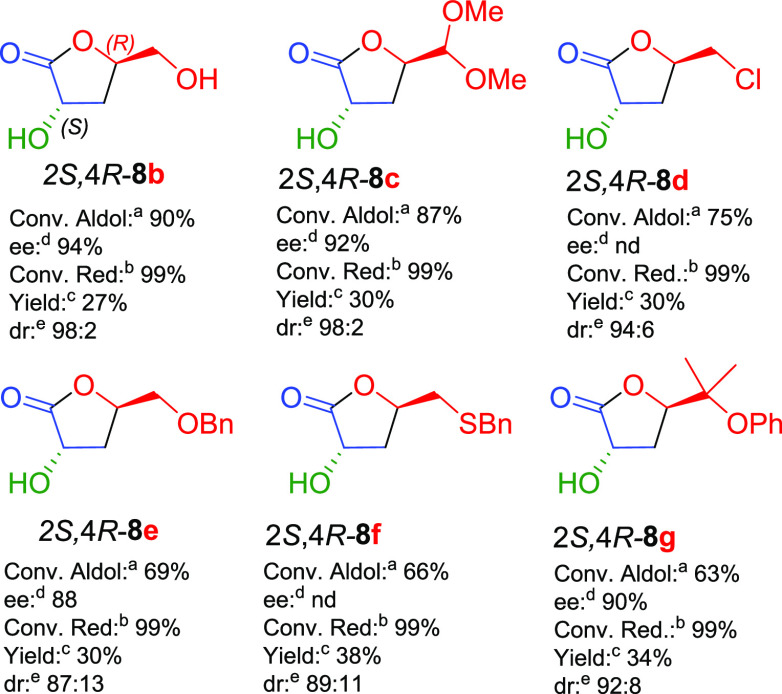
One-Pot Two-Step Synthesis of 4-Substituted-2-hydroxy-4-butyrolactones **8** by Tandem Biocatalytic Aldol-Reduction Reactions Catalyzed by Tandem HBPA_*Pputida*_ and DpkA_*Psyrin*_ Conditions: 1 mmol scale, 8.3 mL total volume at 25 °C, and orbitally stirred at 250 rpm. Sodium pyruvate **2b** (0.1 M), aldehyde **1b**–**g** (0.1 M), HBPA wild-type and H205A variant for substrates **1d** and **1f** (1 mg protein mL^–1^). After 24 h, the reduction reaction, workup, lactonization, purification, and HPLC reaction monitoring were conducted as described in Scheme 3, except the EDTA addition (see also the SI). Conversion of aldol addition. Conversion of the reduction. Isolated yields. Enantiomeric excess of the reduction determined by HPLC on chiral stationary phases. Diastereomeric ratio determined by NMR.

We began our investigations assaying KPR_*Ecoli*_ and DpkA_*Psyrin*_ as catalysts for the reduction of the 4-hydroxy-2-ketoacids **3** and **6**, obtained from the enzymatic aldol addition of 2-oxoacids **2** to aldehydes **1** (Scheme 2). To this end, the aldol reaction was first run under the conditions described in our previous studies.^9,10^ When the aldol adduct attained the maximum concentration (i.e., steady state), YfaU_*Ecoli*_ and KPHMT_*Ecoli*_ were inactivated by adding EDTA to avoid enzymatic retroaldolisis, whereas in the case of HBPA, this was unnecessary. Then, DpkA_*Psyrin*_ or KPR_*Ecoli*_, glucose, glucose dehydrogenase [GDH, from Prozomix, see the Supporting Information (SI)], and NADP^+^ were added. The enzymatic aldol and reduction reactions were monitored by reverse-phase high-performance liquid chromatography (HPLC), and once the reaction did not further evolve, they were worked up, purified, and characterized as 3-substituted-2-hydroxy-4-butyrolactones **5** and 4-substituted-2-hydroxy-4-butyrolactones derivatives **8** (Schemes 3, 4 and 5) (see the SI). The butyrolactones were formed during the lyophilization of the product after the anion exchange purification procedure in the presence of formic acid. This transformation was favored by the water elimination during the freeze-drying process. However, the efficiency of this process was limited, and as a consequence, another purification step was required, implying low isolated yields (Schemes 3, 4 and 5). Moreover, the yields were not improved by performing the lactonization in the presence of catalytic amounts of TsOH in toluene using a Dean–Stark apparatus. This step was not optimized neither the workup nor the purification processes.

The biocatalytic reduction of the aldol adducts always gave quantitative substrate conversions after 24 h of reaction either with DpkA_*Psyrin*_ or KPR_*Ecoli*_. Interestingly, KPR_*Ecoli*_ showed ample substrate tolerance on 3-substituted-4-hydroxy-2-oxoacids **3**, including those bearing *gem*-cycloalkyl quaternary centers **3k**–**n**, homologues to the natural substrate **3j**. An exception was **3i**, which was not converted (Schemes 3 and 4) probably due to the steric limitations imposed by the active-site cavity. DpkA_*Psyrin*_ tolerated all examples assayed of 4-substituted-4-hydroxy-2-oxoacids **6b**–**g**, whereas they were not substrates for KPR_*Ecoli*_ . DpkA_*Psyrin*_ has a more stringent substrate selectivity toward 3-substituted-4-hydroxy-2-oxoacids **3**, accepting only the unsubstituted **3a** or short C3-substituents such as methyl or ethyl, **3b** and **3c**, respectively, with a preference for those 3*S* configured.

The stereochemical outcome of the aldol addition of 2-oxoacids to formaldehyde has already been reported in previous studies on MBP-YfaU_*Ecoli*_, KPHMT_*Ecoli*_, and HBPA_*Pputida*_ catalysis.^9,10^ An identical stereochemical outcome was found for substrates **2e** and **2f**, which have not been previously reported. Compounds **5b**–**h** and **8b**–**g** contain one chiral center with known absolute stereochemistry defined by the aldolase.^9b,10^ This chiral center was used as a reference for the assessment of the overall relative configuration of these compounds by nuclear magnetic resonance (NMR) (see the SI). In addition, the absolute configuration for *R*-**5a** and *S*-**5a** was confirmed by comparing their specific rotation values with those reported (see the SI).^13^ Furthermore, authentic commercial samples and X-ray diffraction were used to unequivocally assign *R*-**5j**^13a^ (see the SI) and *R*-**5l** (Figure S33), respectively. The stereochemical configuration of *R*-**5k**, *R*-**5m**, and *R*-**5n** was inferred considering the high enantioselectivity observed for KPR_*Ecoli*_ found in the examples thereof. Thus, the absolute stereochemistry at C3 coming from the KPR_*Ecoli*_ reduction was mainly *R* and that from DpkA_*Psyrin*_ was preferentially *S*.

**Figure 2:**
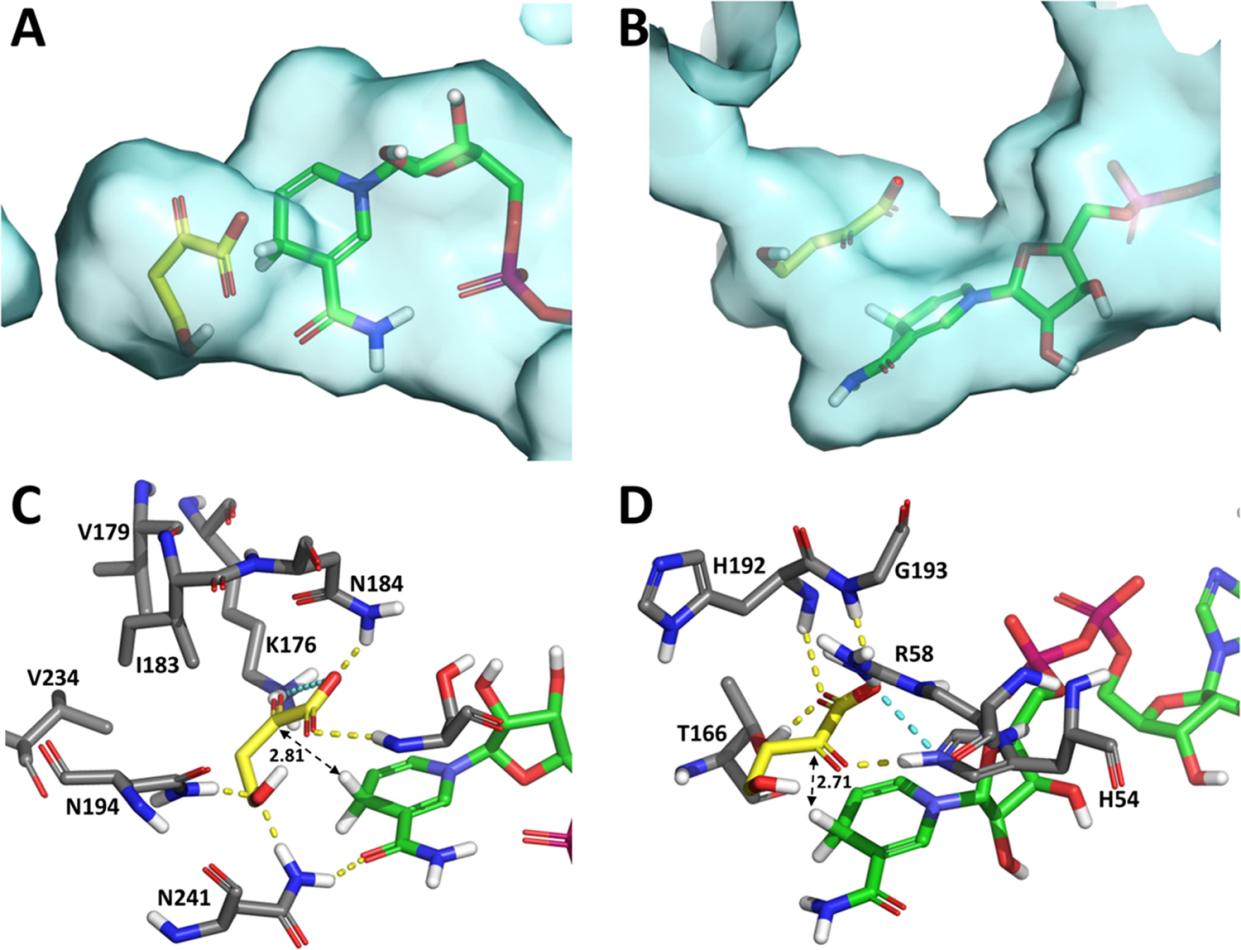
Molecular models of **3a** bound to KPR_*Ecoli*_ (A,C) and DpkA_*Psyrin*_ (B,D). These models were built starting from PDB structures 2OFP (A) and 2CWH(14) (B), and their structure was optimized by QM/MM methods (QM part optimized at the DFT B3LYP/6-31G** level of theory and MM part optimized with the OPLS2005 force field, see the SI). The substrate, NADPH, and close protein residues are shown with yellow, green, and gray C-atoms; H-bonds and salt bridges are shown with yellow and cyan dashed lines; the protein surface of the active-site cavities is shown in cyan (A,B); the distances between the reactive carbonyl C-atom and the NADPH pro-4*S* hydrogen are also displayed (C,D).

Molecular modeling of the 4-hydroxy-2-oxoacid substrates **3** bound to both reductases provided an explanation for these stereochemical results. Both enzymes are known to exist in open and closed forms, whose interconversion is triggered by substrate binding. In the closed forms, substrates and NADPH are buried in a deep and relatively narrow cavity (Figure 2A,B). The optimized energy model of 4-hydroxy-2-oxoacid **3a** bound into the active site of KPR_*Ecoli*_ shows that the pre-reactive conformation of the substrate is stabilized by multiple H-bond interactions (Figure 2C). Thus, the **3a** carboxylate accepts H-bonds from the 4-OH group (intramolecular), the amide group of Asn184, and the backbone-NH of Ser244, while the 4-OH group does the same from the sidechains of Asn194 and Asn241. In addition, the 2-oxo group is fixed by H-bonds with the sidechains of Asn98 and the catalytic Lys176, which is properly disposed to transfer its proton to the developing C2-kalkoxide, when reduction takes place. In this way, this 2-oxo group exposes its *si*-face to the nicotinamide moiety of the reduced NADPH cofactor, which delivers its pro-4*S* hydrogen to render the corresponding intermediate 2*R*-**4a**, the precursor of 2*R*-**5a**.^11c,11d^ Similar interactions and binding modes can be proposed for the rest of 4-hydroxy-2-oxoacid substrates **3** (Figure S69), which correlates with the observed 2*R*-stereochemical outcome for their KPR_*Ecoli*_ reduction products. On the other hand, the corresponding models with DpkA_*Psyrin*_ show that the carboxylate group of the substrates accepts H-bonds from the sidechains of Arg58 and Thr166, as well as from the backbone-NH of His192 and Gly193, and that it also establishes a salt bridge with the protonated guanidine group of Arg58 (Figures 2D and S70). In addition, the 2-oxo group accepts H-bonds from the 4-OH group (intramolecular) and from the protonated imidazole of His54, which acts as a general acid catalyst.^12^ This substrate binding mode forces the exposure of the *re*-face of the 2-oxo group to the reduced NADPH, rendering the 2*S*-**4** products, precursors of the 2-hydroxy-4-butyrolactones 2*S*-**5**. The same can be extended to DpkA_*Psyrin*_ substrates **6b**–**g** (Figure S70).

The degree of stereoselectivity of both reductases depended on the ketoacid. Thus, the 3*S*-configured 3-substituted-4-hydroxy-2-oxoacids (3*S*-**3**) gave very good diastereomeric ratios with KPR_*Ecoli*_ catalysis, i.e., 92:8 to 98:2 inferred from the (2*R*,3*S*):(2*S*:3*S*) ratios of compounds **5** (Scheme 3A). For the 3*R*-configured ones, the diastereomeric ratios were high toward **3b**, **3e**, and **3f** (93:7–96:4) and moderate with **3c** and **3g** (72:28–84:16) (Scheme 4A). A particular case was **3h** because the low dr (60:40) value was essentially due to the moderate 75% ee of the preceding aldol addition reaction. For the unsubstituted 4-hydroxy-2-oxoacid **3a** and the ones containing *gem*-cycloalkyl substituents, **3j**–**n**, excellent enantiomeric excesses >99% were achieved. DpkA_*Psyrin*_ gave excellent stereoselectivities with 2-oxoacids **3a**, **3b**, and **6b**–**d,g**, whereas 3*S*-**3c**, with a C3-ethyl substituent, **6e**, and **6f** rendered moderate diastereomeric ratios (87:13–89:11) (Schemes 3B and 5).

### Kinetic Analysis

The kinetics of the enzymatic reduction of 4-hydroxy-2-oxoacids with KPR_*Ecoli*_ and DpkA_*Psyrin*_ were determined using the products obtained from the aldol addition (Table 1). To avoid artifacts during the assay, the aldolase and metal excess were previously removed (see the SI).

**Table 1:**
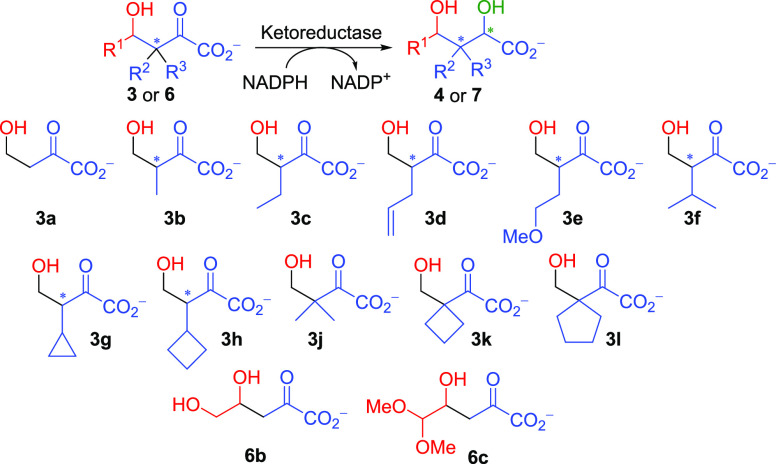
Kinetic Parameters for KPR_*Ecoli*_ and DpkA_*Psyrin*_ Catalyzed 2-Carbonyl Reduction of C3-Substituted 4-Hydroxy-2-oxoacids (**3** and **6**)^a^

substrate **3**	KPR_*Ecoli*_^b^				DpkA_*Psyrin*_^b^		
	*k*_cat_^app^	*K*_m_^app^	(*k*_cat_/*K_m_*)^app^	*k_si_*^app^	*k*_cat_^app^	*K_m_*^app^	(*k*_cat_/*K*_m_)^app^
**3a**	7.1 ± 0.5	4.9 ± 1.2	1.5 ± 0.4		1.7 ± 0.2	33 ± 7	0.052 ± 0.013
3*S***-3b**	348 ± 15	12.9 ± 1.4	27 ± 3		1.1 ± 0.1	19 ± 4	0.058 ± 0.012
3*S***-3c**	926 ± 37	3.0 ± 0.5	314 ± 52		0.23 ± 0.01	14 ± 2	0.016 ± 0.002
3*S***-3d**	89 ± 6	9.5 ± 1.7	9.4 ± 1.8				
3*S***-3f**	1021 ± 69	13 ± 2	77 ± 12				
3*S***-3g**	350 ± 20	15 ± 2	24 ± 4				
3*S***-3h**	820 ± 150	65.5 ± 13.4	13 ± 3	8.4 ± 1.8			
3*R***-3b**	5350 ± 138	4.8 ± 0.4	1109 ± 86		0.0024 ± 0.0002	7.5 ± 1.9	0.047 ± 0.013
3*R***-3c**	700 ± 34	7 ± 1	106 ± 18				
3*R***-3d**	90 ± 5	9.5 ± 1.5	9.4 ± 1.6				
3*R***-3e**	5.9 ± 0.5	18 ± 3	0.34 ± 0.10				
3*R***-3g**	87 ± 4	2.3 ± 0.4	37 ± 6				
3*R***-3h**	40 ± 4	11 ± 3	3.7 ± 1.0				
**3j**	4457 ± 813	0.4 ± 0.2	10,747 ± 4587	3.9 ± 1.3			
**3k**	6791 ± 1022	1.5 ± 0.5	4427 ± 1668	6.6 ± 1.5			
**3l**	1166 ± 67	0.2 ± 0.1	5569 ± 3737	28 ± 4			
4*R*-**6b**					3.00 ± 0.02	11 ± 1	0.28 ± 0.039
4-*R*-**6c**					84 ± 27^c^	97 ± 43^c^	0.87 ± 0.48

a *k*_cat_^app^ = min^–1^; *K*_m_^app^ = mM; (*k*_cat_/*K*_m_)^app^ = min^–1^ mM^–1^; *k*_si_ = mM.

b The kinetic parameters for KPR_*Ecoli*_ and DpkA_*Psyrin*_ were determined in a continuous assay method monitoring the oxidation of NADPH to NADP^+^ at 340 nm. The reactions were monitored during 15 min measuring every 30 s. The assay mixture (0.3 mL) consisted of 50 mM Tris–HCl buffer pH 8.0, containing NADPH (0.16 mM), aldol adducts (1–60 mM), and appropriate amounts of enzymes. One unit of activity was defined as the amount of ketoreductases that catalyzes the formation of 1 μmol NADP^+^ per min at 30 °C. Measurements were carried out in triplicate independent experiments. To determine the kinetic parameters, data were fitted to the Michaelis–Menten kinetic model using the software GraphPad Prism version 5.0 (see Figures S11–S32).

c Estimated parameters because the activity vs concentration curve did not reach a plateau within the range of concentrations studied (12–50 mM) (Figure S32).

As expected, the natural substrate of KPR_*Ecoli*_, i.e., ketopantoate **3j**, gave the highest specificity constant (*k*_cat_/*K*_m_)^app^, exceeding by 1 to 4 orders of magnitude those of the rest of the 2-oxoacids. The introduction of cyclobutyl (**3k**) and cyclopentyl (**3l**) moieties, similar to **3j**, gave (*k*_cat_/*K*_m_)^app^ values of the same order of magnitude as that of the natural substrate. It is noteworthy the inhibition of KPR_*Ecoli*_ by its natural substrate (**3j**) that has not been noticed in previous reports likely due to the limited range of substrate concentration analyzed.^11d^ Besides, in another publication on KPR from *Staphylococcus aureus*, an apparent substrate inhibition constant was reported to be around 270 μM, one order of magnitude lower than that found in this study for KPR_*Ecoli*_.^15^ As suspected, substrate inhibition was also detected for the corresponding cycloalkyl analogues **3k** and **3l**. Compared with the natural substrate, **3j**, the *k*_si_^app^ of the analogue bearing a cyclobutyl moiety, **3k**, increased 1.7-fold, whereas for the one with the cyclopentyl substituent, **3l**, it increased 7.3-fold. The impact of substrate inhibition, i.e., (*k*_si/_*K*_m_)^app^,^16^ is larger for the dimethyl and cyclobutyl analogues (**3k**, (*k*_si_/K_m_)^app^ = 4.3 ± 1.8) (**3j**, (*k*_si_/K_m_)^app^ = 9.3 ± 4.8) and much lower for the cyclopentyl (**3l**, (*k*_si_/K_m_)^app^ = 140 ± 91). This is consistent with the fact that **3k** and **3j** may have similar steric and electronic interactions with the enzyme, whereas **3l** is bulkier and might establish fewer interactions in the active site.

**Figure 3:**
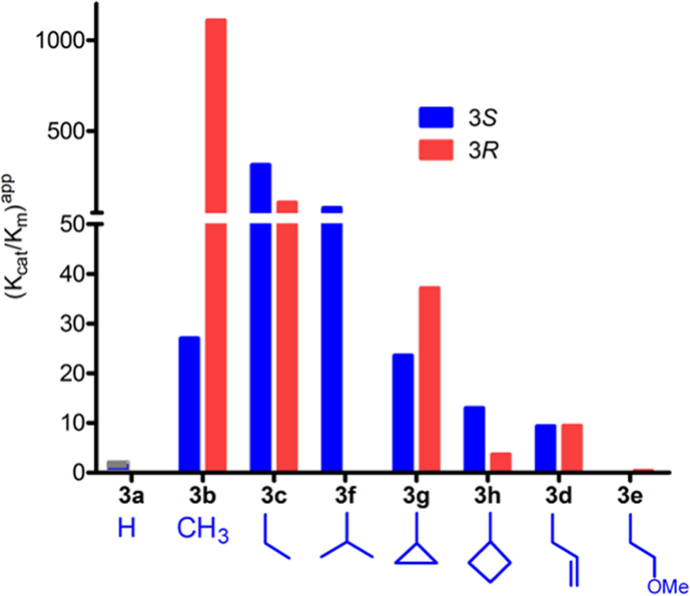
Values of (*k*_cat_/*K*_m_)^app^ for the KPR_*Ecoli*_ catalysis vs the C3-substituent structure of both enantiomers of the 4-hydroxy-2-oxoacids. The (*k*_cat_/*K*_m_)^app^ values were taken from Table 1 and are given in min^–1^.

The (*k*_cat_/*K*_m_)^app^ values of KPR_*Ecoli*_ for 3*S*-substitued-2-oxoacids increase with the size of the C3-alkyl chain up to two carbon atoms (3*S*-**3b** vs 3*S*-**3c**) and then decrease following the order of isopropyl (3*S*-**3f**), cycloalkyl (3*S*-**3g**, 3*S*-**3h**), and allyl (3*S*-**3d**) substituents (Table 1 and Figure 3). On the other hand, (*k*_cat_/*K*_m_)^app^ for the 3*R*-substituted-2-oxoacids decreased with the size of the C3-alkyl chain substituent being the methoxypropyl one with the lowest value. In the case of DpkA_*Psyrin*_, (*k*_cat_/*K*_m_)^app^ remained nearly constant for the 3-substituted-4-hydroxy-2-oxoacids 3*S*-**3b**,**c** and 3*R*-**3b** (Table 1). Kinetic parameters for DpkA_*Psyrin*_ using 4-substituted-4-hydroxy-2-oxoacids **6** indicate that 4*R*-**6b** and 4*R*-**6c** are around 10-fold better substrates than the 3-substituted-4-hydroxy-2-oxoacids. Unfortunately, kinetic parameters for 4*R*-**6d**–**g** could not be measured, owing to the unconverted pyruvate of aldol reactions (>20 mM) that strongly alters their *k*_cat_ and *K*_m_ values.

The stereochemical configuration of the C3-methyl substituent has strong influence on the (*k*_cat_/*K*_m_)^app^ for KPR_*Ecoli*_: 3*R*-**3b** functions 40-fold better than that of 3*S*-**3b** being the best one among the 3-substituted chiral substrates for KPR_*Ecoli*_ (Figure 3). In contrast, the 3*S*-isomers of **3c** and **3h** showed moderately better (*k*_cat_/*K*_m_)^app^ values than their 3*R*-homologues. The rest of the 3*R*-2-oxoacids gave similar results as compared to those of the *S*-configuration. The kinetic parameters of DpkA_*Psyrin*_ for 3*S*- and 3*R*-**3b** substrates indicate that the 3*S*-configuration was preferred for this enzyme (Table 1). Comparing both reductases within the same substrates assayed, KPR_*Ecoli*_ (*k*_cat_/*K*_m_)^app^ values are ca. 10^1^- to 10^4^-fold higher than those of DpkA_*Psyrin*_. On the other hand, 4-substituted-4-hydroxy-2-oxoacids **6b**–**g** were not substrates of KPR_*Ecoli*_.

It is interesting to note that in all cases, complete conversions were reached after 24 h, even though the much lower *k*_cat_^app^ values of some of the substrates, relative to **3j**.

### Enzymatic Cascade Synthesis

**Scheme 6:**
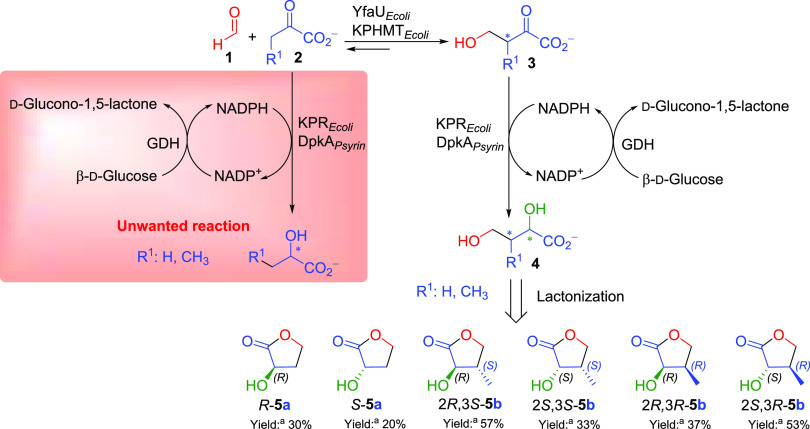
Enzymatic Cascade Process for the Synthesis of the Two Enantiomers of 2-Hydroxy-4-butyrolactones, *R*-**5a** and *R*-**5a**, and the Four Possible Stereoisomers of 3-Methyl-2-hydroxy-4-butyrolactones **5b** Isolated yields.

Results for the cascade process were successful in some examples with isolated yields ranging from 20 to 57% (Scheme 6). Additional examples were tested, but the results were unsuccessful, and no product formation was detected by HPLC. Instead, we observed that formaldehyde was not converted while the starting 2-oxoacid was consumed, indicating that it was reduced by the ketoreductase. Hence, we reasoned that the successful one-pot one-step process depends on the rates of aldol and retroaldol reactions and the 2-oxoacid reduction.

## Conclusions

A biocatalytic route for the synthesis of chiral 3- and 4-substituted-2-hydroxy-4-butyrolactone derivatives was developed. The methodology provides structurally diverse compounds from the achiral starting material in a stereodivergent fashion using stereocomplementary 2-oxoacid aldolases and ketoreductases as catalysts. A total of 33 substrates were tested for both reactions, furnishing 29 different 3- and 4-substituted 2-hydroxy-4-butyrolactones achieving conversions of aldol addition from 32 (only one example) to 98% and ketoreduction >95%, diastereomeric ratios from 60:40 to 98:2, and ee >99%. Apart from the aldol reaction, both KPR_*Ecoli*_ and DpkA_*Psyrin*_ gave quantitative conversions after 24 h of incubation, even for substrates with *k*_cat_^app^ values much lower as compared with **3j** in the case of KPR_*Ecoli*_. KPR_*Ecoli*_ showed broad substrate tolerance toward the C3-substituted 2-oxoacids, including those bearing *gem*-cycloalkyl quaternary centers, homologues to the natural substrate ketopantoate (**3j**). Moreover, substrate inhibition was observed for ketopantoate as well as for its *gem*-cycloalkyl homologues. On the other hand, KPR_*Ecoli*_ does not tolerate the 4-substituted-4-hydroxy-2-oxoacids **6**. Concerning DpkA_*Psyrin*_, it accepted all examples of 4-substituted-4-hydroxy-2-oxoacids (**6**), whereas it has stringent substrate selectivity for the 3-substituted-4-hydroxy-2-oxotoacids **4** accepting only methyl and ethyl C3-substituents, with a strong preference for the 3*S*-configured aldol adducts. Concerning the stereochemical preference, KPR_*Ecoli*_ gave 2*R*-configured 2-hydroxyacids, whereas DpkA_*Psyrin*_ furnished the corresponding 2*S* enantiomers. Finally, we conducted the synthesis of some 2-hydroxy-4-butyrolactones in a one-pot one-step (i.e., aldol addition + ketoreduction) reaction system. This was possible when the rate of the aldol addition reaction was much faster than that of ketoreduction of the starting 2-oxoacid. Under these conditions, the yields were similar to those achieved in a one-pot two-step fashion. Both methodologies are of practical value to carry out the synthesis of the corresponding products in 20–57% isolated yields.

## Supporting Information Available

General methods and protocols for the screening and synthesis, activity determinations, kinetics determination, synthesis of starting materials, synthesis of 2-hydroxy-4-butyrolactone derivatives, NMR spectra, and computational methods and modeling (PDF)
