# Supplementary material for: Biocatalytic Synthesis of Homochiral 2-Hydroxy-4-butyrolactone Derivatives by Tandem Aldol Addition and Carbonyl Reduction
Source: ACS Catal. 2023 Apr 6;13(8):5348–57. doi: 10.1021/acscatal.3c00367 (PMC10127515; doi:10.1021/acscatal.3c00367)
Supplement: Supplementary file 1 — cs3c00367_si_001.pdf [file cs3c00367_si_001.pdf]

Supporting Information

Biocatalytic synthesis of homochiral 2-hydroxy-4-butyrolactone derivatives by tandem aldol addition and carbonyl reduction.

Carlos J. Moreno[a], Karel Hernández[a], Samantha Gittings[b], Michael Bolte[c], Jesús Joglar[a], Jordi Bujons[a], Teodor Parella[d], Pere Clapés*[a]

[a]Catalonia Institute for Advanced Chemistry, Dept. Chemical Biology & Molecular Modelling, IQAC-CSIC, Spain.

[b]Prozomix Ltd. West End Industrial Estate, Haltwhistle, Northumberland, NE49 9HA, UK.

[c]Institut für Anorganische Chemie, J.-W.-Goethe-Universität, Frankfurt/Main, Germany.

[d]Servei de Ressonància Magnètica Nuclear. Universitat Autònoma de Barcelona, Bellaterra, Spain.

*Corresponding author: pere.clapes@iqac.csic.es

Table of Contents

Materials. 2

Methods. 4

Protein production and purification. 5

Thin layer chromatography (TLC) analysis. 7

Specific rotation. 7

HPLC analysis. 7

NMR analysis. 8

Activity determination of $DpkA_{Psyrin}$. 8

Activity determination of KPR_{Ecoli}. 9

Activity determination of glucose dehydrogenase (GDH). 10

Biocatalytic aldol addition of 2-oxoacid (2) to formaldehyde (1). Analytical scale 11

One-pot two-step stereoselective synthesis of 2-hydroxy-4-butyrolactone derivatives (5). 13

One-pot enzymatic cascade for the stereoselective synthesis of 2-hydroxy-4-butyrolactones derivatives (5a,b). 24

Strategy for enantiomeric excess determination of products 5 (a, j-n). 25

Biocatalytic aldol addition of sodium piruvate (2a) to aldehydes (1b-g). Analytical scale 33

One-pot two-step stereoselective synthesis of 4-substituted-2-hydroxy-4-butyrolactones 8 by tandem biocatalytic aldol-reduction reactions catalyzed by $HBPA_{Pputida}$ and $DpkA_{Psyrin}$. 35

Kinetic Studies. 39

X-Ray structures 52
NMR spectra... 56
Computational Methods. 148

Materials.

Formaldehyde (**1**), glycolaldehyde dimer (**1b**), 2,2-dimethoxyacetaldehyde (**1c**), chloroacetaldehyde (**1c**), benxiloxiacetaldehyde (**1d**), sodium pyruvate (**2a**), 2-oxobutyric acid (**2b**), 2-oxopentanoic acid (**2c**), 4-Methyl-2-oxovaleric acid (**2f**), sodium 3-methyl-2-oxobutyrate (**2j**), were purchased from Sigma-Aldrich. The rest of aldehyde and 2-oxoacids used in this study were synthesized in our lab using procedure described in previous works[1-3]. Stock solutions of 2-oxoacids were prepared in H_2O, pH adjusted to 7.0 with 1 M NaOH and stored at 4 °C. Under these conditions precipitation of sodium salt compounds (**2g-n**) was observed. Before using, these solutions were carefully treated with the heat gun under stirring until a clear solution was obtained (Table S1).

Table S1: Substrates (2a-n) stock solution.

2-oxoacids (**2**)	Stock concentration/M
**2a**	2.0
**2b**	1.0
**2c**	1.8
**2d**	2.0
**2e**	1.0
**2f**	0.65
**2g**	1.5
**2h**	1.0
**2i**	0.8
**2j**	1.0
**2k**	1.0
**2l**	1.0
**2m**	0.65
**2n**	0.44

The 3-benzyloxyamine hydrochloride, D-(−)-pantolactone and (*R*)-(+)-α-Hydroxy-γ-butyrolactone were purchased from Sigma-Aldrich. Sodium borohydride DL-(−)-pantolactone and 4-bromobenzoyl chloride were purchased from TCI chemical. NADPH was purchased from CARL ROTH. Glucose dehydrogenase (GDH) as a cell

free extract powder and $NADP^+$ were provided by Prozomix Ltd (PRO-GDH(001)). All reagent for Molecular Biology were supplier from ThermoFischer Scientific.

Synthetic oligonucleotides were purchased from Eurofins Genomics. All reagents for molecular biology were from Life Thermo Scientific. Culture media components for *E. coli* were from Pronadisa (Madrid, Spain). Antibiotics and IPTG were from Carl Roth. Nickel Sepharose™ High Performance was from Cytiva™. Water for analytical HPLC was obtained from an Arium pro ultrapure water purification system (Sartorius Stedim Biotech) and the rest of solvents used in this work were of analytical grade or HPLC grade. Bacterial strains, oligonucleotides and plasmids used in this study are listed in Table S2.

Table S2. Strains, plasmids and oligonucleotides used in this study.

**Strains**	**Relevant genotype**
E. coli Nova Blue (**Novagen ®**) (used for plasmid preparation).	*end*A1, *hsd*R17 (rB^+, mB^+), *sup*E44, *thi*1, *rec*A1, *gyr*A96, *rel*A1, *lac* F´ [proA+ B+, *lac*I^q ZΔM15::Tn10] (*Tet*^{R}).
E coli M15 [pREP4] (used for protein expression).	*nal*^{s} , *str*^{s} , *rif*^{s} , *thi*$^-$, *lac*$^-$, *ara*$^+$, *gal*$^+$, *mtl*$^-$, F^- , *recA*$^+$, *uvr*$^+$, *lon*$^+$, Km^R.[4]
E. coli Bl21-AI (used for protein expression)	F^- *ompT gal dcm lon* $hsdS_B(r_B^- m_B^-)[malB^+]_{K-12}(\lambda^S)$ *araB*:*T7RNAP-tetA*
**Oligonucleotides**	**Sequences**
Primer dpka$_{Psyrin}$ (53)	Forward:5'ATTATAATCCATGGGCTGTCCGCCAGCCACGCTGACCAGCCC3' *Nco*I
Primer dpka$_{Psyrin}$ (35)	Reverse: 3' ATTATAATAGATCTGTGGCCTGCCAGCTCTTGCAAAC 5' *Bgl*II
Primer panE$_{Ecoli}$ (53)	Forward: 5' ATATATATGGATCCATGAAAATTACCGTATTGGGATGC 3' *BamH*I
Primer panE$_{Ecoli}$ (35)	Reverse:5'ATATATATAAGCTTCTACCAGGGGCGAGGCAAACC 3' *Hind*III
**Plasmids**	**Relevant genetic characteristics**
pQE-60	The vector encodes one multiple cloning site preceded by a T5 promoter, *lac* operator and ribosome binding site. The vector also carries the ColE1 replicon and ampicillin resistance gene [4].
pQE-40	The vector encodes one multiple cloning site preceded by a T5 promoter, *lac* operator and ribosome binding site. The vector also carries the ColE1 replicon and ampicillin resistance gene[4].
pQE-40-*panE*	panE gene (912 bp) from *E. coli K-12* (NCBI data base accession number CP015085.1) cloned in pQE40 (*BamH*I and *Hind*III).
pQE-60-*dpka*	dpka gene (1032 bp) from *Pseudomonas syringae* (NCBI data base accession DSM50315) cloned in pQE60 (*Nco*I and *Bgl*II).
pQE60 *panB wt*<br>pQE60 *panB I202A*<br>pQE60 *panB I212A*	Previous work.[1-2]
pQE40 *malE-rmhA wt*	Previous work.[5-6]
pQE40 *malE-rmhA* W23V	Previous work.[7]
pETDuet-1- *HBPA*	Previous work.[3]
pETDuet-1- *HBPA H205A*	Previous work.[3]

Methods.

Genes cloning.

The *dpka* gene (NCBI data base accession number DQ017704.1) was amplified from genomic DNA of *Pseudomonas syringae* van Hall 1902 (DSM No: 50315) using de primers: dpka$_{Psyrin}$ (53) and dpka$_{Psyrin}$ (35). The amplicon (1032 bp) was cloned in the into a pQE 60 plasmid with the restriction enzymes FastDigest^{TM} *Nco*I and *Bgl*II. All DNA manipulation were performed using routine procedures of molecular biology according to the manufacturer´s protocols (Sequence S01 and Sequence S02).

SEQUENCE S01. Base sequence of the *dpka* gene(blue) insert into a pQE-60.

*Nco*I

CCATGGATGGGCATGTCCGCCAGCCACGCTGACCAGCCCACTCAAACCGTTTCTTACCCGCAACTGATCGACCTGCTG
CGCCGGATTTTCGTGGTCCACGGGACCTCACCGGAGGTCGCCGATGTACTCGCTGAAAACTGCGCCAGTGCCCAGCG
CGACGGTTCGCACAGTCATGGCATCTTTCGCATTCCCGGCTATCTGTCTTCGTTGGCCAGCGGTTGGGTGGATGGCAA
GGCGGTGCCGGTGGTCGAGGATGTCGGCGCGGCATTTGTCAGGGTCGATGCTTGCAACGGCTTTGCCCAGCCGGCAC
TGGCGGCGGCCAGGTCACTGTTGATCGATAAGGCGCGCAGTGCCGGGGTTGCGATTCTGGCCATTCGGGGCTCGCAT
CATTTCGCCGCGCTGTGGCCAGATGTCGAACCGTTTGCCGAGCAGGGGCTGGTTGCGCTGAGCATGGTCAACAGCAT
GACCTGCGTTGTACCCCACGGTGCTCGGCAGCCCTTGTTCGGCACCAACCCGATCGCCTTCGGTGCGCCGCGCGCAGG
TGGCGAGCCCATCGTTTTGACCTGGCCACCAGCGCCATTGCCCACGGTGACGTGCAGATTGCTGCGCGTGAAGGGC
GGTTGCTGCCGGCGGGCATGGGTGTTGATCGTGACGGGCTGCCGACGCAGGAGCCTCGCGCCATTCTCGACGGCGGG
GCGCTGTTGCCGTTCGGCGGGCACAAGGGCTCGGCATTGTCGATGATGGTCGAGTTGCTGGCGGCAGGGCTGACCGG
TGGCAATTTCTCGTTCGAGTTCGACTGGTCGAAGCATCCCGGCGCACAGACGCCCTGGACCGGCCAGTTGCTGATCGT
CATTGATCCCGACAAAGGTGCCGGTCAGCACTTTGCGCAGCGCAGCGAGGAACTGGTGCGCCAGCTTCACGGGGTCG
GCCAGGAACGATTGCCCGGCGACAGGCGTTACCTGGAGCGGGCACGCTCGATGGCGCACGGAATAGTCATTGCCCAG
GCCGACCTTGAGCGTTTGCAAGAGCTGGCAGGCCACAGATCTCATCACCATCACCATCACTAA

*Bgl*II

SEQUENCE S02. Amino acid sequence of protein construct. Starting methionine incorporated by open reading frame in pQE-60, Δ1-pyrroline-2-carboxylate reductase (DpkA$_{Psyrin}$, EC 1.5.1.21) and 6x His tag (red).

MGMSASHADQPTQTVSYPQLIDLLRRIFVVHGTSPEVADVLAENCASAQRDGS
HSHGIFRIPGYLSSLASGWVDGKAVPVVEDVGAAFVRVDACNGFAQPALAAAR
SLLIDKARSAGVAILAIRGSHHFAALWPDVEPFAEQGLVALSMVNSMTCVVPH
GARQPLFGTNPIAFGAPRAGGEPIVFDLATSAIAHGDVQIAAREGRLLPAGMGV
DRDGLPTQEPRAILDGGALLPFGGHKGSALSMMVELLAAGLTGGNFSFEFDWS
KHPGAQTPWTGQLLIVIDPDKGAGQHFAQRSEELVRQLHGVGQERLPGDRRYL
ERARSMAHGIVIAQADLERLQELAGHRSHHHHHH*

The *panE* gene (NCBI data base accession number CP015085.1) was amplified from genomic DNA of *E. coli* K-12 using de primers: panE$_{Ecoli}$ (53) and panE$_{Ecoli}$ (35). The amplicon (912 bp) was cloned into a pQE-40 plasmid with the restriction enzymes FastDigest^{TM} *BamH*I and *Hind*III. All DNA manipulation were performed using routine procedures of molecular biology according to the manufacturer´s protocols (Sequence S03 and Sequence S04).

SEQUENCE S03. Bases sequence of the *panE* gene (blue) insert in pQE-40

*BamH*I

ATGAGAGGATCGCATCACCATCACCATCACGGATCCATGAAAATTACCGTATTGGGATGCGGTGCCTTAGGGCAATT
ATGGCTTACAGCACTTTGTAAACAGGGTCACGACGTTCAGGGCTGGCTGCGCGTACCGCAACCTTATTGTAGTGTGAA
TCTGGTTGAGACAGATGGTTCGATATTTAATGAATCGCTGACCGCCAACGATCCCGATTTTCTCGCCACCAGCGATCT
GCTCCTGGTGACGCTGAAAGCATGGCAGGTTTCCGATGCCGTCAAAAGCCTCGCGTCCACACTGCCTGTAACCACGC
CAATACTGTTAATTCACAACGGCATGGGCACCATCGAAGAGTTGCAAAACATTCAGCAGCCATTACTGATGGGCACC
ACCACCCATGCCGCCCGCCGCGACGGCAATGTCATTATTCATGTGGCAAACGGTATCACGCATATTGGTCCGGCACG
GCAACAGGACGGCGATTACAGTTATCTGGCGGATATTTTGCAAACCGTATTGCCTGACGTCGCGTGGCATAACAATA
TTCGCGCCGAGCTGTGGCGCAAGCTGGCAGTCAACTGTGTGATTAATCCACTGACCGCCATCTGGAATTGCCCGAATG
GTGAATTACGTCATCATCCGCAAGAAATTATGCAGATATGCGAAGAAGTCGCGGCAGTGATCGAACGCGAAGGGCAT
CATACTTCAGCAGAAGATTTGCGTGATTACGTGATGCAGGTGATTGATGCAACAGCGGAAAATATCTCGTCGATGTT
GCAGGATATCCGCGCGCTGCGCCACACCGAAATCGACTATATCAATGGTTTTCTCTTACGCCGCGCCCGCGCGCATGG
GATTGCCGTACCGGAAAACACCCGCCTGTTTGAAATGGTAAAAAGAAAGGAGAGTGAATATGAGCGCATCGGCACT
GGTTTGCCTCGCCCCTGGTAGAAGCTT

*Hind*III

SEQUENCE S04. Amino acid sequence of protein construct. Starting methionine incorporated by the open reading frame in pQE-40, ketopantoate reductase (KPR$_{Ecoli}$, EC 1.1.1.169, blue) and 6x His tag (red).

MRGSHHHHHHGSMKITVLGCGALGQLWLTALCKQGHDVQGWLRVPQPYCSV
NLVETDGSIFNESLTANDPDFLATSDLLLVTLKAWQVSDAVKSLASTLPVTTPIL
LIHNGMGTIEELQNIQQPLLMGTTTHAARRDGNVIIHVANGITHIGPARQQDGD
YSYLADILQTVLPDVAWHNNIRAELWRKLAVNCVINPLTAIWNCPNGELRHHP
QEIMQICEEVAAVIEREGHHTSAEDLRDYVMQVIDATAENISSMLQDIRALRHT
EIDYINGFLLRRARAHGIAVPENTRLFEMVKRKESEYERIGTGLPRPW*

Protein production and purification.

General procedure for DpkA$_{Psyrin}$ and KPR$_{Ecoli}$: Home-made chemically competent cells (*E. coli* M-15[pREP-4] strain from QIAGEN)[8] were transformed with the corresponding plasmid and grown in LB plate with ampicillin (100 µg mL^{-1}) plus kanamycin (25 µg mL^{-1}) at 37 ºC overnight. A bacterial plaque scraping was transferred into a baffled shaker flask (100 mL) containing LB medium (30 mL) plus ampicillin (100 µg mL^{-1}) and kanamycin (25 µg mL^{-1}), and grown at 37 °C on a rotary shaker at 200 rpm overnight (OD$_{600}$ ≈ 2-3 after the growth). An aliquot of the pre-culture (20 mL) was transferred into a baffled shaker flask (2 L) containing LB medium (1 L) plus ampicillin (100 µg mL^{-1}), kanamycin (25 µg mL^{-1}) and antifoam SE-15 (0.02% v/v). The culture was incubated at 37 °C with shaking at 200 rpm. During the middle exponential phase growth (DO$_{600}$ ≈ 0.5-0.8), the temperature was lowered and proteins expression were induced with IPTG (1 mM). After 12-16 h, the culture (5 L) were centrifuged (2500 *g* for 45 min at 4 °C) and the pellet of cells (16-22 g) was re-suspended in the lysis buffer (400 mL, 50 mM NaH_2PO_4 buffer pH 8.0, 300 mM NaCl, and 10 mM imidazole). Cells were lysed using a cell disrupter (Constant Systems) and cellular debris were removed by centrifugation (35000 *g* for 45 min at 4 °C). The clear supernatant was applied to a cooled HR 16/40 column (Cytiva™) packed with Nickel

Sepharose™ High Performance (50 mL bed volume, Cytiva™) at 3 mL min^{-1} and washed with lysis buffer (400 mL) at 6 mL min^{-1}. The protein was eluted with elution buffer (200 mL, 50 mM NaH_2PO_4 buffer pH 8.0, 300 mM NaCl, and 500 mM imidazole) at a flow rate of 6 mL min^{-1} (Figure S1A and B). The purified protein was dialyzed against dialysis buffer (3x1 L 24 h each, 20 mM TEA buffer pH 6.5, 100 mM NaCl, and 50% (v/v) of glycerol). The dialyzed solution obtained (95-135 mL) was stored at – 20 °C. Protein concentrations were determined by absorption at 280 nm using extinction coefficients calculated by ProtParam (https://web.expasy.org/protparam/), DpkA$_{Psyrin}$ (Abs 0.1% (= 1 g L^{-1}) 0.709), and KPR$_{Ecoli}$ (Abs 0.1% (= 1 g L^{-1}) 1.35) assuming all Cys residues are reduced (Table S3).

General procedure for GDH: The enzyme, provided by Prozomix Ltd (PRO-GDH(001)) as a cell free extract powder (6 g), was re-suspended in the lysis buffer (400 mL, 50 mM NaH_2PO_4 buffer pH 8.0, 300 mM NaCl, and 10 mM imidazole). Cellular debris was removed by centrifugation (35000 *g* for 45 min at 4 °C). The enzyme was purified following the protocol indicated above (Figure S1C) The purified protein was dialyzed against dialysis buffer (3x1 L 24 h each, 10 mM HEPES buffer pH 6.5, 100 mM NaCl, and 50% (v/v) of glycerol). The dialyzed solution obtained (90-120 mL) was stored at – 20 °C. Protein concentration was determined using Pierce® 660 nm Protein Assay Reagent (Thermo Scientific) in 96 wells plate according to the manufacturer´s protocols (Table S3).

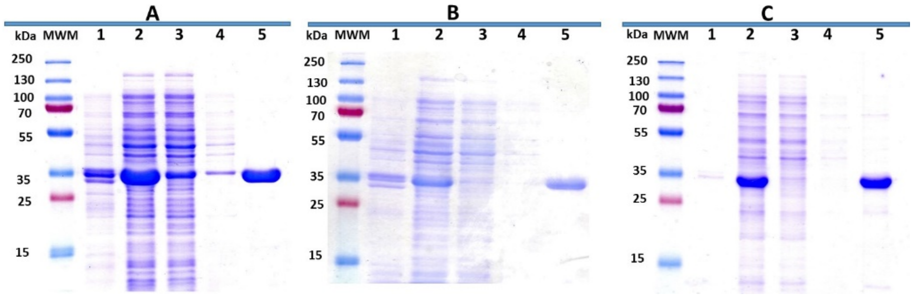

Figure S1: Analysis of enzyme purification steps by Coomassie Blue-stained SDS-PAGE. DpkA$_{Psyrin}$ (**A**), KPR$_{Ecoli}$ (**B**). GDH (**C**). In each case, the gel was loaded with samples from pellet after lysis (lane 1), supernatant of lysis (lane 2), flow-through fraction (lane 3), wash fraction (lane 4) and elution fraction (lane 5). The molecular masses of the proteins in the Standard Molecular Weight Marker are as indicated. The predicted molecular mass of DpkA$_{Psyrin}$, and KPR$_{Ecoli}$ are 37.6 kDa and 35.3 kDa respectively.

General procedure for YfaU$_{Ecoli,}$ HBPA$_{Pputida}$ and KPHMT$_{Ecoli}$ expression and purification were performed as describe in previous works [1, 3, 5]

Table S3. Typical enzyme concentration obtained in this work.

**Enzymes**	**Concentration**/ mg mL^{-1}
KPHMT$_{Ecoli}$ *wt*	9.3
KPHMT$_{Ecoli}$ I202A	7.7
KPHMT$_{Ecoli}$ I212A	8.5
MBP-YfaU$_{Ecoli}$ *wt*	0.5[a]
MBP-YfaU$_{Ecoli}$ W23V	0.5[a]
HBPA$_{Pputida}$	4.0 (0.029 U mL^{-1}U mL^{-1})[b]
HBPA$_{Pputida}$ H205A	3.8 (0.009 U mL^{-1}U mL^{-1})[b]
DpkA$_{Psyrin}$	4.0 (2.6 10^{-2} U mL^{-1})[c]
KPR$_{Ecoli}$	4.0 (52 U mL^{-1})[c]
GDH	5.2 (20.8 U mL^{-1})[c]

[a]mg protein mL^{-1} lyophilized powder. [b]Enzyme activity assays were described in previous work[3]. [c]Enzyme activity assays are described below

Thin layer chromatography (TLC) analysis.

TLC analysis was performed using precoated silica gel plates with or without fluorescent indicator UV254 (Macherey-Nagel GmbH & Co. KG, Kieselgel 60). TLC without fluorescent visualization was stained using ceric ammonium molybdate or potassium permanganate.

Specific rotation.

Specific rotation values were measured with a Perkin Elmer Model 341 (Überlingen, Germany) (Na Lamp, 589 nm). Products (5 to 20 mg) were dissolved in MeOH (1.5 mL) and the samples were analyzed at room temperature 1.0 dm cell with polarized light (Na D line 589 nm) at 25 ºC. Rotation values are described in each compound.

HPLC analysis.

a) HPLC analysis was performed on a RP-HPLC XBridge® C18, 5 µm, 4.6 × 250 mm column (Waters). The solvent system used was: solvent (A): 0.1% (v/v)

trifluoroacetic acid (TFA) in H_2O and solvent (B): 0.095% (v/v) TFA in CH_3CN/H_2O 4:1, flow rate 1 mL min^{-1}, detection at 215 nm and column temperature at 30 °C.

The amount of substrates and products were quantified from the peak areas using an external standard methodology. Reaction monitoring for formaldehyde (**1**), ketoacids (**2**) and aldol adduct (**3**) (**Scheme S1)** were carried out as follows: samples were withdrawn from the reaction mixture (10 µL) and mixed with a solution of $BnONH_2$ (50 µL of a 0.13 M stock solution in pyridine:methanol:water 33:15:2), and then diluted with methanol (500 µL). After centrifugation, samples were analyzed by HPLC. Elution conditions: gradient from 10 to 100% B over 30 min (reaction with compounds **2a-l**) and 10 to 100% B over 60 min (reaction with compounds **2m**, **n**).

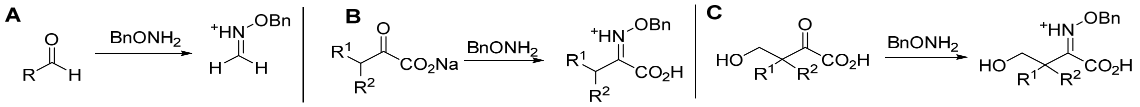

1 2 3

Area= 10^6 C(1a/mM), R^2=0.99, $\lambda = 215$nm Area= 10^6 C(2a/mM), R^2=0.99, $\lambda = 215$nm

Scheme S1. Pre-column derivatization of substrates (**A** and **B**) and products (**C**) bearing carbonyl groups with *O*-benzylhydroxilamine.

b) Enantiomeric excesses were analyzed by HPLC on a CHIRALPAK® ID, column (46 x 250 mm, 5 µm). The solvent system used was: solvent (A): Hexane and solvent (B): Isopropanol, detection by diode array detection (215-350 nm) and column temperature at 30 °C.

NMR analysis.

Routine ^{1}H (400 MHz) and ^{13}C (101 MHz) NMR spectra of compounds were recorded with a Varian Mercury-400 spectrometer. Full characterization of the described compounds was performed using typical gradient-enhanced 2D experiments: COSY, HSQC, NOESY and HMBC recorded under routine conditions.

Activity determination of DpkA*Psyrin*.

The ketoreductase activity of the DpkA*Psyrin* was determined in a continuous assay method monitoring the oxidation of NADPH to $NADP^+$ at 340 nm (NADPH ε_{340} = 6.22 mM^{-1} cm^{-1}) using sodium pyruvate as substrate. The reactions were monitored during 15 min measuring each 30 s. The assay mixture (0.3 mL) consisted of 50 mM Tris-HCl

buffer pH 8.0, containing NADPH (0.16 mM), sodium pyruvate (40 mM) and appropriate amounts of enzyme (0.19 mg mL^{-1} to 3.0 mg mL^{-1}). One unit of activity was defined as the amount of $DpkA_{Psyrin}$ that catalyzes the formation of 1 μmol $NADP^+$ per min at 30 °C (Figure S2). Units: 6.5 10^{-3} U mg^{-1}.

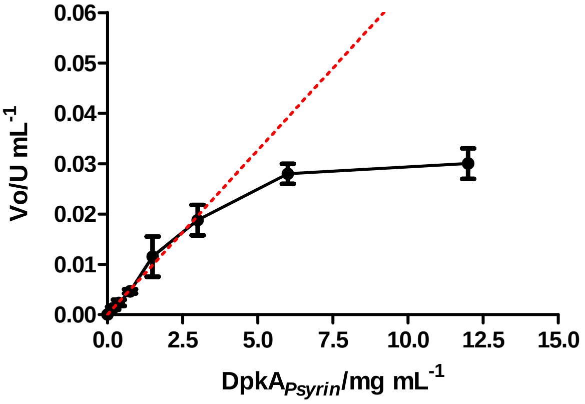

Figure S2. Variation of the ketoreductase activity of the $DpkA_{Psyrin}$ with the enzyme concentrations. Enzymatic specific activity (typically 6.5 10^{-3} U mg^{-1}) was calculated as the slope of the plot of V_0 vs. [$DpkA_{Psyrin}$] in the linear region. One unit of activity was defined as the amount of $DpkA_{Psyrin}$ that catalyzes the formation of 1 μmol $NADP^+$ per min at 30 °C.

Activity determination of KPR_{Ecoli}.

The ketoreductase activity of the KPR_{Ecoli} was determined in a continuous assay method monitoring the oxidation of NADPH to $NADP^+$ at 340 nm (NADPH ε_{340} = 6.22 mM^{-1} cm^{-1}) using sodium 4-hydroxy-3,3-dimethyl-2-oxobutanoate as substrate (synthesized as described below). The reactions were monitored during 15 min measuring each 30 s. The assay mixture (0.3 mL) consisted of 50 mM Tris-HCl buffer pH 8.0, containing NADPH (0.16 mM), sodium 4-hydroxy-3,3-dimethyl-2-oxobutanoate (50 mM) and appropriate amounts of enzyme (0.48 μg mL^{-1} to 7.81 μg mL^{-1}). One unit of activity was defined as the amount of KPR_{Ecoli} that catalyzes the formation of 1 μmol $NADP^+$ per min at 30 °C (Figure S3). Units: 13 U mg^{-1}.

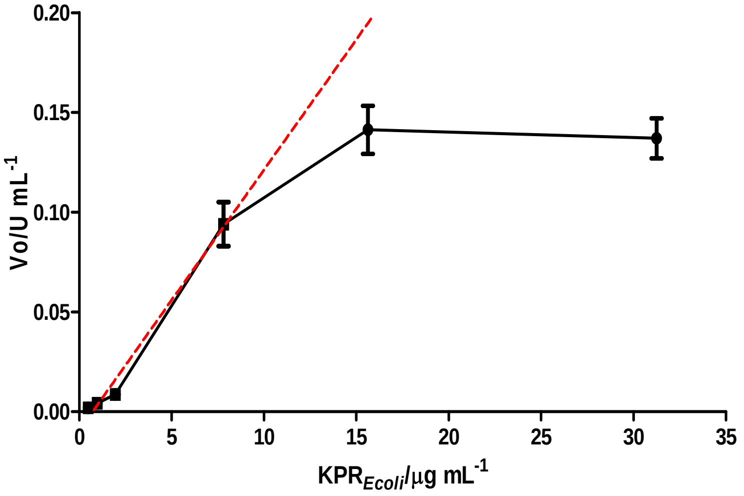

Figure S3. Variation of the ketoreductase activity of the KPR$_{Ecoli}$ with the enzyme concentrations. Enzymatic specific activity (typically 13 U mg^{-1}) was calculated as the slope of the plot of V_0 vs. [KPR$_{Ecoli}$] in the linear region. One unit of activity was defined as the amount of KPR$_{Ecoli}$ that catalyzes the formation of 1 µmol NADP$^+$ per min at 30 °C.

Activity determination of glucose dehydrogenase (GDH).

The dehydrogenase activity of the purified GDH (PRO-GDH (001)) was determined in a continuous assay method monitoring the reduction of NADP$^+$ to NADPH at 340 nm (NADPH ε_{340} = 6.22 mM^{-1} cm^{-1}) using glucose as substrate. The reactions were monitored during 15 min measuring each 30 s. The assay mixture (0.3 mL) consisted of 100 mM Tris-HCl buffer pH 8.0, containing NADP$^+$ (0.25 mM), glucose (30 mM) and appropriate amounts of enzyme (1.9 µg mL^{-1} to 62.5 µg mL^{-1}). One unit of activity was defined as the amount of GDH that catalyzes the formation of 1 µmol NADPH per min at 30 °C (Figure S4). Units: 4 U mg^{-1}.

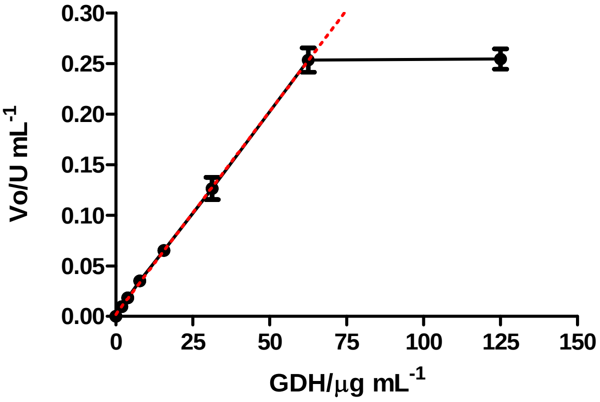

Figure S4. Variation of the dehydrogenase activity of the GDH with the enzyme concentrations. Enzymatic specific activity (typically 4 U mg^{-1}) was calculated as the slope of the plot of V_0 vs. [GDH] in the linear region. One unit of activity was defined as the amount of GDH that catalyzes the formation of 1 μmol NADPH per min at 30 °C.

Biocatalytic aldol addition of 2-oxoacid (2) to formaldehyde (1). Analytical scale

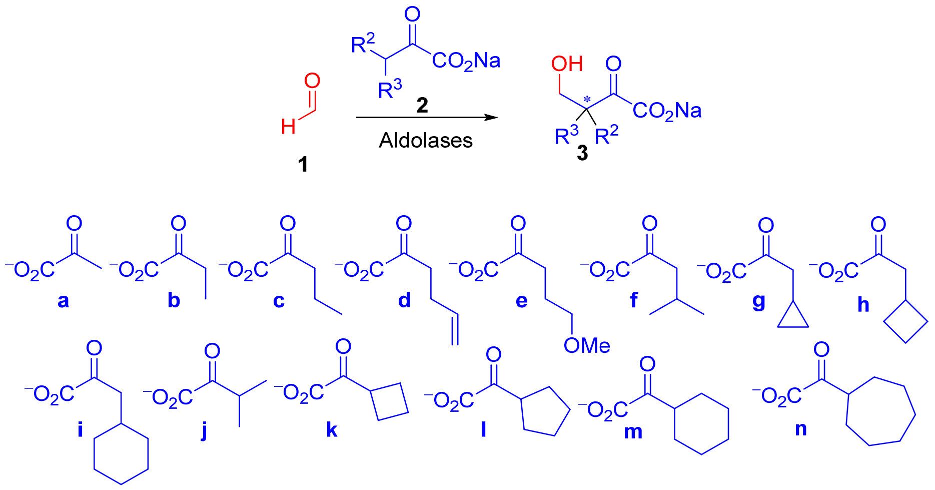

Scheme S2. Biocatalytic aldol addition of 2-oxoacids (**2a-n**) to formaldehyde (**1a**) catalyzed by YfaU$_{Ecoli}$. and KPHMT$_{Ecoli}$.

Aldol addition of 2a-i to 1[9] catalyzed by *S*-stereoselective YfaU$_{Ecoli}$ wt and YfaU$_{Ecoli}$ W23V.

Reactions were carried out at analytic level as follows: The reactions (500 μL total volume) were conducted in Eppendorf tubes (1.5 mL) and placed in a vortex mixer (100 rpm) at 25ºC. To a solution of YfaU$_{Ecoli}$ wt or YfaU$_{Ecoli}$ W23V enzymes (4 mg of lyophilized powder at 0.5 mg protein mg^{-1} lyophilized powder, 2 mg of protein, 2 mg protein mL^{-1} final concentration in the reaction,) in plain water, $NiCl_2$ (5 μL of a 0.1 M

stock solution in water, 1 mM in the reaction), and the 2-oxoacids (**2a-i**, 0.5-2 M stock solution in water pH 7.0, 1 mmol, 1 eq, 0.1 M in the reaction) were added. Reactions were started by adding formaldehyde (**1a**, 50 μL of a 1 M in water solution, 1 eq, 0.1 M in the reaction). Samples were withdrawn immediately after the addition of formaldehyde (0 h) and at 24 h and analyzed by HPLC as described above.

Aldol addition of 2a-n to 1[9-10] catalyzed by *R*-stereoselective KPHMT$_{Ecoli}$ and variants.

Reactions were carried out at analytic level as follows: The reactions (500 μL total volume) were conducted in Eppendorf tubes (1.5 mL) and placed in a vortex mixer (100 rpm) at 25ºC. To a solution of KPHMT$_{Ecoli}$ wt or variants (1 mg protein mL^{-1} in the reaction, from a protein stock solutions in 20 mM TEA buffer pH 7.0, 100 mM NaCl, and 50% (v/v) of glycerol) in plain water, $CoCl_2$ (5 μL of a 0.1 M stock solution, 1 mM in the reaction), and the 2-oxoacids (**2a-n**, 0.5-2 M stock solution in water pH 7.0, 1 mmol, 1 eq, 0.1 M in the reaction) were added. The reaction was started by adding formaldehyde (**2**, 50 μL of a 1 M in water solution, 1 eq, 0.1 M in the reaction). Samples were withdrawn immediately after the formaldehyde addition (0 h) and after 24 h and analyzed by HPLC as described above.

Biocatalytic reduction of 4-hydroxy-2-oxacids (3a-n). Substrate scope of ketoreductases. Analytical scale.

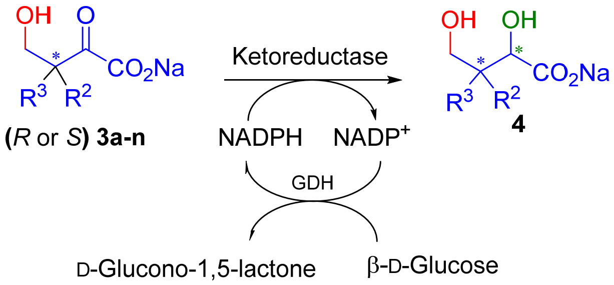

Scheme S3. Biocatalytic reduction of aldol adducts (*R* or *S*) **3a-n** catalyzed by DpkA$_{Psyrin}$ and KPR$_{Ecoli}$.

Biocatalytic reduction catalyzed by KPR$_{Ecoli}$ or DpkA$_{Psyrin}$: Reactions were carried out at analytic level as follows: The reactions (500 μL total volume) were conducted in Eppendorf tubes (1.5 mL) and placed in a vortex mixer (100 rpm) at 25 ºC. The reduction mixture solutions (250 μL) were prepared by adding; KPR$_{Ecoli}$ (125 μL of a

stock solution of 52 U mL^{-1} ,4 mg mL^{-1} in 20 mM TEA buffer pH 7.0, 100 mM NaCl, and 50% (v/v) of glycerol, 13 U mL^{-1} final concentration in the reaction) or DpkA$_{Psyrin}$ (125 μL of a stock solution of 2.6 10^{-2} U mL^{-1}, 4 mg mL^{-1} in 20 mM TEA buffer pH 7.0, 100 mM NaCl, and 50% (v/v) of glycerol, 6.5 10^{-3} U mL^{-1} final concentration in the reaction), EDTA (5 μL of a 0.5M stock solution in 20 mM TEA buffer pH 8.0, 5 mM final concentration in the reaction), GDH (83 μL of a stock solution 20.8 U mL^{-1}, 5.2 mg mL^{-1} in 10 mM HEPES buffer pH 6.5, 50 mM NaCl, and 50% (v/v) of glycerol, 3.5 U mL^{-1} final concentration in the reaction), glucose (18 mg, 4 eq, 0.2 M final concentration in reaction) and NADP$^+$ (1.9 mg, 5 mM in reaction). The reactions were started by the addition of a sample of the corresponding aldol reaction (250 μL), containing adducts **3a-n** (≈ 100 mM as the basis of calculation) (Scheme S3**)**. Samples were withdrawn immediately after the addition of aldol substrate (0 h) and after 24 h and analyzed by HPLC as described above.

One-pot two-step stereoselective synthesis of 2-hydroxy-4-butyrolactone derivatives (5).

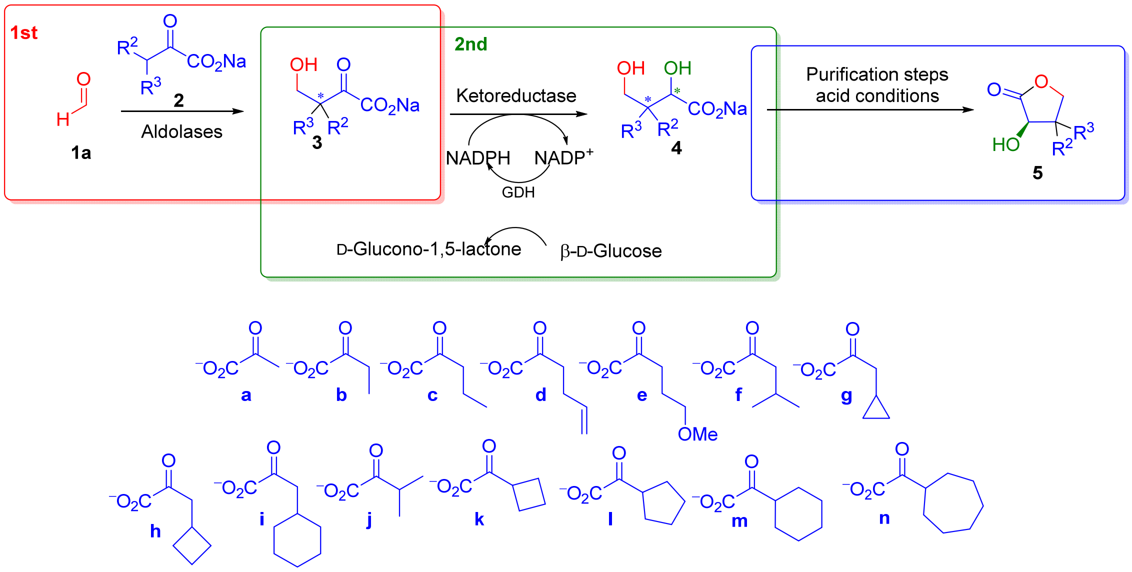

Scheme S4. One-pot two-step stereoselective synthesis of 2-hydroxy-4-butyrolactones derivatives (**5**) combining aldolases (YfaU$_{Ecoli}$ and KPHMT$_{Ecoli}$) and ketoreductases (DpkA$_{Psyrin}$ and KPR$_{Ecoli}$).

The reactions combining YfaU$_{Ecoli}$ and KPR$_{Ecoli}$ were carried out as follows:

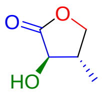

2*R*,3*S*-**5b**

Synthesis of (2*R*,3*S*)-3-methyl-2-hydroxy-4-butyrolactone (2*R*,3*S*-**5b**).Typical procedure (Scheme S4): **Aldol addition (1st)**: The reaction (1 mmol scale, 10 mL total volume) was conducted in a round-bottom flask (100 mL) at 25 °C and magnetically stirred with a bar at 250 rpm. To a solution of YfaU$_{Ecoli}$ wt (60 mg of lyophilized powder at 0.5 mg protein mg^{-1} lyophilized powder, 30 mg of protein, 3 mg protein mL^{-1} final concentration in the reaction) in plain water (4 mL), 2-oxobutyric acid (**2b**) (1 mL of a 1 M stock solution in water pH 7.0, 1 mmol, 1eq, 0.1 M in the reaction) and $NiCl_2$ (100 µL of a 0.1 M stock solution in water, 10 µmol, 1 mM in the reaction) were added. The reaction was started by adding formaldehyde (**1a**, 1 mL of a 1 M stock solution in water, 1 mmol, 1 eq, 0.1 M in the reaction). The reaction was monitored by HPLC as described in the analytical scale reactions.

Aldol reduction (2nd). After 24h, the reduction reaction (20 mL final volume) was carried out adding EDTA (200 µL, of 0.5 M stock solution in 50 mM TEA buffer pH 8.0, 5 mM in the reaction), glucose (721 mg, 4.0 mmol, 4 eq, 0.2 M final concentration in the reaction,), GDH (3.30 mL of a stock solution 20.8 U mL^{-1}, 5.2 mg mL^{-1} in 10 mM HEPES buffer pH 6.5, 50 mM NaCl, and 50% (v/v) of glycerol, 3.4 U mL^{-1} final concentration in the reaction), and KPR$_{Ecoli}$ (1.8 mL of a stock solution 52 U mL^{-1} ,4 mg mL^{-1} in 20 mM TEA buffer pH 7.0, 100 mM NaCl, and 50% (v/v) of glycerol, 4.7 U mL^{-1} final concentration in the reaction). The reaction was started by adding a solution of NADP^{+} (4.7 mL of stock solution 21 mM in 350 mM sodium phosphate buffer pH 8.0, 5 mM final concentration in the reaction). The mixture reaction was stirred at room temperature and the reaction was monitored by HPLC as described in the analytical scale reactions. After no aldol adduct was detected by HPLC (24 h), methanol (200 mL) was added under stirring. The mixture was filtered through Celite® and the filter cake washed with methanol (3 x 50 mL). The organic solvent was removed and the pH of the remaining aqueous solution was adjusted to 9.0 with 1 M NaOH. Then, water was added up to a final volume of 40 mL. The product purification was started with anion exchange chromatography (DOWEX 1X8 ion exchange resin (50-100 mesh) in HCO_2^{-} form, column: 44 cm, Ø = 1,6 cm). The sample was loaded onto the column and the resin was washed with plain water (90 mL). The bound fractions with the compound were eluted with a solution of 1 M HCO_2H (fraction volume 30 mL). Fractions containing the product were pooled freeze-dried, dissolved in methanol (30 mL), absorbed in silica and purified by column chromatography on silica with a step gradient

of hexane:EtOAc: 100:0, 200 mL, 75:25, 200 mL, 50:50, 200 mL and 25:75, 500 mL. Pure fractions were pooled and the solvent removed under vacuum affording the lactone 2*R*,3*S*-**5b** as a yellow oil (43 mg, 45%). During the freeze drying under acid conditions the lactonization of the compound **4b** took place. ^{1}H NMR (400 MHz, D_2O) δ 4.50 (dd, *J* = 9.0, 7.9 Hz, 1H), 4.23 (d, *J* = 11.1 Hz, 1H), 3.94 (dd, *J* = 10.8, 9.0 Hz, 1H), 2.61 – 2.46 (m, 1H), 1.20 (d, *J* = 6.6 Hz, 3H). ^{13}C NMR (101 MHz, D_2O) δ 180.1, 73.3, 71.5, 38.5, 12.8. Selected NMR signals for the minor diastereomer: ^{1}H NMR (400 MHz, D_2O) δ 4.42 (t, *J* = 8.5 Hz, 1H), 4.21 – 4.11 (m, 1H), 3.86 (dd, *J* = 10.8, 9.0 Hz, 1H), 2.51 – 2.35 (m, 1H), 1.12 (dd, *J* = 6.8, 1.6 Hz, 4H). ^{13}C NMR (101 MHz, D_2O) δ 72.8, 69.9, 34.7, 10.9. $[\alpha]_{20}^{D}$ = + 58.7 (*c* = 1, in MeOH). ESI-TOF m/z: Calcd for [M+Na$^+$] $C_5H_8O_3Na^+$:139.0366, found [M+Na$^+$]: 139.0364.

(*R*)-2-hydroxy-4-butyrolactone (*R*-**5a**).

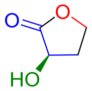

R-**5a**

The title compound was prepared as described for 2*R*,3*S*-**5b**. Starting from **2a** ([**2a**] = [**1a**] = 1 M in the reaction, $V_{\text{aldol reaction}}$ = 1 mL. The aldol reaction was diluted with plain water (9 mL) before starting the reduction reaction $V_{\text{reduction reaction}}$ = 20 mL), *R*-**5a** was obtained as a white solid (15 mg, 30%). In this case the product was eluted with a step gradient of Hexane:EtOAc: 100:0, 200 mL, 90:10, 200 mL, 80:20, 200 mL and 70:30, 500 mL on column chromatography on silica. ^{1}H NMR (400 MHz, $CDCl_3$) δ 4.51 (dd, *J* = 11.5, 9.6 Hz, 1H), 4.44 (td, *J* = 2x9.0, 1.9 Hz, 1H), 4.23 (ddd, *J* = 10.5, 9.3, 6.0 Hz, 1H), 2.67 – 2.56 (m, 1H), 2.29 (dtd, *J* = 12.7, 2x10.4, 8.8 Hz, 1H). ^{13}C NMR (101 MHz, $CDCl_3$) δ 178.0, 67.6, 65.3, 31.0. The NMR data matched that reported in the literature.[11] $[\alpha]_{20}^{D}$ = + 34.1 (*c* = 1, in MeOH), in our hands, commercial (*R*)-3-Hydroxydihydrofuran-2(3*H*)-one (Sigma Aldrich 444286). ee: ≥99% (see below), $[\alpha]_{20}^{D}$ = + 34.8 (*c* = 1, in MeOH). ESI-TOF m/z: Calcd for [M+Na$^+$] $C_4H_6NaO_3^+$:125.0209, found [M+Na$^+$]: 125.0215.

(2*R*,3*S*)-3-ethyl-2-hydroxy-4-butyrolactone (2*R*,3*S*-**5c**).

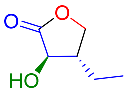

2*R*,3*S*-**5c**

The title compound was prepared using YfaU_{Ecoli} W23V variant with the procedure described for 2*R*,3*S*-**5b**. Starting from **2c** ([**2c**] = [**1a**] = 100 mM), the major lactone 2*R*,3*S*-**5c** was obtained as a yellow oil (57 mg, 44%). ^{1}H NMR (400 MHz, D_2O) δ 4.55 (dd, *J* = 9.0, 8.1 Hz, 1H), 4.32 (d, *J* = 10.9 Hz, 1H), 4.00 (dd, *J* = 10.7, 9.1 Hz, 1H), 2.44 (ttd, *J* = 2x10.8, 8.1, 8.0, 6.0 Hz, 1H),

1.75 (ddd, J = 13.6, 7.6, 6.0 Hz, 1H), 1.56 (dt, J = 13.7, 2x7.6 Hz, 1H), 0.97 (t, J = 2x7.6 Hz, 3H). ^{13}C NMR (101 MHz, D_2O) δ 180.5, 72.1, 70.6, 44.7, 22.6, 10.5. Minor lactone key selected signals: ^{1}H NMR (400 MHz, D_2O) δ 4.64 (d, J = 7.5 Hz, 1H), 4.35 (m, 1H), 4.20 (d, J = 2.4 Hz, 0H), 2.53 (dddt, J = 12.8, 7.4, 5.2, 2.4 Hz, 1H), 1.22 (m, 1H). ^{13}C NMR (101 MHz, D_2O) δ 69.3, 41.2, 18.2, 10.3. $[\alpha]_{20}^{D}$ = + 44.5 (c = 1, in MeOH). ESI-TOF m/z: Calcd for [2M+Na$^+$] $C_{12}H_{20}NaO_6^+$:283.1152, found [2M+Na$^+$]: 283.1165.

(2*R*,3*S*)-3-Allyl-2-hydroxy-4-butyrolactone (2*R*,3*S*-**5d**).

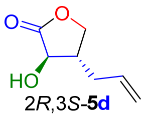
2*R*,3*S*-**5d**

The title compound was prepared using YfaU$_{Ecoli}$ W23V variant with the procedure described for 2*R*,3*S*-**5b**. Starting from **2d** ([**2d**] = [**1a**] = 100 mM), 2*R*,3*S*-**5d** was obtained as a yellow oil (41 mg, 29%). ^{1}H NMR (400 MHz, $CDCl_3$) δ 5.96 – 5.62 (m, 1H), 5.20 – 5.08 (m, 2H), 4.42 (dd, J = 9.3, 7.7 Hz, 1H), 4.14 (d, J = 10.0 Hz, 1H), 3.87 (dd, J = 10.2, 9.3 Hz, 1H), 2.65 – 2.50 (m, 2H), 2.26 (tdd, J = 9.4, 9.4, 7.3, 4.1 Hz, 1H). ^{13}C NMR (101 MHz, $CDCl_3$) δ 177.2, 133.5, 117.8, 71.8, 69.1, 43.0, 34.3. Selected NMR signals for the minor diastereomer: ^{1}H NMR (400 MHz, $CDCl_3$) δ 4.45 (dd, J = 9.3, 7.7 Hz, 1H), 4.16 (d, J = 10.0 Hz, 1H), 3.90 (dd, J = 10.2, 9.2 Hz, 1H), 2.36 – 2.22 (m, 1H). ^{13}C NMR (101 MHz, $CDCl_3$) δ 133.7, 118.01, 72.0, 43.2, 34.5. The NMR data matched that reported in the literature.[12] $[\alpha]_{20}^{D}$ = + 3.8 (c = 1, in MeOH). ESI-TOF m/z: Calcd for [M+Na$^+$] $C_7H_{10}NaO_3^+$:165.0522, found [M+Na$^+$]: 165.0527.

(2*R*,3*S*)-3-isopropyl-2-hydroxy-4-butyrolactone (2*R*,3*S*-**5f**).

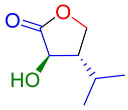
2*R*,3*S*-**5f**

The title compound was prepared using YfaU$_{Ecoli}$ W23V variant with the procedure described for 2*R*,3*S*-**5b**. Starting from **2f** ([**2f**] = [**1a**] = 100 mM), 2*R*,3*S*-**5f** was obtained as a white solid (36 mg, 25%). Mayor diasteromer (2*R*,3*S*-**5f**) ^{1}H NMR (400 MHz, $CDCl_3$) δ 4.42 (dd, J = 9.2, 8.2 Hz, 1H), 4.16 (d, J = 10.4 Hz, 1H), 3.89 (dd, J = 10.6, 9.2 Hz, 1H), 2.24 (tt, J = 2x10.5, 2x8.4 Hz, 1H), 1.79 (dp, J = 8.6, 4x6.7 Hz, 1H), 1.11 (d, J = 6.7 Hz, 3H), 0.95 (d, J = 6.7 Hz, 3H). ^{13}C NMR (101 MHz, $CDCl_3$) δ 178.2, 71.8, 68.9, 50.1, 30.9, 20.7, 20.2. Selected NMR signals for the minor diastereomer: ^{1}H NMR (400 MHz, CDC13) δ 4.36 (dd, J = 9.2, 6.8 Hz, 1H), 4.26 (dd, J = 9.3, 5.9 Hz, 1H), 2.42 – 2.32 (m, 3H), 2.13 (q, J = 6.8 Hz, 1H). ^{13}C NMR (101 MHz, $CDCl_3$) no signal detected. The NMR data matched that reported in the

literature.[13] $[\alpha]_{20}^{D}$ = + 31.4 (*c* = 1, in MeOH). ESI-TOF m/z: Calcd for [M+Na$^+$] $C_7H_{12}NaO_3^+$:167.0679, found [M+Na$^+$]: 167.0686.

(2*R*,2S)-3-Cyclopropyl--2-hydroxy-4-butyrolactone (2*R*,3*S*-**5g**).

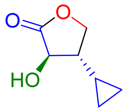

2*R*,3*S*-**5g**

The title compound was prepared using $YfaU_{Ecoli}$ W23V variant with the procedure described for 2*R*,3*S*-**5b**. Starting from **2g** ([**2g**] = [**1a**] = 100 mM), 2*R*,3*S*-**5g** was obtained as a yellow oil (66 mg, 46%). ^{1}H NMR (400 MHz, $CDCl_3$) δ 4.42 (dd, *J* = 9.2, 8.1 Hz, 1H), 4.22 (dd, *J* = 10.4, 2.1 Hz, 1H), 3.96 (dd, *J* = 10.5, 9.2 Hz, 1H), 1.95 (tt, *J* = 2x10.4, 2x8.2 Hz, 1H), 0.85 – 0.73 (m, 1H), 0.66 – 0.50 (m, 2H), 0.50 – 0.38 (m, 1H), 0.21 (dtd, *J* = 9.5, 5.6, 5.4, 4.5 Hz, 1H). ^{13}C NMR (101 MHz, $CDCl_3$) δ 177.6, 72.4, 69.2, 48.2, 10.5, 2.3 (d, *J* = 9.5 Hz). Selected NMR signals for the minor diastereomer: ^{1}H NMR (400 MHz, $CDCl_3$) δ 4.32 (dd, *J* = 9.3, 5.6 Hz, 1H), 4.12 (d, *J* = 8.7 Hz, 1H), 3.85 – 3.79 (m, 1H), 2.62 – 2.44 (m, 1H). ^{13}C NMR (101 MHz, $CDCl_3$) No signals detected. $[\alpha]_{20}^{D}$ = + 30.0 (*c* = 1.3 in MeOH). ESI-TOF m/z: Calcd for [2M+Na$^+$] $C_{14}H_{20}O_6Na^+$:307.1152, found [2M+Na$^+$]: 307.1166.

(2*R*,3*S*)-3-Cyclobutyl-2-hydroxy-4-butyrolactone (2*R*,3*S*-**5h**).

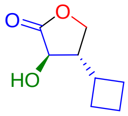

2*R*,3*S*-**5h**

The title compound was prepared using $YfaU_{Ecoli}$ W23V variant with the procedure described 2*R*,3*S*-**5b**. Starting from **2h** ([**2h**] = [**1a**] = 100 mM), 2*R*,3*S*-**5h** was obtained as a yellow oil (73 mg, 46%). ^{1}H NMR (400 MHz, $CDCl_3$) δ 4.40 (dd, *J* = 9.1, 7.7 Hz, 1H), 4.38 (dd, *J* = 10.0, 9.1 Hz, 1H), 4.13 (dd, *J* = 9.7, 2.3 Hz, 1H), 2.56 (ddd *J* = 10.0, 8.1, 2.0 Hz, 1H), 2.51 (dd, *J* = 8.1, 2.3 Hz, 1H), 2.14 – 1.97 (m, 2H), 2.09 – 1.83 (m, 2H), 1.83 – 1.79 (m, 2H). ^{13}C NMR (101 MHz, $CDCl_3$) δ 177.9, 71.4, 68.1, 48.1, 36.0, 25.6 (d, *J* = 4.6 Hz), 18.9. No other diastereomer was detected within the limits of detection by high-field ^{1}H and ^{13}C NMR (≤ 3%). $[\alpha]_{20}^{D}$ = + 47.4 (*c* = 1.2, in MeOH). ESI-TOF m/z: Calcd for [2M+Na$^+$] $C_{16}H_{24}O_6Na^+$:335.1461, found [2M+Na$^+$]: 335.1451.

The reactions combining $YfaU_{Ecoli}$ and $DpkA_{Psyrin}$ were carried out as follows:

(2*S*,3*S*)-4-Methyl-2-hydroxy-4-butyrolactone (2*S*,3*S*-**5b**).

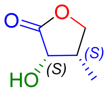

2*S*,3*S*-**5b**

Synthesis of aldol intermediate 2*S*-**3b**, was performed as described for 2*R*,3*S*-**5b**, starting from **2b** ([**2b**] = [**1a**] = 100 mM). After 24 h, the reduction

reaction (20 mL final volume) was carried out as follows: to the aldol reaction mixture were added EDTA (200 μL, of 0.5 M stock solution in 50 mM TEA buffer pH 8.0, 5 mM in the reaction), glucose (721 mg, 4 mmol, 4 eq, 0.2 M final concentration in the reaction), GDH (3.30 mL of a stock solution 20.8 U mL^{-1}, 5.2 mg mL^{-1} in 10 mM HEPES buffer pH 6.5, 50 mM NaCl, and 50% (v/v) of glycerol, 3.4 U mL^{-1} final concentration in the reaction) and $DpkA_{Psyrin}$ (5.0 mL of a stock solution 2.6 10^{-2} U mL^{-1}, 4 mg mL^{-1} in 20 mM TEA buffer pH 7.0, 100 mM NaCl, and 50% (v/v) of glycerol, 6.5 10^{-3} U mL^{-1} final concentration in the reaction). The reaction was started by adding a solution of $NADP^{+}$ (1.5 mL of stock solution 67 mM in 1 M sodium phosphate buffer pH 8.0, 5 mM final concentration in the reaction). The purification was performed as described for 2*R*,3*S*-**5b** and lactone 2*S*,3*S*-**5b** was obtained as a yellow oil (40 mg, 35%).^{1}H NMR (400 MHz, D_2O) δ 4.74 (d, *J* = 7.2 Hz, 1H), 4.47 (dd, *J* = 9.3, 5.3 Hz, 1H), 4.16 (dd, *J* = 9.3, 1.7 Hz, 1H), 2.95 – 2.67 (m, 1H), 1.05 (dd, *J* = 7.2, 1.7 Hz, 3H). ^{13}C NMR (101 MHz, D_2O) δ 179.8, 72.8, 69.9, 34.7, 10.9. Minor diastereoisomer key selected signals: ^{1}H NMR (400 MHz, D_2O) δ 3.86 (dd, *J* = 10.7, 9.0 Hz, 1H), 2.50 – 2.39 (m, 1H), 1.12 (d, *J* = 6.7 Hz, 3H). ^{13}C NMR (101 MHz, D_2O) δ 71.5, 38.5, 12.8. $[\alpha]_{20}^{D}$ = + 30.9 (*c* = 1, in MeOH). ESI-TOF m/z: Calcd for [M+Na^{+}] $C_5H_8O_3Na^{+}$:139.0366, found [M+Na^{+}]: 139.0359.

(*S*)-2-Hydroxy-4-butyrolactone (*S*-**5a**).

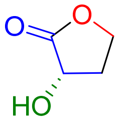

S-**5a**

The title compound was prepared as described for 2*S*,3*S*-**5b**. Starting from **2a** ([**2a**] = [**1a**] = 1 M in the reaction, $V_{aldol\ reaction}$ = 1 mL. The aldol reaction was diluted with plain water (9 mL) before starting the reduction reaction, $V_{reduction\ reaction}$ = 20 mL). The purification was performed as described for *R*-**5a,** and the title compound ***S*-5a** was obtained as a yellow oil (20 mg, 20%). NMR spectra were indistinguishable from that of ***R*-5a**. $[\alpha]_{20}^{D}$ = – 23.3 (*c* = 1, in MeOH). In our hands, commercial (*R*)-3-hydroxydihydrofuran-2(3*H*)-one (Sigma Aldrich 444286). ee: ≥99% (see below), $[\alpha]_{20}^{D}$ = + 35 (*c* = 1, in MeOH). ESI-TOF m/z: Calcd for [M+Na^{+}] $C_4H_6O_3Na^{+}$:125.0210, found [M+Na^{+}]: 125.0205.

(2*S*,3*S*)-3-Ethyl-2-hydroxy-4-butyrolactone (2*S*,3*S*-**5c**).

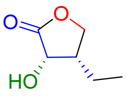

2*S*,3*S*-**5c**

Synthesis of aldol adduct 2*S*-**3c** was prepared using $YfaU_{Ecoli}$ W23V variant with the procedure described 2*S*,3*S*-**5b** and starting from **2c** ([**2c**] = [**1a**] =

100 mM). Lactone 2*S*,3*S*-**5c** was obtained as a yellow oil (22 mg, 17%). ^{1}H NMR (400 MHz, D_2O) δ 4.74 (d, *J* = 7.5 Hz, 1H), 4.44 (dd, *J* = 9.5, 5.6 Hz, 1H), 4.31 (dd, *J* = 9.5, 2.4 Hz, 1H), 2.74 – 2.46 (m, 1H), 1.64 (dtd, *J* = 14.9, 7.5, 7.4, 5.1 Hz, 1H), 1.31 (ddt, *J* = 16.4, 14.4, 7.3, 7.3 Hz, 1H), 0.96 (t, *J* = 2x7.5 Hz, 3H). ^{13}C NMR (101 MHz, D_2O) δ 180.1, 70.6, 69.3, 41.2, 18.2, 10.3. Minor lactone key selected signals: ^{1}H NMR (400 MHz, D_2O) δ 4.46 (t, *J* = 8.6 Hz, 1H), 3.92 (m, 1H), 2.36 (m, 1H), 1.73 – 1.61 (m, 1H). ^{13}C NMR (101 MHz, D_2O) δ 180.5, 72.1, 44.7, 22.6, 10.5. $[\alpha]_{20}^{D}$ = + 0.8 (*c* = 1, in MeOH). ESI-TOF m/z: Calcd for $[2M+Na^+]$ $C_{12}H_{20}O_6Na^+$:283.1152, found $[2M+Na^+]$: 283.1149.

The reactions combining KPHMT$_{Ecoli}$, and KPR$_{E.coli}$ were carried out as follows:

(2*R*,3*R*)-3-methyl-2-hydroxy-4-butyrolactone (2*R*,3*R*-**5b**).

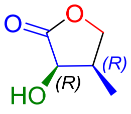

2*R*,3*R*-**5b**

Aldol addition (1st): The reaction (1 mmol scale, 10 mL total volume) was conducted in a round-bottom flask (100 mL) at 25 °C and magnetically stirred with a bar at 250 rpm. To a solution of KPHMT$_{Ecoli}$ wt (1.1 mL of 9.3 mg mL^{-1} stock solution 20 mM TEA buffer pH 7.0, 100 mM NaCl, and 50% (v/v) of glycerol, 1 mg protein mL^{-1} in the reaction) in plain (6.8 mL) water, $CoCl_2$ (100 µL of a 0.1 M stock solution, 1 mM in the reaction), and **2b** (1 mL of a 1 M stock solution in water pH 7.0, 1 mmol, 1 eq, 0.1 M in the reaction) were added. The reaction was started by adding formaldehyde (**1a**, 1 mL of a 1 M in water solution, 1 eq, 0.1 M in the reaction). The reaction was monitored by HPLC as described in the analytical scale reactions.

Aldol reduction (2nd). The reduction of aldol adduct with KPR$_{Ecoli}$ and final lactone purification was performed as describe for 2*R*,3*S*-**5b**. Final product 2*R*,3*R*-**5b** was obtained as a yellow oil (43 mg, 37%). ^{1}H NMR (400 MHz, D_2O) δ 4.51 (t, *J* = 2x8.4 Hz, 1H), 4.24 (d, *J* = 11.1 Hz, 1H), 3.95 (dd, *J* = 10.8, 9.0 Hz, 1H), 2.61 – 2.34 (m, 1H), 1.21 (d, *J* = 6.5 Hz, 3H). ^{13}C NMR (101 MHz, D_2O) δ 180.1, 73.3, 71.5, 38.5, 12.8. Minor diastereoisomer key selected signals: ^{1}H NMR (400 MHz, D_2O) δ 4.15 (d, *J* = 11.2 Hz, 1H), 3.91 – 3.82 (m, 1H), 2.44 (tdd, *J* = 12.4, 9.2, 6.3 Hz, 1H), 1.12 (d, *J* = 6.5 Hz, 1H). ^{13}C NMR (101 MHz, D_2O) δ 73.3, 71.5, 38.5, 12.8. $[\alpha]_{20}^{D}$ = – 41.7 (*c* = 1, in MeOH). ESI-TOF m/z: Calcd for $[M+Na^+]$ $C_5H_8O_3Na^+$:139.0366, found $[M+Na^+]$: 139.0371.

(2*R*,3*R*)-3-Ethyl-2-hydroxy-4-butyrolactone (2*R*,3*R*-**5c**).

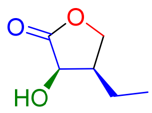

2*R*,3*R*-**5c**

The synthesis of the title compound was performed as described for 2*R*,3R-**5b**. Starting from **2c** ([**2c**] = [**1a**] = 100 mM), 2*R*,3*R*-**5c** was obtained as a yellow oil (23 mg, 25%). ^{1}H NMR (400 MHz, D_2O) δ 4.74 (d, *J* = 7.4 Hz, 1H), 4.44 (dd, *J* = 9.5, 5.6 Hz, 1H), 4.31 (dd, *J* = 9.5, 2.4 Hz, 1H), 2.63 (ddd, *J* = 7.3, 5.3, 2.2 Hz, 1H), 1.64 (dtd, *J* = 15.0, 7.6, 7.5, 5.0 Hz, 1H), 1.31 (ddq, *J* = 14.5, 9.0, 3x7.4 Hz, 1H), 0.96 (t, *J* = 2x7.5 Hz, 3H). ^{13}C NMR (101 MHz, D_2O) δ 180.1, 70.6, 69.3, 41.1, 18.2, 10.3. Minor lactone key selected signals: ^{1}H NMR (400 MHz, D_2O) δ 4.46 (t, *J* = 8.6 Hz, 1H), 3.92 (m, 1H), 2.36 (m, 1H), 1.73 – 1.61 (m, 1H). ^{13}C NMR (101 MHz, D_2O) δ 180.5, 72.1, 44.7, 22.6, 10.5. $[\alpha]_{20}^{D}$ = – 2.0 (*c* = 1, in MeOH). Calcd for [2M+Na$^+$] $C_{12}H_{20}O_6Na^+$:283.1152, found [2M+Na$^+$]: 283.1159.

(2*R*,3*R*)-3-Allyl-2-hydroxy-4-butyrolactone (2*R*,3*R*-**5d**).

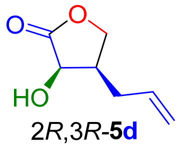

2*R*,3*R*-**5d**

The title compound was prepared using KPHMT$_{Ecoli}$ I212A variant and following the procedure described for 2*R*,3*R*-**5b**. Starting from **2d** ([**2d**] = [**1a**] = 100 mM), 2*R*,3*R*-**5d** was obtained as a yellow oil (36 mg, 25%).

^{1}H NMR (400 MHz, $CDCl_3$) 5.87 – 5.63 (m, 1H), 5.26 – 5.07 (m, 2H), 4.56 (d, *J* = 7.4 Hz, 1H), 4.25 (d, *J* = 2.3 Hz, 1H), 2.78 – 2.63 (m, 1H), 2.58 – 2.49 (m, 1H), 2.04 (dt, *J* = 14.5, 2x9.0 Hz, 1H). ^{13}C NMR (101 MHz, $CDCl_3$) δ 177.3, 134.6, 118.2, 69.4, 68.9, 39.5, 29.9. Selected NMR signals for the minor diastereomer: ^{1}H NMR (400 MHz, $CDCl_3$) δ 5.86 – 5.81 (m, 1H), 5.17 (dq, *J* = 6.1, 1.6, 1.5, 1.5 Hz, 2H), 4.42 (dd, *J* = 9.3, 7.7 Hz, 1H), 4.27 (ddd, *J* = 9.4, 5.4, 0.7 Hz, 3H), 4.14 (d, *J* = 10.0 Hz, 1H), 2.64 – 2.56 (m, 1H), 2.32 – 2.20 (m, 1H). ^{13}C NMR (101 MHz, $CDCl_3$) δ 177.1, 133.5, 117.8, 71.8, 69.1, 43.0, 34.3. $[\alpha]_{20}^{D}$ = – 20.0 (*c* = 0.6, in MeOH). ESI-TOF m/z: Calcd for [M+Na$^+$] $C_7H_{10}O_3Na^+$: 165.0522, found [M+Na$^+$]: 165.0528.

(2*R*,3*R*)-3-(3-methoxypropyl)-2-hydroxy-4-butyrolactone (2*R*,3*R*-**5e**).

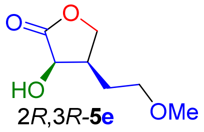

2*R*,3*R*-**5e**

The title compound was prepared using KPHMT$_{Ecoli}$ I212A variant and following the procedure described for 2*R*,3*R*-**5b**. Starting from **2e** ([**2e**] = [**1a**] = 100 mM), 2*R,3R*-**5e** was obtained as a yellow oil (64 mg, 40%).^{1}H NMR (400 MHz, DMSO) δ 4.35 (dd, *J* = 7.0, 6.0 Hz, 1H), 4.26 (dd, *J* = 8.9, 6.1 Hz, 1H), 4.07 (dd, *J* = 9.0, 4.2 Hz, 1H), 3.39 (td, *J* = 2x6.4, 4.4 Hz, 2H), 3.23 (s,

3H), 2.57 – 2.45 (m, 1H), 1.81 (ddd, J = 14.3, 7.1, 5.6 Hz, 1H), 1.39 (ddd, J = 14.0, 10.7, 6.5 Hz, 1H). ^{13}C NMR (101 MHz, DMSO) δ 176.9, 69.9, 69.5, 68.0, 57.9, 37.5, 25.2. Minor lactone key selected signals: ^{1}H NMR (400 MHz, DMSO) δ 3.83 (dd, J = 10.6, 8.9 Hz, 1H), 2.31 (m, 1H), 1.72 – 1.59 (m, 1H). ^{3}C NMR (101 MHz, DMSO) δ 71.9, 70.5, 69.3, 42.2, 30.4. $[\alpha]_{20}^{D}$ = + 2.8 (c = 1, in MeOH). ESI-TOF m/z: Calcd for [M+Na^{+}] $C_7H_{12}O_4Na^{+}$: 183.0628, found [M+Na^{+}]: 183.0635.

(2*R*,3*R*)-3-isopropyl-2-hydroxy-4-butyrolactone (2*R*,3*R*-**5f**).

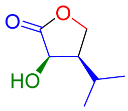
2*R*,3*R*-**5f**

The title compound was prepared using KPHMT$_{Ecoli}$ I202A variant and following the procedure described for 2*R*,3*R*-**5b**. Starting from **2f** ([**2f**] = [**1a**] = 100 mM), 2*R*,3*R*-**5f** was obtained as a yellow oil (52 mg, 36%). ^{1}H NMR (400 MHz, $CDCl_3$) δ 4.42 (dd, J = 7.1, 1.9 Hz, 1H), 4.36 (dd, J = 9.2, 6.9 Hz, 1H), 4.26 (dd, J = 9.3, 5.9 Hz, 1H), 2.35 (qd, J = 7.0, 7.0, 7.0, 5.9 Hz, 1H), 2.13 (h, J = 6.8 Hz, 1H), 1.04 (d, J = 6.8 Hz, 3H), 0.91 (d, J = 6.8 Hz, 3H).^{13}C NMR (101 MHz, $CDCl_3$) δ 177.6, 69.2, 68.3, 46.3, 24.6, 20.8, 18.6. Selected NMR signals for the minor diastereomer ^{1}H NMR (400 MHz, $CDCl_3$) δ 3.92 (dd, J = 10.7, 9.2 Hz, 1H), 2.26 (ddd, J = 10.5, 8.4, 2.1 Hz, 1H), 1.86 – 1.75 (m, 1H), 1.13 (d, J = 6.7 Hz, 1H), 0.97 (d, J = 6.7 Hz, 1H). ^{13}C NMR (101 MHz, $CDCl_3$) δ (very small signals) 71.7, 68.8, 49.9, 20.5, 20.1. The NMR data matched that reported in the literature.[13] $[\alpha]_{20}^{D}$ = + 42.4 (c = 1.1, in MeOH). ESI-TOF m/z: Calcd for [M+Na^{+}] $C_7H_{12}O_3Na^{+}$: 167.0679, found [M+Na^{+}]: 167.0674.

(2*R*,3*R*)-3-Cyclopropyl-2-hydroxy-4-butyrolactone (2*R*,3*R*-**5g**).

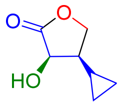
2*R*,3*R*-**5g**

The title compound was prepared as described for 2*R*,3R-**5b**. Starting from **2g** ([**2g**] = [**1**] = 100 mM), 2*R*,3*R*-**5g** was obtained as a white solid (40 mg, 28%). ^{1}H NMR (400 MHz, $CDCl_3$) δ 4.51 (d, J = 7.2 Hz, 1H), 4.32 (dd, J = 9.3, 5.5 Hz, 1H), 4.22 (dd, J = 9.3, 2.5 Hz, 1H), 2.06 – 1.93 (m, 1H), 0.95 – 0.83 (m, 1H), 0.71 (dddd, J = 9.2, 8.2, 5.7, 4.8 Hz, 1H), 0.58 – 0.51 (m, 1H), 0.32 (ddd, J = 10.5, 9.5, 5.0 Hz, 1H), 0.17 (dddd, J = 14.2, 9.5, 5.5, 4.7 Hz, 1H). ^{13}C NMR (101 MHz, $CDCl_3$) δ 177.2, 70.3, 70.2, 45.5, 7.9, 4.2, 2.0. Selected NMR signals for the minor diastereomer ^{1}H NMR (400 MHz, $CDCl_3$) δ 4.42 (dd, J = 9.3, 8.0 Hz, 1H), 3.96 (dd, J = 10.5, 9.2 Hz, 1H), 0.79 (tdd, J = 8.3, 4.8, 3.3 Hz, 1H), 0.44 (dq, J = 9.3, 4.9 Hz, 1H). ^{13}C NMR (101 MHz, $CDCl_3$) δ 72.4, 69.2, 48.2, 10.5, 2.3, 2.2. $[\alpha]_{20}^{D}$ = – 2.9 (c = 1.3, in

MeOH). ESI-TOF m/z: Calcd for [2M+Na$^+$] $C_{14}H_{20}O_6Na^+$: 307.1152, found [2M+Na$^+$]: 307.1166.

(2*R*,3*R*)-3-Cyclobutyl-2-hydroxy-4-butyrolactone (2*R*,3*R*-**5h**).

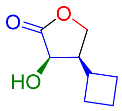

2*R*,3*R*-**5h**

The title compound was prepared using KPHMT$_{Ecoli}$ I202A variant and following the procedure described for 2*R*,3*R*-**5b**. Starting from **2h** ([**2h**] = [**1a**] = 100 mM), 2*R*,3*R*-**5h** was obtained as a white solid (30 mg, 30%). ^{1}H NMR (400 MHz, $CDCl_3$) δ 4.54 (d, *J* = 7.1 Hz, 1H), 4.26 (dd, *J* = 9.4, 5.3 Hz, 1H), 4.14 (d, *J* = 1.9 Hz, 1H), 2.65-2.59 (m, 1H), 2.58-2.51 (m, 1H), 2.18-2.13 (m, 1H), 2.09-2.02 (m, 1H), 1.96-1.98 (m, 1H), 1.87-1.84 (m, 2H), 1.73-164 (m, 1H). ^{13}C NMR (101 MHz, $CDCl_3$) δ 177.6, 70.1, 68.2, 45.6, 33.0, 28.1, 26.2, 19.0. Selected NMR signals for the minor diastereomer: ^{1}H NMR (400 MHz, $CDCl_3$) δ 4.39 (dd, *J* = 9.1, 7.8 Hz, 1H), 4.13 – 4.07 (m, 1H), 3.84 (t, *J* = 2x9.6 Hz, 1H), 2.58-2.51 (m, 1H), 2.50-2.46 (m, 1H), 1.97-1.90 (m, 3H), 1.88-1.81 (m, 3H). ^{13}C NMR (101 MHz, $CDCl_3$) 177.8, 71.5, 68.2, 48.0, 36.0, 25.6, 25.6, 18.9. $[\alpha]_{20}^{D}$ = + 2 (*c* = 0.8 in MeOH). ESI-TOF m/z: Calcd for [M+Na$^+$] $C_8H_{10}O_3Na^+$: 179.0678, found [M+Na$^+$]: 179.0671.

(*R*)-3,3-dimethyl-2-hydroxy-4-butyrolactone (*R*-**5j**).

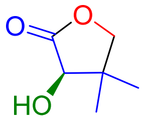

R-**5j**

The title compound was prepared as described for 2*R*,3R-**5b**. Starting from **2j** ([**2j**] = [**1a**] = 100 mM), *R*-**5j** was obtained as a white solid (66 mg, 51%). ^{1}H NMR (400 MHz, $CDCl_3$) δ 4.12 (s, 1H), 4.03 (d, *J* = 8.9 Hz, 1H), 3.94 (dd, *J* = 8.9, 0.8 Hz, 1H), 1.23 (s, 3H), 1.08 (s, 3H). ^{13}C NMR (101 MHz, $CDCl_3$) δ 177.6, 76.5, 75.9, 41.0, 23.1, 18.9. The NMR data matched that reported in the literatura[14]. ee: ≥99% (see below), $[\alpha]_{20}^{D}$ = – 22.5 (*c* = 1 in MeOH), In our hands, commercial D-(−)-pantolactone (Sigma Aldrich 237817) $[\alpha]_{20}^{D}$ = – 28.0 (*c* = 1 in MeOH). ESI-TOF m/z: Calcd for [M+Na$^+$] $C_6H_{10}O_3Na^+$: 153.0522, found [M+Na$^+$]: 153.0527.

(*R*)-8-Hydroxy-6-oxaspiro[3.4]octan-7-one (*R*-**5k**).

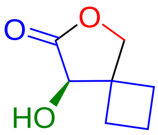

R-**5k**

The title compound was prepared as described for 2*R*,3R-**5b**. Starting from **2k** ([**2k**] = [**1a**] = 100 mM), *R*-**5k** was obtained as a white solid (58 mg, 40%).^{1}H NMR (400 MHz, $CDCl_3$) δ 4.41 (d, *J* = 9.1 Hz, 1H), 4.18

(s, 1H), 4.10 (d, *J* = 9.1 Hz, 1H), 2.54 (dd, *J* = 14.6, 5.5 Hz, 1H), 2.31 (dtd, *J* = 9.9, 8.5, 8.1, 2.0 Hz, 1H), 2.01 (dp, *J* = 8.5, 2x2.4, 2x2.2 Hz, 1H), 1.98 – 1.88 (m, 2H), 1.83 – 1.73 (m, 1H). ^{13}C NMR (101 MHz, $CDCl_3$) δ 177.0, 75.4, 73.1, 46.7, 26.3, 25.1, 15.8. The NMR data matched that reported in the literature.[14] ee: ≥99% (see below), $[\alpha]_{20}^{D}$ = + 20.1 (*c* = 1 in MeOH). ESI-TOF m/z: Calcd for [M+Na^+] $C_7H_{10}O_3Na^+$: 165.0522, found [M+Na^+]: 165.0533.

(*R*)-4-Hydroxy-2-oxaspiro[4.4]nonan-3-one (*R*-**5l**).

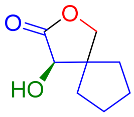
R-**5l**

The title compound was prepared using KPHMT$_{Ecoli}$ I202A variant and following the procedure described for 2*R*,3*R*-**5b**. Starting from **2l** ([**2l**] = [**1a**] = 100 mM), *R*-**5l** was obtained as a white solid (63 mg, 40%). ^{1}H NMR (400 MHz, $CDCl_3$) δ 4.28 (s, 1H), 4.13 (d, *J* = 8.9 Hz, 1H), 4.02 (dd, *J* = 8.9, 1.0 Hz, 1H), 2.06 – 1.95 (m, 1H), 1.94 – 1.84 (m, 1H), 1.83 – 1.75 (m, 1H), 1.75 – 1.68 (m, 1H), 1.68 – 1.60 (m, 3H), 1.48 – 1.37 (m, 1H). ^{13}C NMR (101 MHz, $CDCl_3$) δ 177.5, 76.1, 73.9, 51.8, 33.8, 29.3, 25.2, 25.1. The NMR data matched that reported in the literature.[15] ee: ≥99% (see below). The optical rotation values [α] was determined for *R*-**10l** (see below). ESI-TOF m/z: Calcd for [2M+Na^+] $C_{16}H_{24}O_6Na^+$: 335.1465, found [2M+Na^+]: 335.1461.

(*R*)-4-Hydroxy-2-oxaspiro[4.5]decan-3-one (*R*-**5m**).

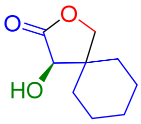
R-**5m**

The title compound was prepared using KPHMT$_{Ecoli}$ I212A variant and following the procedure described for 2*R*,3*R*-**5b**. Starting from **2m** ([**2m**] = [**1a**] = 100 mM), *R*-**5m** was obtained as a yellow solid (26 mg, 15%).^{1}H NMR (400 MHz, $CDCl_3$) δ 4.35 (d, *J* = 9.1 Hz, 1H), 4.07 (s, 1H), 3.88 (dd, *J* = 9.2, 1.5 Hz, 1H), 1.83 – 1.68 (m, 2H), 1.69 – 1.55 (m, 4H), 1.50 – 1.35 (m, 2H), 1.34 – 1.18 (m, 2H). ^{13}C NMR (101 MHz, $CDCl_3$) δ 177.4, 75.6, 73.4, 44.0, 33.6, 25.7, 25.2, 22.8, 21.6. The NMR data matched that reported in the literature.[15] ee: 98% (see below). The optical rotation values [α] was determined for *R*-**10m** (see below). ESI-TOF m/z: Calcd for [2M+Na^+] $C_{18}H_{28}O_6Na^+$: 363.1778, found [2M+Na^+]: 363.1769.

(*R*)-4-Hydroxy-2-oxaspiro[4.6]undecan-3-one (*R*-**5n**).

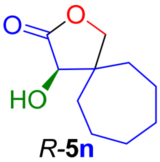
R-**5n**

The title compound was prepared using KPHMT$_{Ecoli}$ I202A variant and following the procedure described for 2*R*,3*R*-**5b**. Starting from **2n** ([**2n**] =

[**1a**] = 100 mM), *R*-**5n** was obtained as a yellow solid (20 mg, 11%). ^{1}H NMR (400 MHz, $CDCl_3$) δ 4.16 (s, 1H), 4.14 (s, 1H), 3.85 (dd, *J* = 9.0, 1.5 Hz, 1H), 1.97 – 1.76 (m, 2H), 1.77 – 1.56 (m, 5H), 1.57 – 1.36 (m, 5H). ^{13}C NMR (101 MHz, $CDCl_3$) δ 177.9, 77.8, 75.1, 47.4, 37.0, 30.2, 30.1, 29.3, 23.4, 22.9. The NMR data matched that reported in the literature.[15] ee: 98% (see below). The optical rotation values [α] was determined for *R*-**10n** (see below). ESI-TOF m/z: Calcd for [M+Na$^+$] $C_{10}H_{16}O_3Na^+$: 207.0992, found [M+Na$^+$]: 207.0981.

The reactions combining KPHMT$_{Ecoli}$, and DpkA$_{Psyrin}$ were carried out as follows:
(2*S*,3*R*)-3-Methyl-2-hydroxy-4-butyrolactone (2*S*,3*R*-**5b**).

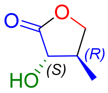

2*S*,3*R*-**5b**

The title compound was prepared as follows. The aldol reaction ([**2b**] = [**1**] = 100 mM) was performed as described for 2*R*,3*R*-**5b**, and then was submitted to reduction with DpkA$_{Psyrin}$ catalysis using the procedure described for 2*S*,3*S*-**5b**. Compound 2*S*,3*R*-**5b** was obtained as a yellow oil (61 mg, 53%). ^{1}H NMR (400 MHz, D_2O) δ 4.5 (t, *J* = 2x8.5 Hz, 1H), 4.2 (d, *J* = 11.1 Hz, 1H), 3.9 (dd, *J* = 10.8, 9.0 Hz, 1H), 2.6 – 2.4 (m, 1H), 1.2 (d, *J* = 6.5 Hz, 3H). ^{13}C NMR (101 MHz, D_2O) δ 180.1, 73.3, 71.5, 38.5, 12.8. Selected NMR signals for the minor diastereomer: ^{1}H NMR (400 MHz, D_2O) δ 4.67 – 4.64 (m, 1H), 4.10 – 4.03 (m, 1H), 2.79 – 2.68 (m, 1H), 0.97 (d, *J* = 7.1 Hz, 3H). ^{13}C NMR (101 MHz, D_2O) δ 72.8, 69.9, 34.7, 10.9. $[\alpha]_{20}^{D}$ = – 53.4 (*c* = 1 in MeOH). ESI-TOF m/z: Calcd for [M+Na$^+$] $C_5H_8O_3Na^+$:139.0366, found [M+Na$^+$]: 139.0375.

One-pot enzymatic cascade for the stereoselective synthesis of 2-hydroxy-4-butyrolactones derivatives (5a,b).

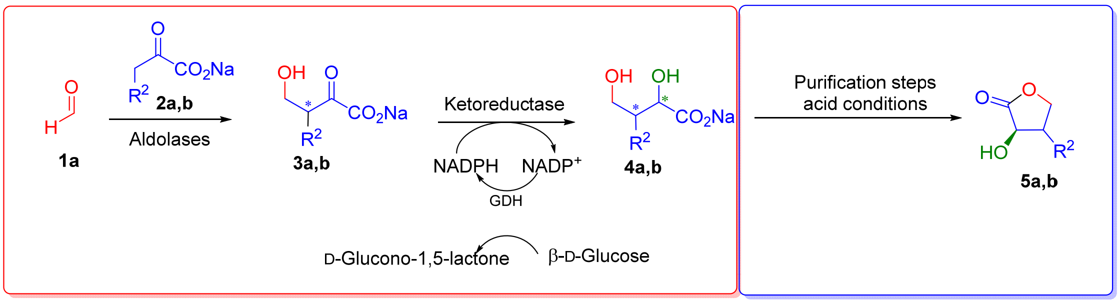

Scheme S5. One-pot enzymatic cascade for the stereoselective synthesis of 2-hydroxy-4-butyrolactones derivatives (**5a,b**) combining aldolases (YfaU$_{Ecoli}$ and KPHMT$_{Ecoli}$) and ketoreductases (DpkA$_{Psyrin}$ and KPR$_{Ecoli}$).

The reaction (1 mmol scale, 10 mL total volume) was conducted in a round-bottom flask (100 mL) at 25 °C and magnetically stirred with a bar at 250 rpm. To a solution of aldolase (YfaU$_{Ecoli}$ *wt*, 60 mg of lyophilized powder at 0.5 mg protein mg^{-1} lyophilized powder, 30 mg of protein, 3 mg protein mL^{-1} final concentration in the reaction or KPHMT$_{\mathrm{Ecoli}}$ wt, 1.1 mL of 9.3 mg mL^{-1} stock solution 20 mM TEA buffer pH 7.0, 100 mM NaCl, and 50% (v/v) of glycerol, 1 mg protein mL^{-1} in the reaction) in sodium phosphate buffer pH 8.0 (83 mM in the reaction), 2-oxoacid (**2a or 2b**) (stock solution in water pH 7.0, 1 mmol, 1eq, 0.1 M in the reaction) and MCl_2 (100 µL of a 0.1 M stock solution in water, 10 µmol, 1 mM in the reaction, M^{2+} = Ni^{2+} or $Co^{2+}$for YfaU$_{\mathrm{Ecoli}}$ wt and KPHMT$_{\mathrm{Ecoli}}$ wt respectively) were added. Then, ketoreductase (KPR$_{\mathrm{Ecoli}}$,0.9 mL of a stock solution 52 U mL^{-1} ,4 mg mL^{-1} in 20 mM TEA buffer pH 7.0, 100 mM NaCl, and 50% (v/v) of glycerol, 4.7 U mL^{-1} final concentration in the reaction or DpkA$_{\mathrm{Psyrin}}$, 2.5 mL of a stock solution 2.6 10^{-2} U mL^{-1}, 4 mg mL^{-1} in 20 mM TEA buffer pH 7.0, 100 mM NaCl, and 50% (v/v) of glycerol, 6.5 10^{-3} U mL^{-1} final concentration in the reaction), glucose (721 mg, 4.0 mmol, 4 eq, 0.4 M final concentration in the reaction), GDH (1.6 mL of a stock solution 20.8 U mL^{-1}, 5.2 mg mL^{-1} in 10 mM HEPES buffer pH 6.5, 50 mM NaCl, and 50% (v/v) of glycerol, 3.4 U mL^{-1} final concentration in the reaction) and $NADP^+$ (42 mg, 0.05 mmol, 5 mM final concentration in the reaction) were added. The reaction was started by adding formaldehyde (**1**, 1 mL of a 1 M stock solution in water, 1 mmol, 1 eq, 0.1 M in the reaction). The reaction was monitored by HPLC (see above in the description of analytical scale reactions) and the purification was performed as described for 2*R*,3*S*-**5b** and *R*-**5a**. Products: *R*-**5a**, *S*-**5a**, 2*R*,3*S*-**5b**, 2*S*,3*S*-**5b**, 2*R*,3*R*-**5b** and 2*S*,3*R*-**5b** were obtained with 30, 20, 57, 33, 37 and 57 % of isolated yield respectively. The physical and chemical properties of the products were indistinguishable from the lactones obtained in one pot two steps strategy.

Strategy for enantiomeric excess determination of products 5 (a, j-n).

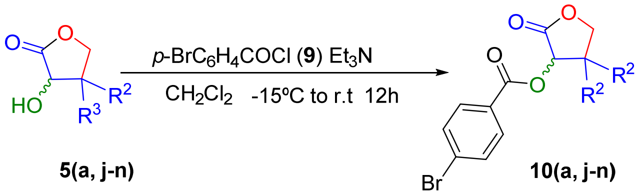

Scheme S6. Chemical modification of lactones **5** (**a**, **j-n**) for enantiomeric excess determination.

(*R*)-4,4-Dimethyl-2-oxotetrahydrofuran-3-yl 4-bromobenzoate (*R*-**10j**)

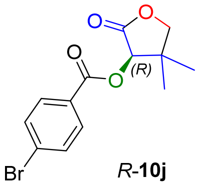

R-**10j**

Typical procedure: A dried three-necked round bottomed flask was charged with anhydrous CH_2Cl_2 (20 mL) under N_2 atmosphere. Then, *R*-**5j** (100 mg, 0.8 mmol, 1.0 eq) was added and the mixture was cooled at –15 °C. The reaction was started by addition of 4-bromobenzoyl chloride (**9**) (219 mg, 1.0 mmol, 1.3 eq) and Et_3N (161 µL, 117 mg, 1.2 mmol, 1.5 eq). The mixture was stirred 1h at –15 °C and 15 h at r.t. The reaction was diluted with CH_2Cl_2 (200 mL), transferred to a separation funnel and extracted with 1 M HCl (200 mL), 5% $NaHCO_3$ (3 x 100 mL) and brine (3 x 100 mL). The organic phase was dried over anhydrous magnesium sulfate, filtered and the solvent was removed under vacuum. The product was loaded onto a silica column chromatography and eluted with a step gradient of Hexane:EtOAc: 100:0, 200 mL, 90:10, 200 mL and 80:20, 700 mL. Pure fractions were pooled and the solvent was removed under vacuum affording the title compound (*R*-**10j**) as a white solid (172 mg, 72%). ^{1}H NMR (400 MHz, $CDCl_3$) δ 7.95 (d, J = 8.5 Hz, 1H), 7.62 (d, J = 8.5 Hz, 1H), 5.60 (s, 1H), 4.11 (d, J = 3.8 Hz, 2H), 1.28 (s, 3H), 1.22 (s, 3H). ^{13}C NMR (101 MHz, $CDCl_3$) δ 172.6, 165.2, 132.4, 132.0, 129.5, 128.1, 76.7, 76.2, 41.0, 23.6, 20.5. ee: ≥99%. $[\alpha]_{20}^{D}$ = + 16.9 (c = 1 in MeOH).

***rac*-4,4-Dimethyl-2-oxotetrahydrofuran-3-yl 4-bromobenzoate** (*rac*-**10j**).

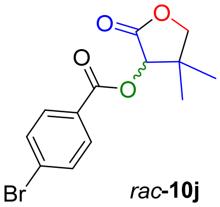

rac-**10j**

Synthesis of *rac*-**10j** was performed as described for *R*-**10j**. Starting from commercial DL-pantolactone (100 mg, TCI EUROPE P0010), affording *rac*-**10j** as a white solid (54 mg, 22%). NMR spectra were indistinguishable from that of *R*-**10j**.

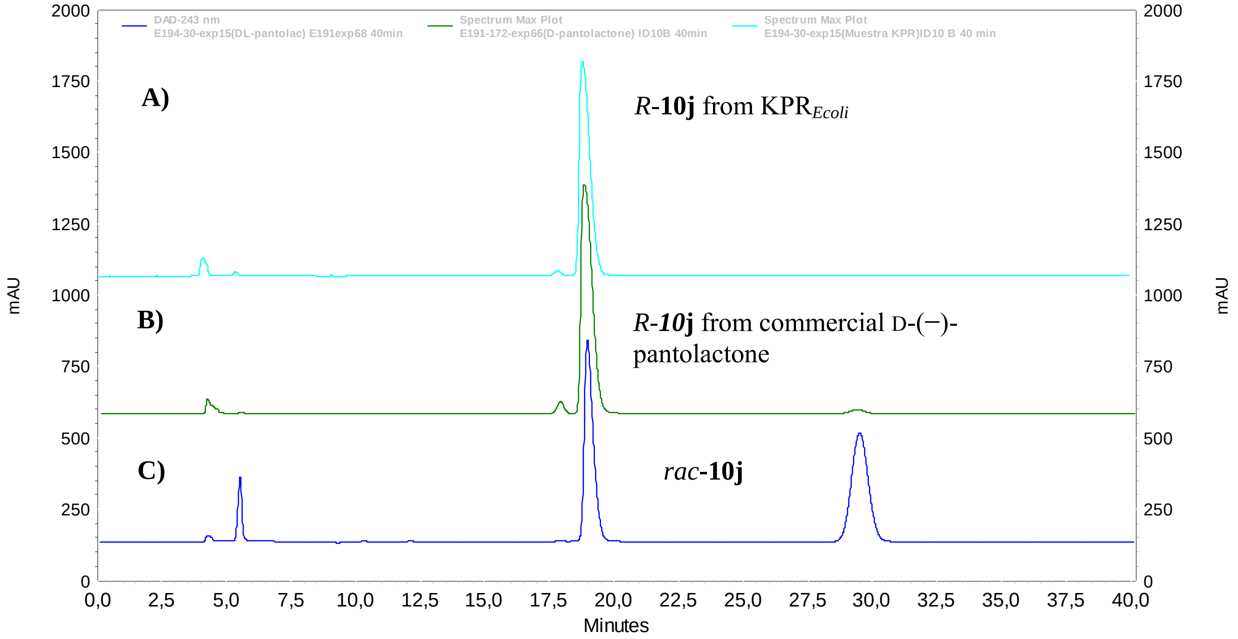

Figure S5. HPLC analysis on chiral stationary phase (CPHPLC) of *R*-**10j** from KPR$_{Ecoli}$ catalysis (**A**), *R*-**10j** from commercial D-(−)-pantolactone (Sigma-Aldrich 237817) (**B**) and *rac*-**10j** from commercial D,L-pantolactone (TCI EUROPE P0010) (**C**). The analyte *R*-**10j** was identified by comparing retention time to that of a commercial standard. Conditions: CHIRALPACK® ID, flow rate 1 mL min^{-1} at 30 °C and detection at 254 nm. Isocratic elution Hexane:^{i}PrOH 90:10 (v/v). tr (*R*) = 19.7 min, tr (*S*) = 29.6 min.

(*R*)-2-Oxotetrahydrofuran-3-yl 4-bromobenzoate (*R*-**10a**).

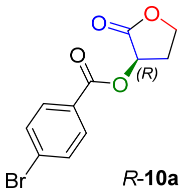

R-**10a**

The synthesis of *R*-**10a** was performed as described for *R*-**10j**. Starting from a solution of *R*-**5a** (30 mg) in anhydrous acetonitrile (5 mL), *R*-**10a** was obtained as a yellow oil (64 mg, 76%).^{1}H NMR (400 MHz, $CDCl_3$) δ 7.94 (d, *J* = 8.6 Hz, 1H), 7.61 (d, *J* = 8.6 Hz, 1H), 5.64 (dd, *J* = 9.7, 8.6 Hz, 1H), 4.55 (td, *J* = 2x9.1, 2.5 Hz, 1H), 4.37 (td, *J* = 2x9.6, 6.5 Hz, 1H), 2.90 – 2.77 (m, 1H), 2.43 (dtd, *J* = 12.9, 2x9.8, 9.0 Hz, 1H). ^{13}C NMR (101 MHz, $CDCl_3$) δ 172.9, 165.3, 132.4, 132.0, 129.5, 128.1, 68.8, 65.5, 29.6. ee: ≥99%. $[\alpha]_{20}^{D}$ = + 15.6 (*c* = 1.2 in MeOH).

(*S*)-2-Oxotetrahydrofuran-3-yl 4-bromobenzoate (*S*-**10a**).

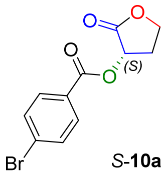

S-**10a**

The synthesis of *S*-**10a** was performed as described for *R*-**10j**. Starting from a solution *S*-**5a** (30 mg) in anhydrous acetonitrile (5 mL), *S*-**10a** was obtained as a yellow oil (20 mg, 20%). NMR spectra were

indistinguishable from that of *R*-**10a**. ee: ≥99%. $[\alpha]_{20}^{D}$ = –14.7 (*c* = 1 in MeOH).

***rac*-2-Oxotetrahydrofuran-3-yl 4-bromobenzoate** (*rac*-**10a**).

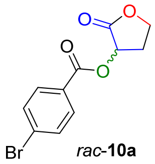

rac-**10a**

The aldol precursor **3a** was prepared following the procedure described for 2*R*,3*S*-**5b**. Starting from sodium pyruvate **2a** (440 mg, 4 mmol, 1 eq, 1 M concentration in the reaction) and **1a** (120 mg, 4 mmol, 1 eq, 1 M in the reaction). **Chemical reduction**: after 24 h of the aldol reaction, $NaBH_4$ (756 mg, 20 mmol, 5 eq) was added to the mixture and stirred at 25 °C for 1 h. Formation of *rac-4a* was estimated by measuring the aldol adduct **3a** consumed by HPLC as described above and product purification was performed as described for *R*-**5a** (*rac*-**5a**, 89 mg, 22%). The synthesis of title compound was performed as described for *R*-**10j**, starting from a solution of *rac*-**5a** (30 mg) in acetonitrile (5 mL), afforded *S*-**10a** as a white solid (39 mg, 47%). NMR spectra were indistinguishable from *R*-**10a**.

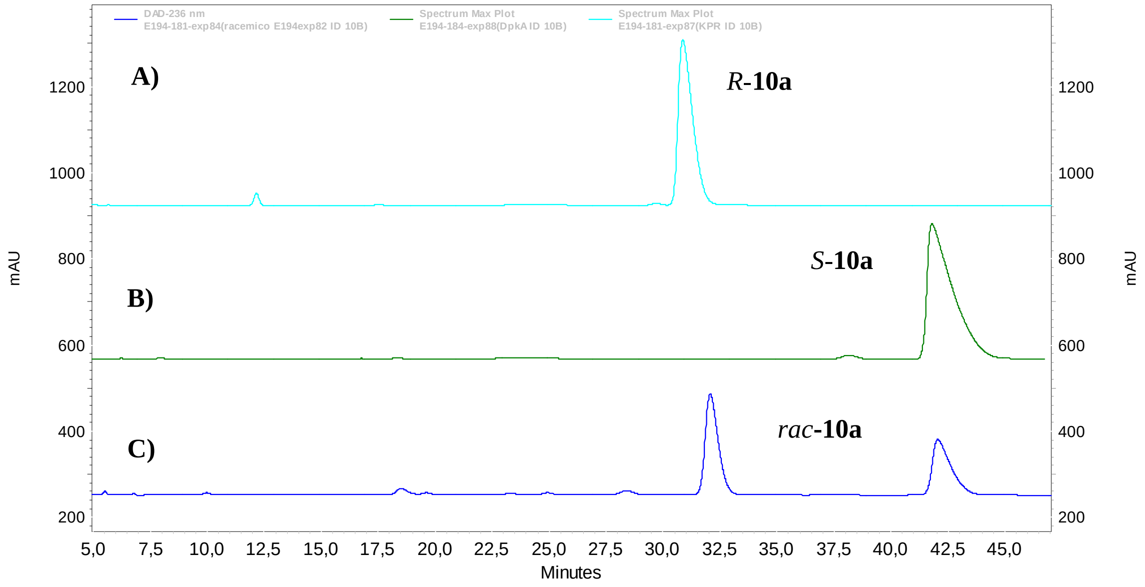

Figure S6. CPHPLC chromatogram of *R*-**10a** from KPR$_{Ecoli}$ catalysis (**A**), ***S*-10a** from DpkA$_{Psyrin}$ catalysis (**B**) *rac*-**10a** (**C**). Conditions: CHIRALPACK® ID, flow rate 1 mL min^{-1} at 30 °C and detection at 254 nm. Isocratic elution hexane:^{i}PrOH 90:10 (v/v). tr (*R*) = 32.9 min, tr (*S*) = 43.0 min.

(*R*)-7-Oxo-6-oxaspiro[3.4]octan-8-yl 4-bromobenzoate (*R*-**10k**).

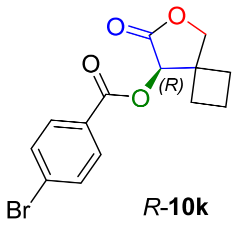

R-**10k**

Synthesis of title compound was performed as described for *R*-**10j** starting from *R*-**5k** (66 mg). *R*-**10k** was obtained as a white solid (115 mg, 76% yield). ^{1}H NMR (400 MHz, $CDCl_3$) δ 8.02 (d, *J* =

8.5 Hz, 1H), 7.66 (d, J = 8.5 Hz, 1H), 5.70 (s, 1H), 4.53 (d, J = 9.2 Hz, 1H), 4.30 (d, J = 9.3 Hz, 1H), 2.62 – 2.47 (m, 1H), 2.44 – 2.26 (m, 1H), 2.17 – 1.77 (m, 4H). ^{13}C NMR (101 MHz, $CDCl_3$) δ 171.7, 164.9, 132.0, 131.6, 129.1, 127.7, 45.9, 27.1, 26.6, 15.9. ee: ≥99%. $[\alpha]_{20}^{D}$ = + 44.7 (c = 1.5 in MeOH).

***rac*-7-Oxo-6-oxaspiro[3.4]octan-8-yl 4-bromobenzoate** (*rac*-**10k**).

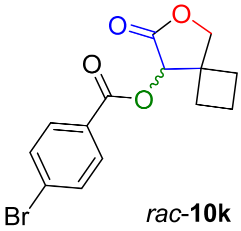

rac-**10k**

The aldol precursor **3k** was prepared following the procedure described for 2*R*,3*S*-**5b**, starting from **2k** (150 mg, 1 eq, 0.1 M concentration in the reaction) and **1a** (30 mg, 0.1 M concentration in the reaction). Chemical reduction of aldol adduct was prepared as described for *rac*-**10a**. Benzoylation of *rac*-**5k** (30 mg) was performed as describe for *R*-**10j**. The title compound was obtained as a yellow oil (32 mg, 47%). NMR spectra were indistinguishable from that of *R*-**10k**.

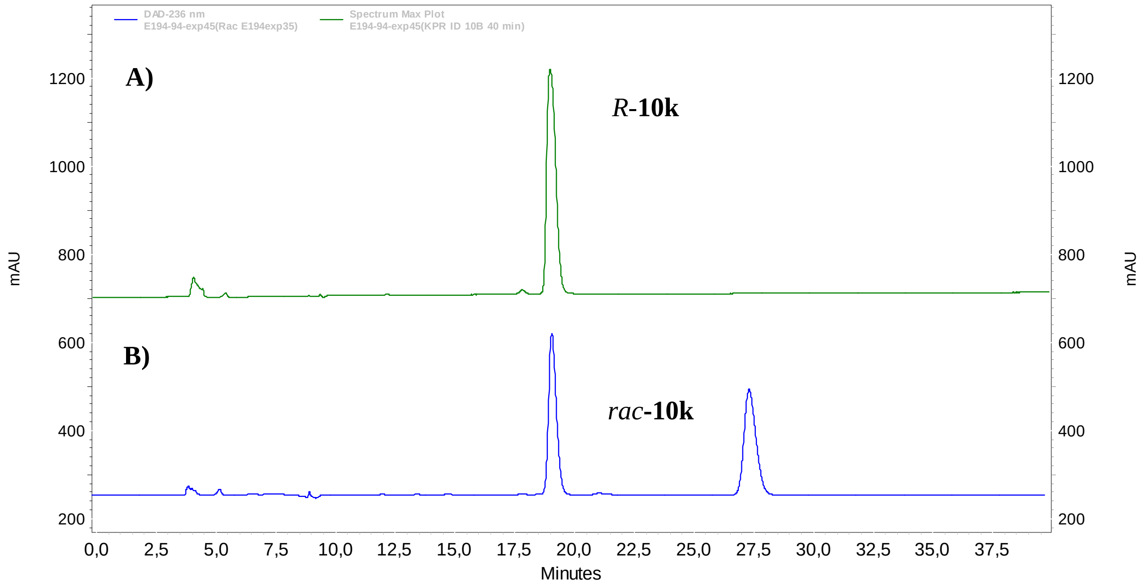

Figure S7. CPHPLC chromatogram of *R*-**10k** from KPR$_{Ecoli}$ catalysis (**A**) and *rac*-**10k** (**B**). Conditions: CHIRALPACK® ID, flow rate 0.8 mL min^{-1} at 30 °C and detection at 254 nm. Isocratic elution hexane:^{i}PrOH 90:10 (v/v). tr (*R*) = 19.2 min and tr (*S*) = 27.8 min.

(*R*)-3-Oxo-2-oxaspiro[4.4]nonan-4-yl 4-bromobenzoate (*R*-**10l**).

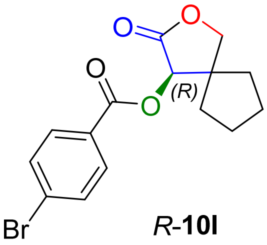

R-**10l**

Synthesis of title compound was performed as described for *R*-**10j** starting from *R*-**5l** (65 mg). *R*-**10l** was obtained as a yellow oil (96

mg, 68% yield). ^{1}H NMR (400 MHz, $CDCl_3$) δ 7.95 (d, J = 8.6 Hz, 2H), 7.62 (d, J = 8.6 Hz, 2H), 5.75 (s, 1H), 4.23 (d, J = 9.0 Hz, 1H), 4.17 (d, J = 9.9 Hz, 1H), 2.08 – 1.90 (m, 1H), 1.78 – 1.53 (m, 7H). ^{13}C NMR (101 MHz, $CDCl_3$) δ 172.3, 164.8, 132.2, 131.6, 129.2, 127.8, 76.0, 74.0, 51.3, 33.8, 30.9, 24.9, 24.9. ee: ≥99%. $[\alpha]_{20}^{D}$ = + 35.1 (c = 1 in MeOH).

***rac*-3-Oxo-2-oxaspiro[4.4]nonan-4-yl 4-bromobenzoate** (*rac*-**10l**).

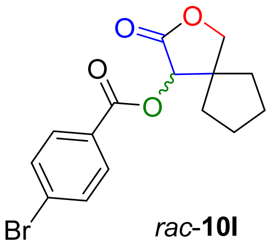

The aldol precursor **3l** was prepared as described for 2*R*,3*S*-**5b**, starting from **2l** (164 mg, 1 eq, 0.1 M concentration in the reaction) and **1a** (30 mg, 1 eq, 0.1 M concentration in the reaction). Chemical reduction was carried out as described for *rac*-**10a**. Benzoylation of *rac*-**5l** (35 mg) was performed as describe for *R*-**10j**. The title compound was obtained as a yellow oil (39 mg, 51%). NMR spectra were indistinguishable from that of *R*-**10l**.

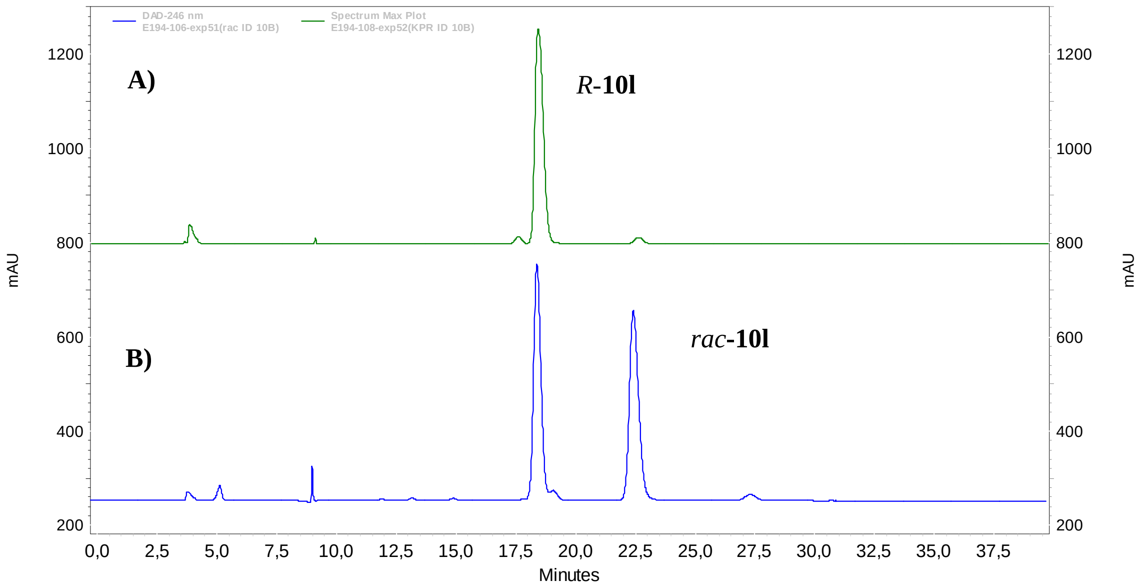

Figure S8. CPHPLC chromatogram of *R*-**10l** from KPR$_{Ecoli}$ catalysis (**A**) and *rac*-**10l** (**B**). Conditions: CHIRALPACK® ID, flow rate 0.8 mL min^{-1} at 30 °C and detection at 254 nm. Isocratic elution Hexane:^{i}PrOH 90:10 (v/v). tr (*R*) = 18.8 min and tr (*S*) = 22.8 min.

(*R*)-3-Oxo-2-oxaspiro[4.4]nonan-4-yl 4-bromobenzoate (*R*-**10m**).

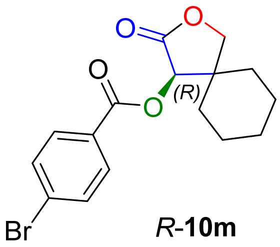

R-**10m**

Synthesis of title compound was performed as described for *R*-**10j** starting from *R*-**5m** (26 mg) in acetonitrile (5 mL). *R*-**10m** was obtained as a yellow oil (9 mg, 17% yield). ^{1}H NMR (400 MHz, $CDCl_3$) δ 7.95 (d, *J* = 8.6 Hz, 1H), 7.62 (d, *J* = 8.6 Hz, 1H), 5.59 (s, 1H), 4.44 (d, *J* = 9.3 Hz, 1H), 4.07 (dd, *J* = 9.3, 1.3 Hz, 1H), 1.80 – 1.32 (m, 10H). ^{13}C NMR (101 MHz, $CDCl_3$) δ 172.3, 164.7, 132.0, 131.5, 129.1, 127.7, 75.5, 73.4, 43.8, 33.7, 29.7, 27.2, 25.2, 22.7. ee: 98%. $[\alpha]_{20}^{D}$ = + 12.7 (*c* = 0.75 in MeOH).

***rac*-3-oxo-2-oxaspiro[4.5]decan-4-yl 4-bromobenzoate** (*rac*-**10m**).

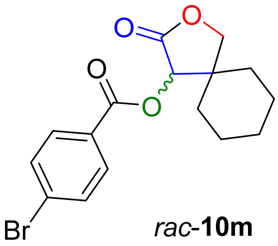

rac-**10m**

The aldol precursor **3m** was prepared following the procedure described for 2*R*,3*S*-**5b**, starting from **2m** (156 mg, 1 eq, 0.1 M concentration in the reaction) and **1a** (30 mg, 1 eq, 0.1 M concentration in the reaction). Chemical reduction was performed as described for *rac*-**7a**. Benzoylation of *rac*-**10m** was performed as describe for *R*-**10j**. The title compound was obtained as a yellow oil (26 mg, 17%). NMR spectra were indistinguishable from that of *R*-**10m**.

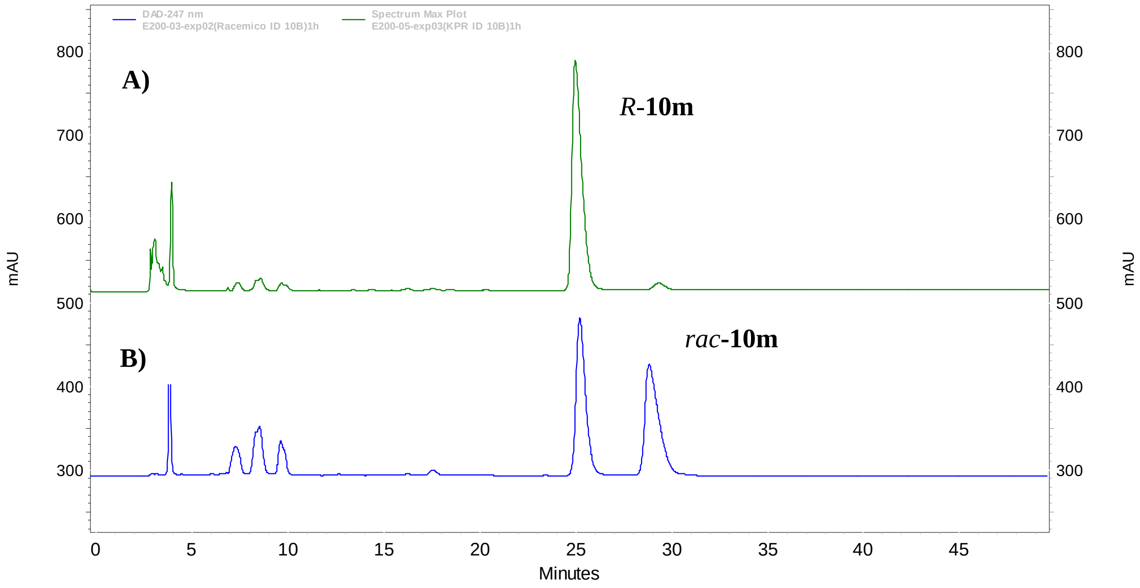

Figure S9. CPHPLC chromatogram of *R*-**10m** from KPR_{Ecoli} catalysis (**A**) and *rac*-**10m** (**B**). Conditions: CHIRALPACK® ID, flow rate 1 mL min^{-1} at 30 °C and detection at 254 nm. Isocratic elution Hexane:^{i}PrOH 90:10 (v/v). tr (*R*) = 25.6 min, tr (*S*) = 29.2 min.

(*R*)-3-oxo-2-oxaspiro[4.6]undecan-4-yl 4-bromobenzoate (*R*-**10n**).

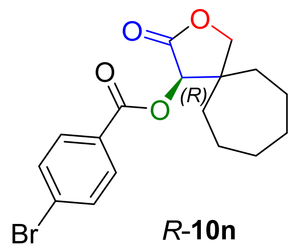

Synthesis of title compound was performed as described for *R*-**10j** starting from *R*-**5n** (20 mg) in acetonitrile (5 mL). *R*-**10n** was obtained as a yellow oil (9 mg, 23% yield). ^{1}H NMR (400 MHz, $CDCl_3$) δ 7.95 (dd, *J* = 8.7, 2.3 Hz, 2H), 7.70 – 7.58 (m, 2H), 5.63 (s, 1H), 4.26 (d, *J* = 9.1 Hz, 1H), 4.04 (d, *J* = 9.1 Hz, 1H), 2.4 – 2.3 (m, 1H), 2.10 – 1.85 (m, 1H), 1.80 – 1.24 (m, 10H). ^{13}C NMR (101 MHz, $CDCl_3$) δ 172.9, 165.1, 132.5, 132.0, 129.5, 128.2, 77.6, 75.4, 47.3, 36.9, 31.2, 30.5, 30.3, 23.7, 23.3. ee: 98%. $[\alpha]_{20}^{D}$ = + 31.2 (*c* = 0.4 in MeOH).

***rac*-3-oxo-2-oxaspiro[4.6]undecan-4-yl 4-bromobenzoate** (*rac*-**10n**).

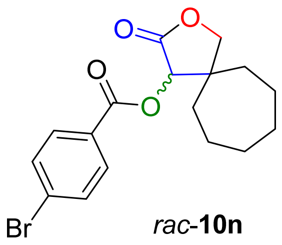

The aldol precursor **3n** was prepared following the procedure described for 2*R*,3*S*-**5b**, starting from **2n** (283.4 mg, 1 eq, 0.1 M concentration in the reaction) and **1a** (50 mg, 1 eq, 0.1 M concentration in the reaction). Chemical reduction was performed as described for *rac*-**7a**. Benzoylation of *rac*-**10n** was performed as describe for *R*-**10j**. The title compound was obtained as a yellow oil (20 mg, 7%). NMR spectra were indistinguishable from that of *R*-**10n**.

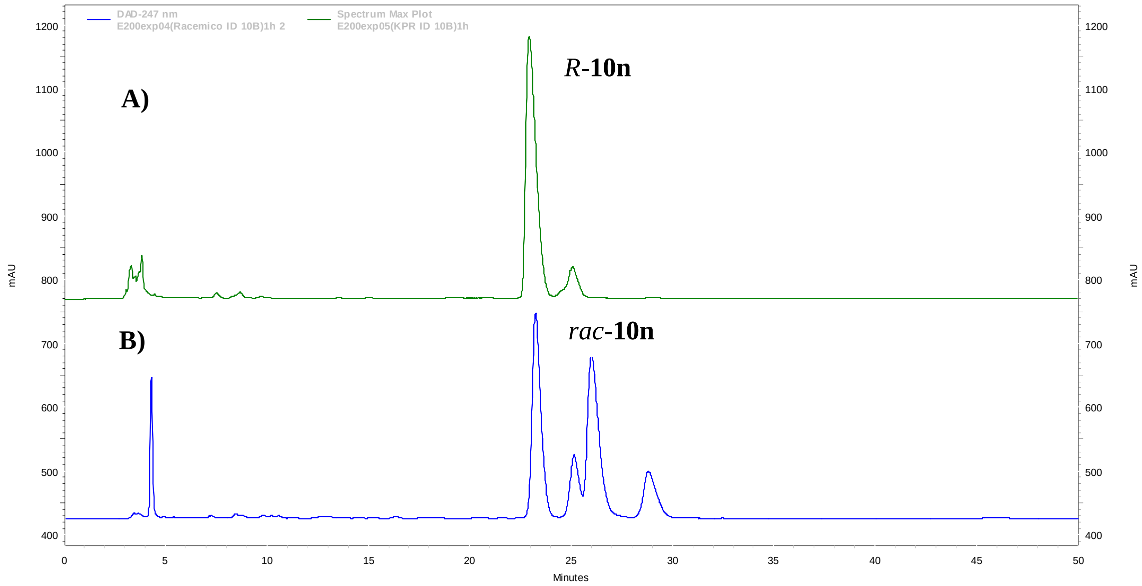

Figure S10. CPHPLC chromatogram of *R*-**10n** from KPR$_{Ecoli}$ catalysis (**A**) and *rac*-**10n** (**B**). Conditions: CHIRALPACK® ID, flow rate 1 mL min^{-1} at 30 °C and detection at

254 nm. Isocratic elution Hexane:^{i}PrOH 90:10 (v/v). tr (*R*) = 22.9 min, tr (*S*) = 25.9 min.

Biocatalytic aldol addition of sodium piruvate (2a) to aldehydes (1b-g). Analytical scale

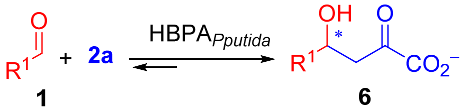

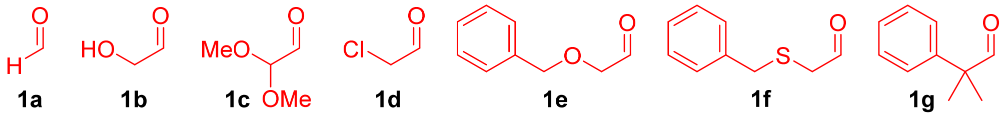

Reactions were carried out at analytic level as follows: The reactions (500 µL total volume) were conducted in Eppendorf tubes (1.5 mL) and placed in a vortex mixer (1000 rpm) at 25ºC. To a solution of the aldehyde (**1b-g**) (dissolved in 50 mM sodium phosphate buffer pH 7.0, 100 mM final concentration in the reaction; in case of partial water solubility, DMF (20% v/v in the reaction was used), a solution of sodium pyruvate (**2a**) (25 µL of a 2.0 M aqueous stock solution, pH 6.5-7.0, 100 mM final concentration in the reaction, *(Caution: The solution of sodium pyruvate has to be freshly prepared before use!)* was added. The reaction was started by adding of HBPA$_{Pputida}$ *wild-type* (125 µL of a stock solution 0.029 U mL^{-1}, 4 mg mL^{-1} in 50 mM TEA buffer, 50 mM NaCl, 0.5 mM EDTA and 50% (v/v) of glycerol, 0.007 U mL^{-1}, 1 mg protein mL^{-1} final concentration in the reaction, for enzyme reactions with **1b-c**, and **1e**). Or HBPA$_{Pputida}$ H205A (132 µL of a stock solution 0.009 U mL^{-1}, 3.8 mg mL^{-1} in 50 mM TEA buffer, 50 mM NaCl, 0.5 mM EDTA and 50% (v/v) of glycerol, 0.00324 U mL^{-1}, 1 mg mL^{-1} protein final concentration in the reaction, for enzyme reactions with **1d**, **1f**, and **1g**). The reaction mixture was placed in a vortex mixer (1000 rpm) at 25 °C for 24 h. Samples were withdrawn immediately after the enzyme addition (0 h) and after 24 h and analyzed by HPLC as described above.

Biocatalytic reduction of 4-hydroxy-2-oxacids (6b-g). Substrate scope of ketoreductases. Analytical scale.

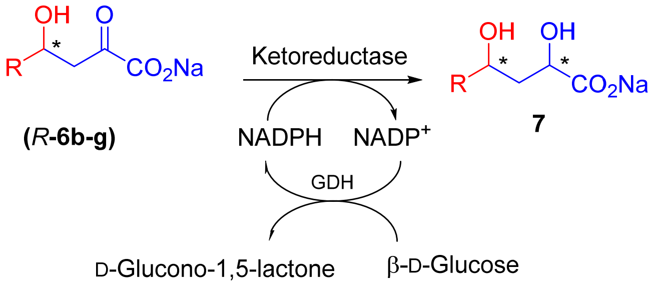

Scheme S7. Biocatalytic reduction of aldol adducts (*R*-**6b-g**) catalyzed by $DpkA_{Psyrin}$ and KPR_{Ecoli}.

Biocatalytic reduction catalyzed by KPR_{Ecoli} or $DpkA_{Psyrin}$: Reactions were carried out at analytic level as follows: The reactions (500 µL total volume) were conducted in Eppendorf tubes (1.5 mL) and placed in a vortex mixer (100 rpm) at 25 ºC. The reduction mixture solutions (250 µL) were prepared by adding; KPR_{Ecoli} (125 µL of a stock solution of 52 U mL^{-1} ,4 mg mL^{-1} in 20 mM TEA buffer pH 7.0, 100 mM NaCl, and 50% (v/v) of glycerol, 13 U mL^{-1} final concentration in the reaction) or $DpkA_{\text{Psyrin}}$ (125 µL of a stock solution of 2.6 10^{-2} U mL^{-1}, 4 mg mL^{-1} in 20 mM TEA buffer pH 7.0, 100 mM NaCl, and 50% (v/v) of glycerol, 6.5 10^{-3} U mL^{-1} final concentration in the reaction), GDH (83 µL of a stock solution 20.8 U mL^{-1}, 5.2 mg mL^{-1} in 10 mM HEPES buffer pH 6.5, 50 mM NaCl, and 50% (v/v) of glycerol, 3.5 U mL^{-1} final concentration in the reaction), glucose (18 mg, 4 eq, 0.2 M final concentration in reaction) and $NADP^+$ (1.9 mg, 5 mM in reaction). The reactions were started by the addition of a sample of the corresponding aldol reaction (250 µL), containing adducts **6b-g** (≈ 100 mM as the basis of calculation). Samples were withdrawn immediately after the addition of aldol substrate (0 h) and after 24 h and analyzed by HPLC as described above.

One-pot two-step stereoselective synthesis of 4-substituted-2-hydroxy-4-butyrolactones 8 by tandem biocatalytic aldol-reduction reactions catalyzed by HBPA$_{Pputida}$ and DpkA$_{Psyrin}$.

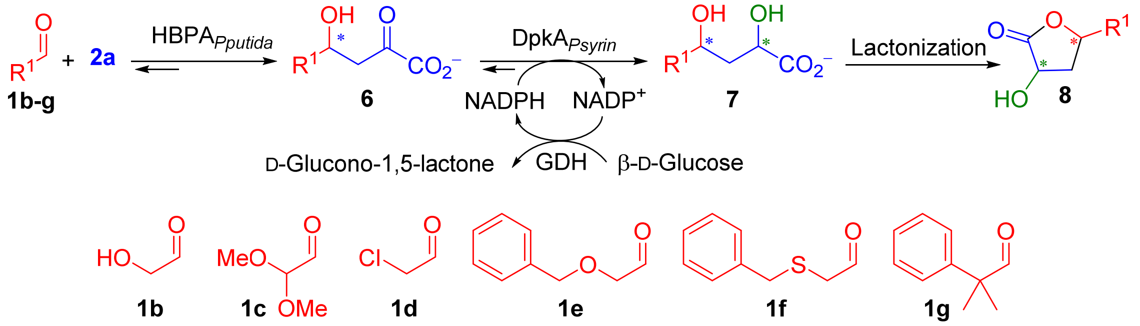

Scheme 8. One-pot two-step synthesis of 4-substituted-2-hydroxy-4-butyrolactones **8** by tandem biocatalytic aldol-reduction reactions catalyzed by HBPA$_{Pputida}$ and DpkA$_{Psyrin}$.

The reactions combining HBPA$_{Pputida}$ **and** DpkA$_{Psyrin}$. **were carried out as follows:**

(2*S*,4*R*)-4-(hydroxymethyl)-2-(hydroxy)-4-butyrolactone (2*S*,4*R*-**8b**).

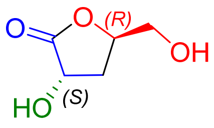

2*S*,4*R*-**8b**

Aldol addition (1st): The reaction (1 mmol scale, 8.3 mL total volume) was conducted in a round-bottom flask (100 mL) at 25 °C for 24 h and orbitally stirred at 250 rpm. Sodium pyruvate **2b** (91.6 mg, 1 mmol, 1 eq, 0.1 M in the reaction) and glycolaldehyde dimer **1b** (50 mg, 1 mmol monomer, 1 eq, 0.1 M in the reaction) were dissolved in 5.9 mL of water. Finally, reaction was started by adding the enzyme (HBPA *wild-type*, 2 mL of a stock solution 0.029 U mL^{-1}, 4 mg mL^{-1} in 50 mM TEA buffer, 50 mM NaCl, 0.5 mM EDTA and 50% (v/v) of glycerol, 0.007 U mL^{-1}, 1 mg protein mL^{-1} final concentration in the reaction). Reaction was monitored by HPLC and samples were withdrawn immediately after the enzyme addition (0 h) and after 24 h as described above. **Aldol reduction (2nd)**. After 24 h, the reduction reaction (16.6 mL final volume) was carried out as follows: to the aldol reaction mixture were added glucose (598 mg, 4 mmol, 4 eq, 0.2 M final concentration in the reaction), GDH (2.75 mL of a stock solution 20.8 U mL^{-1}, 5.2 mg mL^{-1} in 10 mM HEPES buffer pH 6.5, 50 mM NaCl, and 50% (v/v) of glycerol, 3.4 U mL^{-1} final concentration in the reaction) and DpkA$_{Psyrin}$ (4.15 mL of a stock solution 2.6 10^{-2} U mL^{-1}, 4 mg mL^{-1} in 20 mM TEA buffer pH 7.0, 100 mM NaCl, and 50%

(v/v) of glycerol, 6.5 10^{-3} U mL^{-1} final concentration in the reaction). The reaction was started by adding a solution of $NADP^+$ (1.4 mL of stock solution 69 mM in 1 M sodium phosphate buffer pH 8.0, 5 mM final concentration in the reaction). The mixture reaction was stirred at room temperature and the reaction was monitored by HPLC as described above. After no aldol adduct was detected by HPLC (24 h), methanol (200 mL) was added under stirring. The mixture was filtered through Celite® and the filter cake washed with methanol (3 x 50 mL). The organic solvent was removed and the pH of the remaining aqueous solution was adjusted to 9.0 with 1 M NaOH. Then, water was added up to a final volume of 40 mL. The product purification was started with anion exchange chromatography (DOWEX 1X8 ion exchange resin (50-100 mesh) in HCO_2^- form, column: 44 cm, Ø = 1,6 cm). The sample was loaded onto the column and the resin was washed with plain water (90 mL). The bound fractions with the compound were eluted with a solution of 1 M HCO_2H (fraction volume 30 mL). Fractions containing the product were pooled freeze-dried, dissolved in methanol (30 mL), absorbed in silica and purified by column chromatography on silica with a step gradient of hexane:EtOAc: 100:0, 200 mL, 0:100, 300 mL. Pure fractions were pooled and the solvent removed under vacuum affording the lactone 2*S*,4*R*-**8b** as a yellow oil (30 mg, 27%). ^{1}H NMR (400 MHz, D_2O) δ 4.83 (ddt, *J* = 8.9, 4.4, 2x2.7 Hz, 1H), 4.73 (t, *J* = 2x8.9 Hz, 1H), 3.85 (dd, *J* = 12.9, 2.7 Hz, 1H), 3.68 (dd, *J* = 12.9, 4.5 Hz, 1H), 2.54 (ddd, *J* = 13.5, 9.0, 2.9 Hz, 1H), 2.34 (dt, *J* = 13.5, 2x8.9 Hz, 1H). ^{13}C NMR (101 MHz, D_2O) δ 180.0, 79.5, 67.0, 62.9, 31.9. Minor diastereomer selected key signals ^{1}H NMR (400 MHz, D_2O) δ 4.50 (dd, *J* = 3.0, 0.9 Hz, 1H), 3.83 (dd, *J* = 13.0, 2.6 Hz, 1H). ^{13}C NMR (101 MHz, D_2O) peaks detected by HSQC experiment: δ 73.1, 62.1. $[\alpha]_{20}^{D}$ = –49.6 (*c* = 1, in MeOH), dr: 98:2. ESI-TOF m/z: Calcd for $[M+Na^+]$ $C_5H_8O_4Na^+$:155.0315, found $[M+Na^+]$: 155.0319.

(2*S*,4*R*)-4-(dimethoxymethyl)-2-(hydroxy)-4-butyrolactone (2*S*,4*R*-**8c**)

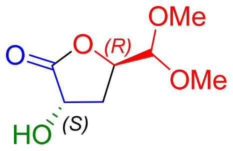

2*S*,4*R*-**8c**

The title compound was prepared as described for 2*S*,4*R*-**8b**. Starting from **1c** ([**1c**] = [**2a**] = 100 mM in the reaction, $V_{\text{aldol reaction}}$ = 10,6 mL. 2*S*,4*R*-**8c** was obtained as a yellow oil (54 mg, 29%). In this case the product was eluted with a step gradient of Hexane:EtOAc: 100:0, 200 mL, 75:25, 200 mL, 50:50, 200 mL and 25:75, 500 mL on column chromatography on silica. ^{1}H NMR (400 MHz, MeOD) δ 4.42 (ddd, *J* = 8.9, 3.2, 2.2 Hz, 1H), 4.32 (t, *J* = 2x8.8 Hz, 1H), 4.26 (d, *J* = 3.2 Hz, 1H),

3.32 (s, 3H), 3.31 (s, 3H), 2.43 (ddd, J = 13.4, 8.9, 2.3 Hz, 1H), 1.93 (dt, J = 13.4, 2x8.8 Hz, 1H). ^{13}C NMR (101 MHz, MeOD) δ 179.5, 106.2, 77.7, 67.7, 57.4, 56.7, 32.2. No signals were detected from minor diastereomers $[\alpha]_{20}^{D}$ = – 35.7 (c = 0.5, in MeOH), dr: 98:2. ESI-TOF m/z: Calcd for [M+Na$^+$] $C_7H_{12}O_5Na^+$:199.0577, found [M+Na$^+$]: 199.0566.

(2*S*,4*R*)-4-(chloromethyl)-2-(hydroxy)-4-butyrolactone (2*S*,4*R*-**8d**).

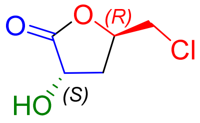

2*S*,4*R*-**8d**

Synthesis of aldol adduct **6d** was prepared using HBPA$_{Pputida}$ H205A variant with the procedure described 2*S*,4*R*-**8d**, starting from **1d** ([**1d**] = [**2a**] = 100 mM in the reaction, V$_{\text{aldol reaction}}$ = 7,6 mL). 2*S*,4*R*-**8d** was obtained as a yellow oil (30 mg, 26%). In this case the product was eluted with a step gradient of Hexane:EtOAc: 100:0, 200 mL, 75:25, 200 mL and 50:50,750 mL on column chromatography on silica. ^{1}H NMR (400 MHz, $CDCl_3$) δ 4.92 (ddt, J = 8.7, 4.5, 2x3.4 Hz, 1H), 4.68 (dd, J = 8.9, 7.7 Hz, 1H), 3.78 (dd, J = 12.1, 4.5 Hz, 1H), 3.67 (dd, J = 12.1, 3.4 Hz, 1H), 2.58 (ddd, J = 13.8, 8.9, 3.3 Hz, 1H), 2.42 (ddd, J = 13.8, 8.6, 7.7 Hz, 1H). ^{13}C NMR (101 MHz, $CDCl_3$) δ 177.1, 76.5, 67.5, 46.8, 33.5. Minor diastereomer selected key signals ^{1}H NMR (400 MHz, $CDCl_3$) δ 4.42 (dd, J = 7.4, 4.1 Hz, 1H), 3.76 (dd, J = 5.2, 1.3 Hz, 1H), 3.60 (dd, J = 7.4, 5.3 Hz, 2H), 2.20 – 2.12 (m, 1H), 1.94 (ddd, J = 14.4, 9.4, 7.4 Hz, 1H). ^{13}C NMR (101 MHz, $CDCl_3$) peaks detected by HSQC experiment: δ 68.8, 49.4. The NMR data matched that reported in the literature.[16] $[\alpha]_{20}^{D}$ = – 59.7 (c = 0.5, in MeOH), dr: 94:6. ESI-TOF m/z: Calcd for [M+Na$^+$] $C_5H_7ClO_3Na^+$: 172.9976, found [M+Na$^+$]: 245.0779.

(2*S*,4*R*)-4-((benzyloxy)methyl)-2-(hydroxy)-4-butyrolactone (2*S*,4*R*-**8e**).

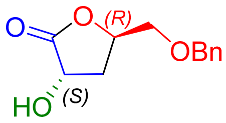

2*S*,4*R*-**8e**

The synthesis of aldol intermediate **6b** was conducted in an Erlenmeyer (50 mL). Reaction volume was 5.3 mL. Benzyloxyacetaldehyde (**1e**) (80.0 mg, 5.3 mmol, 1 eq, 0.1 M in the reaction) was dissolved in DMF (1.1 mL, 20% (v/v) final concentration in the reaction) and sodium pyruvate **2a** (58.6mg, 5.3 mmol, 1 eq, 0.1 M final concentration in the reaction) dissolved in water (2.9 mL) was added. Finally, reaction was started by adding the enzyme (HBPA *wild-type*, 1.3 mL of a stock solution 0.029 U mL^{-1}, 4 mg mL^{-1} in 50 mM TEA buffer, 50 mM NaCl, 0.5 mM EDTA and 50% (v/v) of glycerol, 0.007 U mL^{-1}, 1 mg protein mL^{-1} final concentration in the

reaction). Reaction was monitored by HPLC and samples were withdrawn immediately after the enzyme addition (0 h) and after 24 h as described above. **Aldol reduction**, the reduction of aldol adduct with DpkA$_{Psyrin}$ was performed as describe for 2*S*,4*R*-**8b**. In this case, the product was obtained after anion exchange purification as a white solid (41 mg, 30%). Containing: (2*S*,4*R*)-5-(benzyloxy)-2,4-dihydroxypentanoic acid (2*S*,4*R*-**7e**): ^{1}H NMR (400 MHz, D_2O) δ 7.49 – 7.06 (m, 5H), 4.53 (s, 2H), 4.27 (dd, *J* = 6.6, 5.4 Hz, 1H), 4.05 – 3.94 (m, 1H), 3.51 (dd, *J* = 10.7, 3.7 Hz, 1H), 3.44 (dd, *J* = 10.7, 6.9 Hz, 1H), 1.93 (dt, *J* = 14.5, 2x5.1 Hz, 1H), 1.80 (ddd, *J* = 14.7, 8.1, 6.6 Hz, 1H).^{13}C NMR (101 MHz, D_2O) δ 177.6, 137.3, 128.7, 128.7, 128.5, 128.4, 73.0, 73.3, 66.7, 67.4, 37.0, and (2*S*,4*R*-**8e**) ^{1}H NMR (400 MHz, D_2O) δ 7.49 – 7.17 (m, 5H), 4.80 (ddt, *J* = 9.0, 5.4, 2x2.8 Hz, 1H), 4.64 – 4.55 (m, 1H), 4.52 (s, 2H), 3.71 (dd, *J* = 11.7, 2.7 Hz, 1H), 3.60 (dd, *J* = 11.7, 5.0 Hz, 1H), 2.41 (ddd, *J* = 13.4, 8.9, 2.9 Hz, 1H), 2.23 (dt, *J* = 13.4,2x8.9 Hz, 1H).^{13}C NMR (101 MHz, D_2O) δ 179.8, 137.3, 128.7, 128.7, 128.5, 128.4, 78.0, 73.0, 70.9, 66.6, 32.2. $[\alpha]_{20}^{D}$ = – 33.3 (*c* = 0.5, in MeOH), dr: 94:6. ESI-TOF m/z: Calcd for [M+Na$^+$] $C_{12}H_{14}O_4Na^+$: 245.0784, found [M+Na$^+$]: 245.0779.

(2*S*,4*R*)-4-((benzylthio)methyl)-2-(hydroxy)-4-butyrolactone (2*S*,4*R*-**8f**).

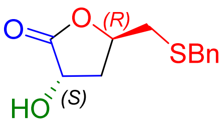

2*S*,4*R*-**8f**

Synthesis of aldol adduct **6f** was prepared using HBPA$_{Pputida}$ H205A variant with the procedure described 2*S*,4*R*-**8e**, starting from **1f** ([**1f**] = [**2a**] = 100 mM in the reaction, $V_{\text{aldol reaction}}$ = 10.2 mL). The **Aldol reduction** with DpkA$_{Psyrin}$ was performed as describe for 2*S*,4*R*-**8b**. After no aldol adduct was detected by HPLC (24 h), methanol (200 mL) was added under stirring. The mixture was filtered through Celite® and the filter cake washed with methanol (3 x 50 mL). The solvent was removed under vacuum. The residue was re-suspended in toluene (200 mL) and p-toluene-sulfonic acid (19.5 mg, 0.01 eq) was added. The mixture was heated to reflux for 16h, using a dean-stark apparatus. This is to promote the formation of lactone.

After which it was cooled, 5% $NaHCO_3$ (100 mL) was added, toluene was removed and the aqueous solution was extracted with CH_2Cl_2 (3 × 50 mL). Aqueous phase was then acidified to pH 2 with 3 M HCl and extracted with EtOAc (3 × 50 mL). The organic layer was washed with water (3 × 50 mL) and brine (3 × 50 mL), and dried over $MgSO_4$. The sample was absorbed in silica and purified by column chromatography on silica with a step gradient of hexane:EtOAc: 100:0, 200 mL, 75:25, 200 mL, 50:50,

200mL and 75:25, 500 mL. Pure fractions were pooled and the solvent removed under vacuum affording the lactone 2*S*,4*R*-**8f** as a brown oil (92 mg, 38%). ^{1}H NMR (400 MHz, $CDCl_3$) δ 7.40 – 7.22 (m, 5H), 4.76 (dtd, *J* = 7.9, 2x5.4, 3.9 Hz, 1H), 4.61 (td, *J* = 2x8.1, 2.2 Hz, 1H), 3.76 (d, *J* = 2.9 Hz, 2H), 2.66 (d, *J* = 5.5 Hz, 2H), 2.42 – 2.27 (m, 2H). ^{13}C NMR (101 MHz, $CDCl_3$) δ 176.9, 137.4, 129.1, 128.7, 127.4, 77.5, 67.3, 37.1, 35.3, 34.6. Minor diastereomer selected key signals: ^{1}H NMR (400 MHz, $CDCl_3$) δ 4.56 – 4.42 (m, 1H), 2.01 (dt, *J* = 12.7, 10.3 Hz, 1H). ^{13}C NMR (101 MHz, $CDCl_3$) δ no signals detected. $[\alpha]_{20}^{D}$ = – 30.4 (*c* = 0.5, in MeOH), dr: 87:13. ESI-TOF m/z: Calcd for $[M+Na^+]$ $C_{12}H_{14}SO_3Na^+$: 261.0556, found $[M+Na^+]$: 261.0551.

(2*S*,4*R*)-4-(2-phenoxypropan-2-yl)-2-(hydroxy)-4-butyrolactone (2*S*,4*R*-**8g**).

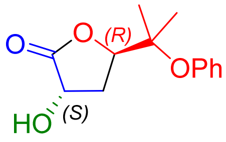

2*S*,4*R*-**8g**

Synthesis of aldol adduct **6g** was prepared as described for 2*S*,4*R*-**8e**. Starting from **1g** ([**1g**] = [**2a**] = 100 mM in the reaction, $V_{aldol\ reaction}$ = 6 mL). The **Aldol reduction** with DpkA*Psyrin* was performed as describe for 2*S*,4*R*-**8b**. Lactonization reaction and purification were carried out as described for 2*S*,4*R*-**8f** was obtained as a white solid (52 mg, 34%). ^{1}H NMR (400 MHz, $CDCl_3$) δ 7.32 – 7.22 (m, 2H), 7.16 – 7.06 (m, 1H), 6.92 (dd, *J* = 8.6, 1.1 Hz, 2H), 4.76 (t, *J* = 2x8.7 Hz, 1H), 4.45 (dd, *J* = 9.0, 2.2 Hz, 1H), 2.90 (ddd, *J* = 13.4, 9.0, 2.3 Hz, 1H), 2.39 – 2.27 (m, 1H), 1.32 (s, 3H), 1.29 (s, 3H). ^{13}C NMR (101 MHz, $CDCl_3$) δ 178.0, 153.9, 129.2, 124.2, 123.8, 83.7, 80.3, 67.3, 31.4, 23.6, 22.6. No signals were detected from minor diastereomers. $[\alpha]_{20}^{D}$ = – 47.2 (*c* = 0.5, in MeOH), dr: 98:2. ESI-TOF m/z: Calcd for $[M+Na^+]$ $C_{13}H_{16}O_4Na^+$: 259.0941, found $[M+Na^+]$: 259.0958.

Kinetic Studies.

Substrates (3a-m) and (**6b-g**) **stock solution preparation.**

Stock solutions of aldol adduct (**3a-m)** in H_2O pH 7.0 were prepared using the best 2-oxoacid aldolase variant as described above. Likewise for stock solutions of aldol adduct (**6b-g**) was prepared in 50 mM phosphate buffer pH 7.0 using the best HBPA variant as described above (Table S4). In both cases, enzymes were removed from the mixture using Amicon Ultra-15, PLBC Ultracel-PL membrane, 3 KDa cutoff.

Table S4. Adduct aldol stock solutions in water.

Substrate **3**	Aldolases	Concentration/mM[a]	ee[b]/%
**3a**	YfaU$_{Ecoli}$ *wt*	85	-
3*S*-**3b**	YfaU$_{Ecoli}$ W23V	92	98
3*S*-**3c**		95	99
3*S*-**3d**		88	98
3*S*-**3f**		89	99
3*S*-**3g**		98	98
3*S*-**3h**		98	94
3*R*-**3b**	KPHMT$_{Ecoli}$ *wt*	94	95
3*R*-**3c**	KPHMT$_{Ecoli}$ *wt*	98	95
3*R*-**3d**	KPHMT$_{Ecoli}$ I212A	97	96
3*R*-**3e**	KPHMT$_{Ecoli}$ I212A	82	-[c]
3*R*-**3g**	KPHMT$_{Ecoli}$ *wt*	84	87
3*R*-**3h**	KPHMT$_{Ecoli}$ I202A	80	75
**3j**	KPHMT$_{Ecoli}$ *wt*	88	-[d]
**3k**	KPHMT$_{Ecoli}$ *wt*	80	-[d]
**3l**	KPHMT$_{Ecoli}$ *wt*	96	-[d]
**4*R*-6b**	HBPA$_{Pputida}$ *wt*	82	94
**4*R*-6c**	HBPA$_{Pputida}$ *wt*	85	87

[a]Concentration was estimated on the basis of the conversion percentage of the aldol addition at 24h. [b]Determined by HPLC on a chiral stationary phase in our lab using procedure published in previous works [9].[c]Not determined. [d]Not applicable.

Steady-State Kinetic Studies of ketoreductases.

The kinetic parameters for KPR$_{Ecoli}$ and DpkA$_{Psyrin}$ were determined in a continuous assay method monitoring the oxidation of NADPH to $NADP^+$ at 340 nm (NADPH ε_{340} = 6.22 mM^{-1} cm^{-1}) using aldol adduct as substrates (Table S4). The reactions were monitored during 15 min measuring each 30 s. The assay mixture (0.3 mL) consisted of 50 mM Tris-HCl buffer pH 8.0, containing NADPH (0.16 mM), aldol adducts (1-60 mM) and appropriate amounts of enzymes (optimal range of enzyme concentration determined for each substrate, see S11-S32). One unit of activity was defined as the amount of ketoreductases that catalyzes the formation of 1 μmol $NADP^+$ per min at 30 °C. Measurements were carried out in triplicate independent experiments. To determine the kinetic parameters, data were fitted to the Michaelis-Menten kinetic model using the software GraphPad Prism version 5.0.

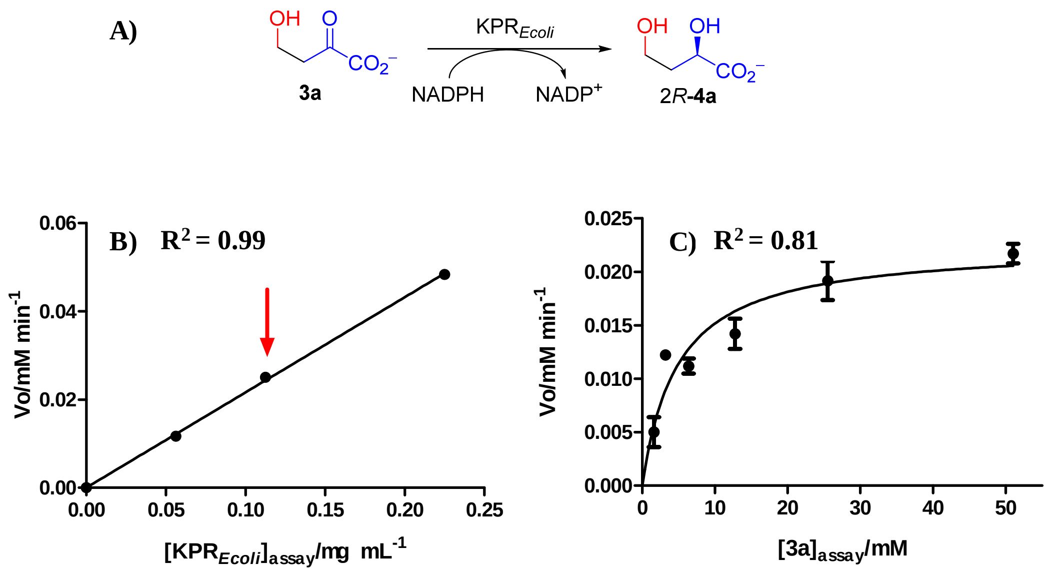

Figure S11. Reduction of **3a** to 2*R*-**4a** catalyzed by KPR$_{Ecoli}$. (**A**). Initial reaction rate (V_o) vs the enzyme concentration. The arrow shows the enzyme concentration selected to perform the enzyme assay (**B**) and V_o vs substrate concentration (0.2 (Km^{app}) ≤ [S] ≤ 20.2 (Km^{app})), adjusted to a Michaelis-Menten model by non-linear regression method (**C**). Each point is the mean of three independent experiments.

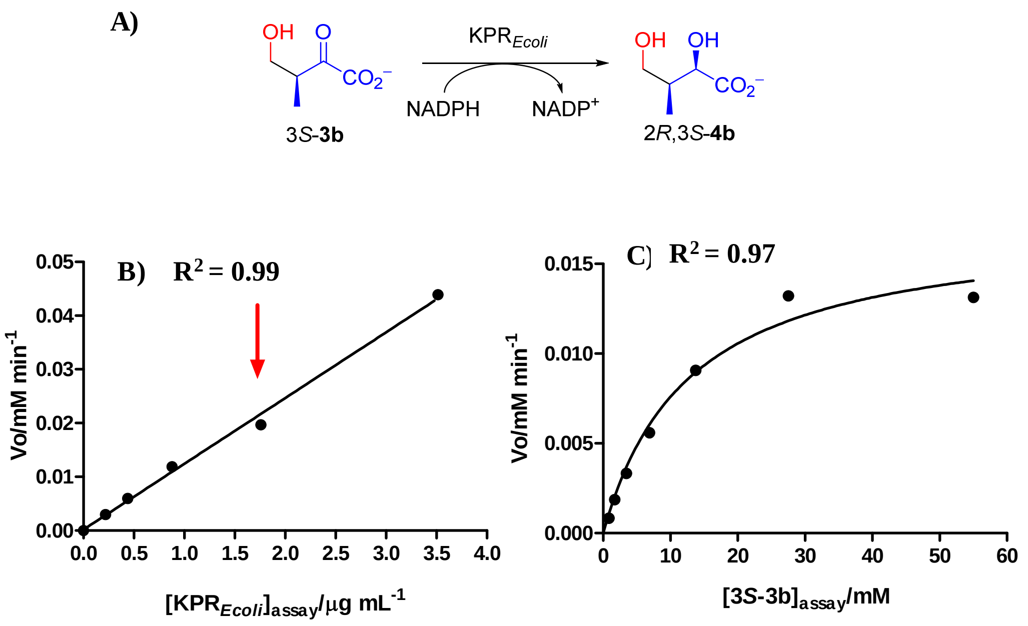

Figure S12. Reduction of 3*S*-**3b** to 2*R*,3*S*-**4b** catalyzed by KPR$_{Ecoli}$. (**A**). Initial reaction rate (V_o) vs the enzyme concentration. The arrow shows the enzyme concentration selected to perform the enzyme assay (**B**) and V_o vs substrate concentration (0.07 (Km^{app}) ≤ [S] ≤ 4.5 (Km^{app})), adjusted to a Michaelis-Menten model by non-linear regression method (**C**). Each point is the mean of three independent experiments.

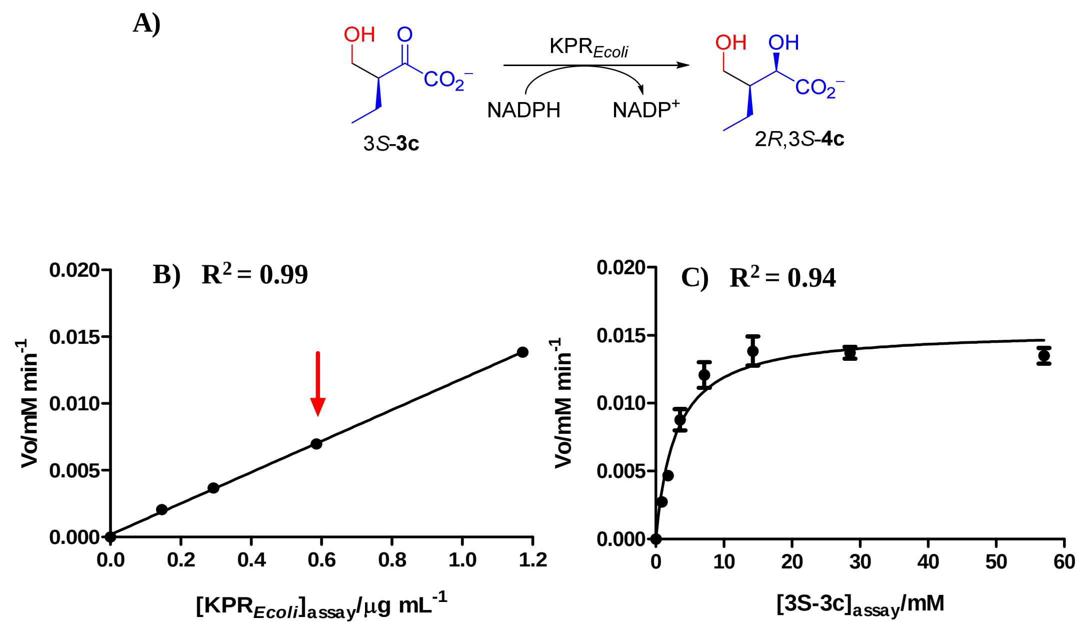

Figure S13. Reduction of 3*S*-**3c** to 2*R*,3*S*-**4c** catalyzed by KPR$_{Ecoli}$. (**A**). Initial reaction rate (V_o) vs the enzyme concentration. The arrow shows the enzyme concentration selected for the enzyme assay (**B**) and V_o vs substrate concentration (0.3 ($K\mathrm{m}^{app}$) ≤ [S] ≤ 19.3 ($K\mathrm{m}^{app}$)), adjusted to a Michaelis-Menten model by non-linear regression method (**C**). Each point is the mean of three independent experiments.

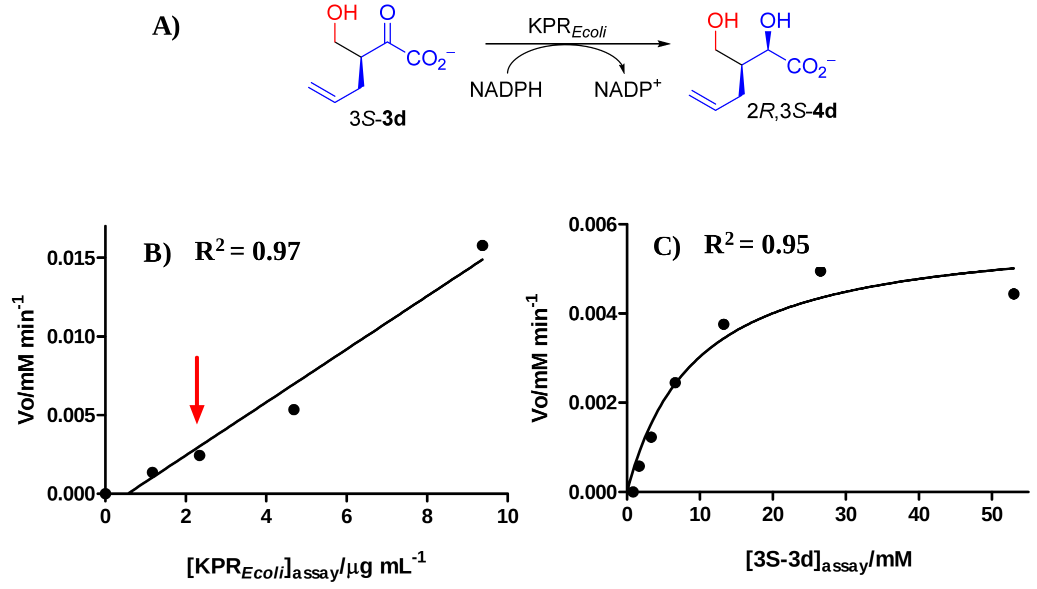

Figure S14. Reduction of 3*S*-**3d** to 2*R*,3*S*-**4d** catalyzed by KPR$_{Ecoli}$. (**A**). Initial reaction rate (V_o) vs the enzyme concentration. The arrow shows the enzyme concentration selected for the enzyme assay (**B**) and V_o vs substrate concentration (0.11 ($K\mathrm{m}^{app}$) ≤ [S]

≤ 5.8 (Km^{app})), adjusted to a Michaelis-Menten model by non-linear regression method (**C**). Each point is the mean of three independent experiments.

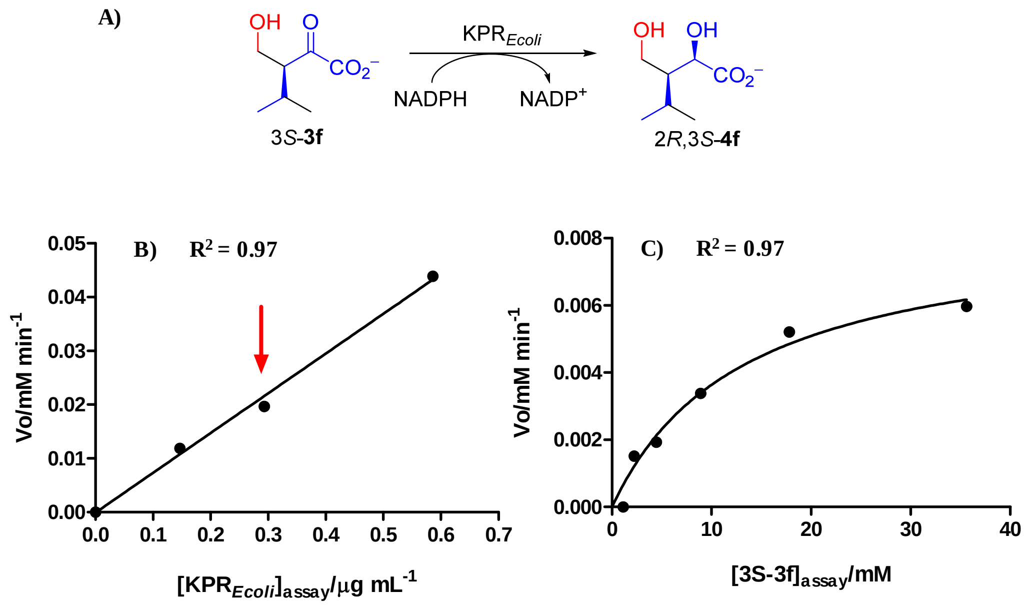

Figure S15. Reduction of 3*S*-**3f** to 2*R*,3*S*-**4f** catalyzed by KPR_{Ecoli}. (**A**). Initial reaction rate (V_o) vs the enzyme concentration. The arrow shows the enzyme concentration selected for the enzyme assay (**B**) and V_o vs substrate concentration (0.08 (Km^{app}) ≤ [S] ≤ 2.8 (Km^{app})), adjusted to a Michaelis-Menten model by non-linear regression method (**C**). Each point is the mean of three independent experiments.

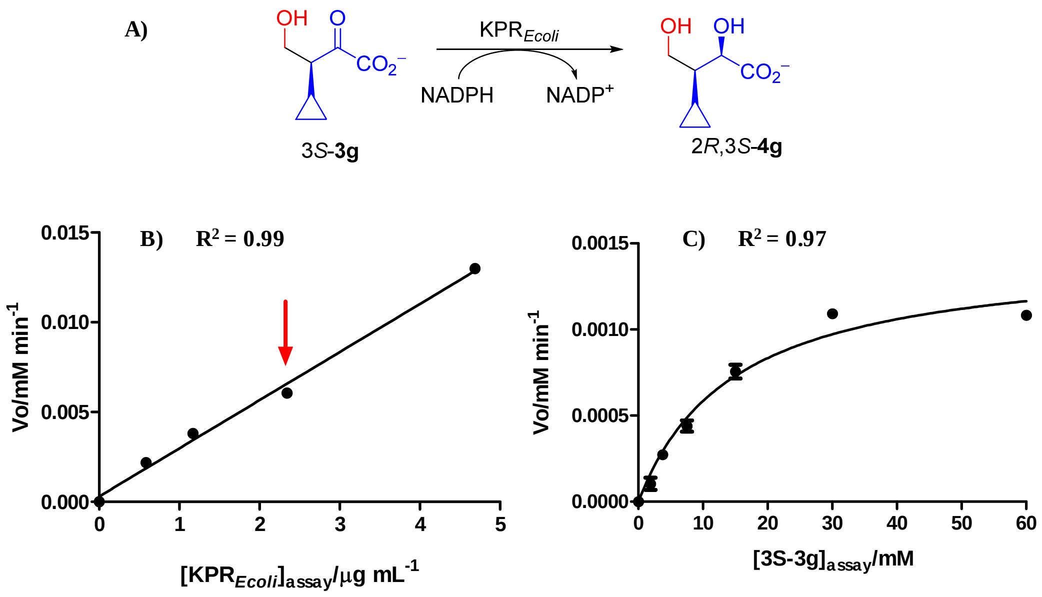

Figure S16. Reduction of 3*S*-**3g** to 2*R*,3*S*-**4g** catalyzed by KPR_{Ecoli}. (**A**). Initial reaction rate (V_o) vs the enzyme concentration. The arrow shows the enzyme concentration

selected for the enzyme assay (**B**) and V_o vs substrate concentration (0.07(Km^{app}) ≤ [S] ≤ 4(Km^{app})), adjusted to a Michaelis-Menten model by non-linear regression method (**C**). Each point is the mean of three independent experiments.

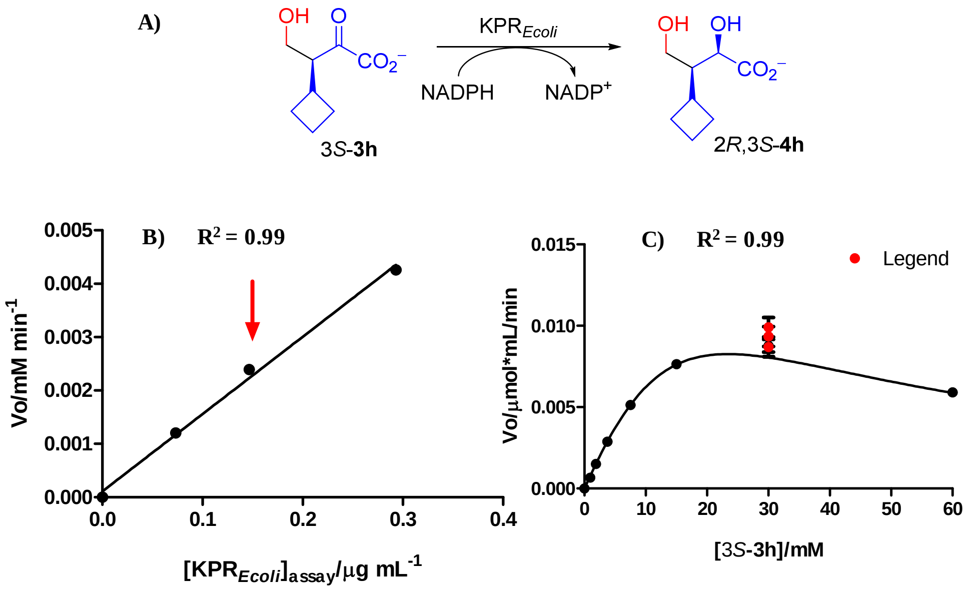

Figure S17. Reduction of 3*S*-**3h** to 2*R*,3*S*-**4h** catalyzed by KPR$_{Ecoli}$. (**A**). Initial reaction rate (V_o) vs the enzyme concentration. The arrow shows the enzyme concentration selected for the enzyme assay (**B**) and V_o vs substrate concentration (0.015 (Km^{app}) ≤ [S] ≤ 0.9 (Km^{app})), adjusted to a Michaelis-Menten model by non-linear regression method (**C**). Each point is the mean of three independent experiments. The data were fitted to a substrate inhibition model using statistical analysis in GraphPad Prism 5 software. Comparison of fits between Michaelis-Menten: null hypothesis, and substrate inhibition: alternative hypothesis. For a P<0.0001, the conclusion (α = 0.05) was: reject null hypothesis, and the preferred model was substrate inhibition. The outlier point is shown in red.

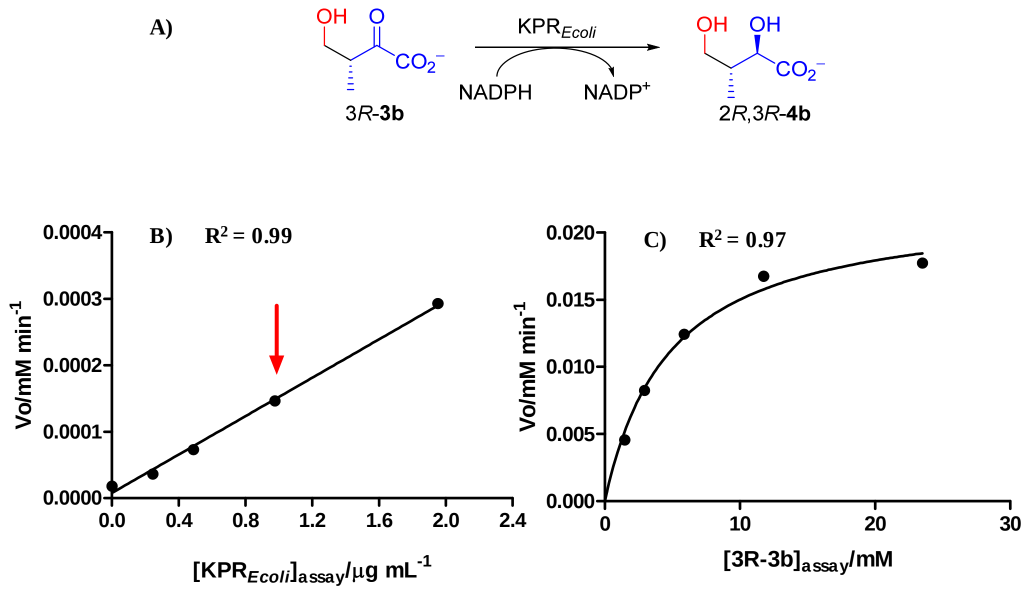

Figure S18. Reduction of 3*R*-**3b** to 2*R*,3*R*-**4b** catalyzed by KPR_{Ecoli}. (**A**). Initial reaction rate (V_o) vs the enzyme concentration. The arrow shows the enzyme concentration selected for the enzyme assay (**B**) and V_o vs substrate concentration (0.2 (Km^{app}) ≤ [S] ≤ 5(Km^{app})), adjusted to a Michaelis-Menten model by non-linear regression method (**C**). Each point is the mean of three independent experiments.

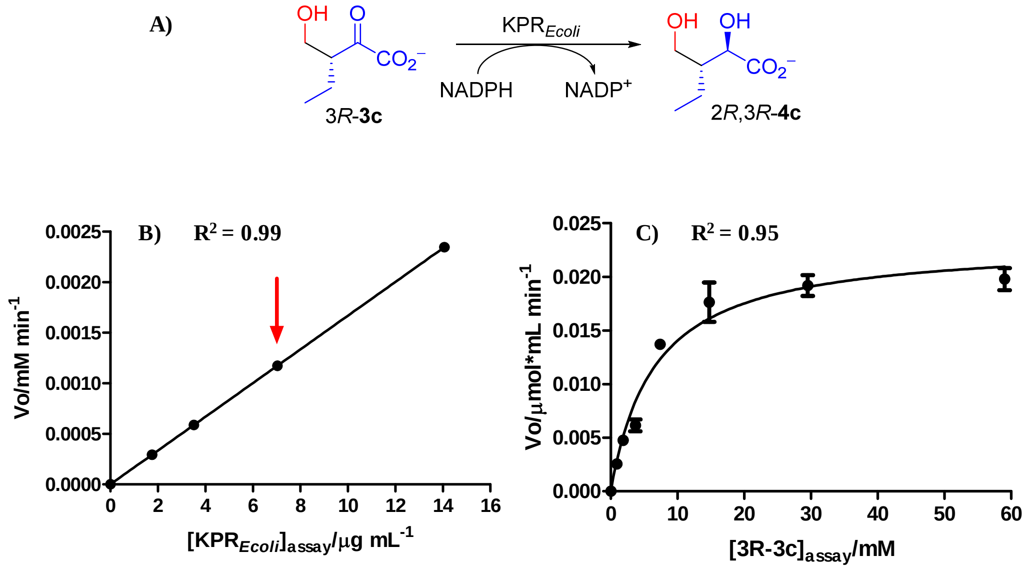

Figure S19. Reduction of 3*R*-**3c** to 2*R*,3*R*-**4c** catalyzed by KPR_{Ecoli}. (**A**). Initial reaction rate (V_o) vs the enzyme concentration. The arrow shows the enzyme concentration selected for the enzyme assay (**B**) and V_o vs substrate concentration (0.14 (Km^{app}) ≤ [S] ≤ 8.6 (Km^{app})), adjusted to a Michaelis-Menten model by non-linear regression method (**C**). Each point is the mean of three independent experiments.

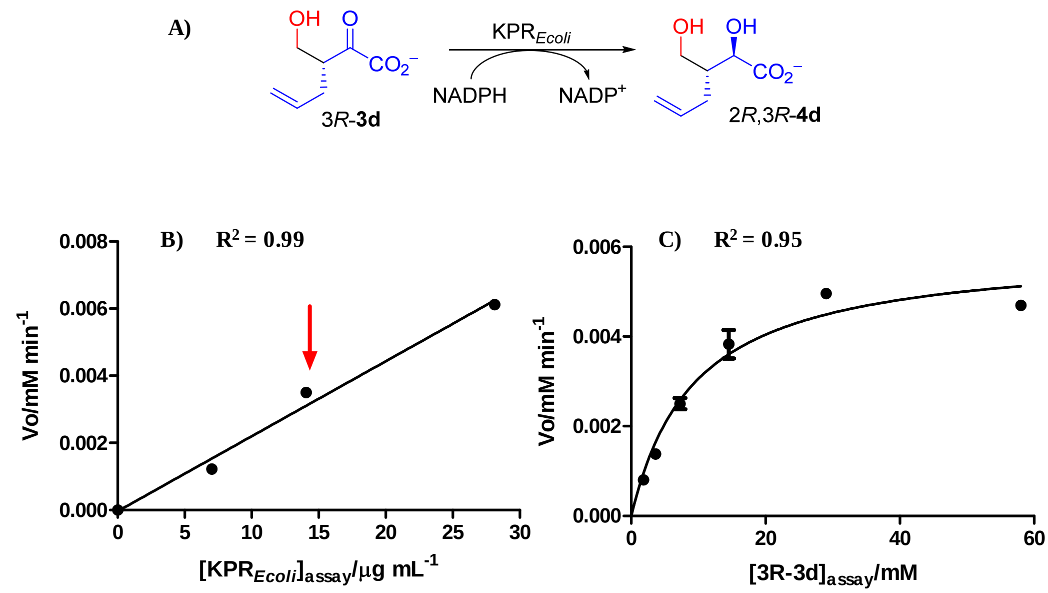

Figure S20 Reduction of 3*R*-**3d** to 2*R*,3*R*-**4d** catalyzed by KPR_{Ecoli}. (**A**). Initial reaction rate (V_o) vs the enzyme concentration. The arrow shows the enzyme concentration selected for the enzyme assay (**B**) and V_o vs substrate concentration (0.11 ($K\text{m}^{\text{app}}$) ≤ [S] ≤ 6.1 ($K\text{m}^{\text{app}}$)), adjusted to a Michaelis-Menten model by non-linear regression method (**C**). Each point is the mean of three independent experiments.

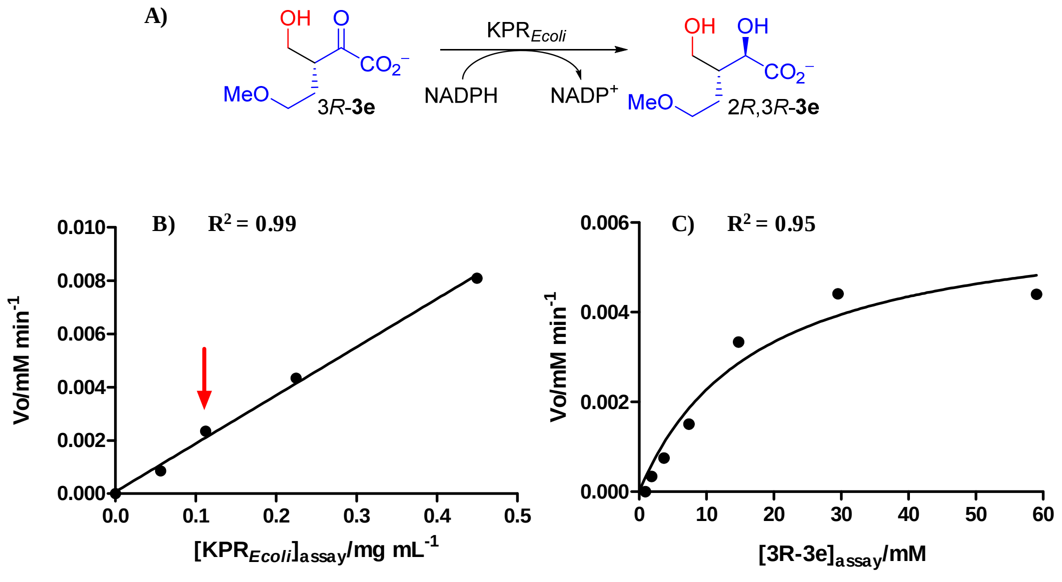

Figure S21. Reduction of 3*R*-**3e** to 2*R*,3*R*-**4e** catalyzed by KPR_{Ecoli}. (**A**). Initial reaction rate (V_o) vs the enzyme concentration. The arrow shows the enzyme concentration selected for the enzyme assay (**B**) and V_o vs substrate concentration (0.05 ($K\text{m}^{\text{app}}$) ≤ [S] ≤ 3.3 ($K\text{m}^{\text{app}}$)), adjusted to a Michaelis-Menten model by non-linear regression method (**C**). Each point is the mean of three independent experiments.

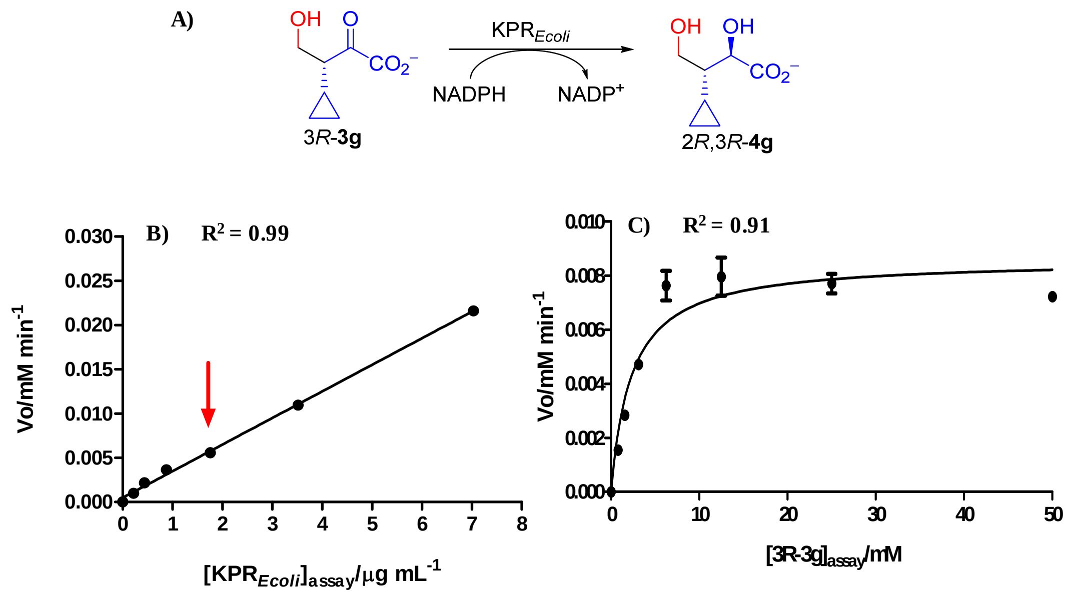

Figure S22. Reduction of 3*R*-**3g** to 2*R*,3*R*-**4g** catalyzed by KPR$_{Ecoli}$. (**A**). Initial reaction rate (V_o) vs the enzyme concentration. The arrow shows the enzyme concentration selected for the enzyme assay (**B**) and V_o vs substrate concentration (0.4 (Km^{app}) ≤ [S] ≤ 21.7 (Km^{app})), adjusted to a Michaelis-Menten model by non-linear regression method (**C**). Each point is the mean of three independent experiments.

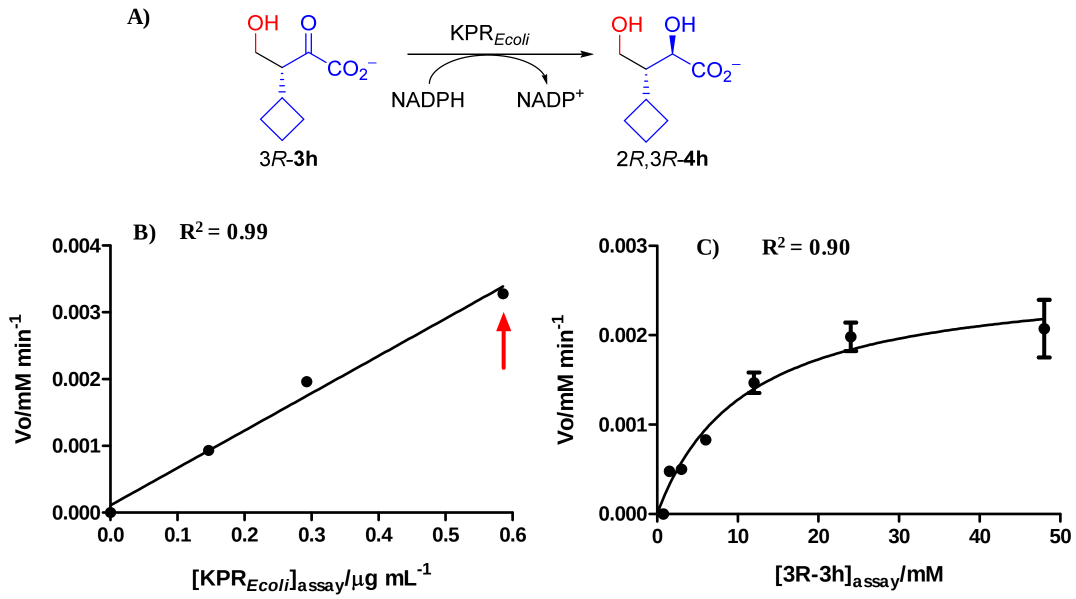

Figure S23. Reduction of 3*R*-**3h** to 2*R*,3*R*-**4h** catalyzed by KPR$_{Ecoli}$. (**A**). Initial reaction rate (V_o) vs the enzyme concentration. The arrow shows the enzyme concentration selected for the enzyme assay (**B**) and V_o vs substrate concentration (0.09(Km^{app}) ≤ [S] ≤ 4.4 (Km^{app})), adjusted to a Michaelis-Menten model by non-linear regression method (**C**). Each point is the mean of three independent experiments.

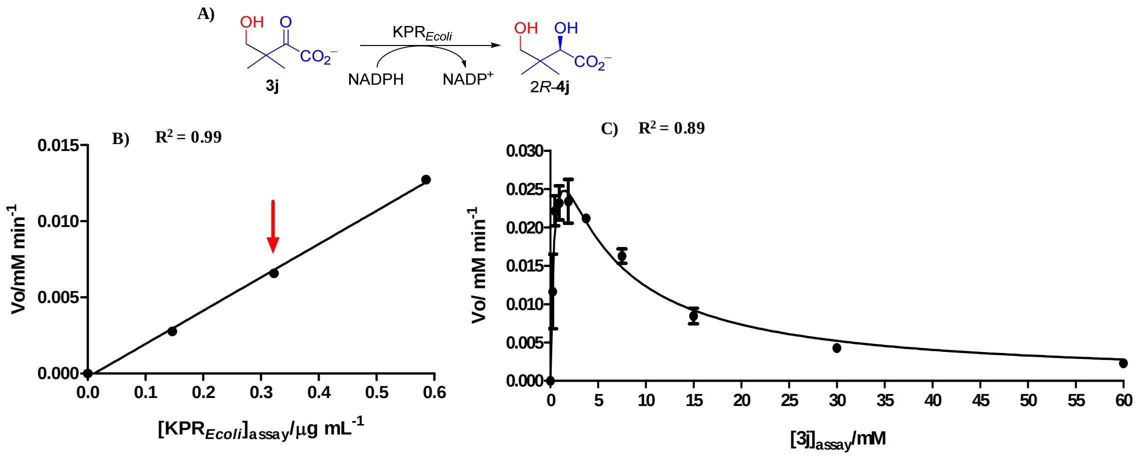

Figure S24. Reduction of **3j** to 2*R*-**4j** catalyzed by KPR$_{Ecoli}$. (**A**). Initial reaction rate (V_o) vs the enzyme concentration. The arrow shows the enzyme concentration selected for the enzyme assay (**B**) and V_o vs substrate concentration (2.5(Km^{app}) ≤ [S] ≤ 150(Km^{app})), adjusted to a Michaelis-Menten model by non-linear regression method (**C**). Each point is the mean of three independent experiments.

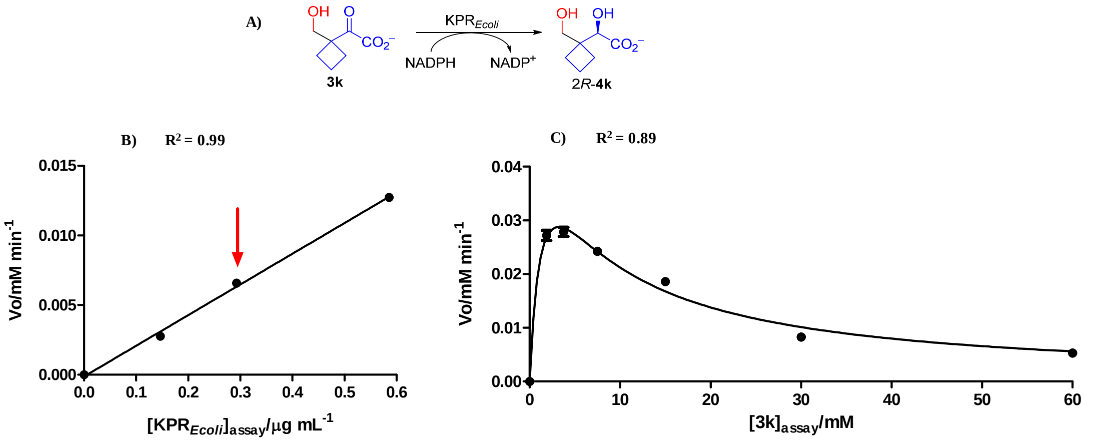

Figure S25. Reduction of **3k** to 2*R*-**4k** catalyzed by KPR$_{Ecoli}$. (**A**). Initial reaction rate (V_o) vs the enzyme concentration. The arrow shows the enzyme concentration selected for the enzyme assay (**B**) and V_o vs substrate concentration (0.7(Km^{app}) ≤ [S] ≤ 40 (Km^{app})), adjusted to a Michaelis-Menten model by non-linear regression method (**C**). Each point is the mean of three independent experiments.

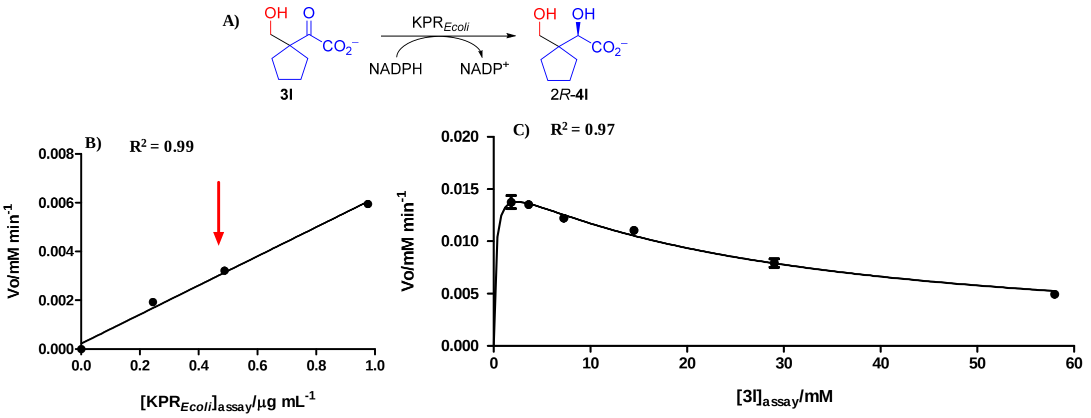

Figure S26. Reduction of **3l** to 2*R*-**4l** catalyzed by KPR$_{Ecoli}$. (**A**). Initial reaction rate (V_o) vs the enzyme concentration. The arrow shows the enzyme concentration selected for the enzyme assay (**B**) and V_o vs substrate concentration (5(Km^{app}) ≤ [S] ≤ 290 (Km^{app})), adjusted to a Michaelis-Menten model by non-linear regression method (**C**). Each point is the mean of three independent experiments.

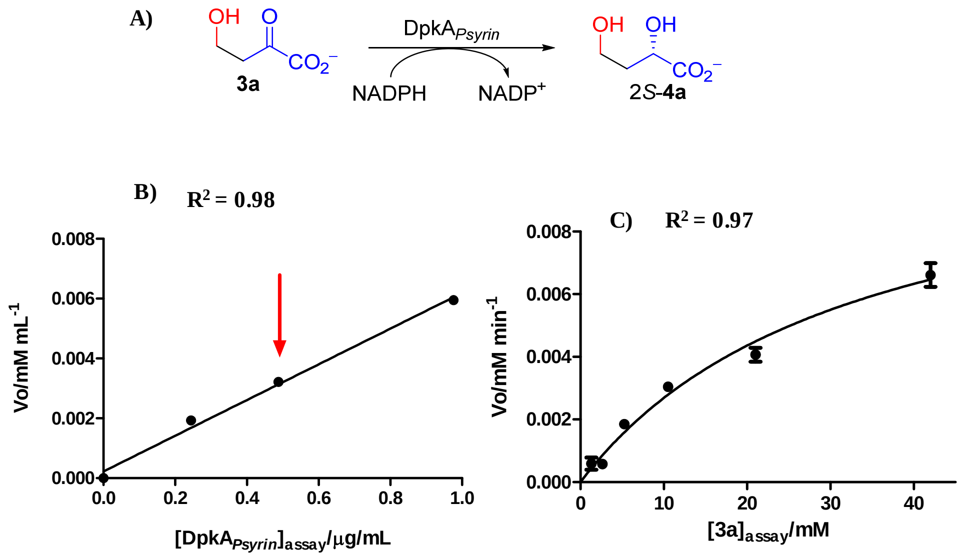

Figure S27. Reduction of **3a** to 2*S*-**4a** catalyzed by DpkA$_{Psyrin}$. (**A**). Initial reaction rate (V_o) vs the enzyme concentration. The arrow shows the enzyme concentration selected for the enzyme assay (**B**) and V_o vs substrate concentration (0.03(Km^{app}) ≤ [S] ≤ 1.3 (Km^{app})), adjusted to a Michaelis-Menten model by non-linear regression method (**C**). Each point is the mean of three independent experiments.

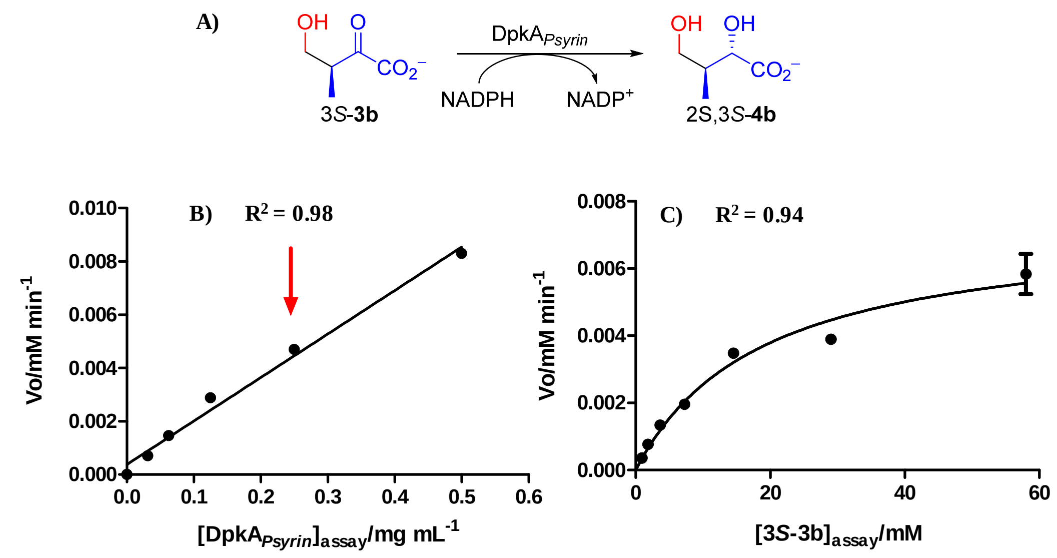

Figure S28. Reduction of 3*S*-**3b** to 2*S*,3S-**4b** catalyzed by DpkA*Psyrin*. (**A**). Initial reaction rate (V_o) vs the enzyme concentration. The arrow shows the enzyme concentration selected for the enzyme assay (**B**) and V_o vs substrate concentration (0.05(*K*m^{app}) ≤ [S] ≤ 3 (*K*m^{app})), adjusted to a Michaelis-Menten model by non-linear regression method (**C**). Each point is the mean of three independent experiments.

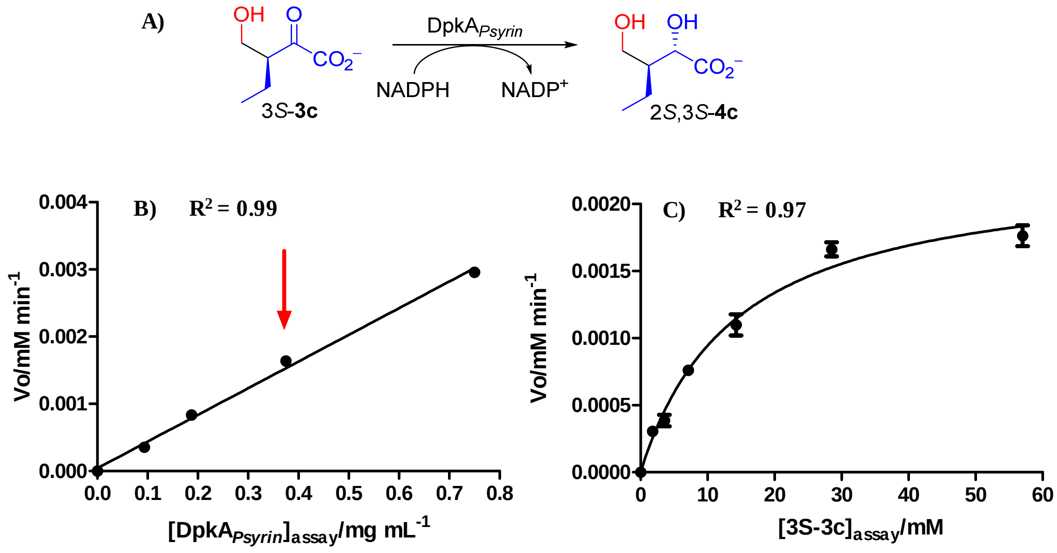

Figure S29. Reduction of 3*S*-**3c** to 2*S*,3*S*-**4c** catalyzed by DpkA*Psyrin*. (**A**). Initial reaction rate (V_o) vs the enzyme concentration. The arrow shows the enzyme concentration selected for the enzyme assay (**B**) and V_o vs substrate concentration (0.07(*K*m^{app}) ≤ [S] ≤ 4.1 (*K*m^{app})), adjusted to a Michaelis-Menten model by non-linear regression method (**C**). Each point is the mean of three independent experiments.

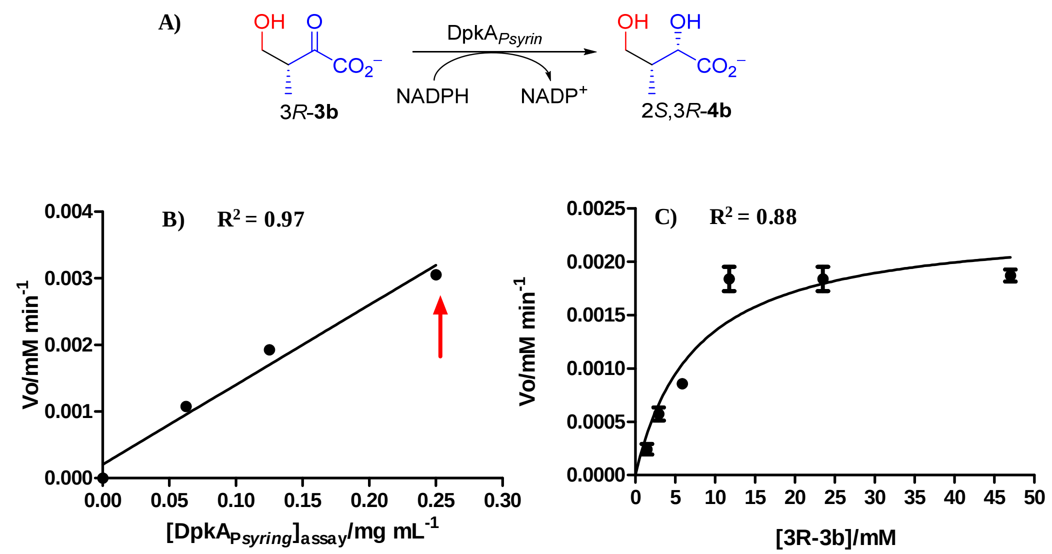

Figure S30. Reduction of 3*R*-**3b** to 2*S*,3*R*-**4b** catalyzed by DpkA$_{Psyrin}$. (**A**). Initial reaction rate (V_o) vs the enzyme concentration. The arrow shows the enzyme concentration selected for the enzyme assay (**B**) and V_o vs substrate concentration (0.13(Km^{app}) ≤ [S] ≤ 6.3 (Km^{app})), adjusted to a Michaelis-Menten model by non-linear regression method (**C**). Each point is the mean of three independent experiments.

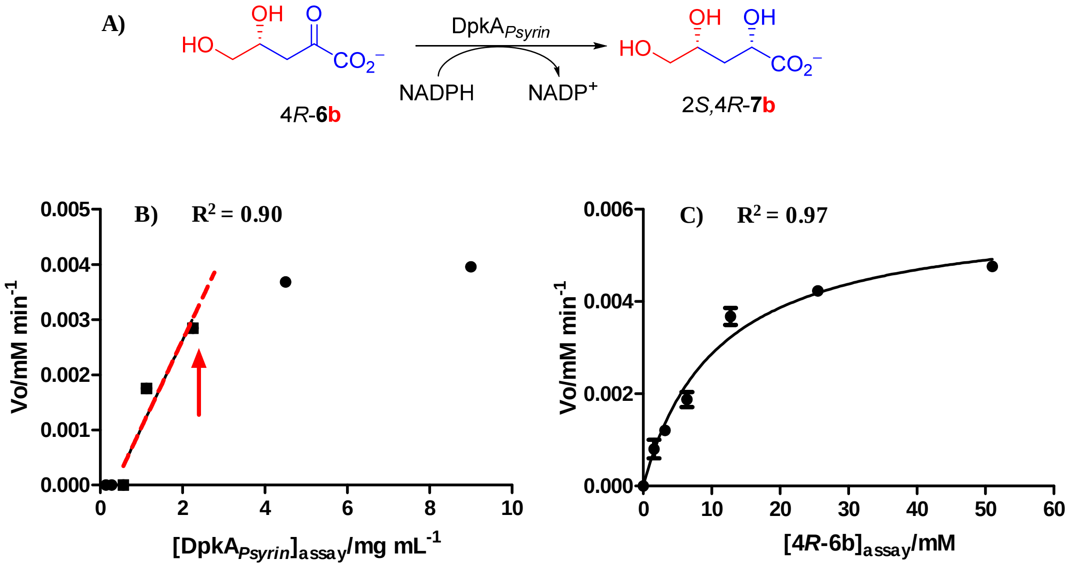

Figure S31. Reduction of 4*R*-**6b** to 2*S*,4*R*-**7b** catalyzed by DpkA$_{Psyrin}$. (**A**). Initial reaction rate (V_o) vs the enzyme concentration. The arrow shows the enzyme concentration selected for the enzyme assay (**B**) and V_o vs substrate concentration (0.09(Km^{app}) ≤ [S] ≤ 4.6 (Km^{app})), adjusted to a Michaelis-Menten model by non-linear regression method (**C**). Each point is the mean of three independent experiments.

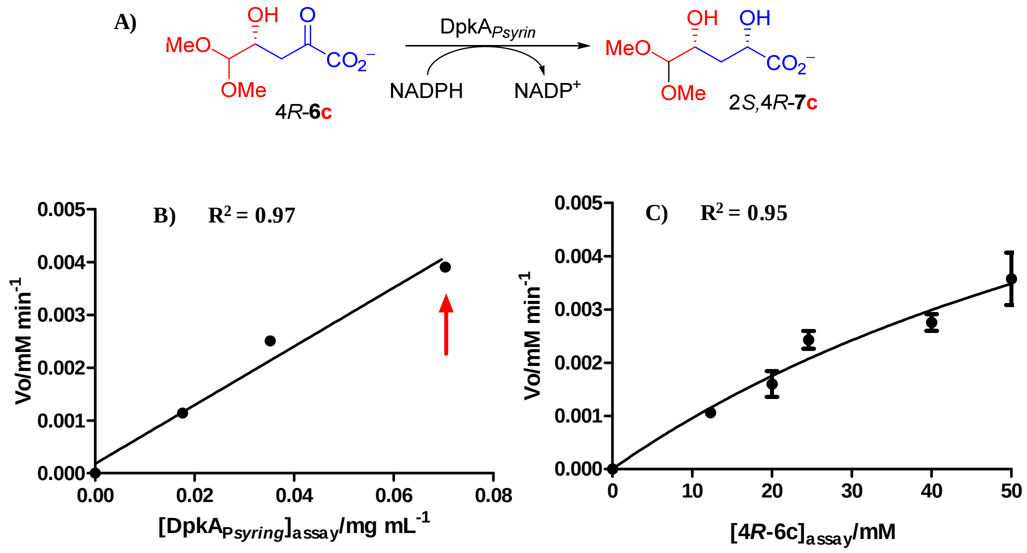

Figure S32. Reduction of 4*R*-**6c** to 2*S*,4*R*-**7c** catalyzed by DpkA$_{Psyrin}$. (**A**). Initial reaction rate (V_o) vs the enzyme concentration. The arrow shows the enzyme concentration selected for the enzyme assay (**B**) and V_o vs substrate concentration (0.01(Km^{est}) ≤ [S] ≤ 0.6 (Km^{est})), adjusted to a Michaelis-Menten model by non-linear regression method (**C**). Each point is the mean of three independent experiments.

X-Ray structures

Suitable single crystals for X-ray structural analysis of *R*-**5l** were obtained at room temperature. Compound *R*-**5l** (40 mg) was dissolved in Hexane:methanol 3:1 (v/v) (5 mL). Crystals were obtained by evaporation in glass vials (6 mL, 3.5 cm, Ø 1.4 cm) after 48 h at 25 °C. The X-ray diffraction analysis on the *R*-**5l** indicates that KPR$_{Ecoli}$ rendered *R*-**5l** having *R* configuration (**Figure S33**).

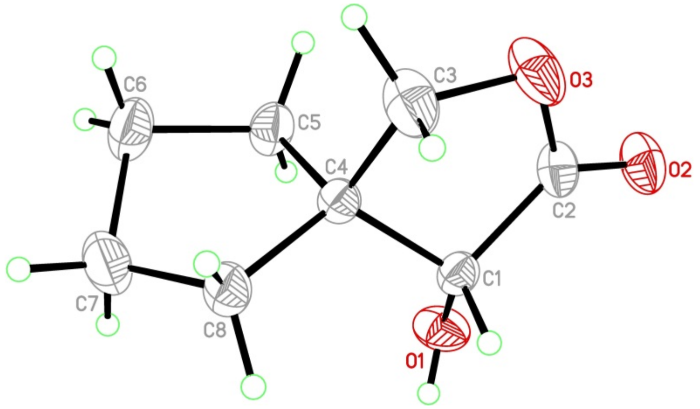

Figure S33. X-ray structure of *R*-**5l**. ORTEP-type plot displaying one molecule with 50% probability ellipsoids. The data can be obtained free of charge from The Cambridge Crystallographic Data Centre via www.ccdc.cam.ac.uk/data_request/cif

Data were collected on a STOE IPDS II two-circle diffractometer with a Genix Microfocus tube with mirror optics using MoKα radiation (λ = 0.71073 Å). The data were scaled using the frame scaling procedure in the X-Area program system (Software X-Area - STOE & Cie GmbH. https://www.stoe.com/product/software-x-area). The structures was solved by direct methods using the program SHELXS and refined against F^2 with full-matrix least-squares techniques using the program SHELXL[17] (Table S5 and S6).

Table S5. Crystal data and structure refinement for *R*-**5l**.

Identification code	*R*-**5l**	
CCDC number	2208404	
Empirical formula	$C_8H_{12}O_3$	
Formula weight	156.18	
Temperature	173(2) K	
Wavelength	0.71073 Å	
Crystal system	Orthorhombic	
Space group	P 21 21 21	
Unit cell dimensions	a = 5.9887(4) Å	α = 90°
	b = 9.1619(6) Å	β = 90°
	c = 14.1820(7) Å	γ = 90°
Volume	778.14(8) $Å^3$	
Z	4	
Density (calculated)	1.333 Mg/m^3	
Absorption coefficient	0.101 mm^{-1}	
F(000)	336	
Crystal color, shape	colorless needle	
Crystal size	0.280 x 0.120 x 0.110 mm^3	
Theta range for data collection	3.633 to 27.182°.	
Index ranges	–7<=h<=7, –11<=k<=11, –18<=l<=17	
Reflections collected	10506	
Independent reflections	1713 [R(int) = 0.0447]	
Completeness to theta = 25.000°	99.3 %	
Absorption correction	Semi-empirical from equivalents	
Max. and min. transmission	1.000 and 0.310	
Refinement method	Full-matrix least-squares on F^2	

Data / restraints / parameters	1713 / 0 / 104	
Goodness-of-fit on F^2	1.177	
Final R indices [I>2sigma(I)]	R1 = 0.0433, wR2 = 0.1068	
R indices (all data)	R1 = 0.0443, wR2 = 0.1077	
Absolute structure parameter	– 0.3(9)	
Extinction coefficient	n/a	
Largest diff. peak and hole	0.209 and -0.147 e.Å^{-3}	

Table S6 Atomic coordinates (x 10^4) and equivalent isotropic displacement parameters (Å^{2}x 10^3) for *R*-**5l**. U(eq) is defined as one third of the trace of the orthogonalized U^{ij} tensor.

	x	y	z	U(eq)
O(1)	4931(4)	7397(2)	4334(1)	33(1)
O(2)	2558(3)	5162(2)	5316(1)	35(1)
O(3)	4827(4)	3606(2)	4601(2)	41(1)
C(1)	5909(4)	6032(2)	4491(2)	22(1)
C(2)	4222(4)	4952(3)	4856(2)	27(1)
C(3)	6880(5)	3694(3)	4029(2)	37(1)
C(4)	6871(4)	5239(2)	3630(2)	20(1)
C(5)	5373(4)	5368(3)	2748(2)	30(1)
C(6)	6993(5)	5350(4)	1924(2)	37(1)
C(7)	8943(5)	6236(4)	2285(2)	40(1)
C(8)	9213(4)	5740(3)	3306(2)	28(1)

In addition, Cu K-α radiation (λ = 1.5406 Å) was employed at room temperature to assess the absolute stereochemistry of *R*-**5l**. The Flack-x-parameter was determined to be - 0.04(16) (Table S7).

Table S7. Crystal data and structure refinement for *R*-**5l** with Cu K-α radiation.

Identification code	*R*-**5l** c13_cu	
CCDC number	2208405	
Empirical formula	$C_8H_{12}O_3$	
Formula weight	156.18	
Temperature	293(2) K	
Wavelength	1.54178 Å	
Crystal system	Orthorhombic	

Space group	P 21 21 21	
Unit cell dimensions	a = 6.0593(5) Å	α = 90°.
	b = 9.2089(7) Å	β = 90°.
	c = 14.3338(11) Å	γ = 90°.
Volume	799.82(11) $Å^3$	
Z	4	
Density (calculated)	1.297 Mg/m^3	
Absorption coefficient	0.820 mm^{-1}	
F(000)	336	
Crystal colour, shape	colorless needle	
Crystal size	0.280 x 0.120 x 0.110 mm^3	
Theta range for data collection	5.710 to 68.675°.	
Index ranges	–7<=h<=7, –11<=k<=11, –16<=l<=16	
Reflections collected	13027	
Independent reflections	1427 [R(int) = 0.1262]	
Completeness to theta = 68.000°	98.3 %	
Absorption correction	Semi-empirical from equivalents	
Max. and min. transmission	1.000 and 0.239	
Refinement method	Full-matrix least-squares on F^2	
Data / restraints / parameters	1427 / 0 / 104	
Goodness-of-fit on F^2	1.785	
Final R indices [I>2sigma(I)]	R1 = 0.1178, wR2 = 0.3220	
R indices (all data)	R1 = 0.1419, wR2 = 0.3904	
Absolute structure parameter	-0.04(16)	
Extinction coefficient	n/a	
Largest diff. peak and hole	0.325 and -0.750 $e.Å^{-3}$	

NMR spectra.

a)

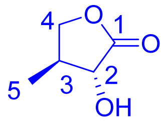

2*R*,3*S*-**5b**

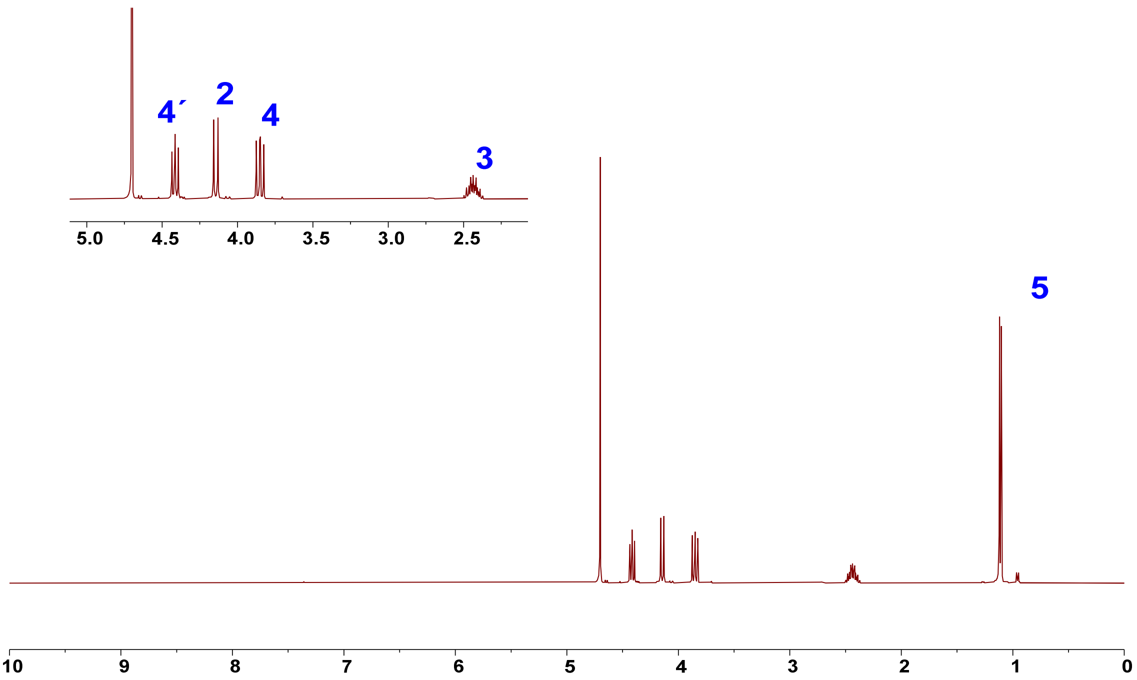

b)

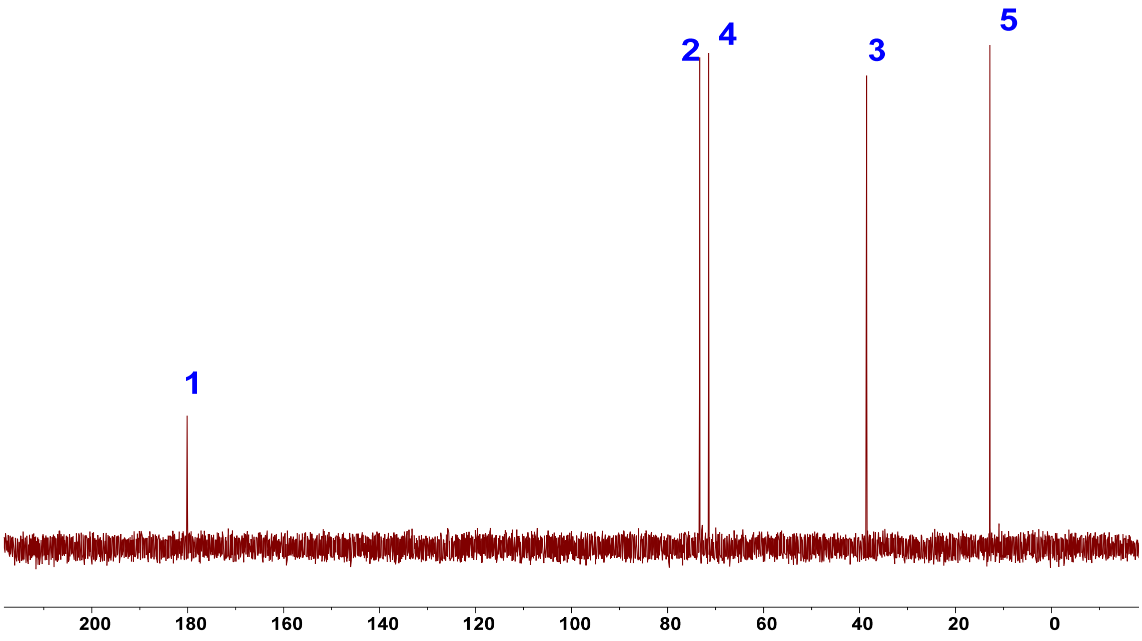

c)

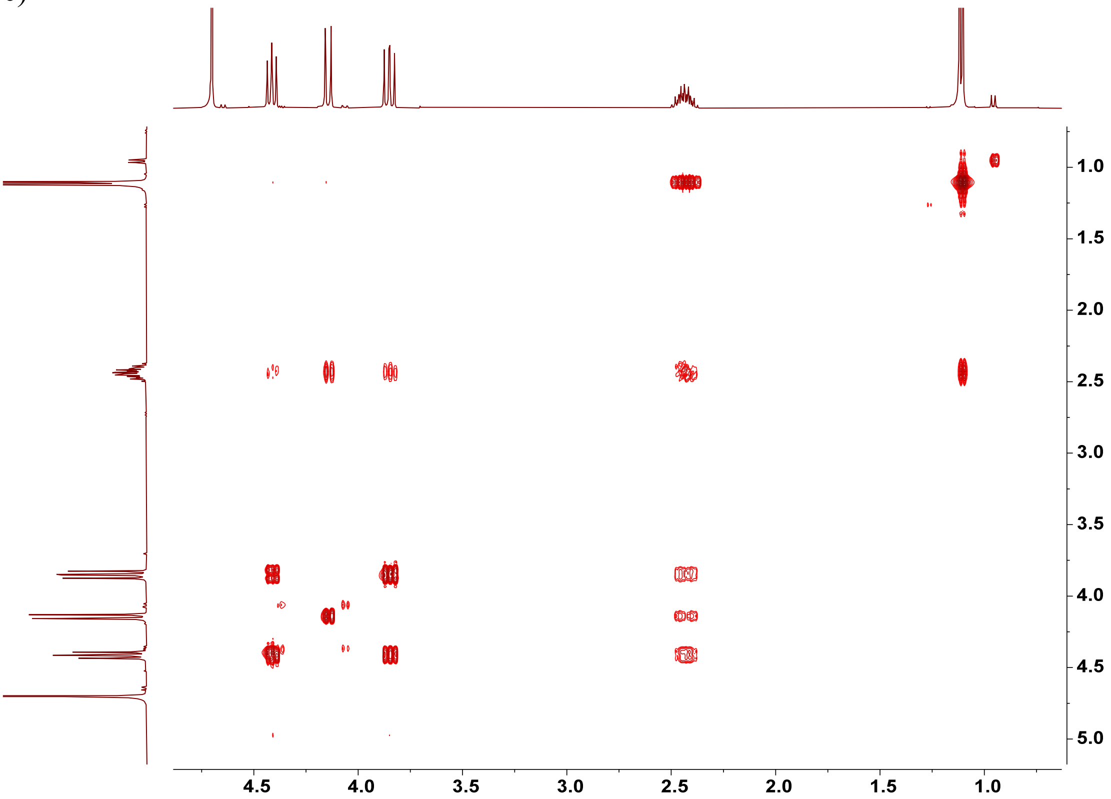
1.0
1.5
2.0
2.5
3.0
3.5
4.0
4.5
5.0
4.5
4.0
3.5
3.0
2.5
2.0
1.5
1.0

d)

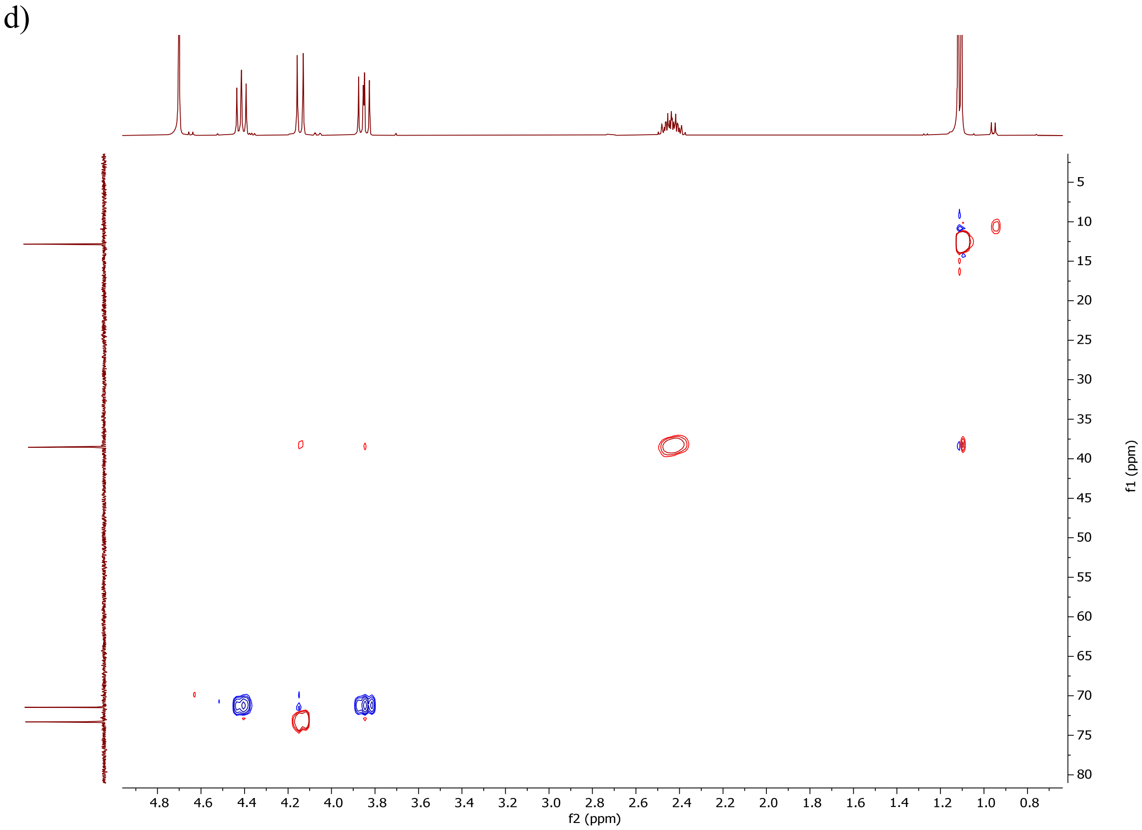
5
10
15
20
25
30
35
40
45
50
55
60
65
70
75
80
f1 (ppm)
4.8 4.6 4.4 4.2 4.0 3.8 3.6 3.4 3.2 3.0 2.8 2.6 2.4 2.2 2.0 1.8 1.6 1.4 1.2 1.0 0.8
f2 (ppm)

e)

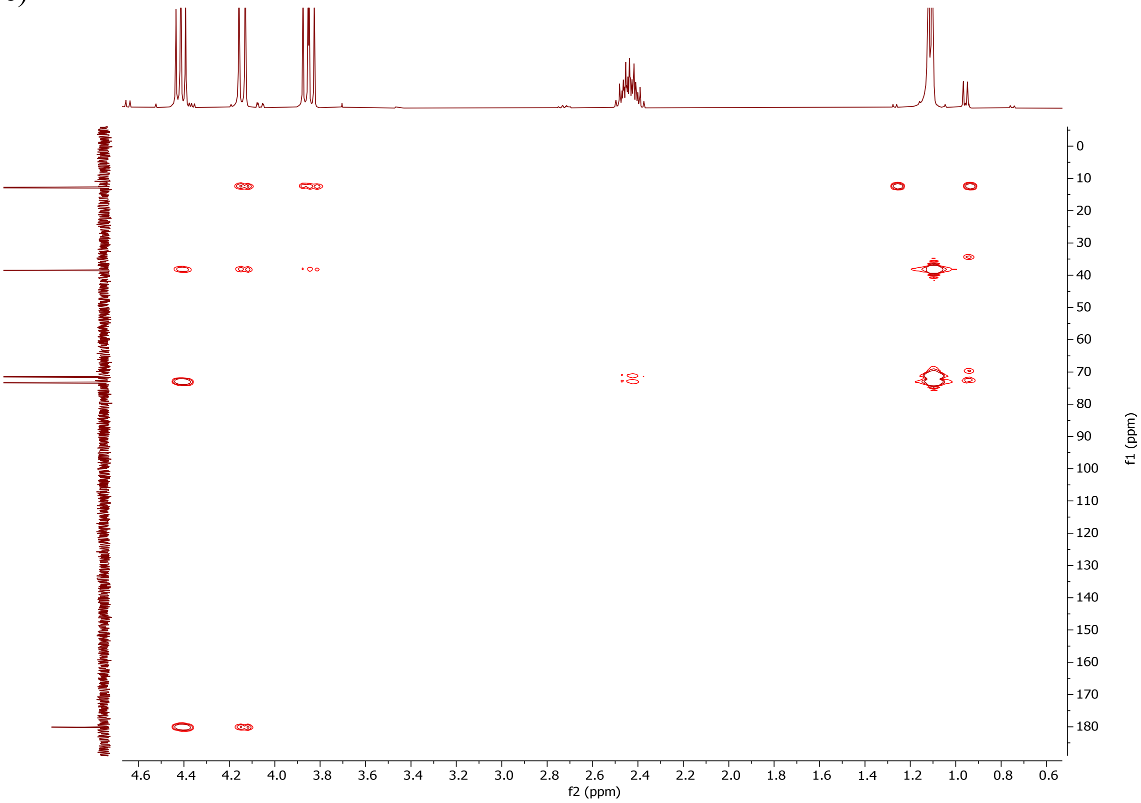

f)

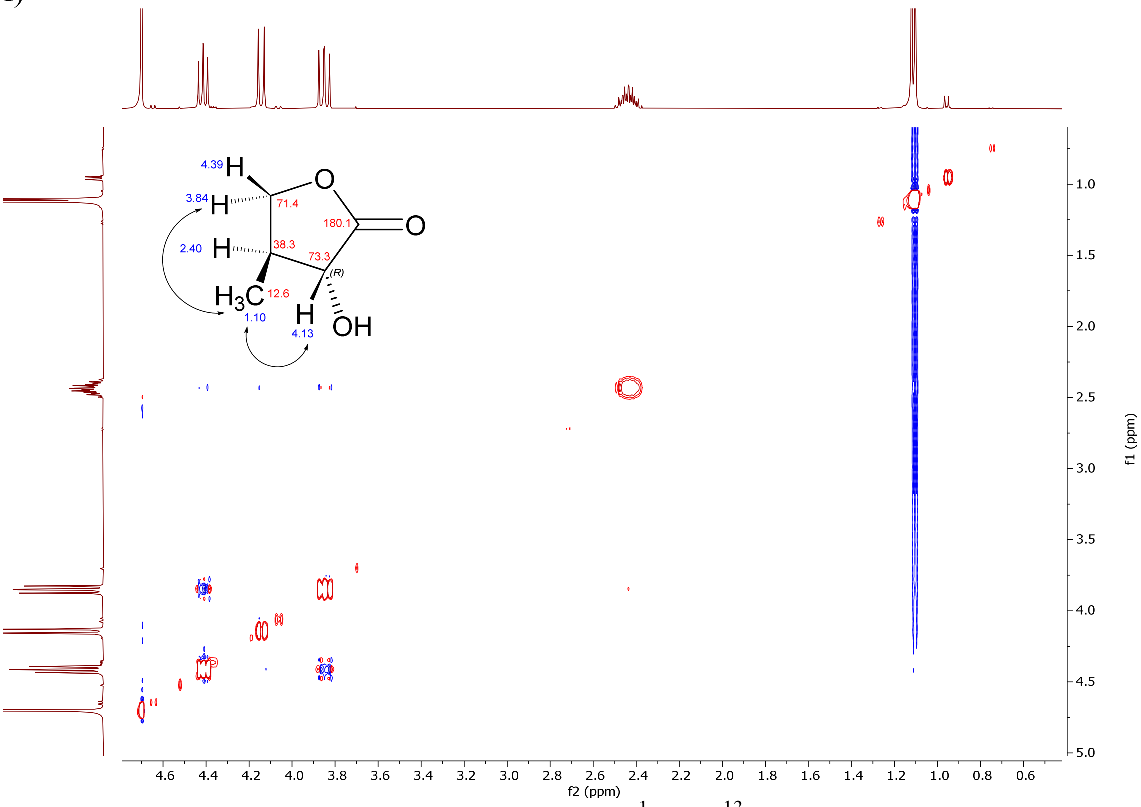

Figure S34. NMR spectra (D_2O) of 2*R*,3*S*-**5b**: a) ^{1}H, b) ^{13}C, c) COSY, d) HSQC, e) HMBC and f) NOESY.

a)

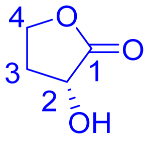
4
O
O
3
1
2
OH

R-**5a**

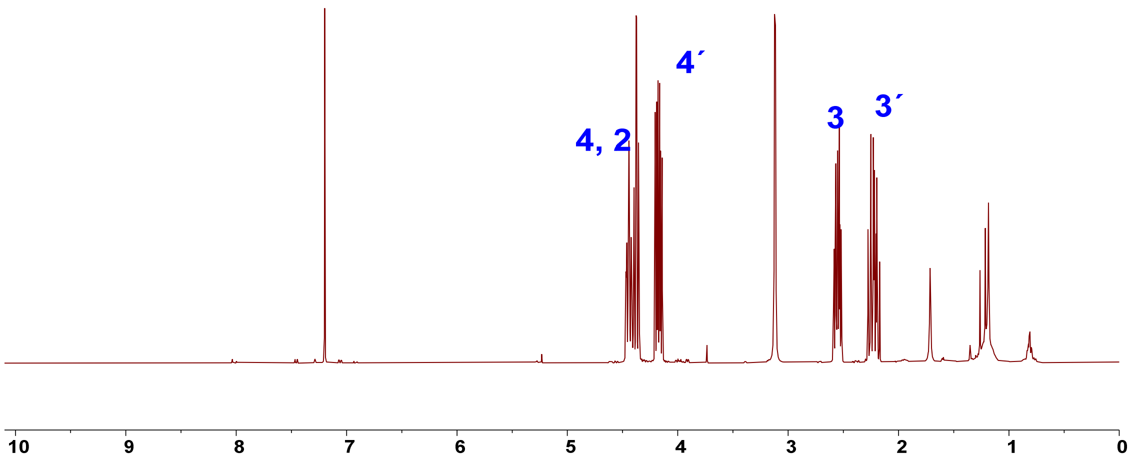
4, 2
4´
3
3´
10
9
8
7
6
5
4
3
2
1
0

b)

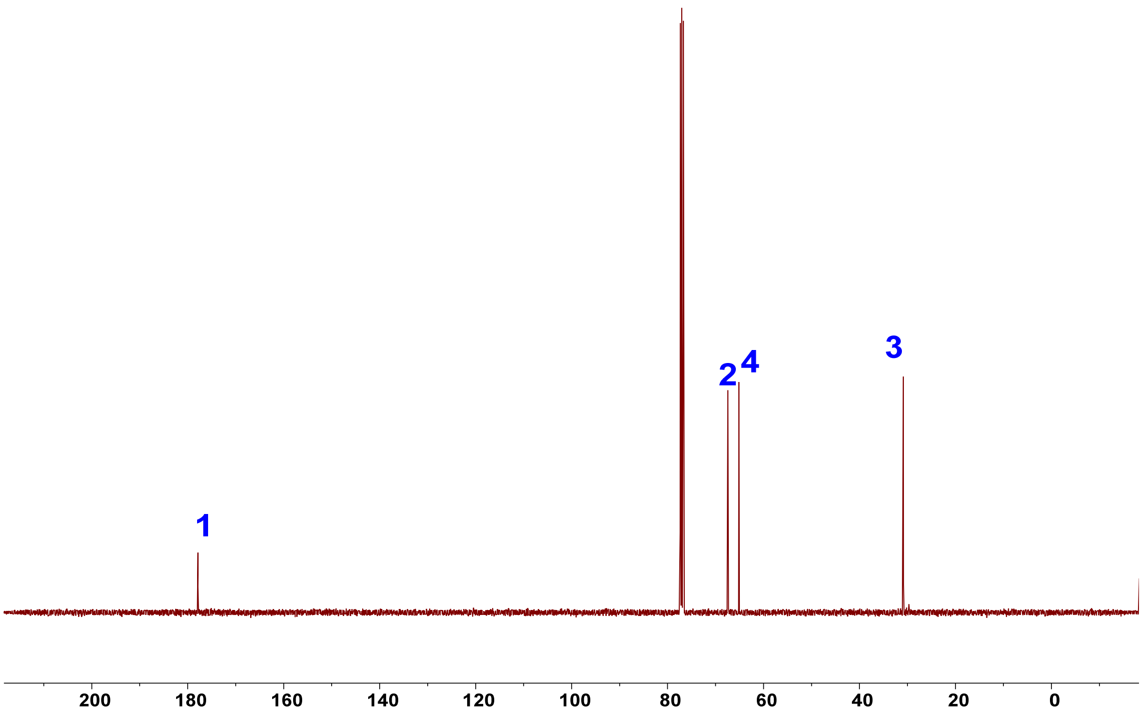
1
2
4
3
200
180
160
140
120
100
80
60
40
20
0

c)

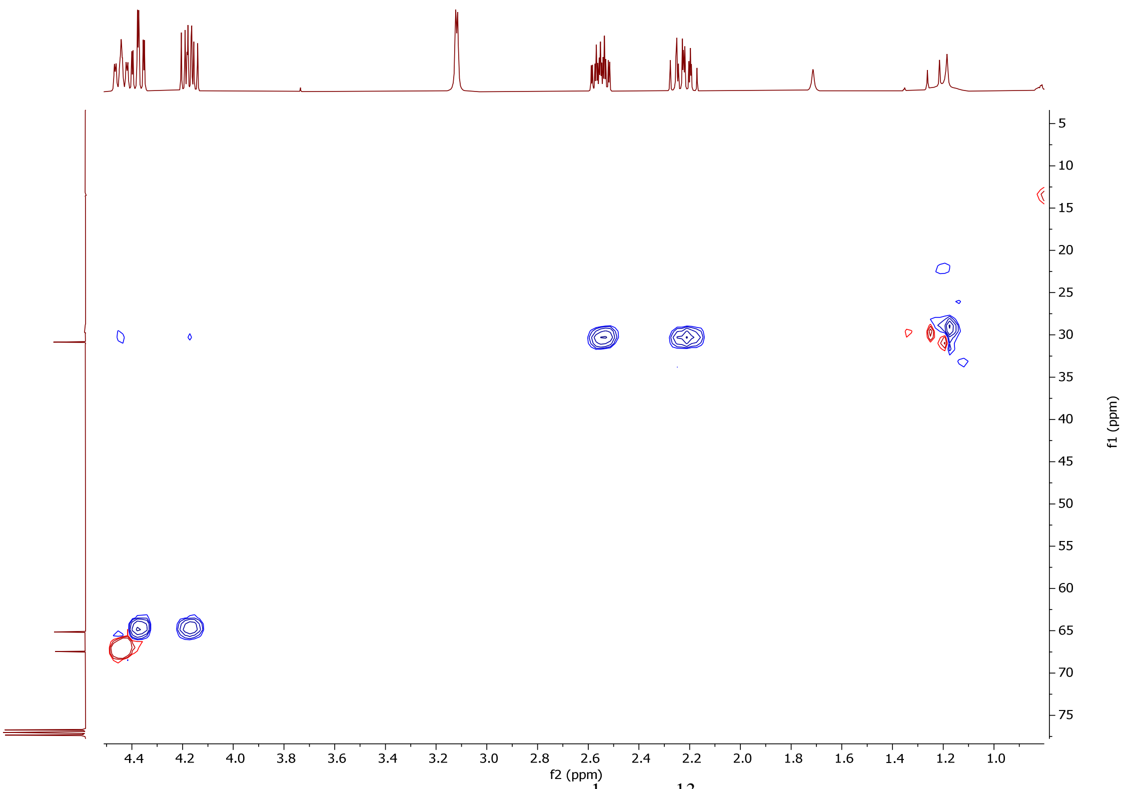

Figure S35. NMR spectra ($CDCl_3$) of *R*-**5a**: a) ^{1}H, b) ^{13}C and c) HSQC.

a)

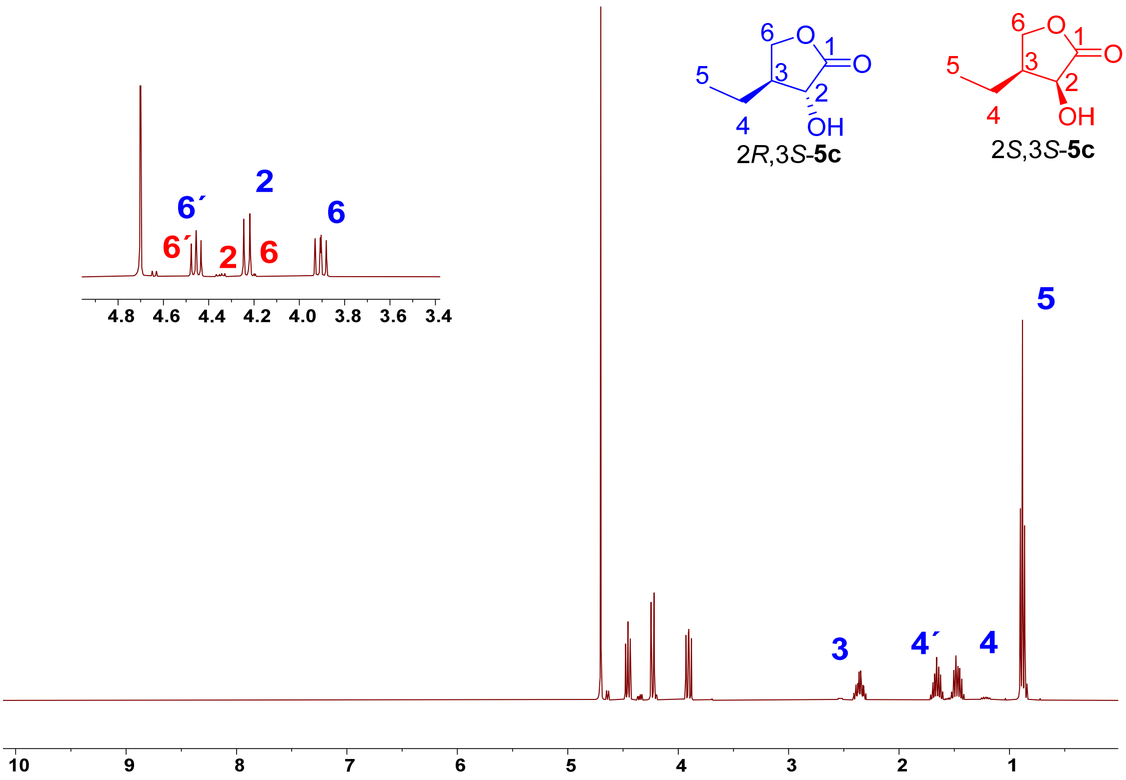

b)

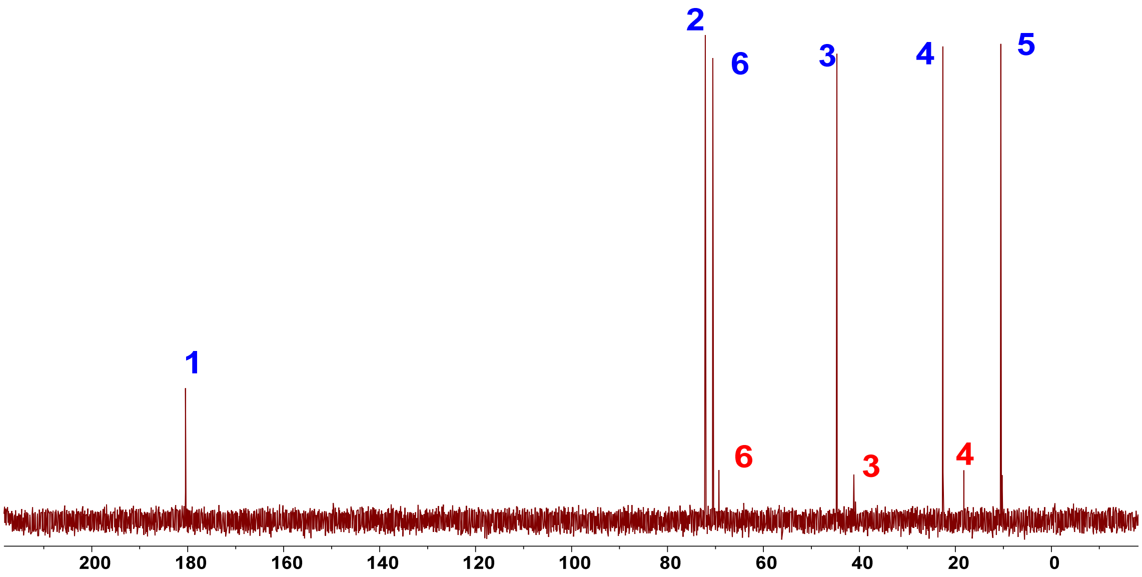

1
2
6
3
4
5
6
3
4
200
180
160
140
120
100
80
60
40
20
0

c)

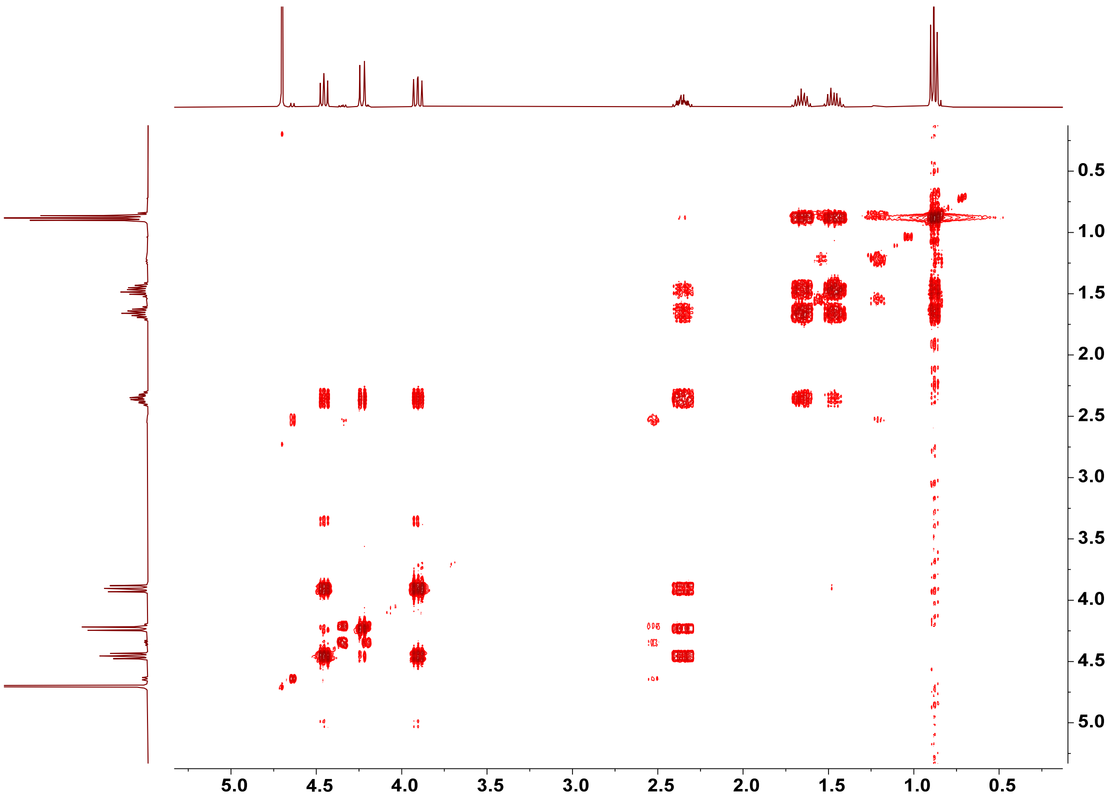

0.5
1.0
1.5
2.0
2.5
3.0
3.5
4.0
4.5
5.0
5.0
4.5
4.0
3.5
3.0
2.5
2.0
1.5
1.0
0.5

d)

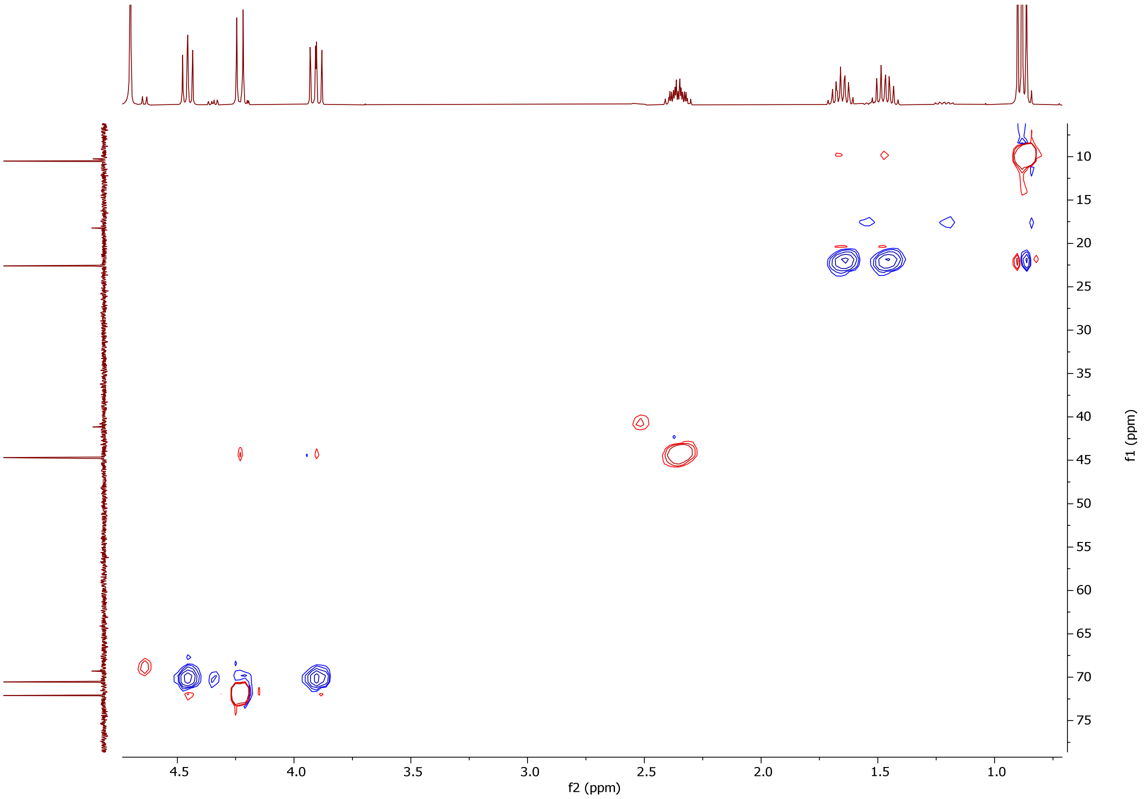
4.5
4.0
3.5
3.0
2.5
2.0
1.5
1.0
f2 (ppm)
10
15
20
25
30
35
40
45
50
55
60
65
70
75
f1 (ppm)

e)

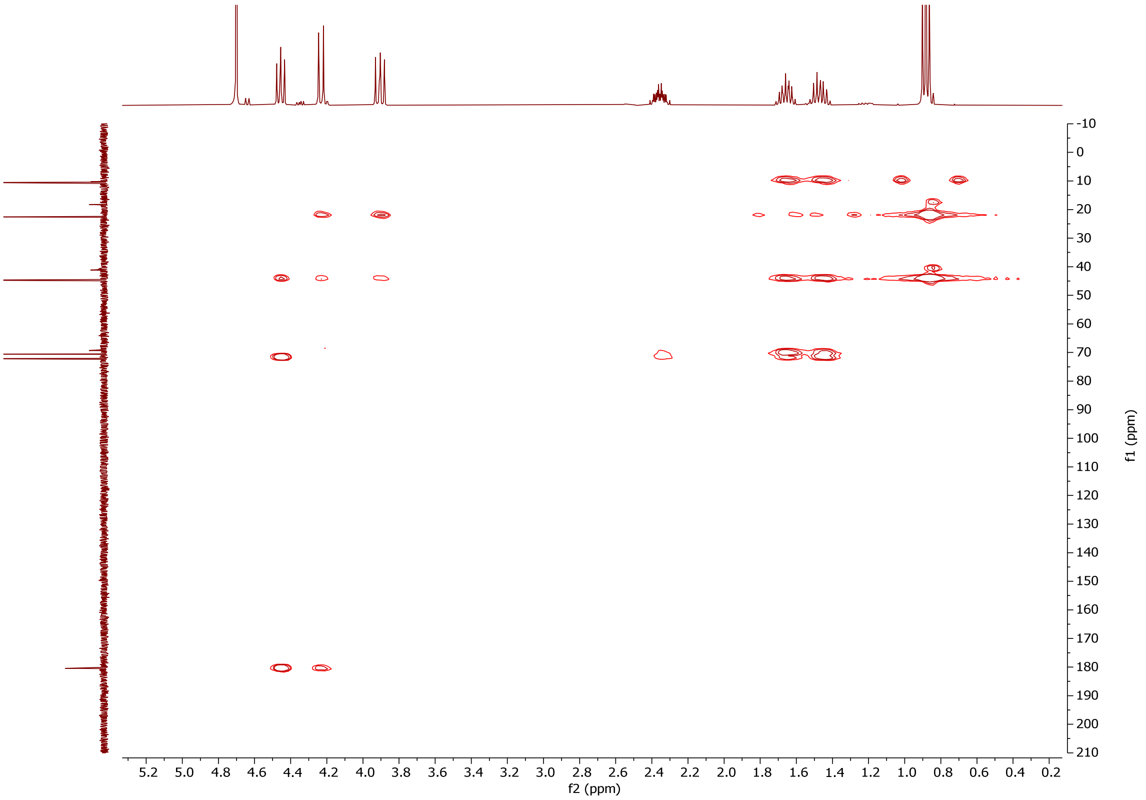
5.2
5.0
4.8
4.6
4.4
4.2
4.0
3.8
3.6
3.4
3.2
3.0
2.8
2.6
2.4
2.2
2.0
1.8
1.6
1.4
1.2
1.0
0.8
0.6
0.4
0.2
f2 (ppm)
-10
0
10
20
30
40
50
60
70
80
90
100
110
120
130
140
150
160
170
180
190
200
210
f1 (ppm)

f)

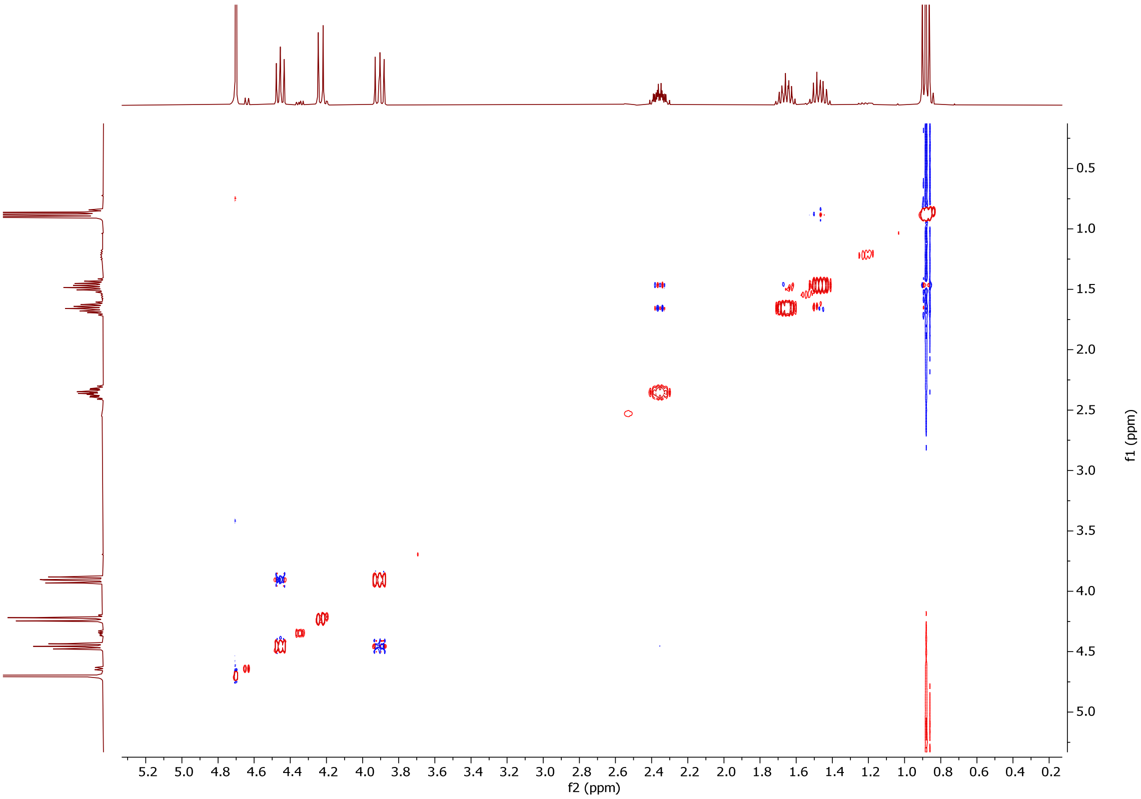

Figure S36. NMR spectra (D_2O) of 2*R*,3*S*-**5c**: a) ^{1}H, b) ^{13}C, c) COSY, d) HSQC, e) HMBC and f) NOESY.

a)

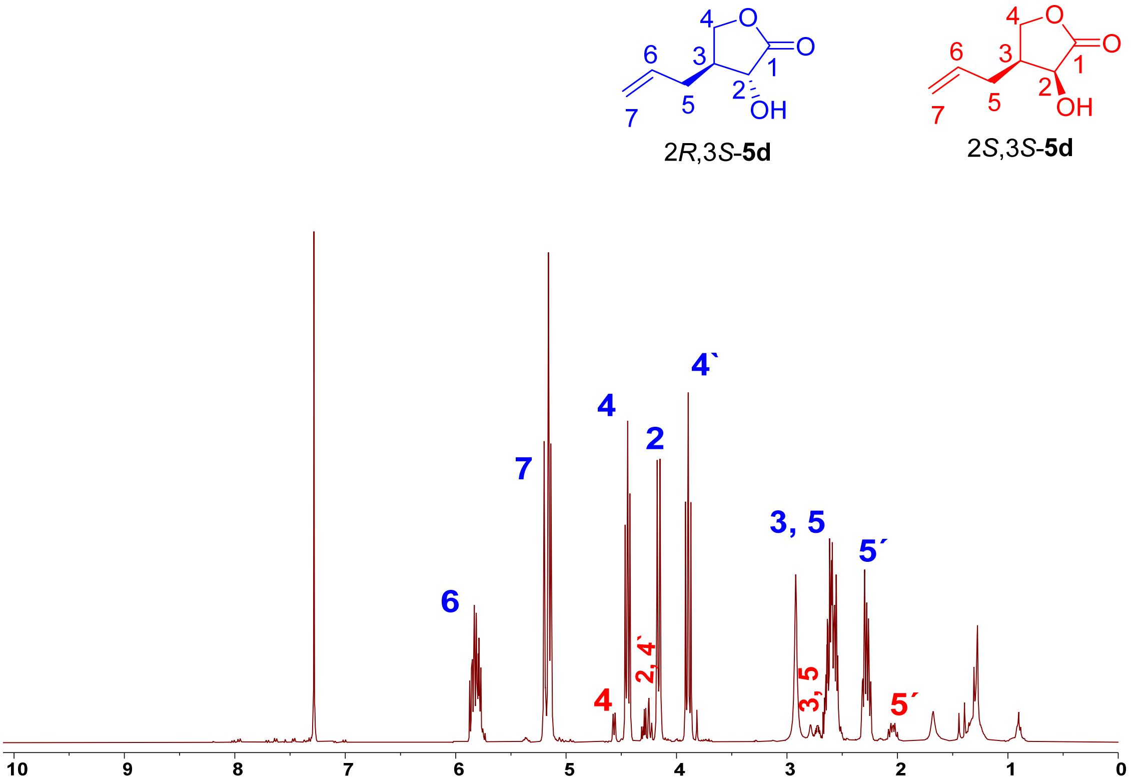

b)

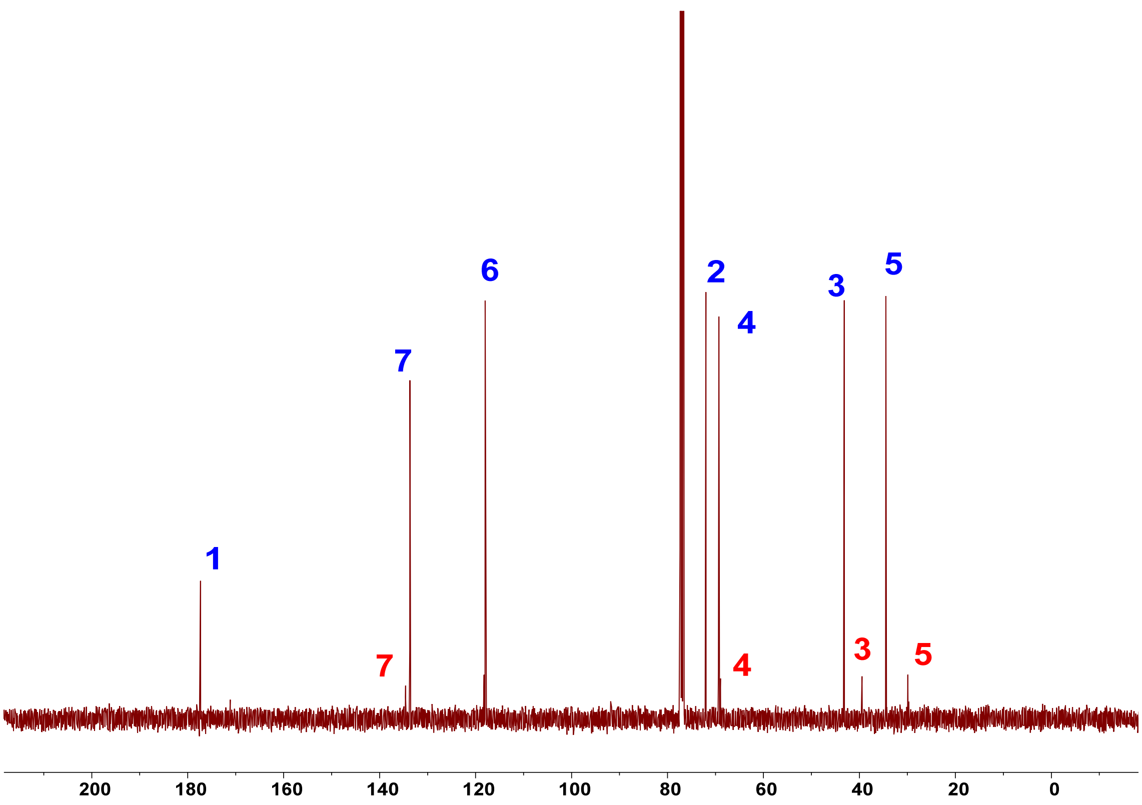

1
7
6
2
4
3
5
7
4
3
5
200
180
160
140
120
100
80
60
40
20
0

c)

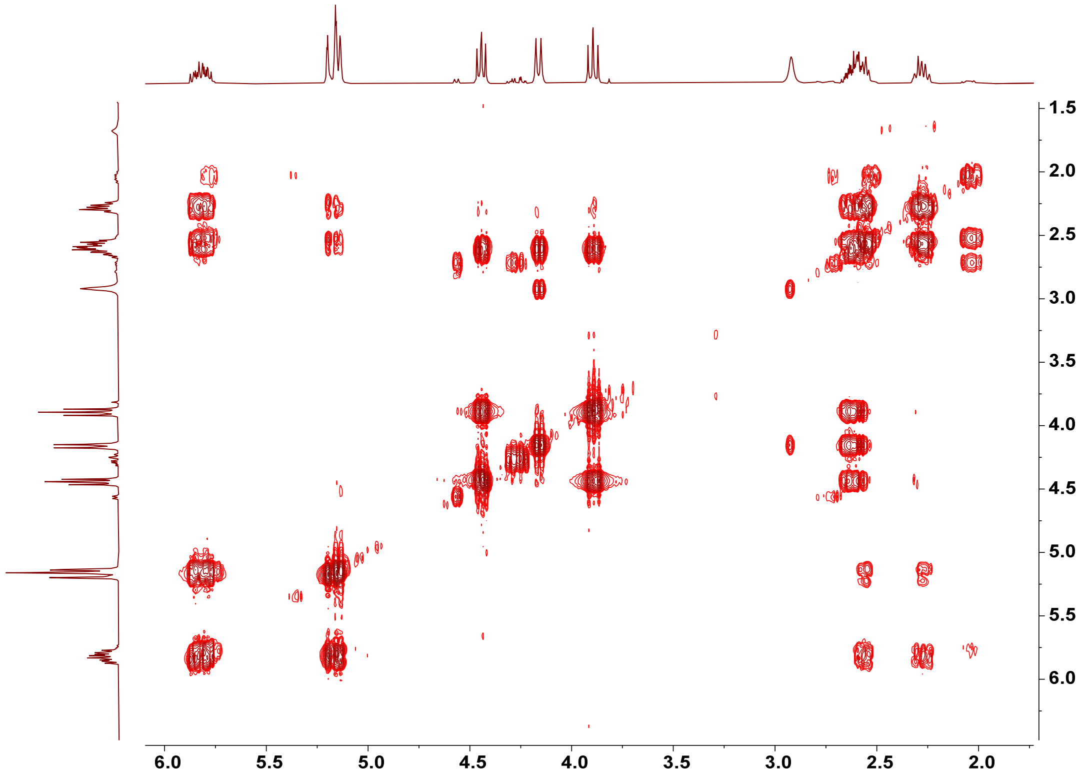

6.0
5.5
5.0
4.5
4.0
3.5
3.0
2.5
2.0
1.5

d)

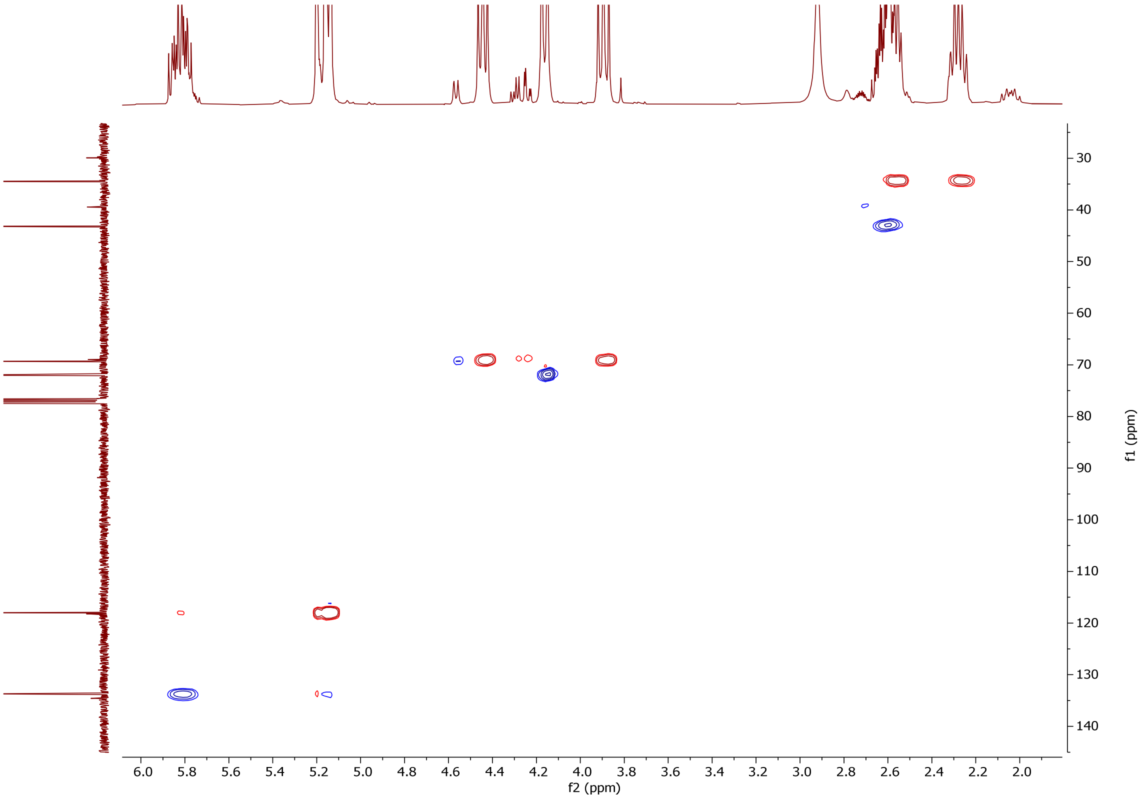

e)

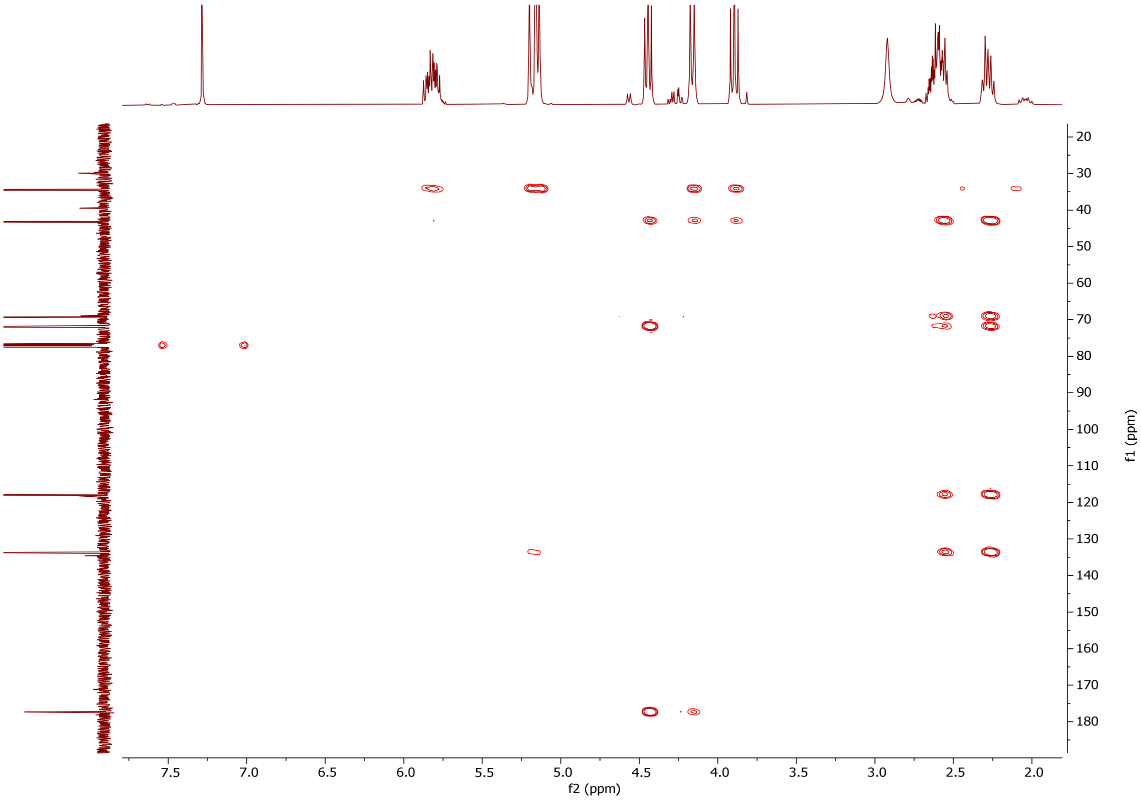

f)

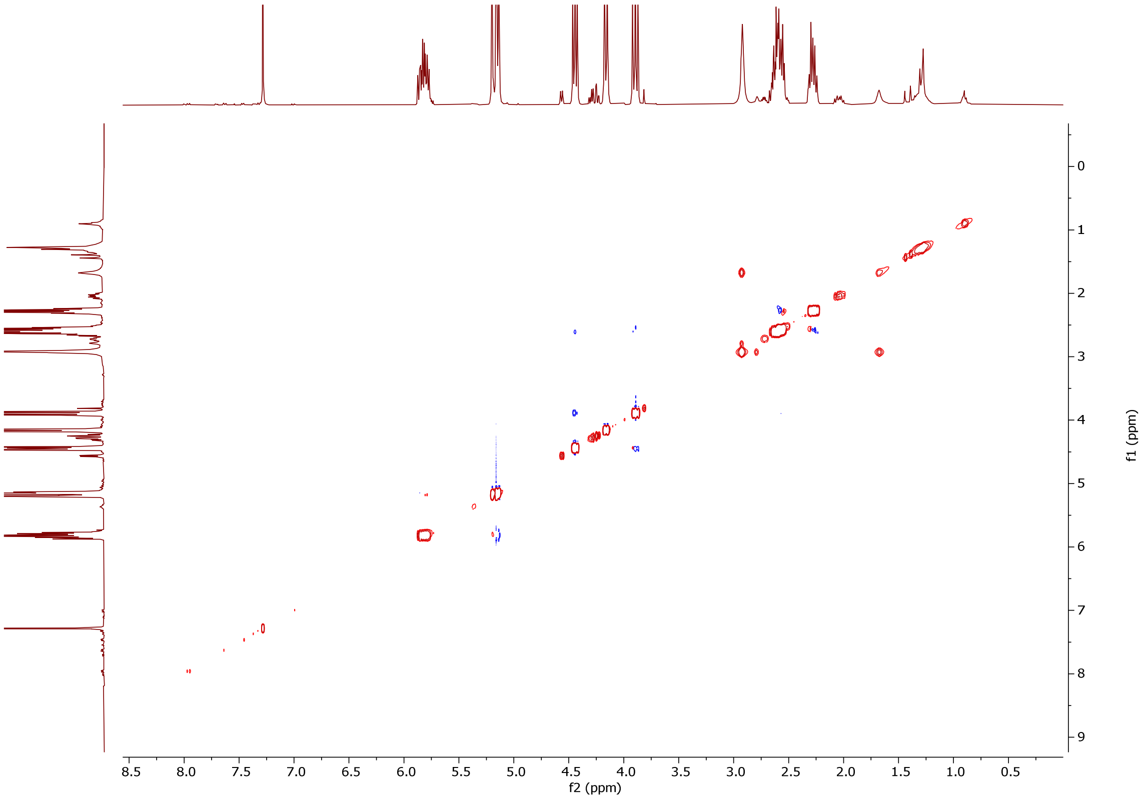

Figure S37. NMR spectra ($CDCl_3$) of 2*R*,3*S*-**5d**: a) ^{1}H, b) ^{13}C, c) COSY, d) HSQC, e) HMBC and f) NOESY.

a)

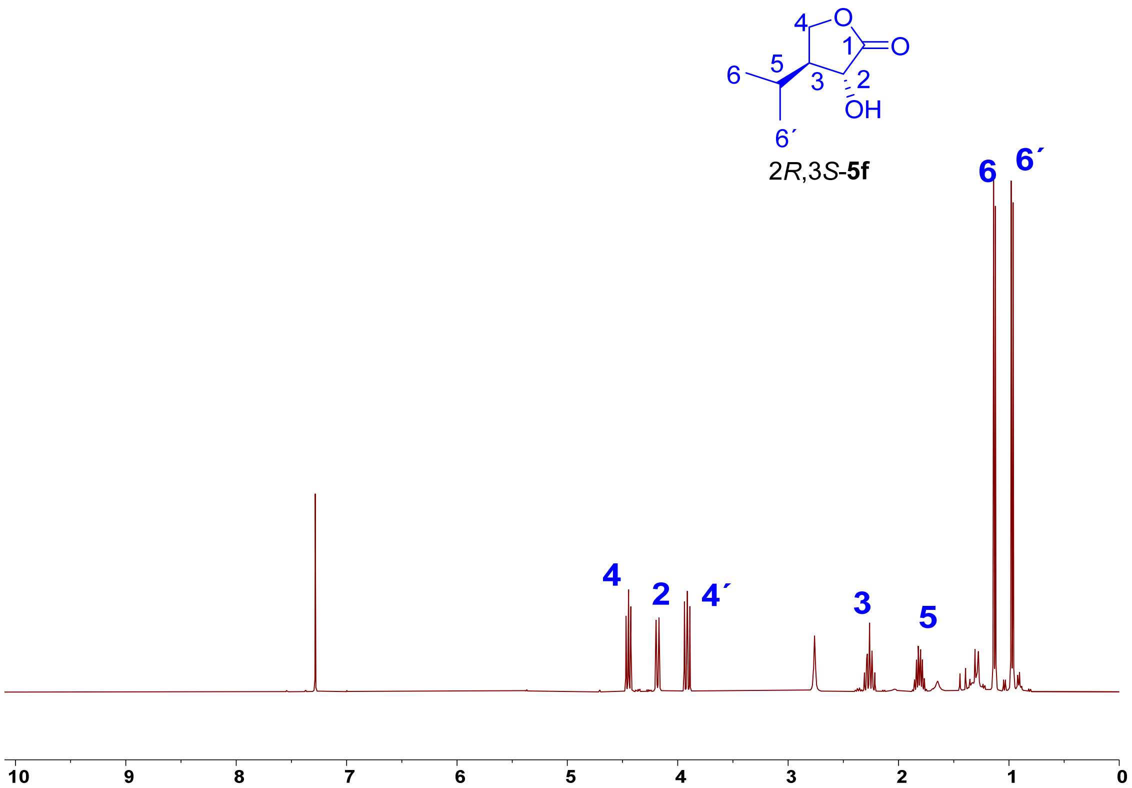

b)

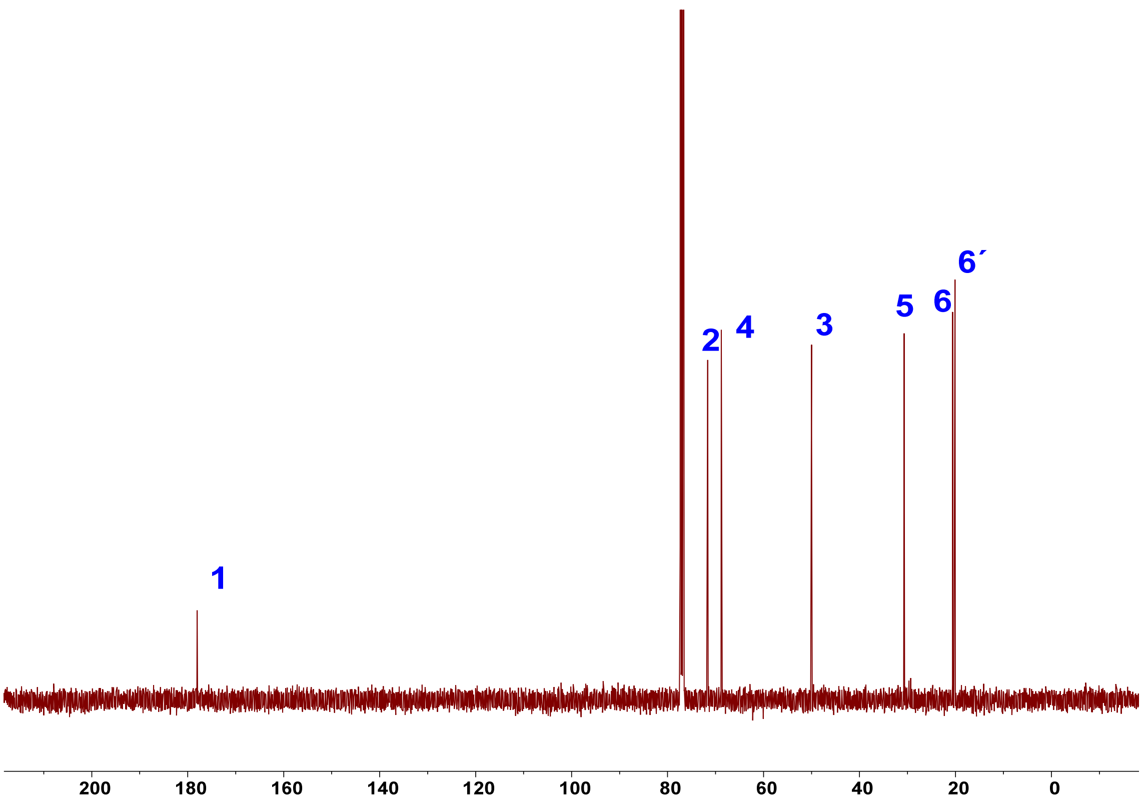
1
2
4
3
5
6
6´
200
180
160
140
120
100
80
60
40
20
0

c)

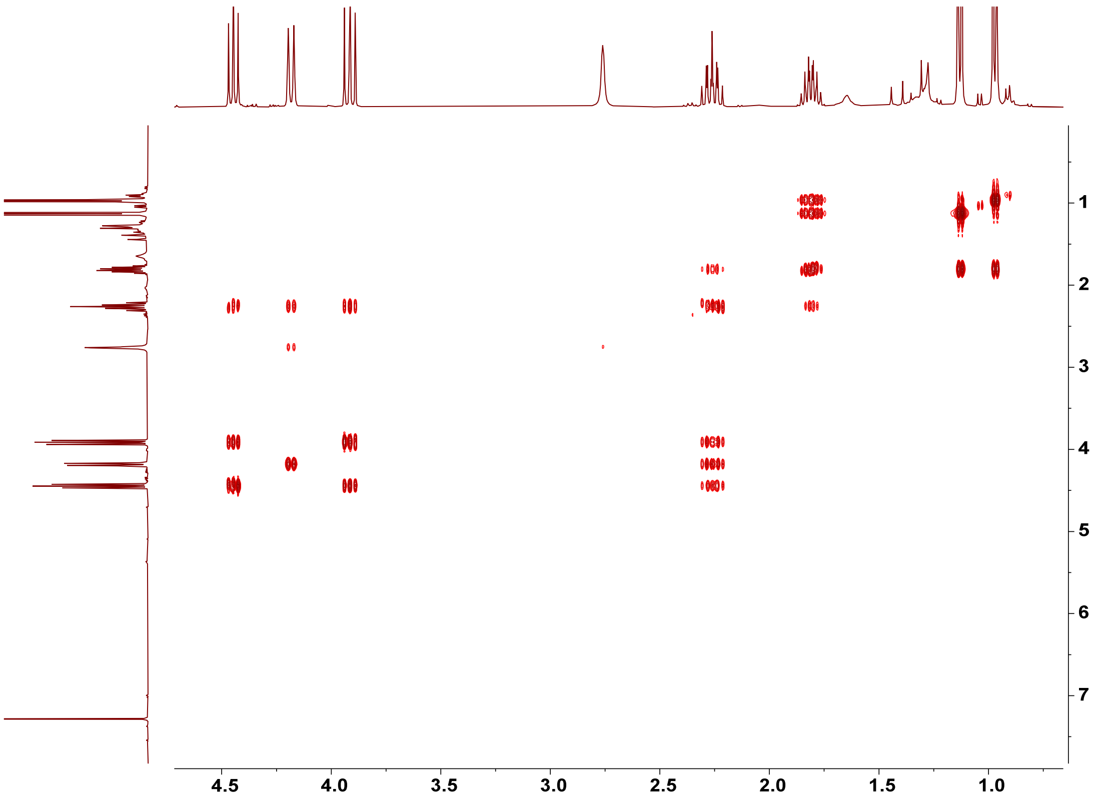
4.5
4.0
3.5
3.0
2.5
2.0
1.5
1.0
1
2
3
4
5
6
7

d)

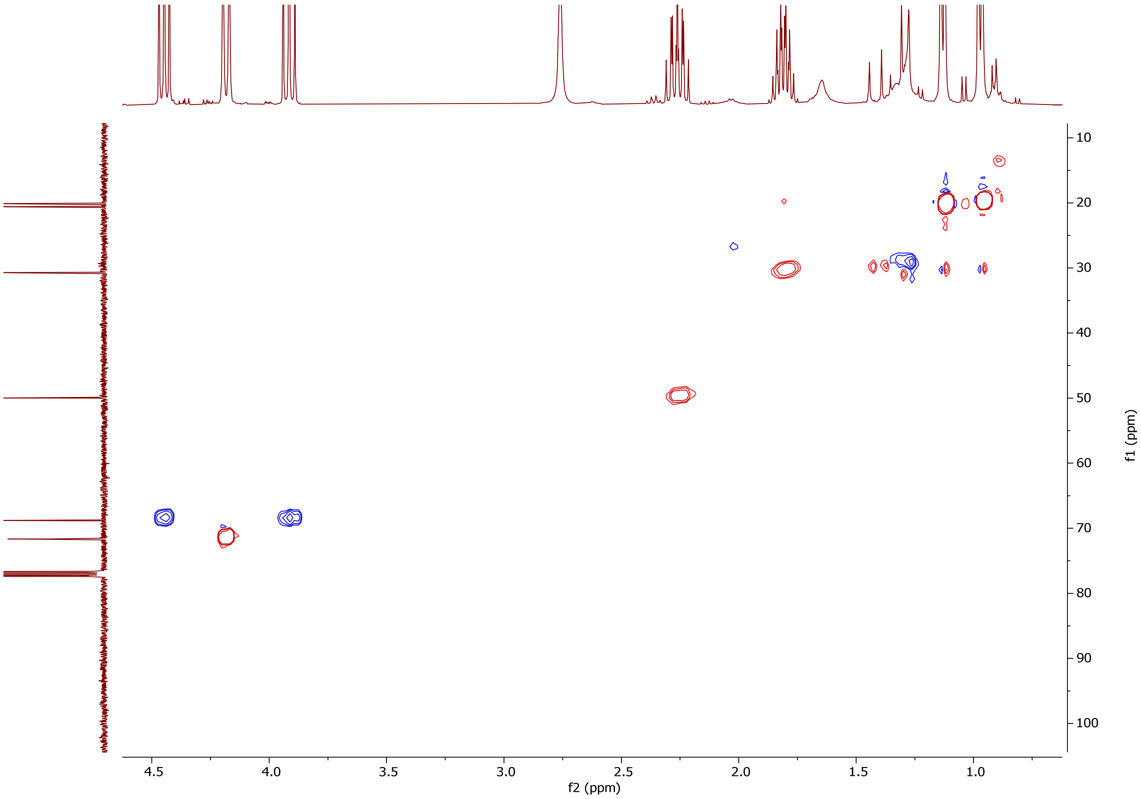

e)

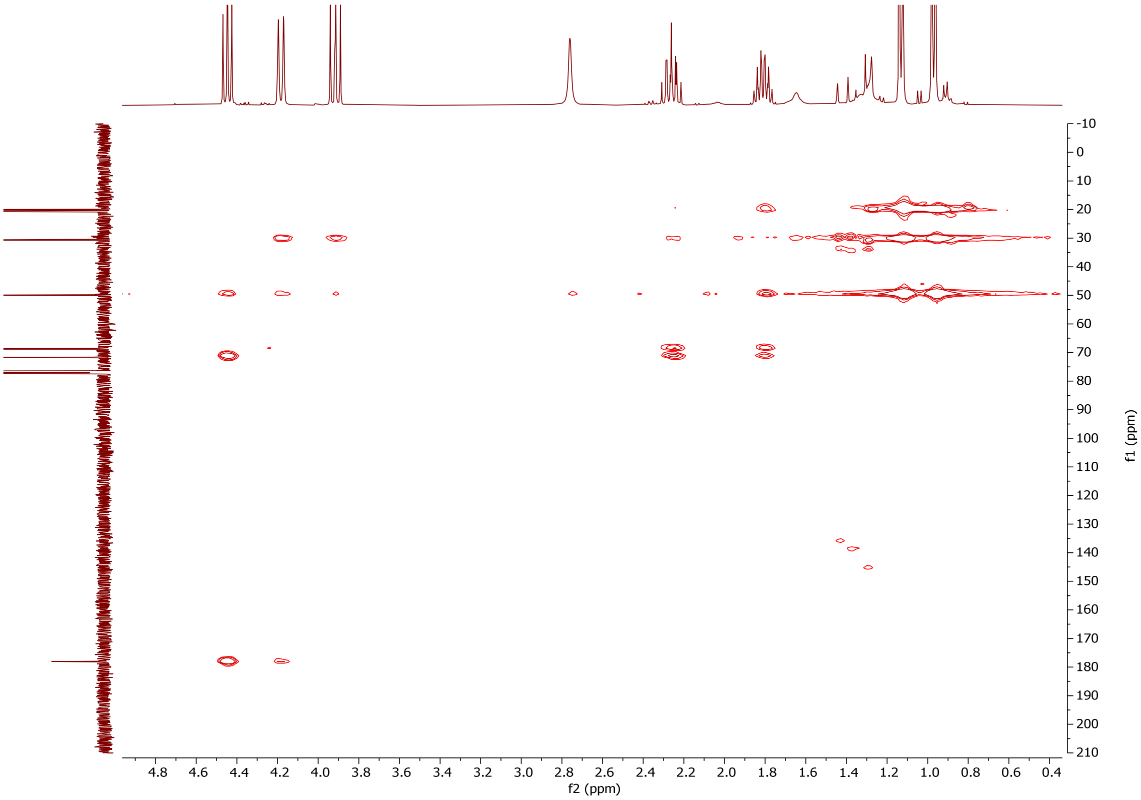

f)

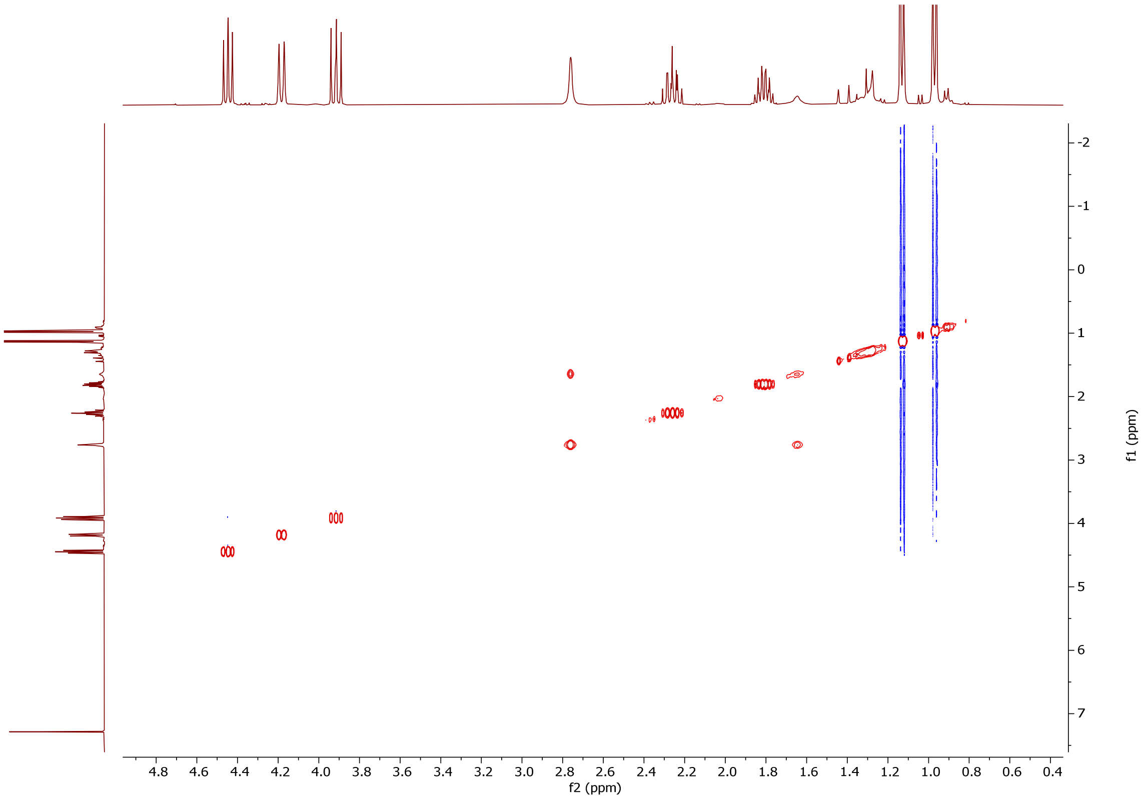

Figure S38. NMR spectra ($CDCl_3$) of 2*R*,3*S*-**5f**: a) ^{1}H, b) ^{13}C, c) COSY, d) HSQC, e) HMBC and f) NOESY.

a)

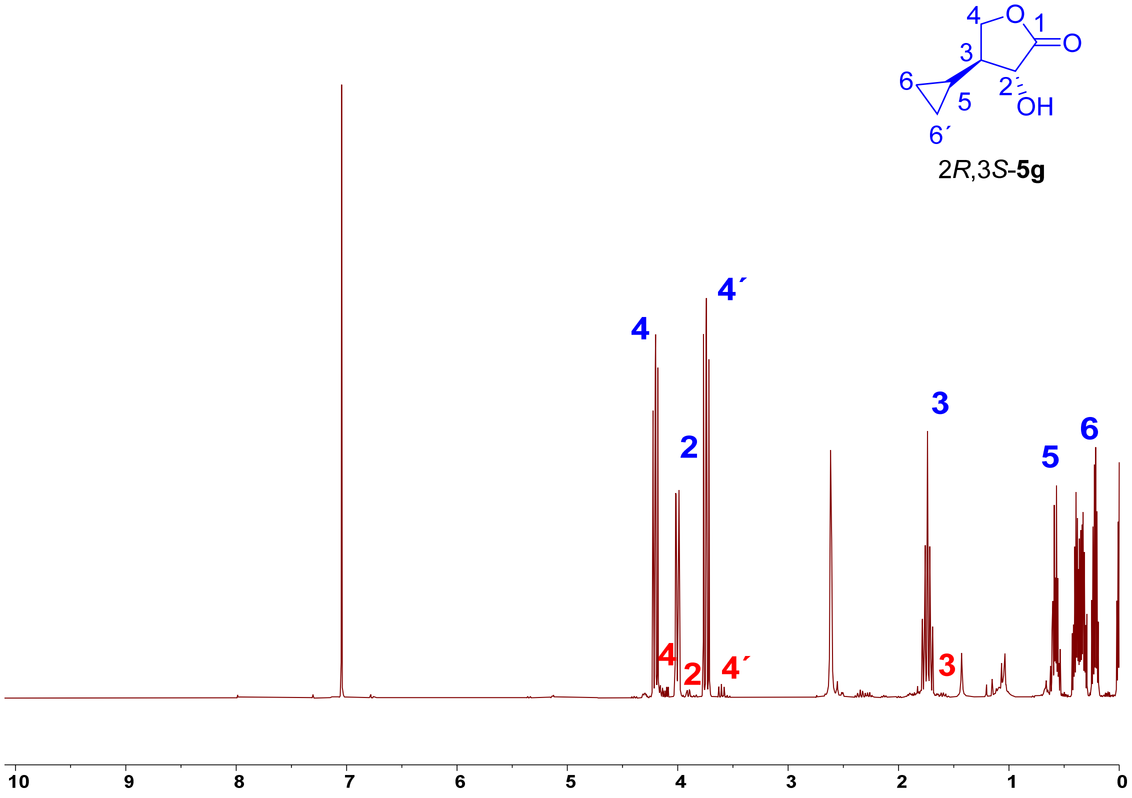

b)

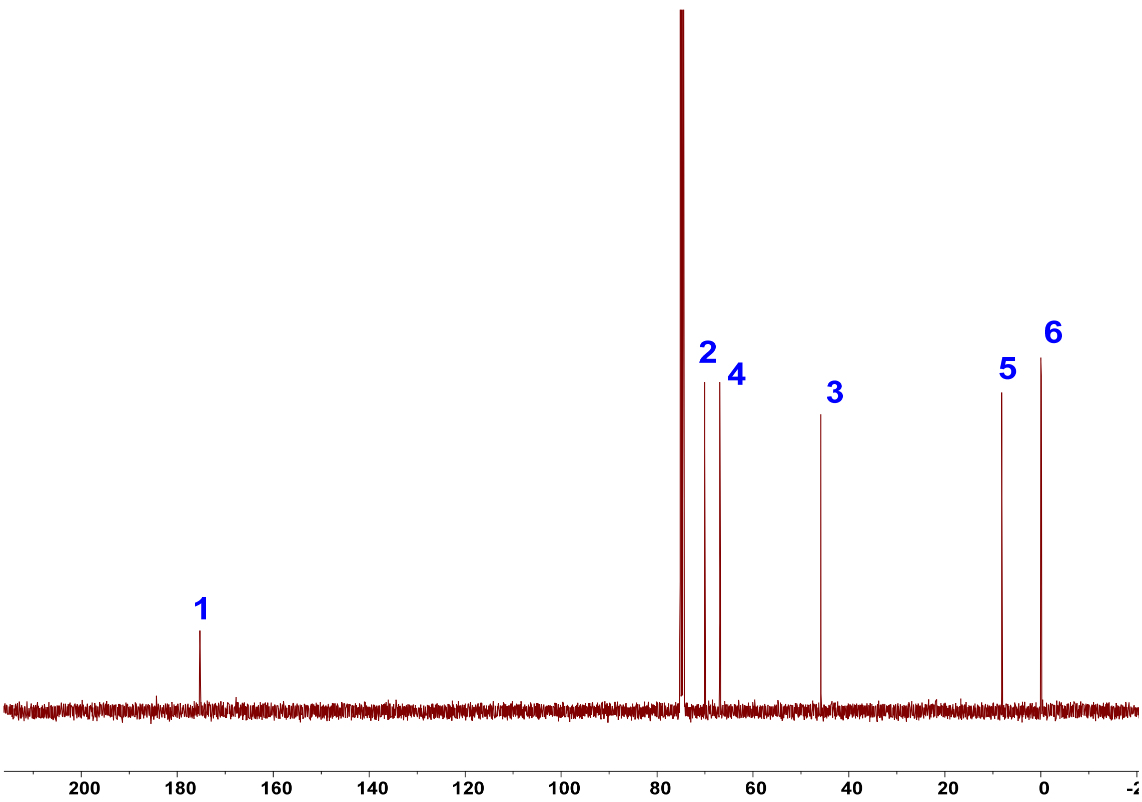

1
2
4
3
5
6
200
180
160
140
120
100
80
60
40
20
0

c)

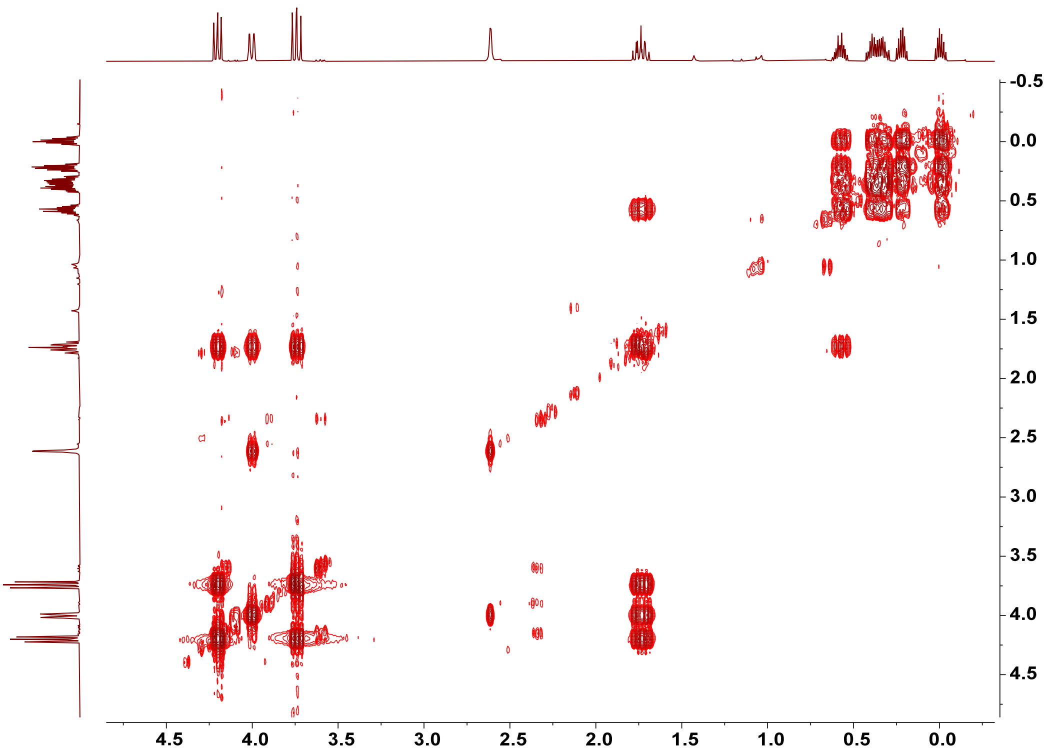

-0.5
0.0
0.5
1.0
1.5
2.0
2.5
3.0
3.5
4.0
4.5

d)

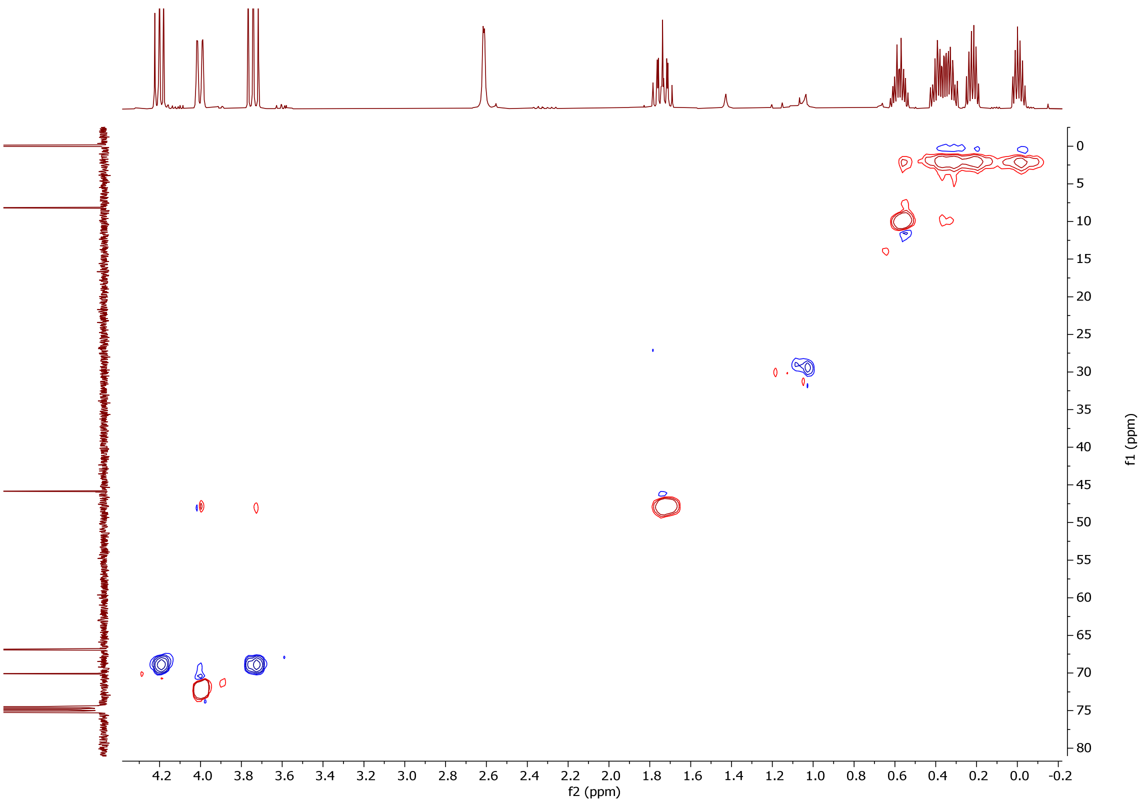

e)

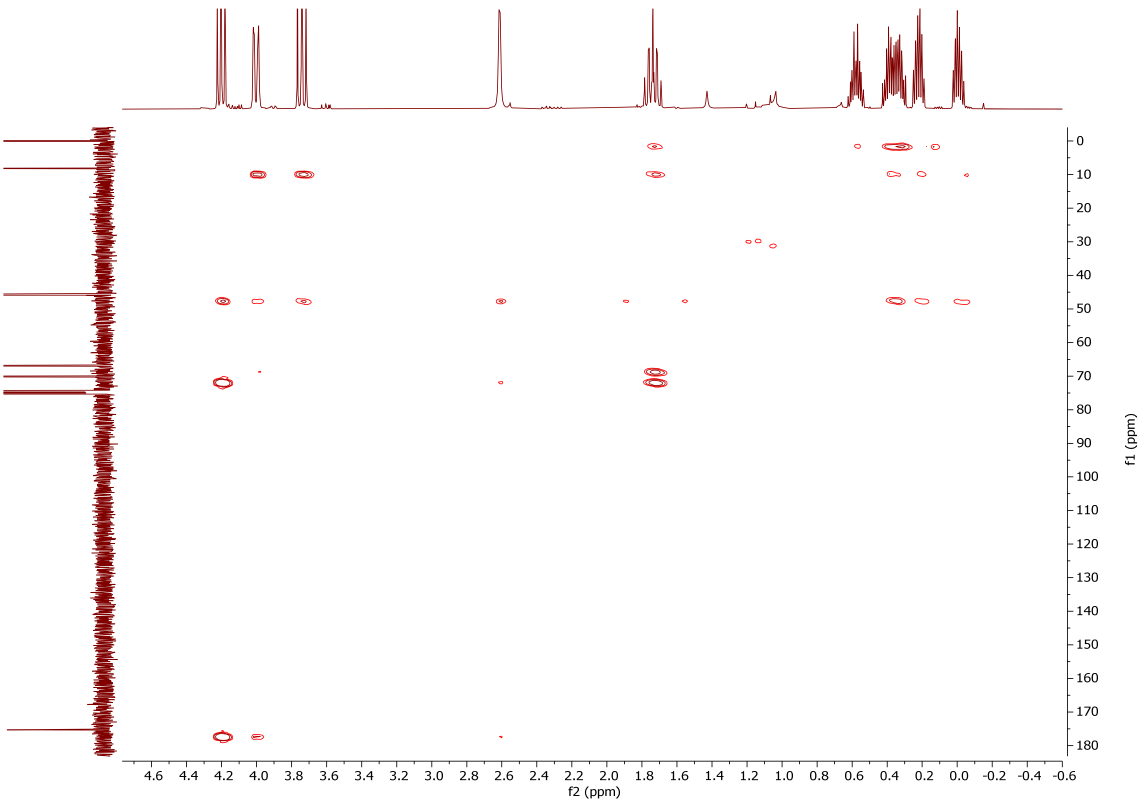

f)

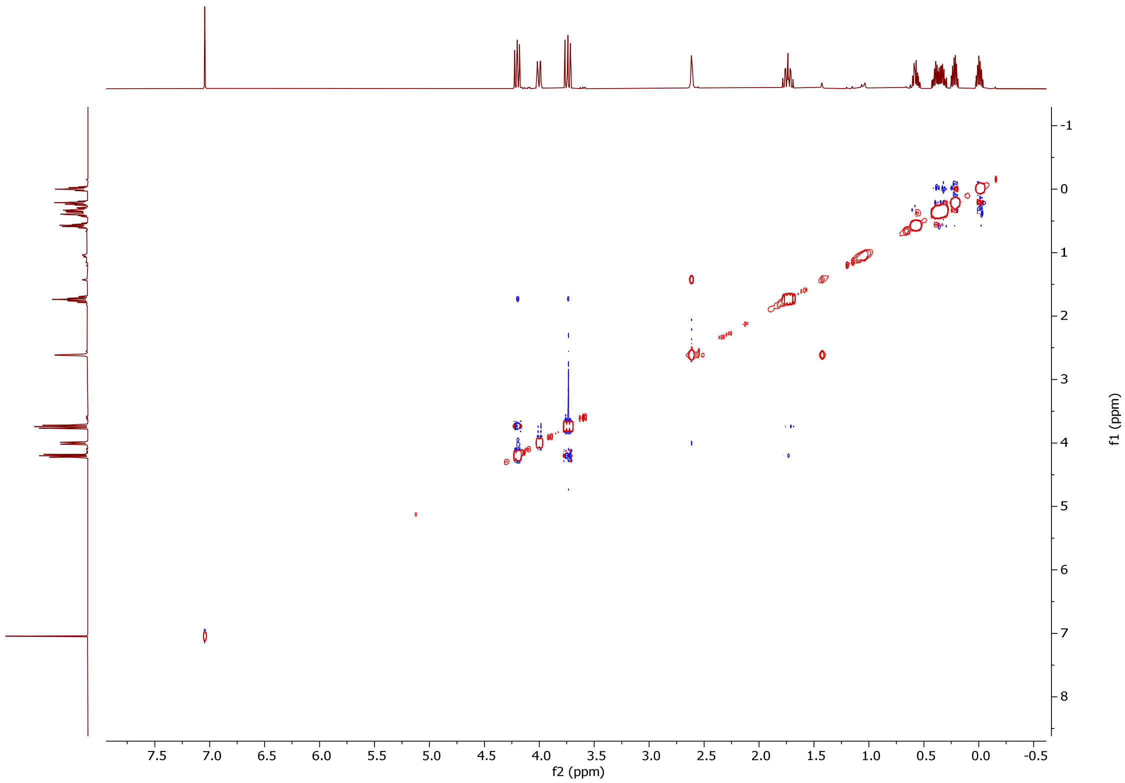

Figure S39. NMR spectra ($CDCl_3$) of 2*R*,3*S*-**5g**: a) ^{1}H, b) ^{13}C, c) COSY, d) HSQC, e) HMBC and f) NOESY.

a)

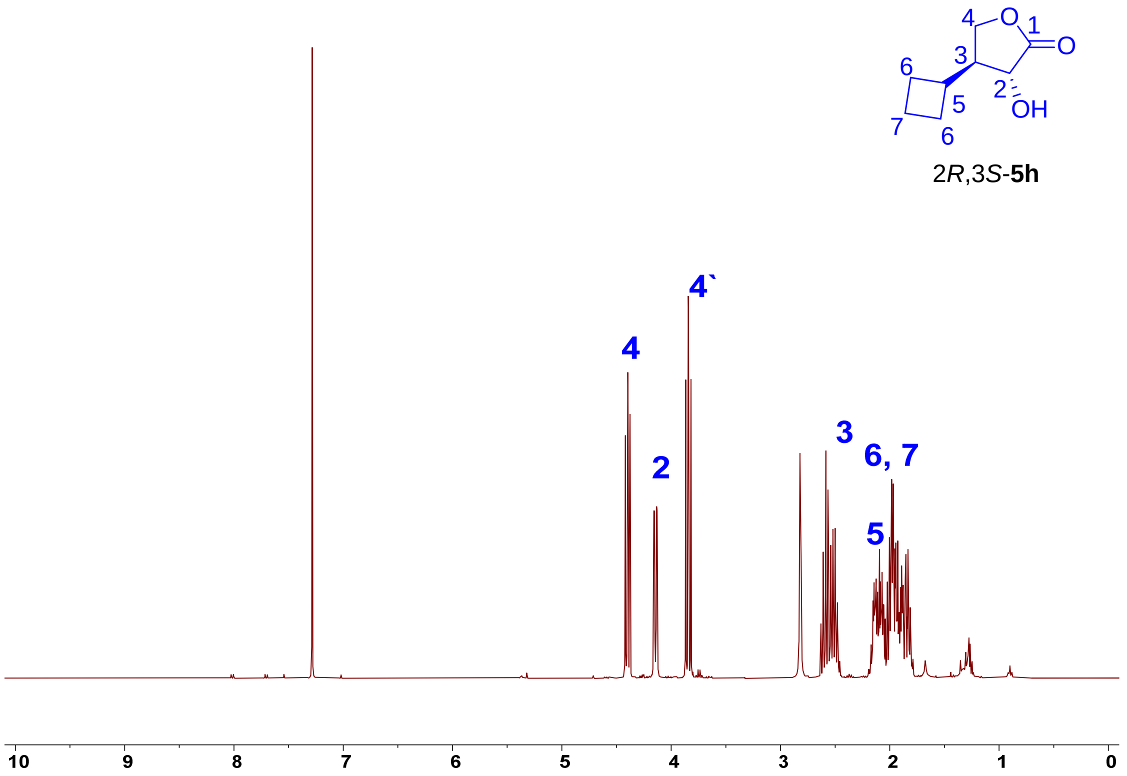

b)

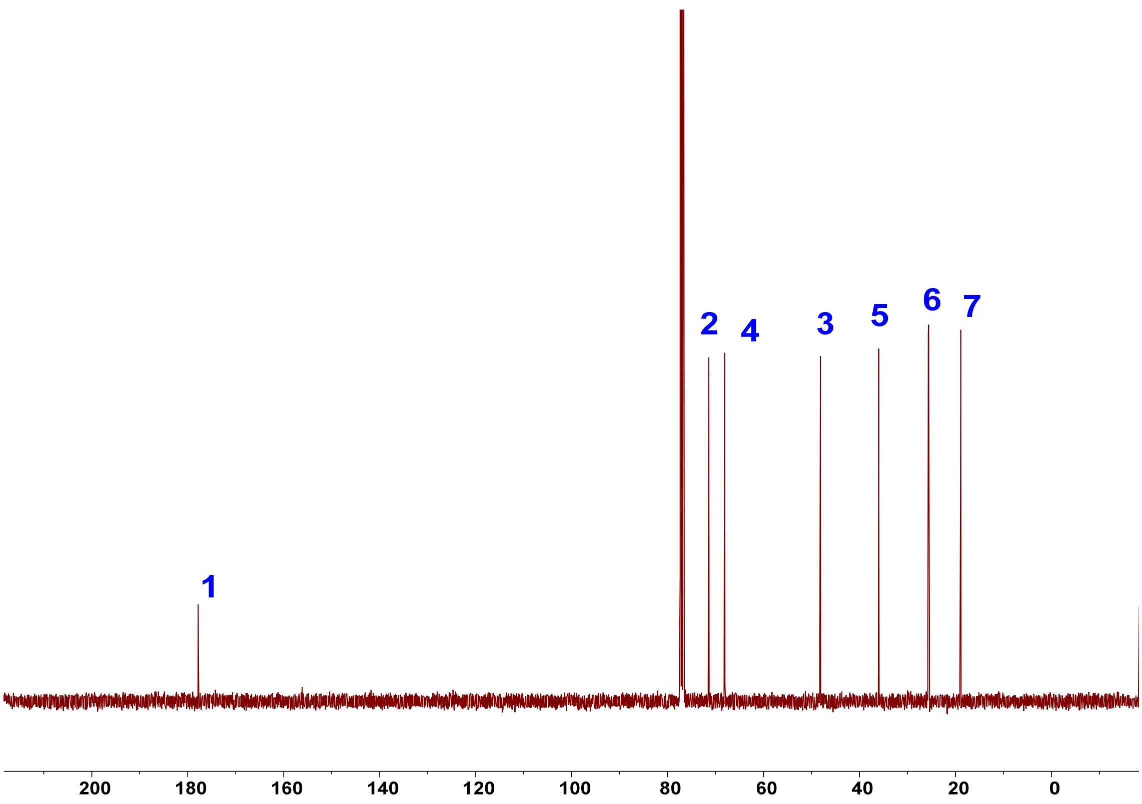
1
2
4
3
5
6
7
200
180
160
140
120
100
80
60
40
20
0

c)

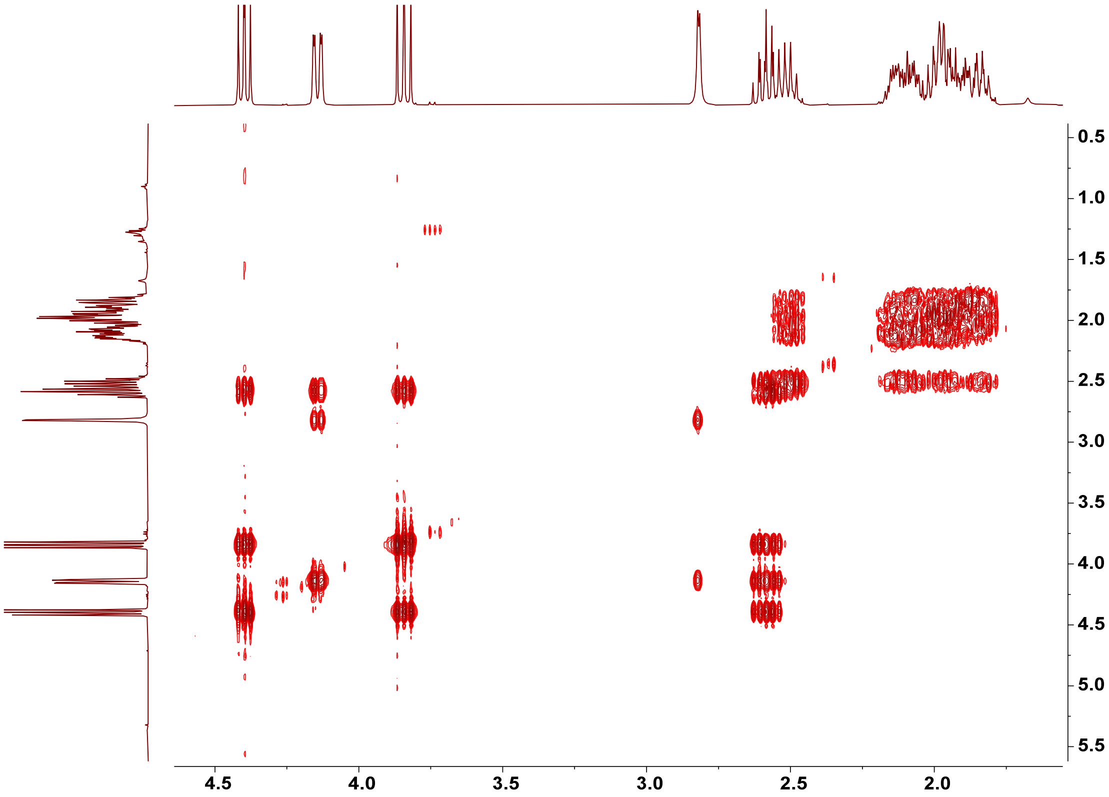
4.5
4.0
3.5
3.0
2.5
2.0
0.5
1.0
1.5
2.0
2.5
3.0
3.5
4.0
4.5
5.0
5.5

d)

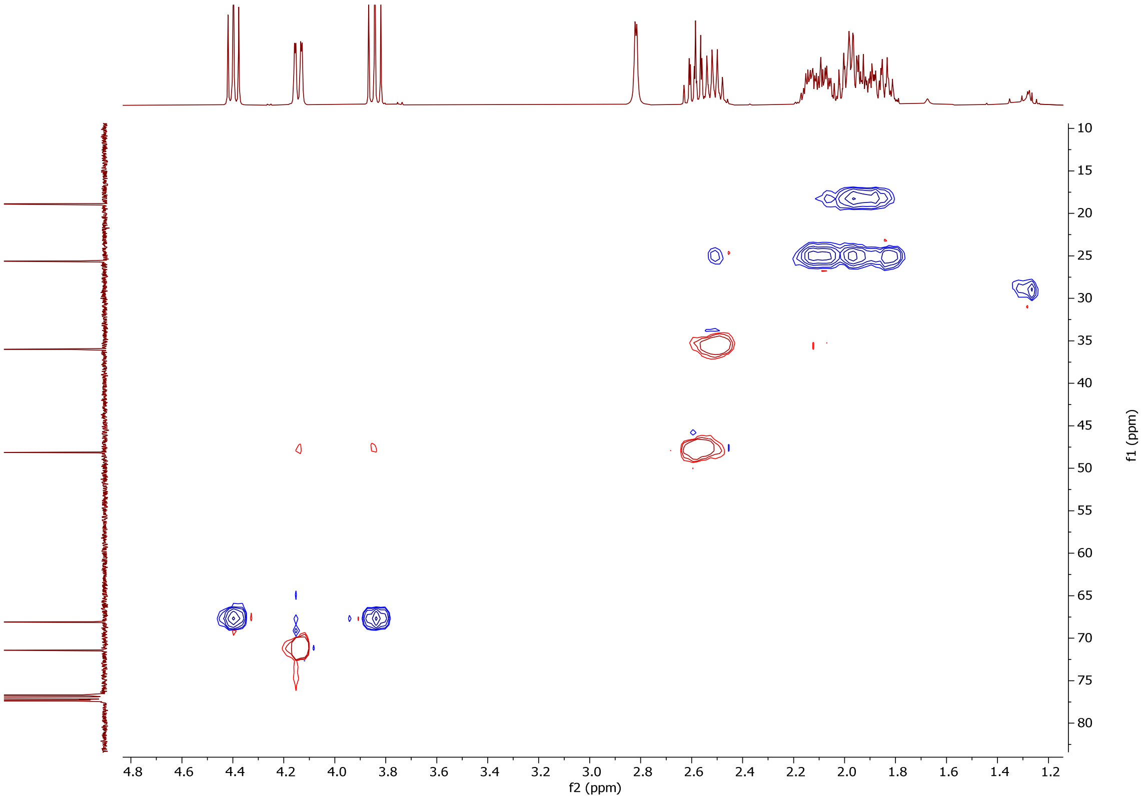

e)

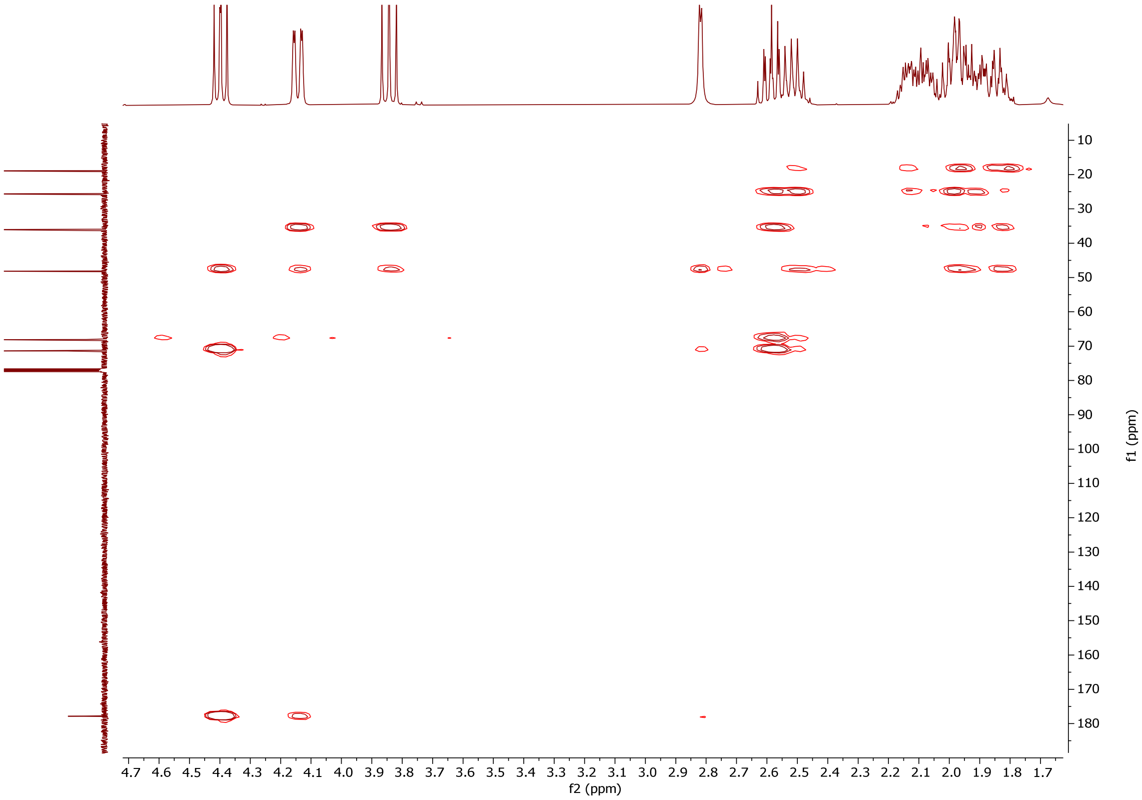

f)

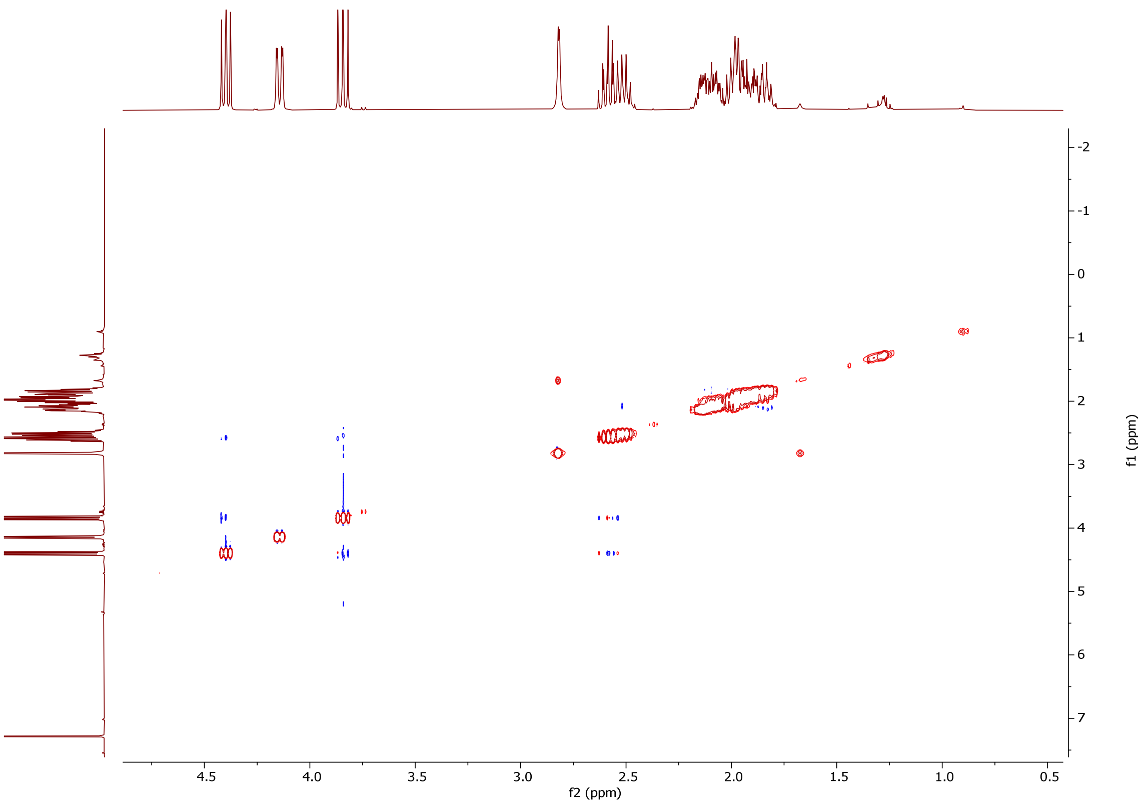

Figure S40. NMR spectra ($CDCl_3$) of 2*R*,3*S*-**5h**: a) ^{1}H, b) ^{13}C, c) COSY, d) HSQC, e) HMBC and f) NOESY.

a)

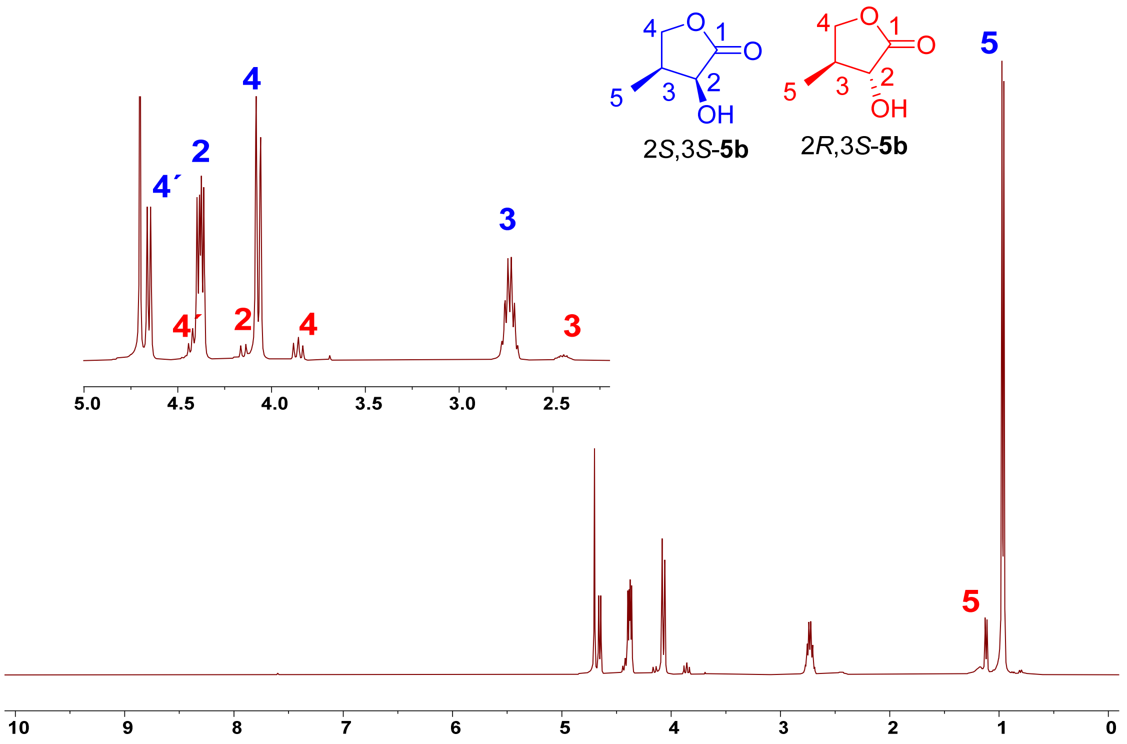

b)

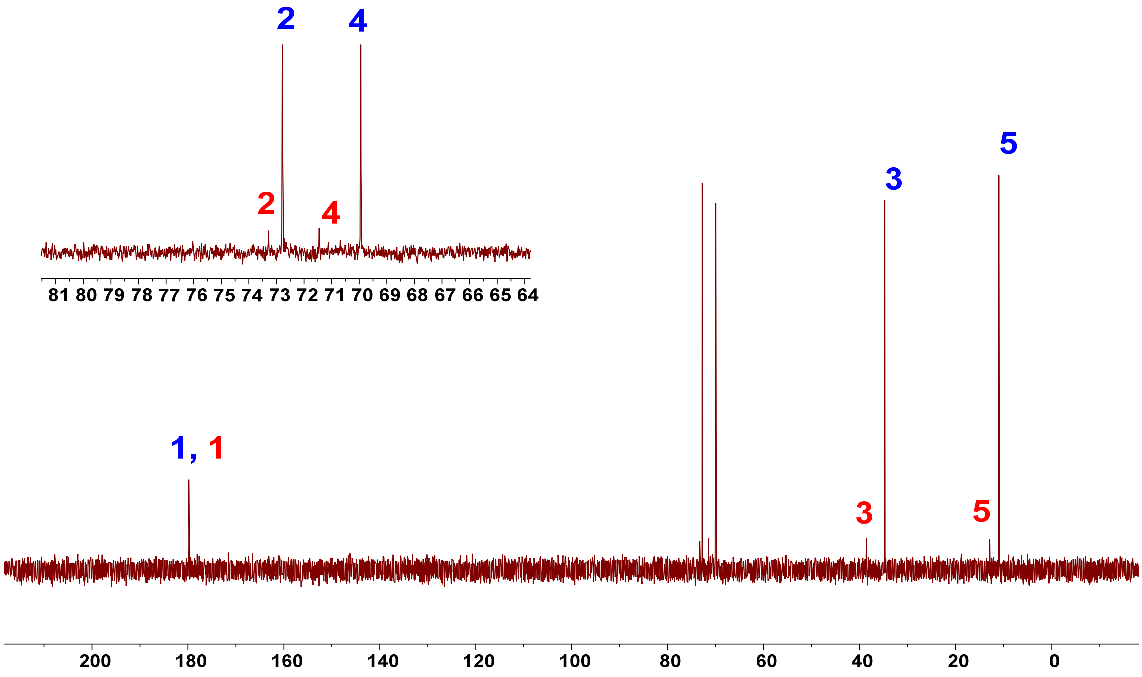
2
4
2
4
81 80 79 78 77 76 75 74 73 72 71 70 69 68 67 66 65 64
1, 1
3
5
3
5
200
180
160
140
120
100
80
60
40
20
0

c)

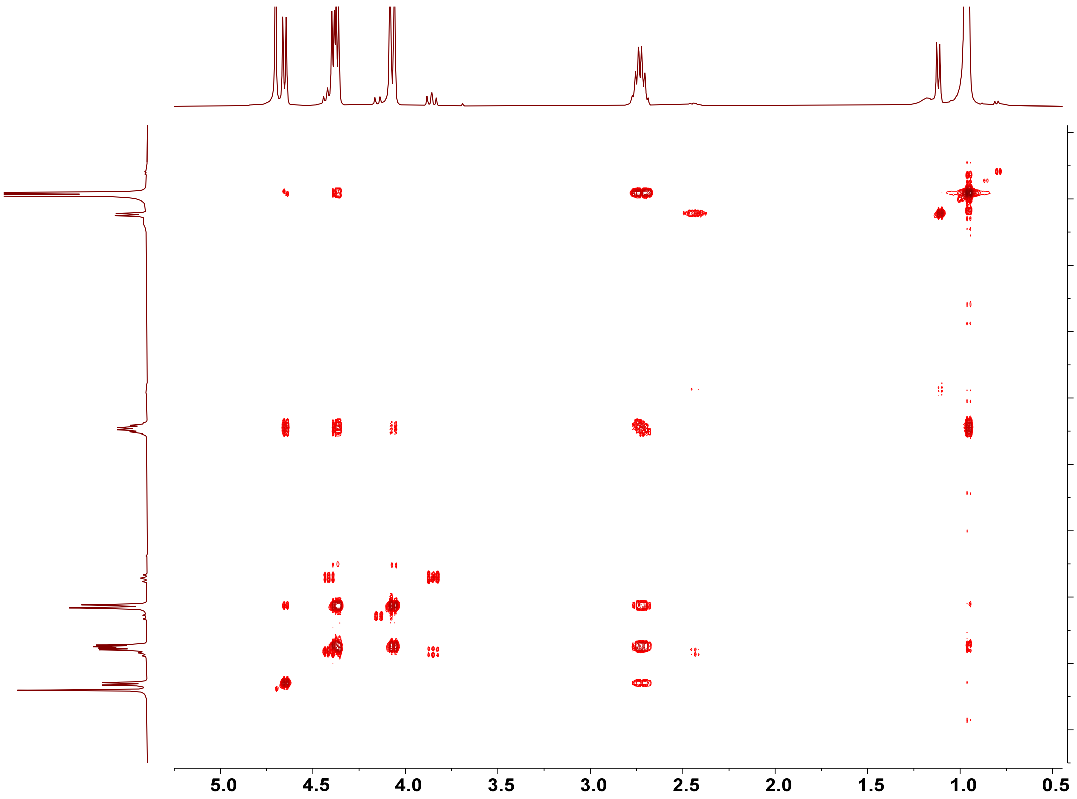
5.0
4.5
4.0
3.5
3.0
2.5
2.0
1.5
1.0
0.5

d)

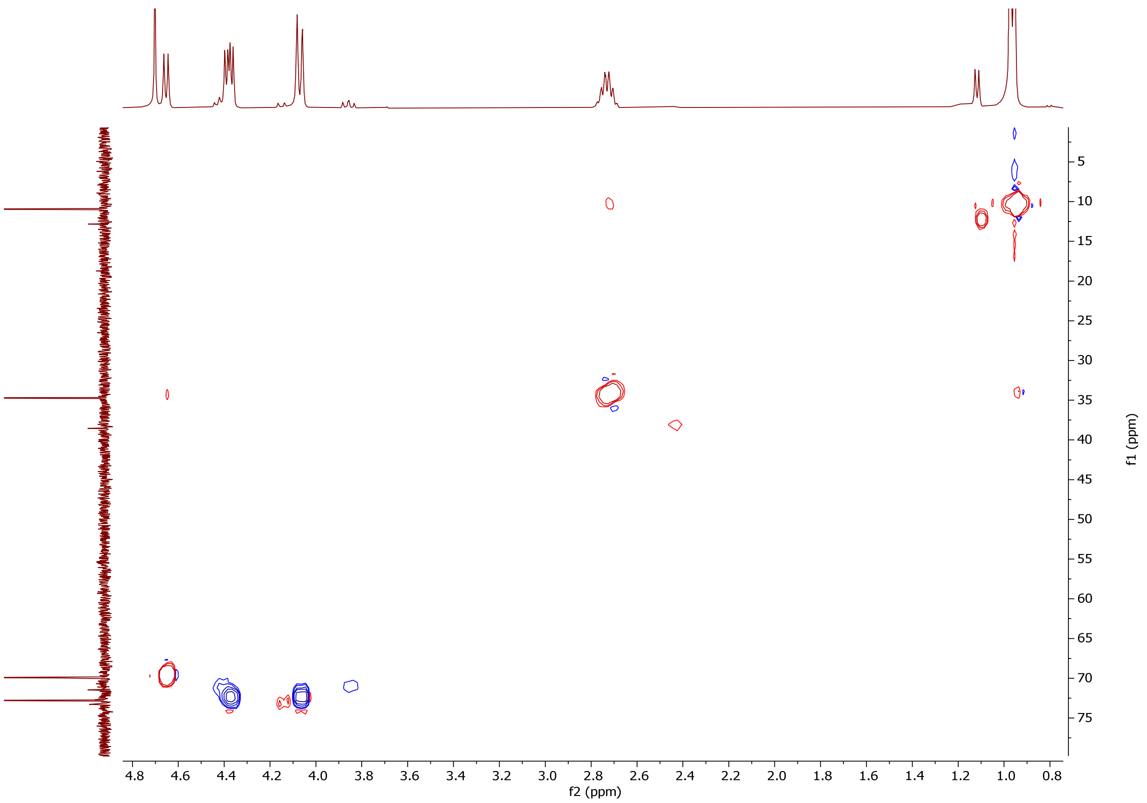

e)

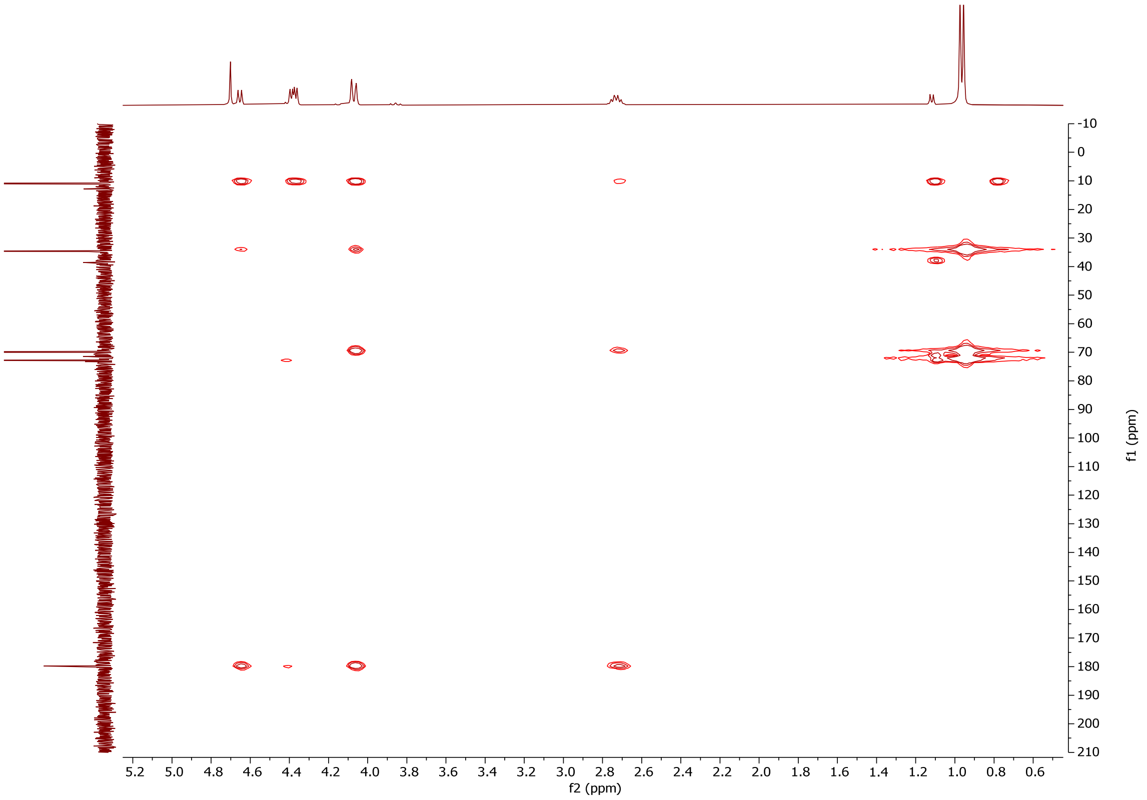

f)

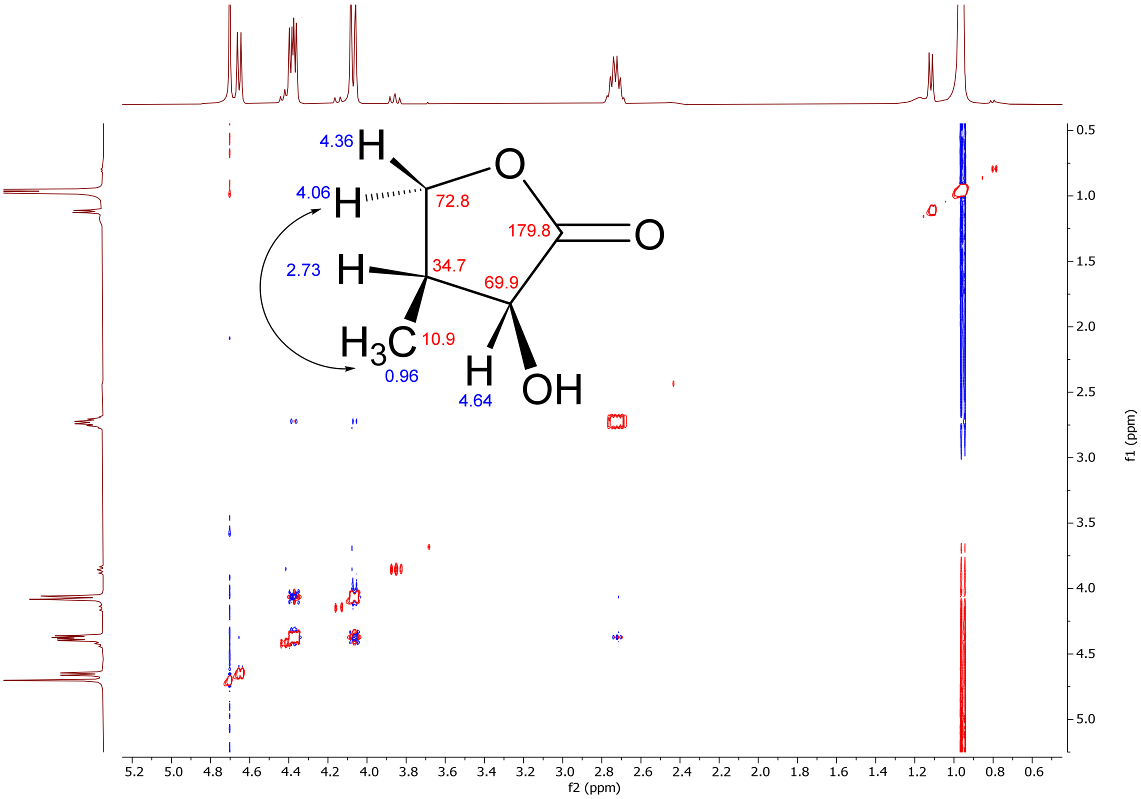

Figure S41. NMR spectra (D_2O) of 2*S*,3*S*-**5b**: a) ^{1}H, b) ^{13}C, c) COSY, d) HSQC, e) HMBC and f) NOESY.

a)

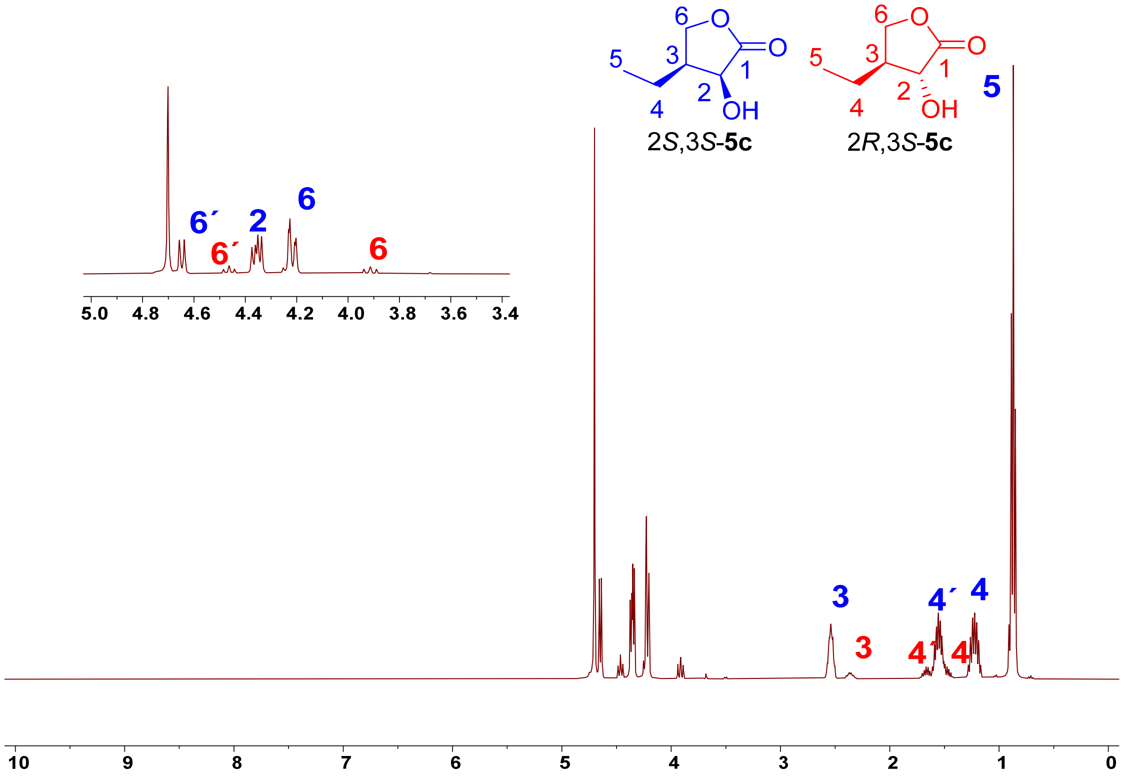

b)

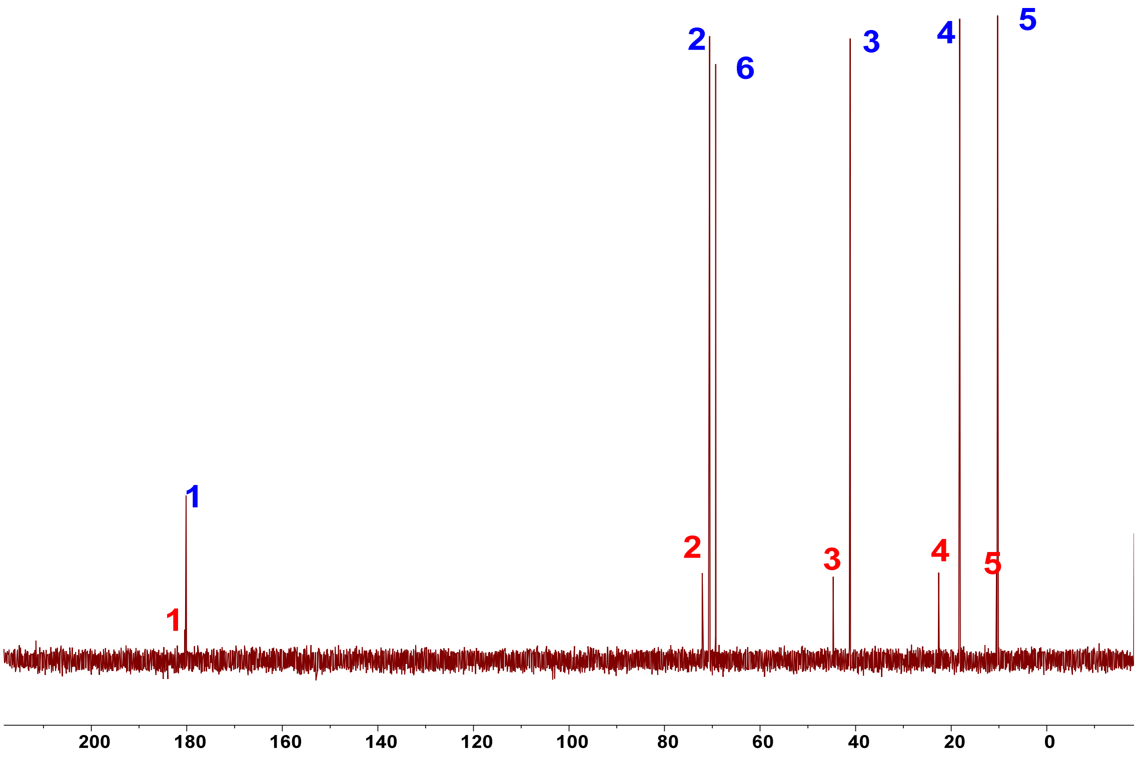

1
2
6
3
4
5
1
2
3
4
5
200
180
160
140
120
100
80
60
40
20
0

c)

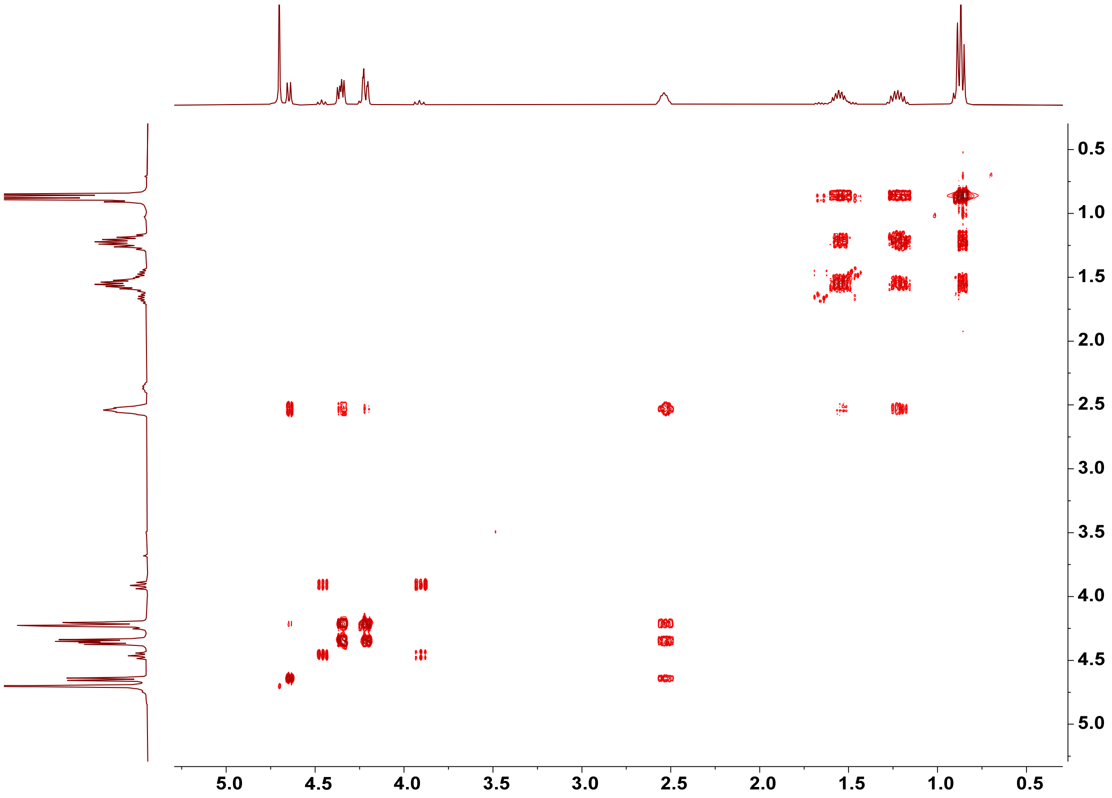

0.5
1.0
1.5
2.0
2.5
3.0
3.5
4.0
4.5
5.0
5.0
4.5
4.0
3.5
3.0
2.5
2.0
1.5
1.0
0.5

d)

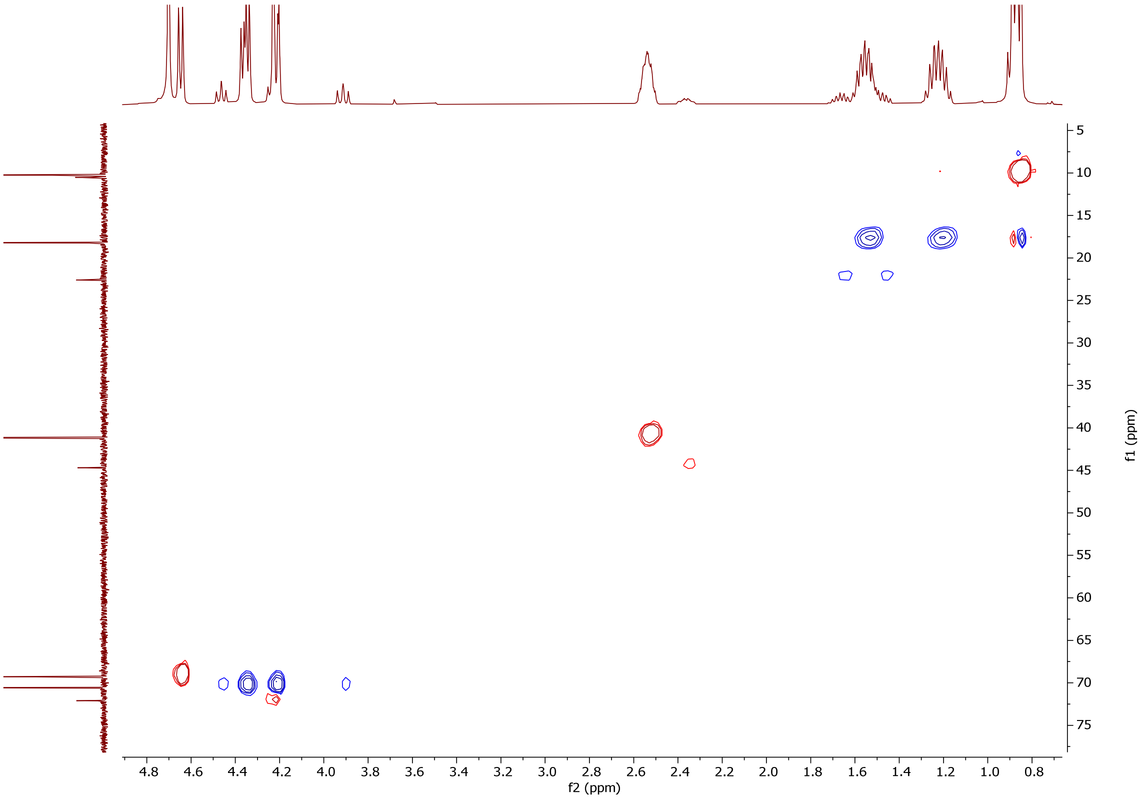

e)

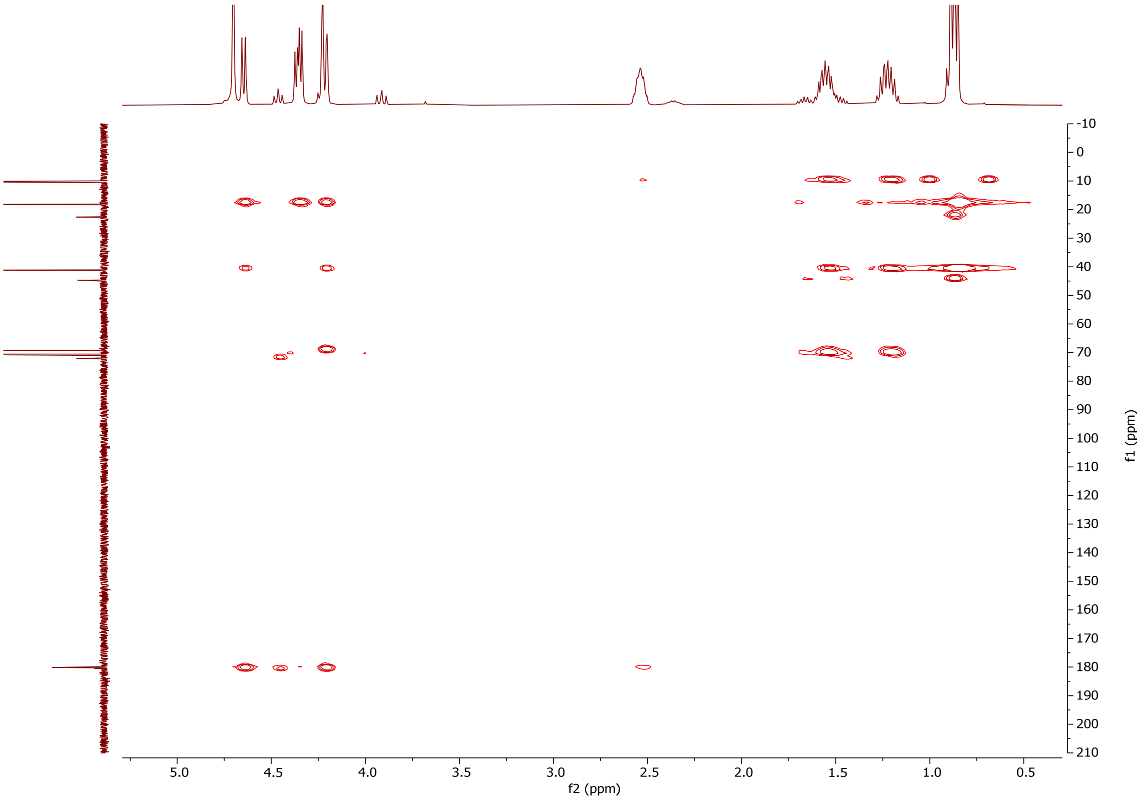

f)

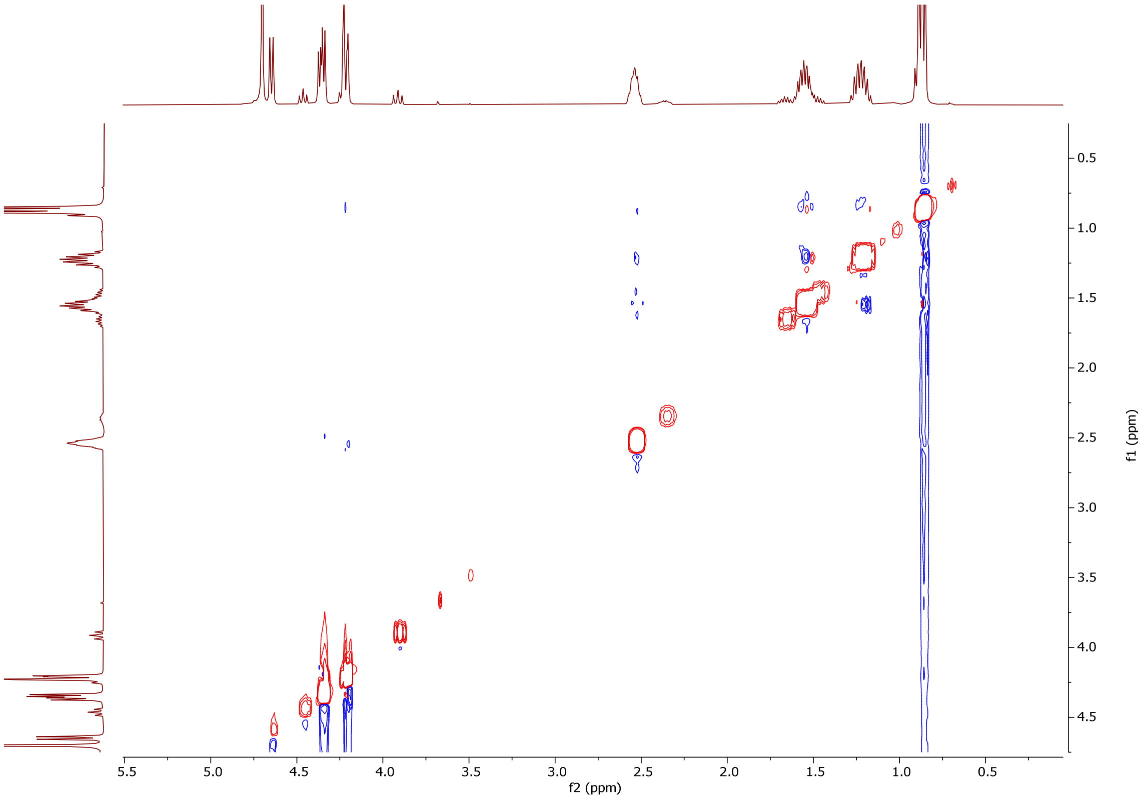

Figure S42. NMR spectra (D_2O) of 2*S*,3*S*-**5c**: a) ^{1}H, b) ^{13}C, c) COSY, d) HSQC, e) HMBC and f) NOESY.

a)

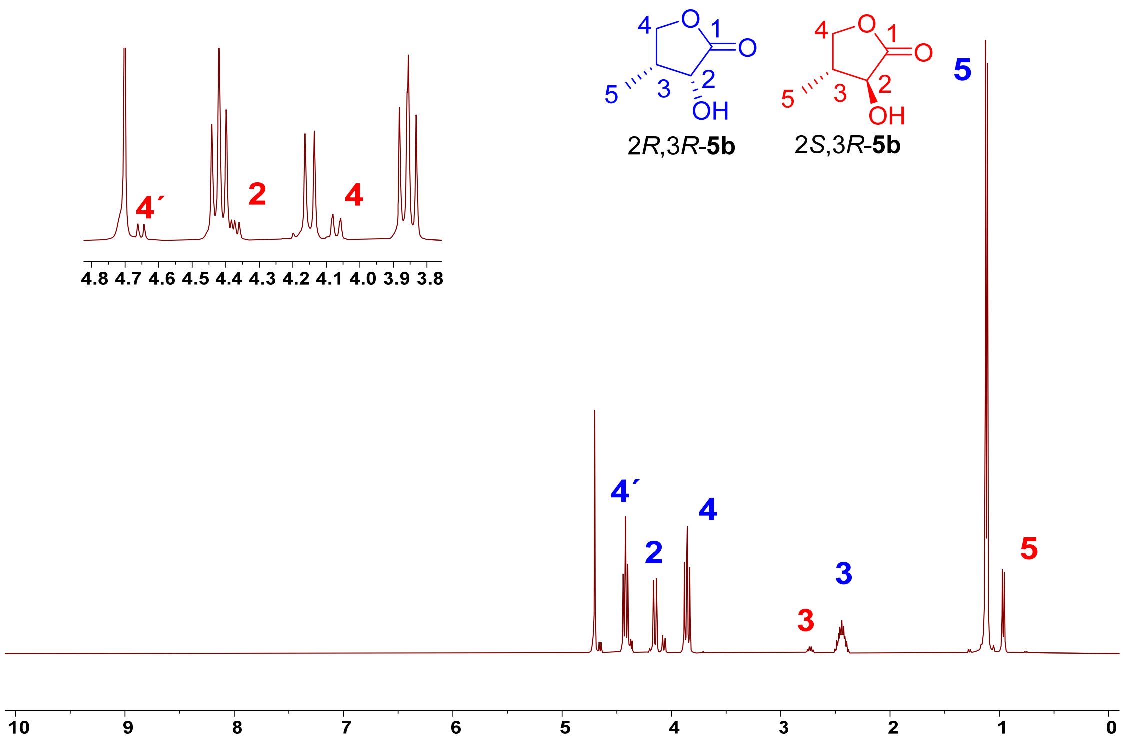

b)

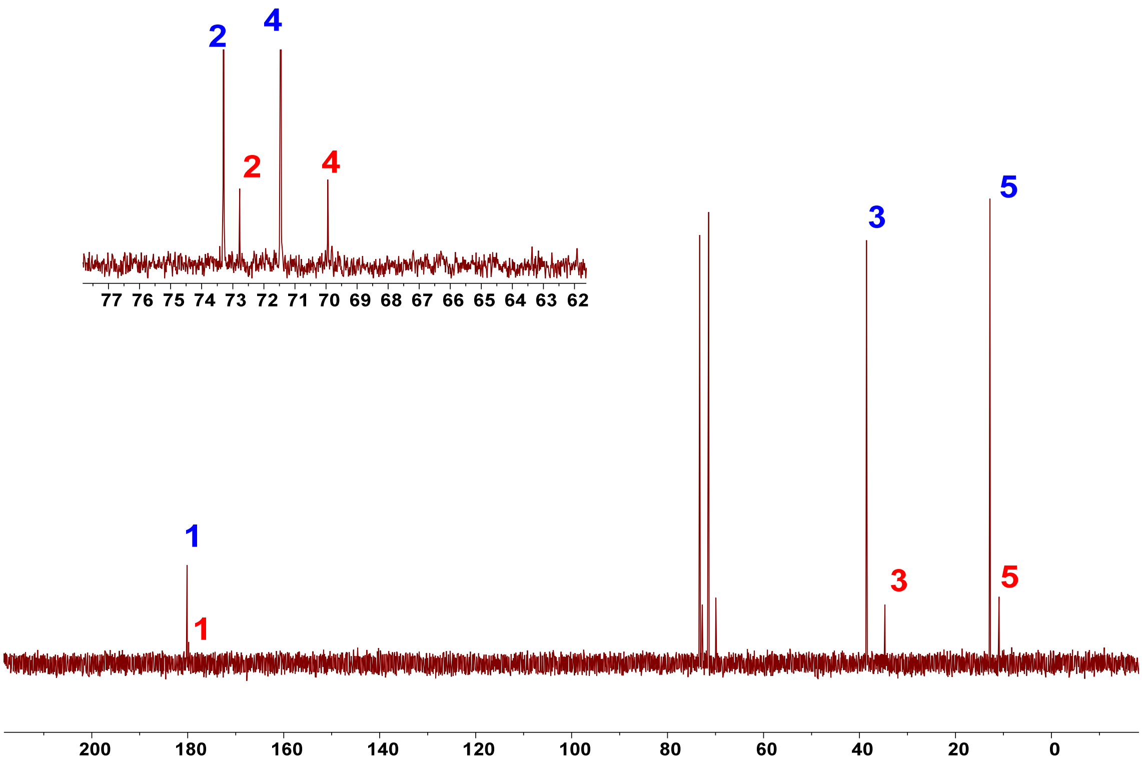
2
4
2
4
77 76 75 74 73 72 71 70 69 68 67 66 65 64 63 62
1
1
3
3
5
5
200 180 160 140 120 100 80 60 40 20 0

c)

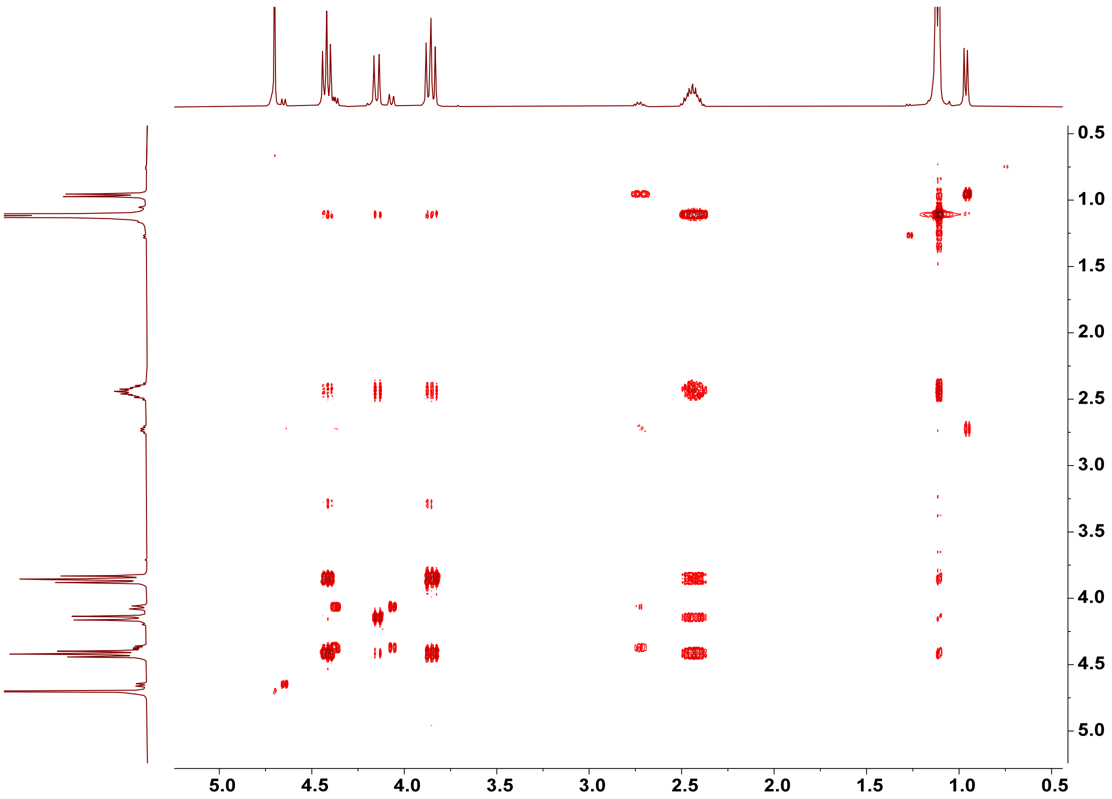
0.5
1.0
1.5
2.0
2.5
3.0
3.5
4.0
4.5
5.0
5.0 4.5 4.0 3.5 3.0 2.5 2.0 1.5 1.0 0.5

d)

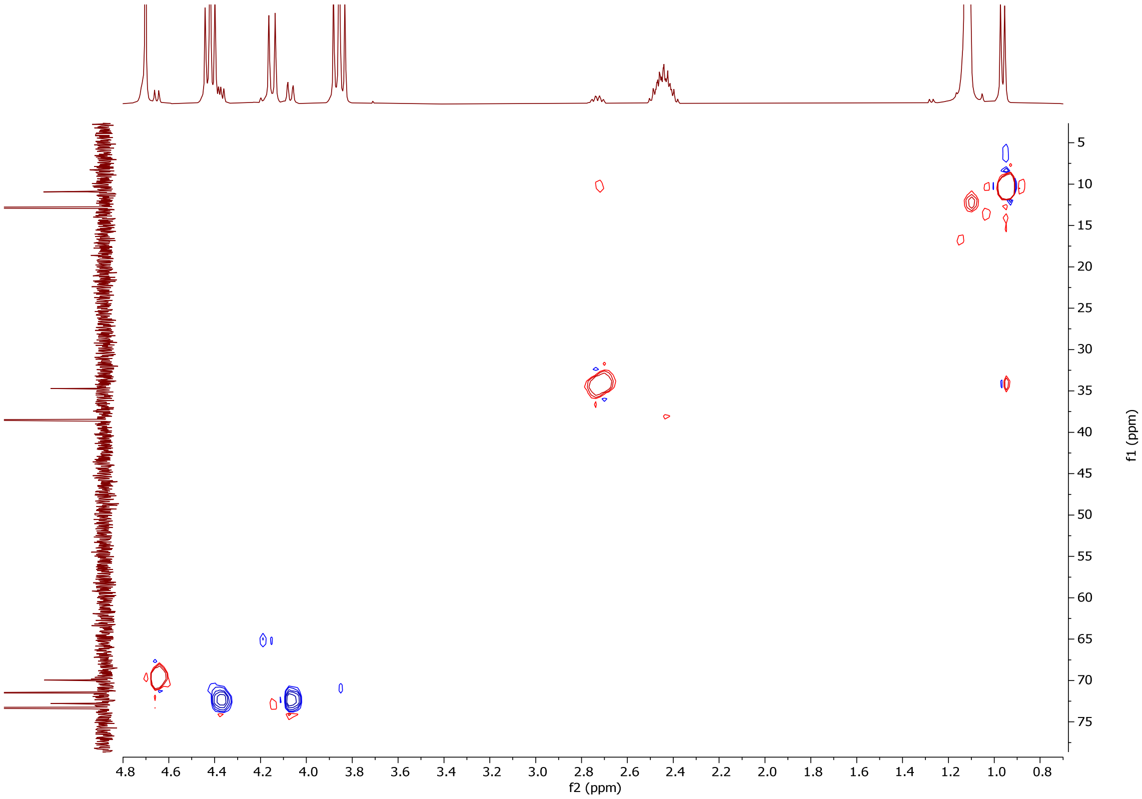

e)

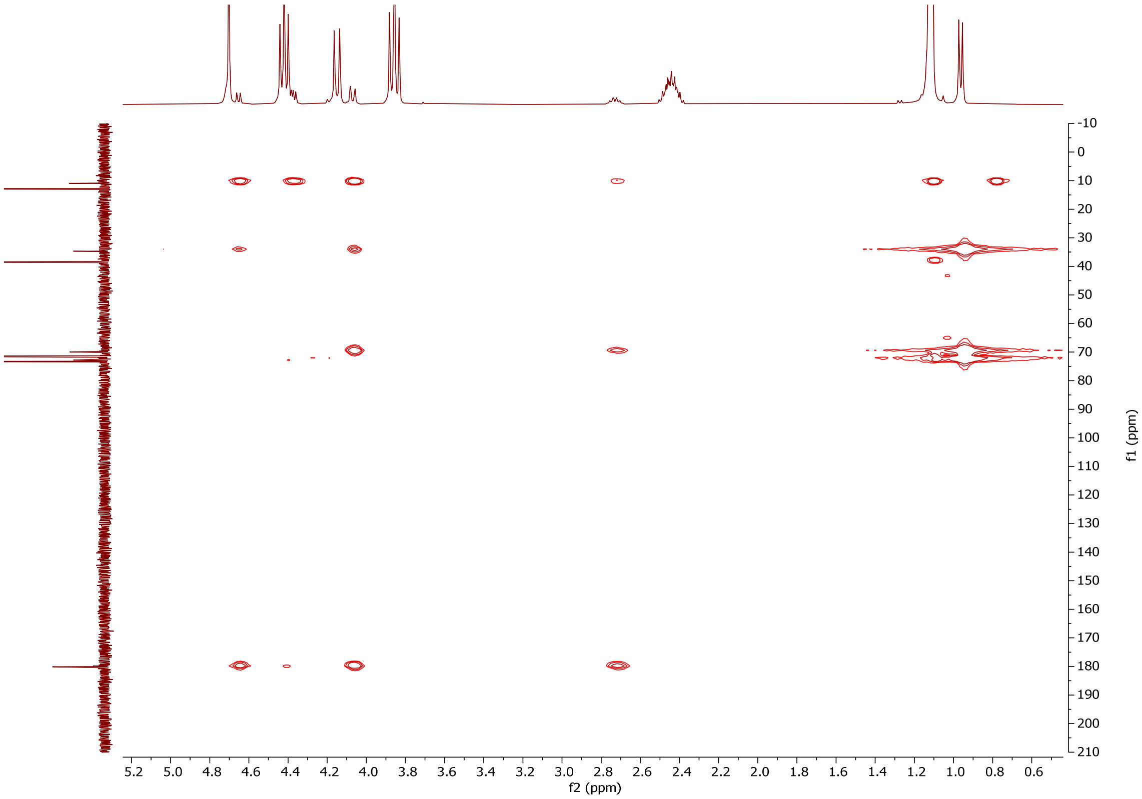

f)

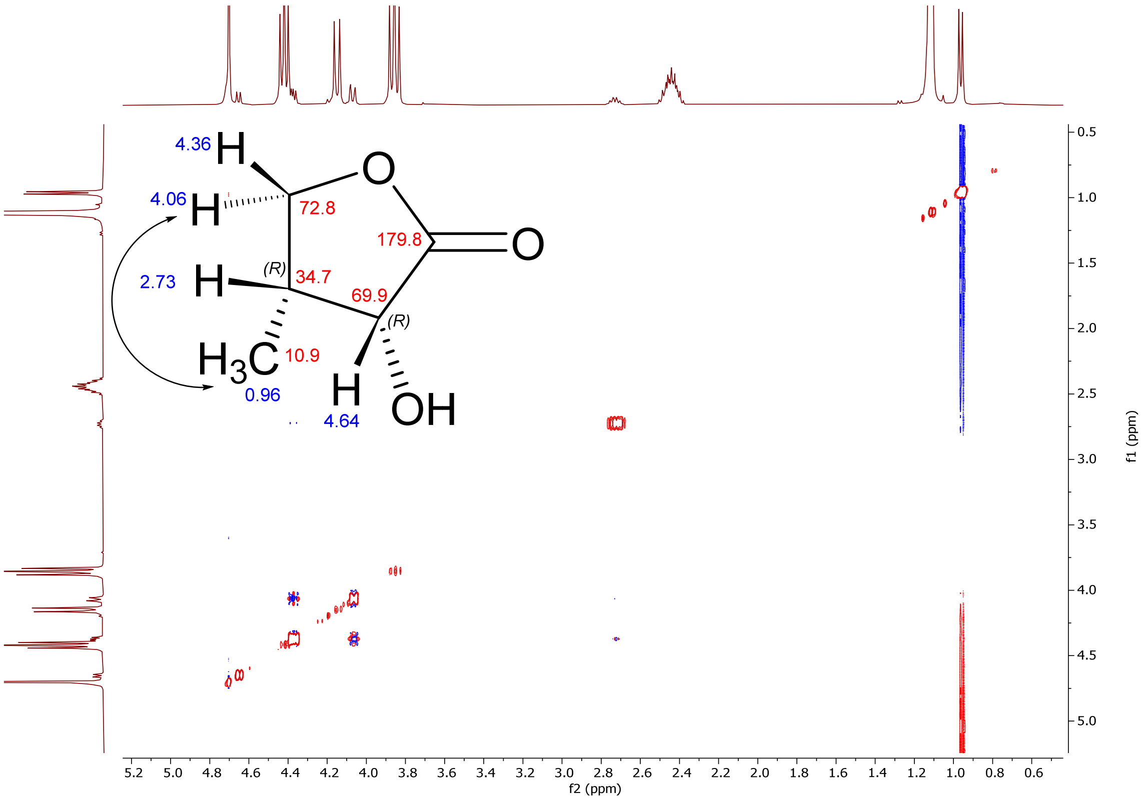

Figure S43. NMR spectra (D_2O) of 2*R*,3*R*-**5b**: a) ^1H, b) ^{13}C, c) COSY, d) HSQC, e) HMBC and f) NOESY.

a)

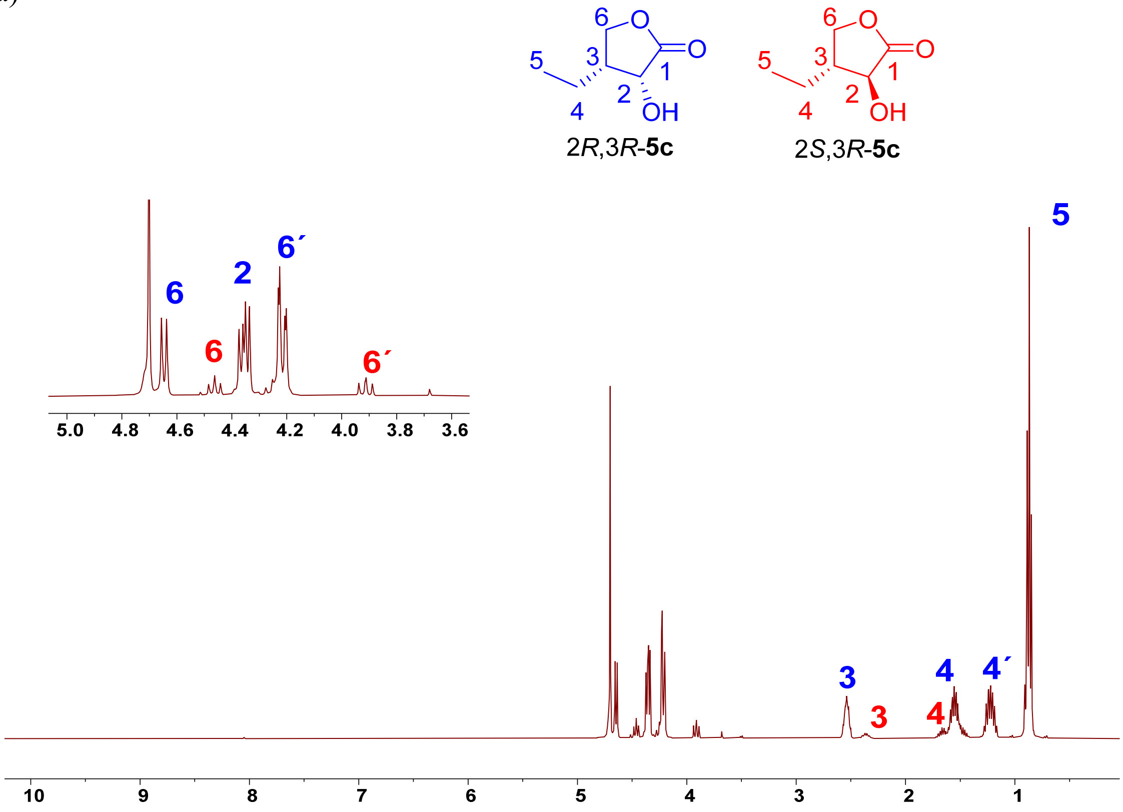

b)

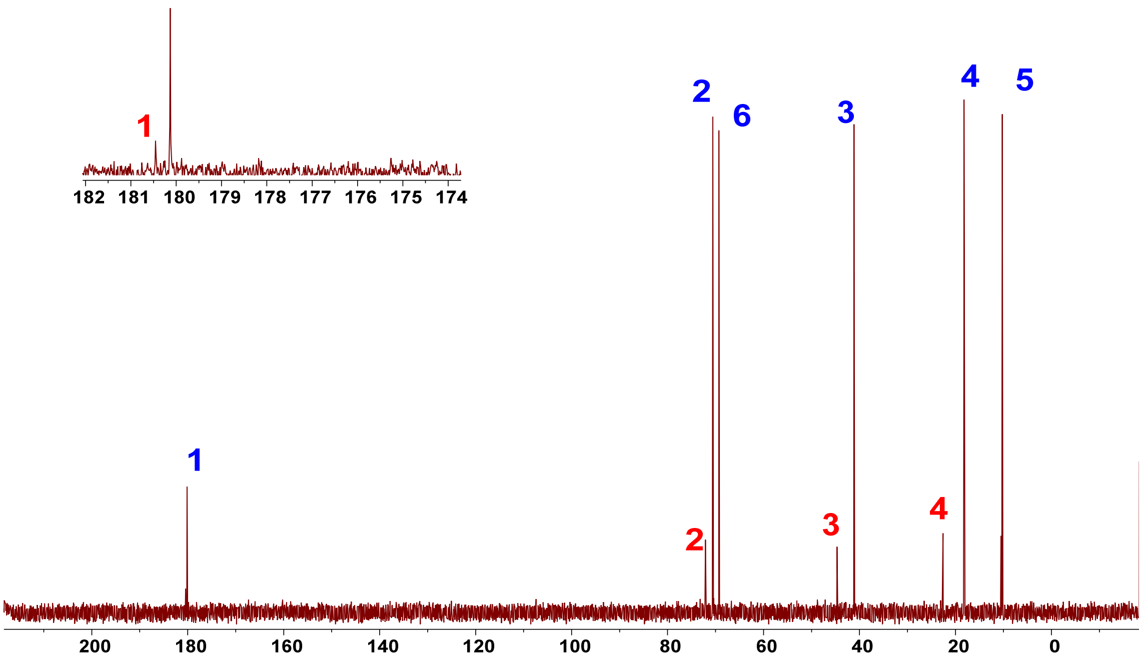
1
182 181 180 179 178 177 176 175 174
2
6
3
4
5
1
2
3
4
200 180 160 140 120 100 80 60 40 20 0

c)

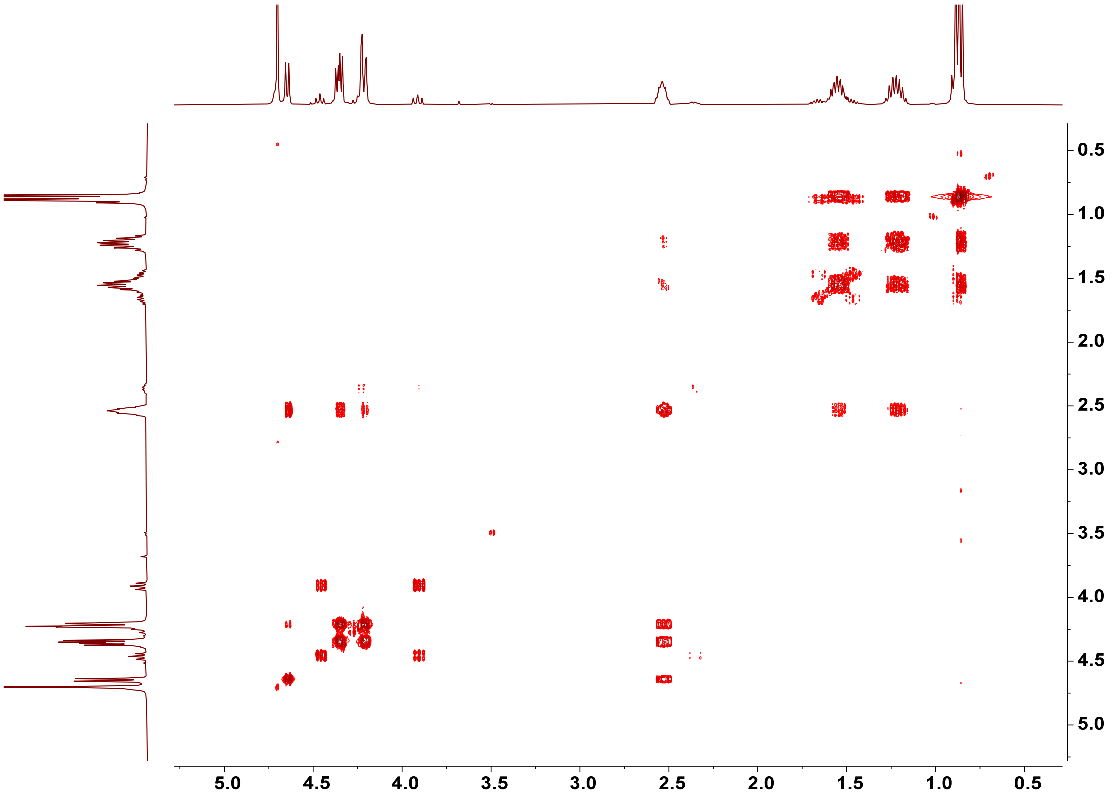
0.5
1.0
1.5
2.0
2.5
3.0
3.5
4.0
4.5
5.0
5.0 4.5 4.0 3.5 3.0 2.5 2.0 1.5 1.0 0.5

d)

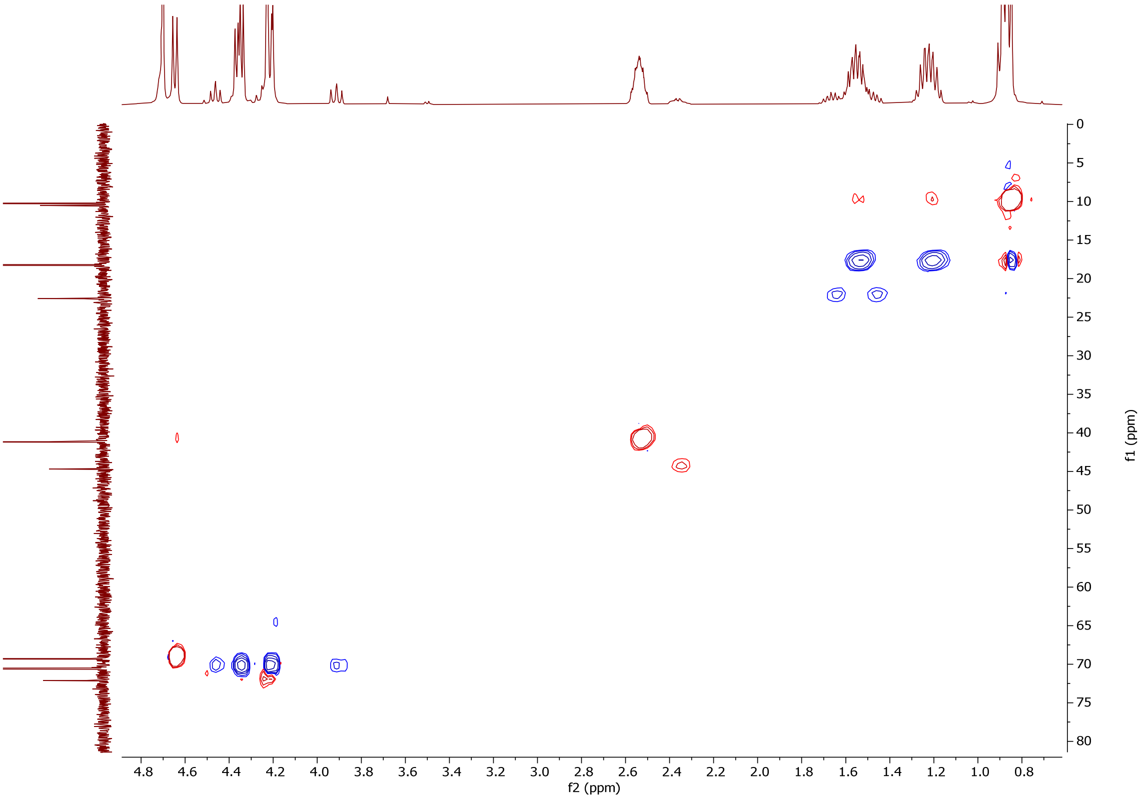
4.8 4.6 4.4 4.2 4.0 3.8 3.6 3.4 3.2 3.0 2.8 2.6 2.4 2.2 2.0 1.8 1.6 1.4 1.2 1.0 0.8
f2 (ppm)
0 5 10 15 20 25 30 35 40 45 50 55 60 65 70 75 80
f1 (ppm)

e)

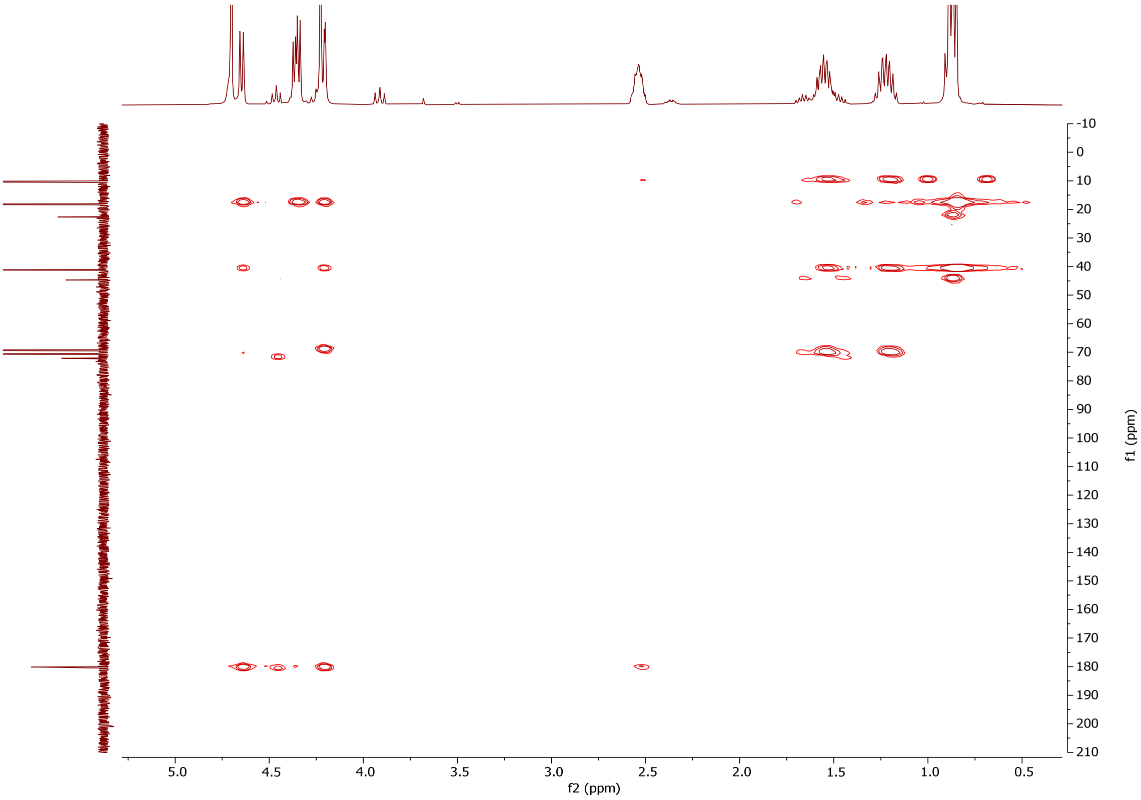
5.0 4.5 4.0 3.5 3.0 2.5 2.0 1.5 1.0 0.5
f2 (ppm)
-10 0 10 20 30 40 50 60 70 80 90 100 110 120 130 140 150 160 170 180 190 200 210
f1 (ppm)

f)

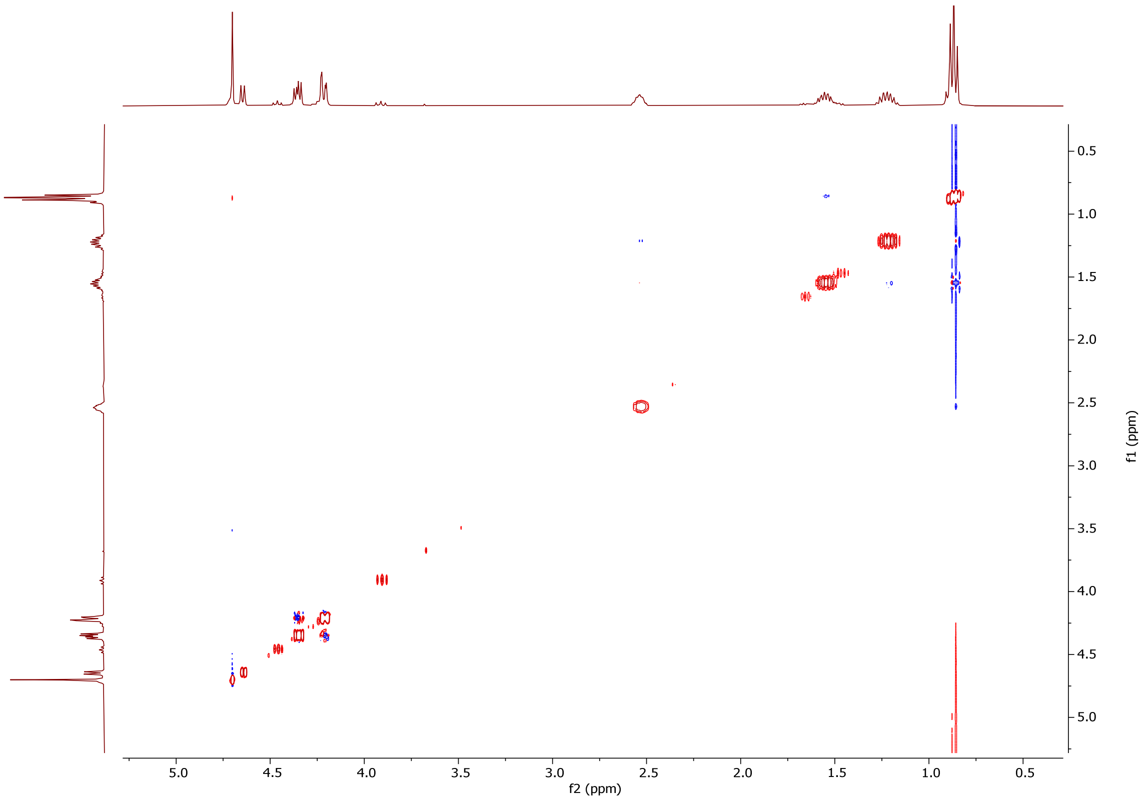

Figure S44. NMR spectra (D_2O) of 2*R*,3*R*-**5c**: a) ^{1}H, b) ^{13}C, c) COSY, d) HSQC, e) HMBC and f) NOESY.

a)

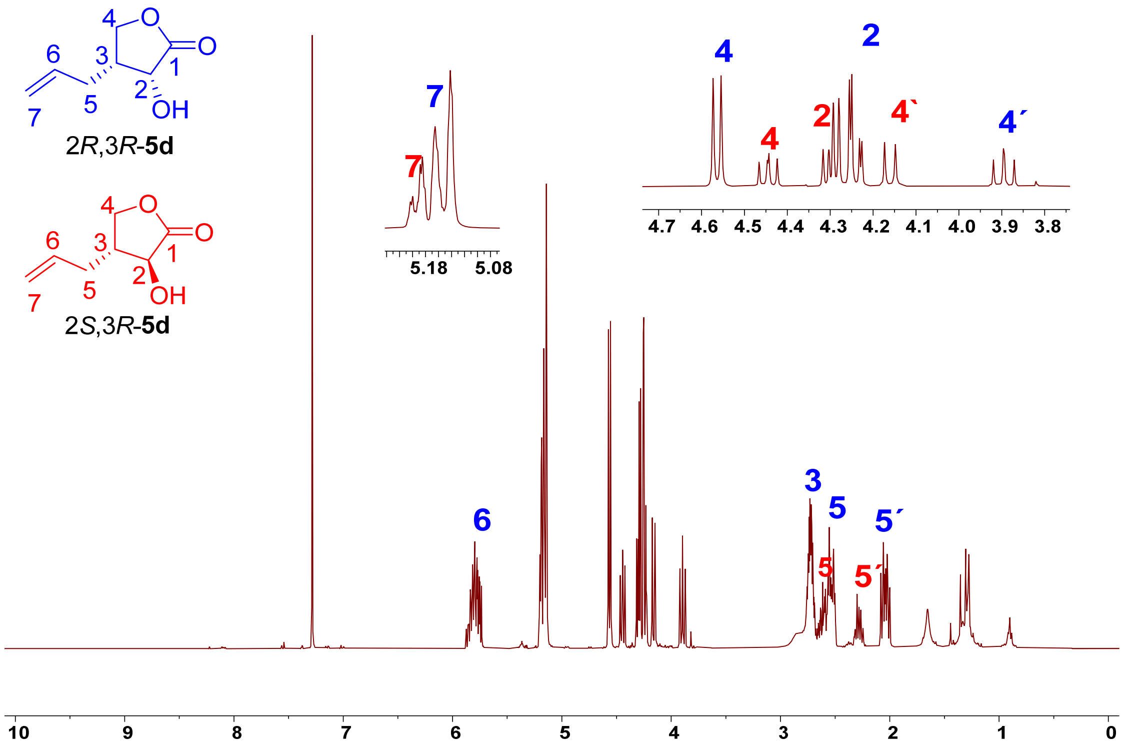

b)

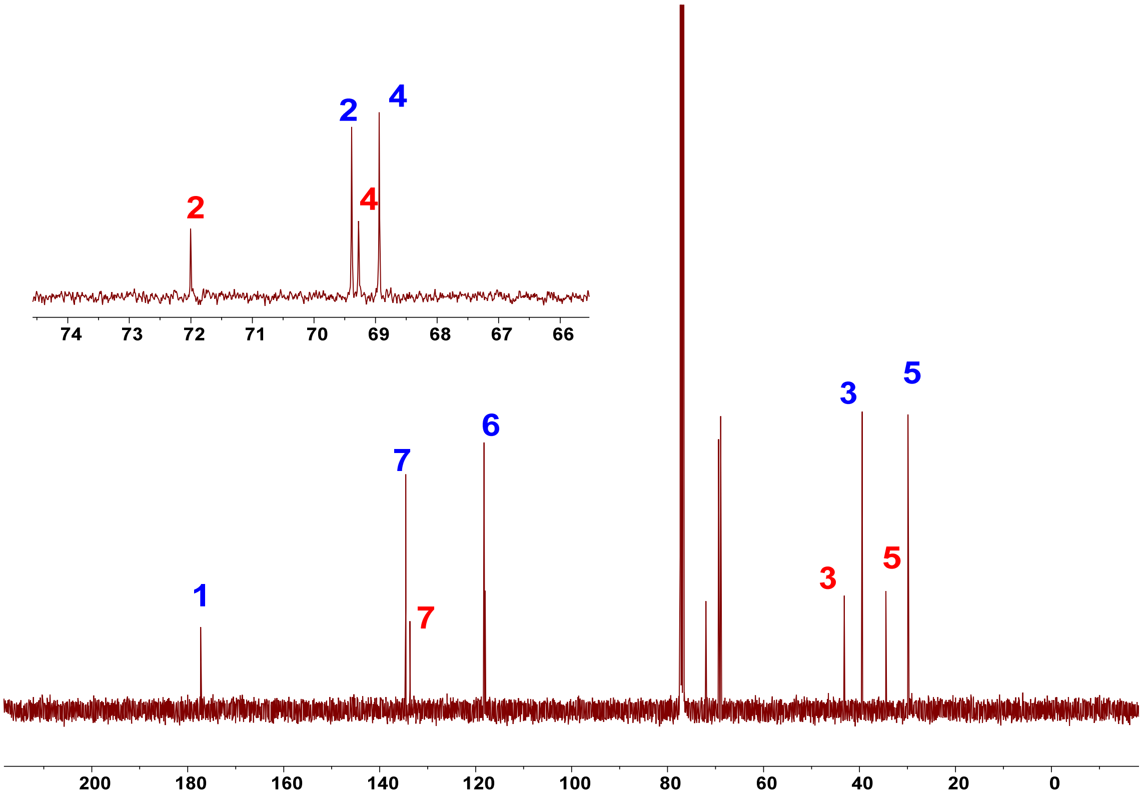
2
4
2
4
74
73
72
71
70
69
68
67
66
1
7
7
6
3
5
3
5
200
180
160
140
120
100
80
60
40
20
0

c)

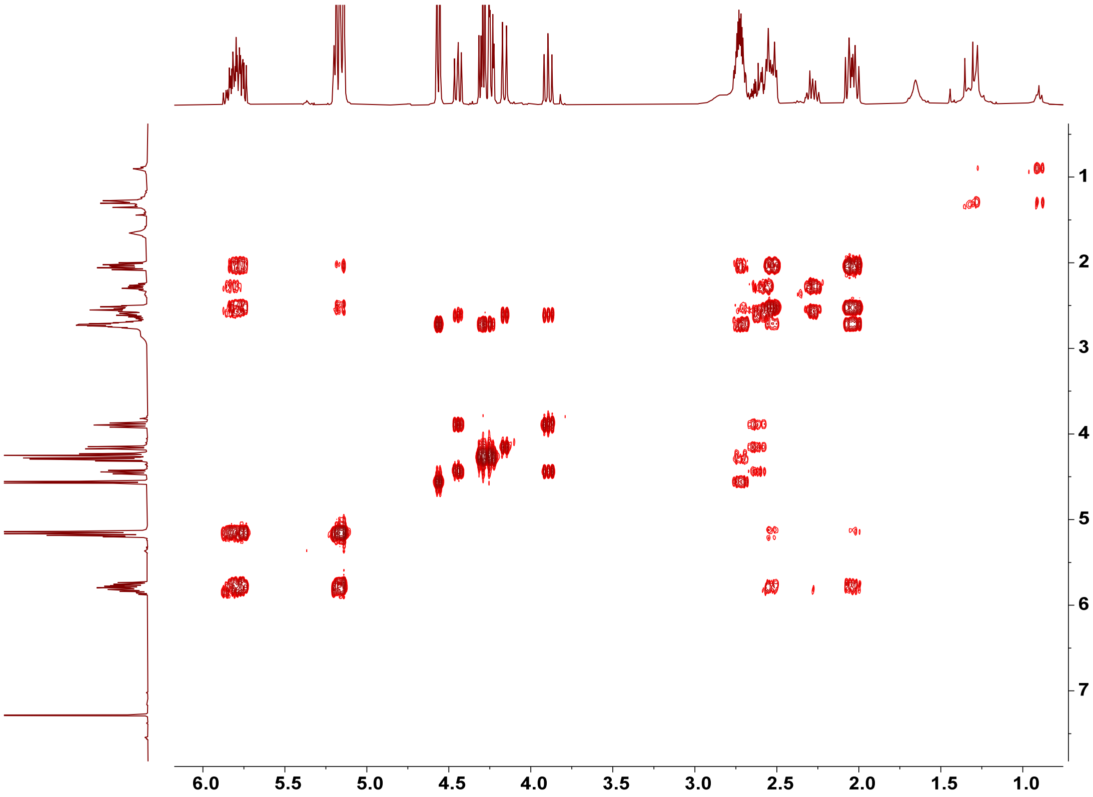
1
2
3
4
5
6
7
6.0
5.5
5.0
4.5
4.0
3.5
3.0
2.5
2.0
1.5
1.0

d)

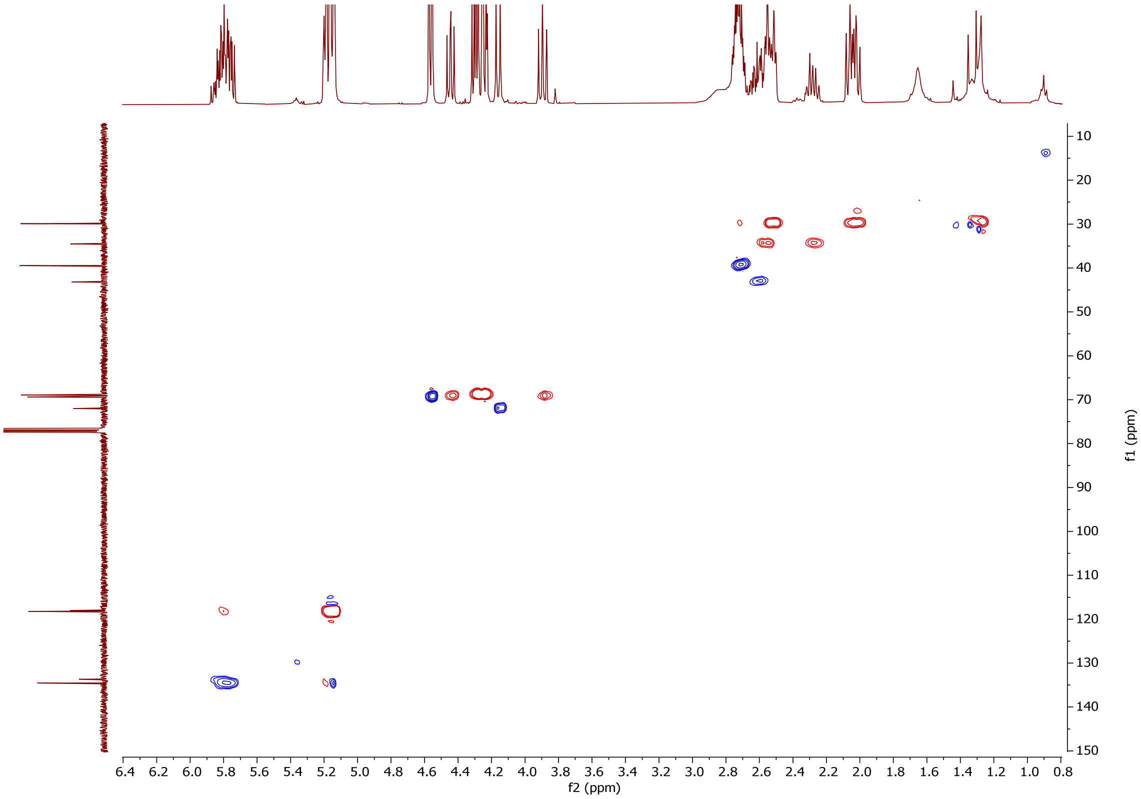
6.4 6.2 6.0 5.8 5.6 5.4 5.2 5.0 4.8 4.6 4.4 4.2 4.0 3.8 3.6 3.4 3.2 3.0 2.8 2.6 2.4 2.2 2.0 1.8 1.6 1.4 1.2 1.0 0.8
f2 (ppm)
10 20 30 40 50 60 70 80 90 100 110 120 130 140 150
f1 (ppm)

e)

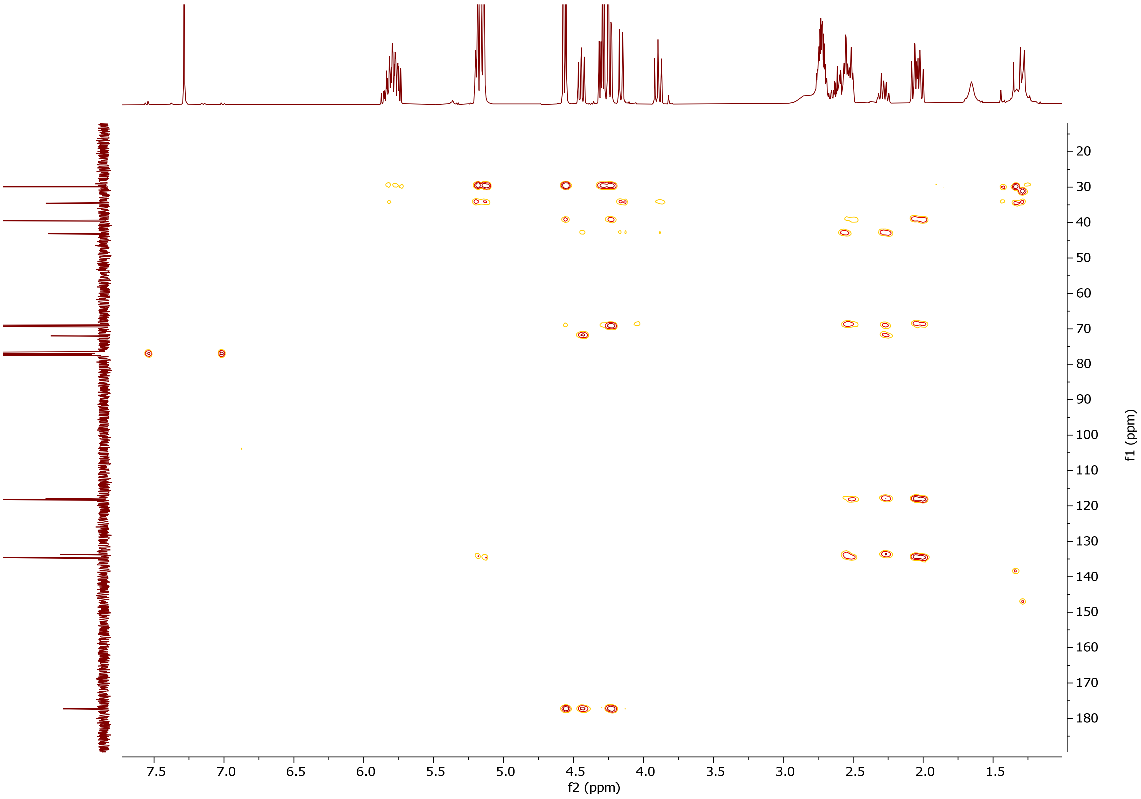
7.5 7.0 6.5 6.0 5.5 5.0 4.5 4.0 3.5 3.0 2.5 2.0 1.5
f2 (ppm)
20 30 40 50 60 70 80 90 100 110 120 130 140 150 160 170 180
f1 (ppm)

f)

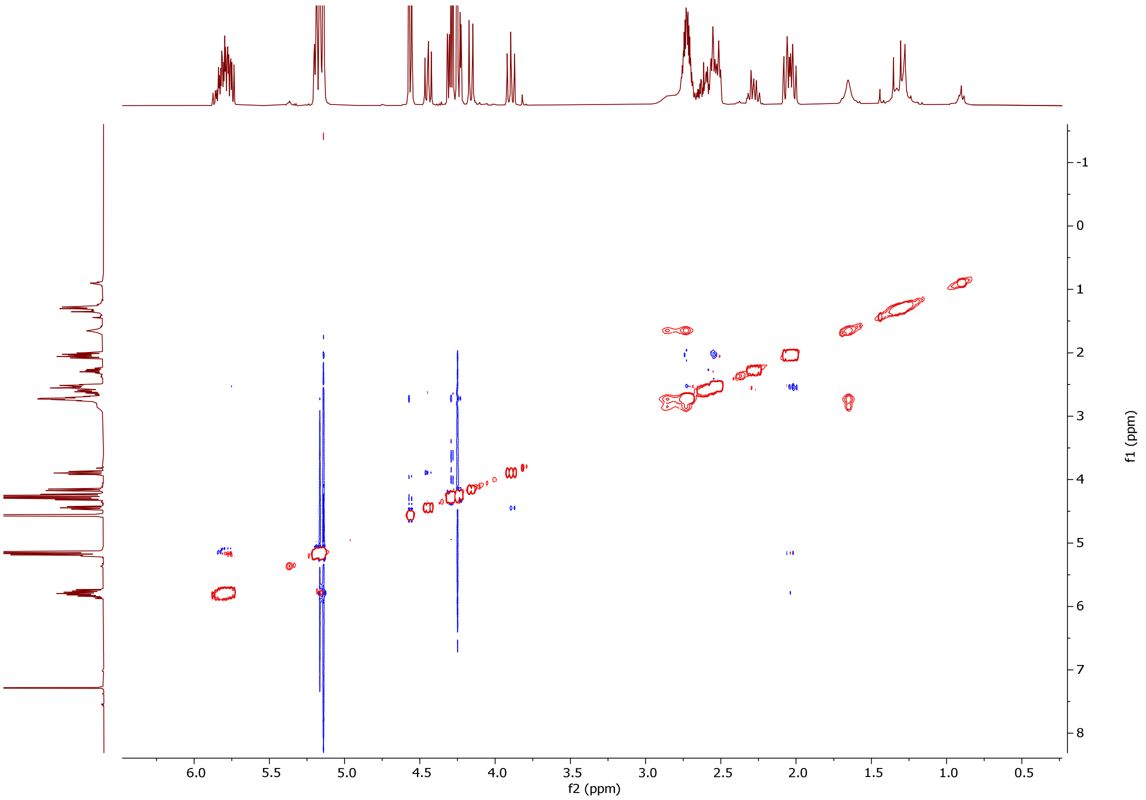

Figure S45. NMR spectra ($CDCl_3$) of 2*R*,3*R*-**5d**: a) ^{1}H, b) ^{13}C, c) COSY, d) HSQC, e) HMBC and f) NOESY.

a)

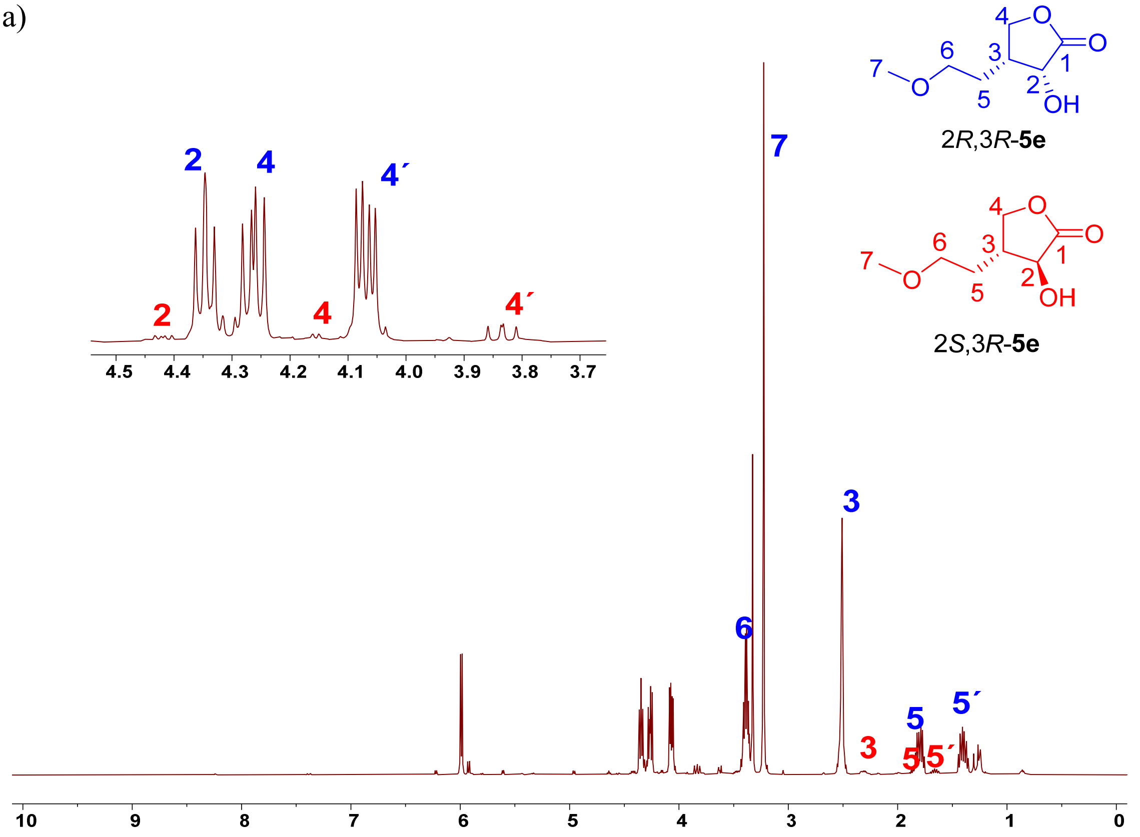

b)

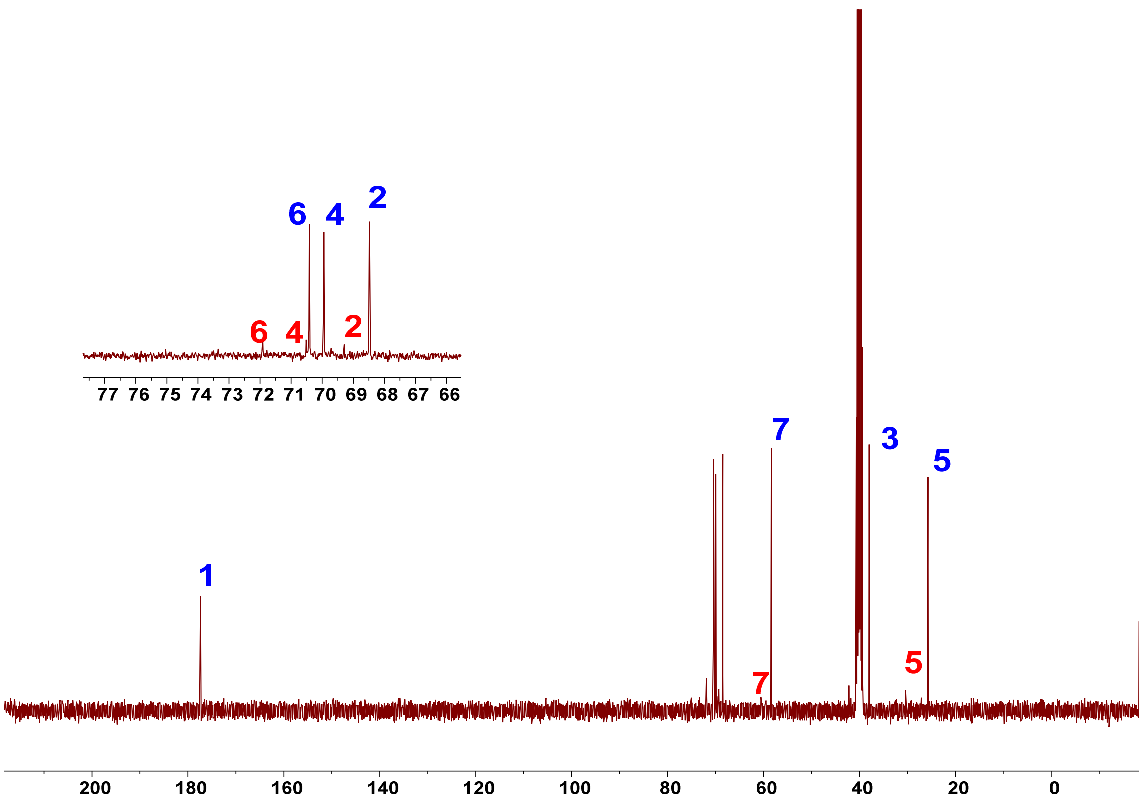
6
4
2
6
4
2
77 76 75 74 73 72 71 70 69 68 67 66
1
7
3
5
7
5
200
180
160
140
120
100
80
60
40
20
0

c)

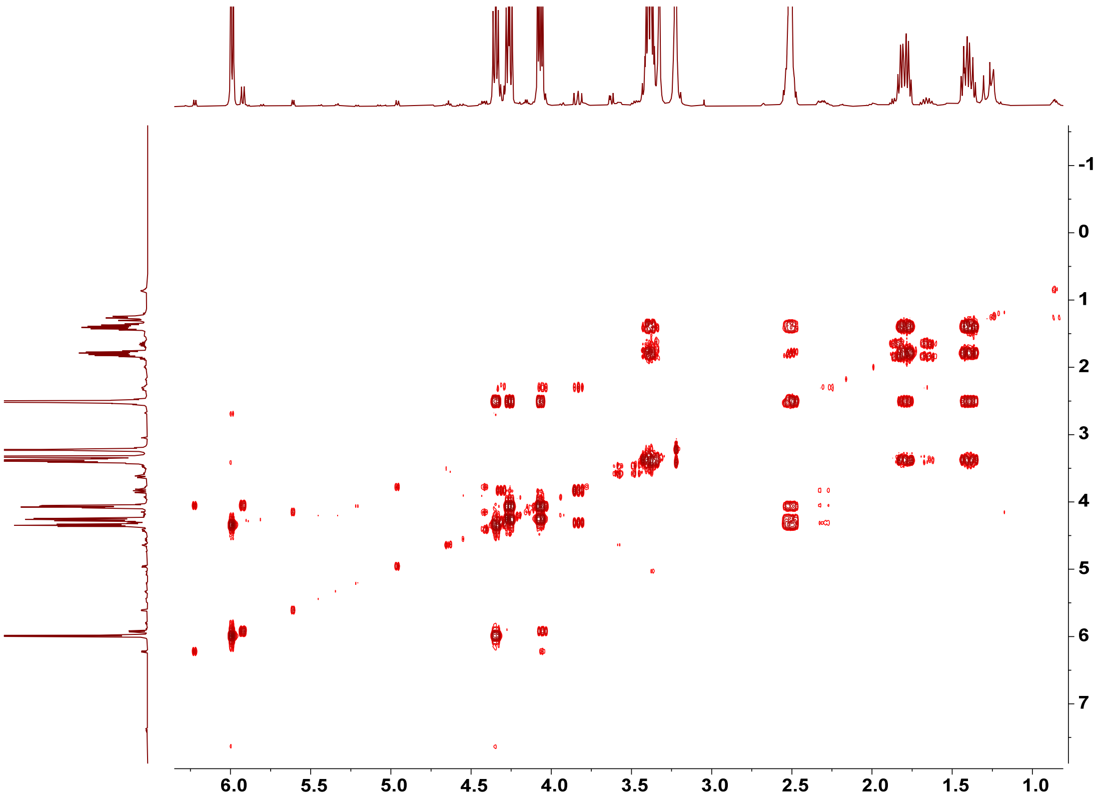
-1
0
1
2
3
4
5
6
7
6.0
5.5
5.0
4.5
4.0
3.5
3.0
2.5
2.0
1.5
1.0

d)

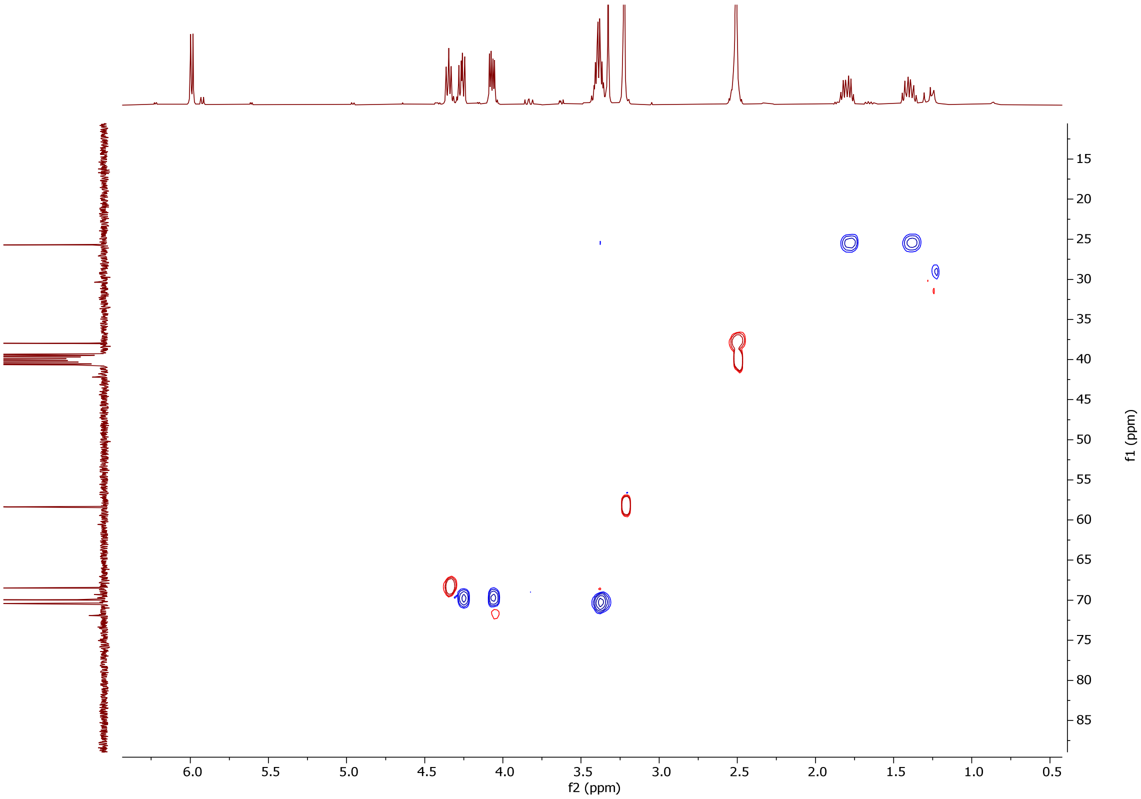

e)

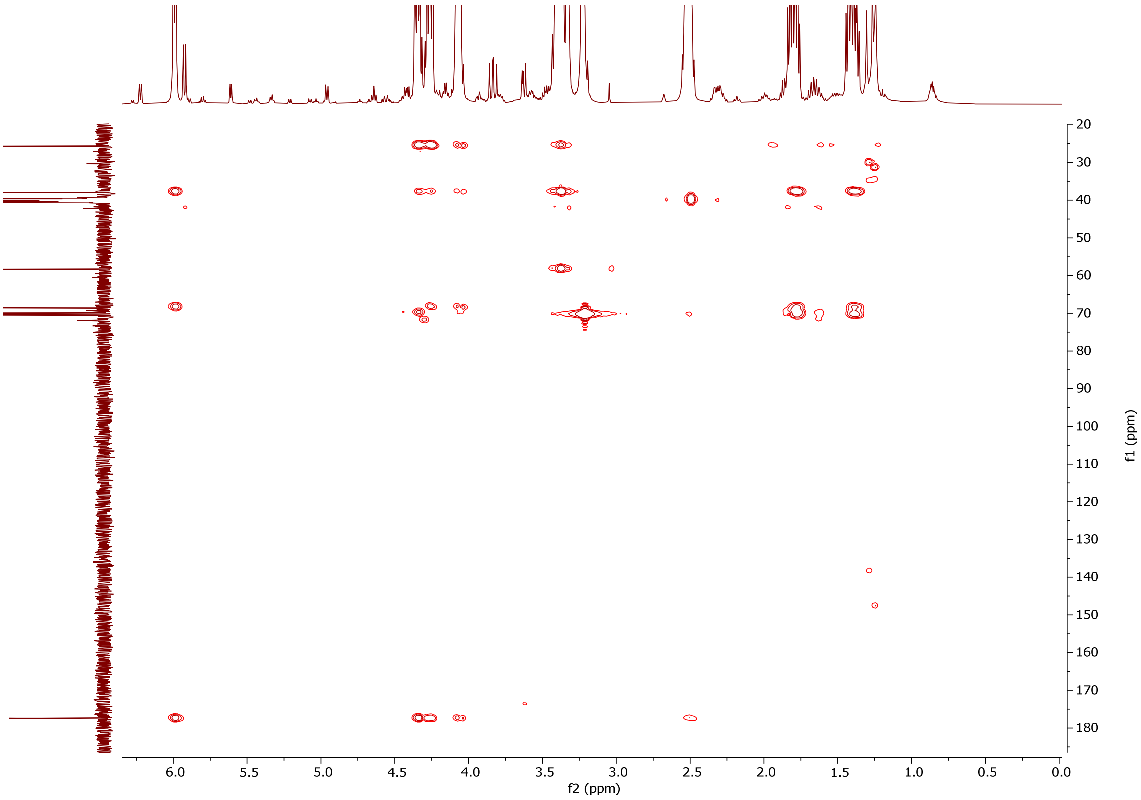

f)

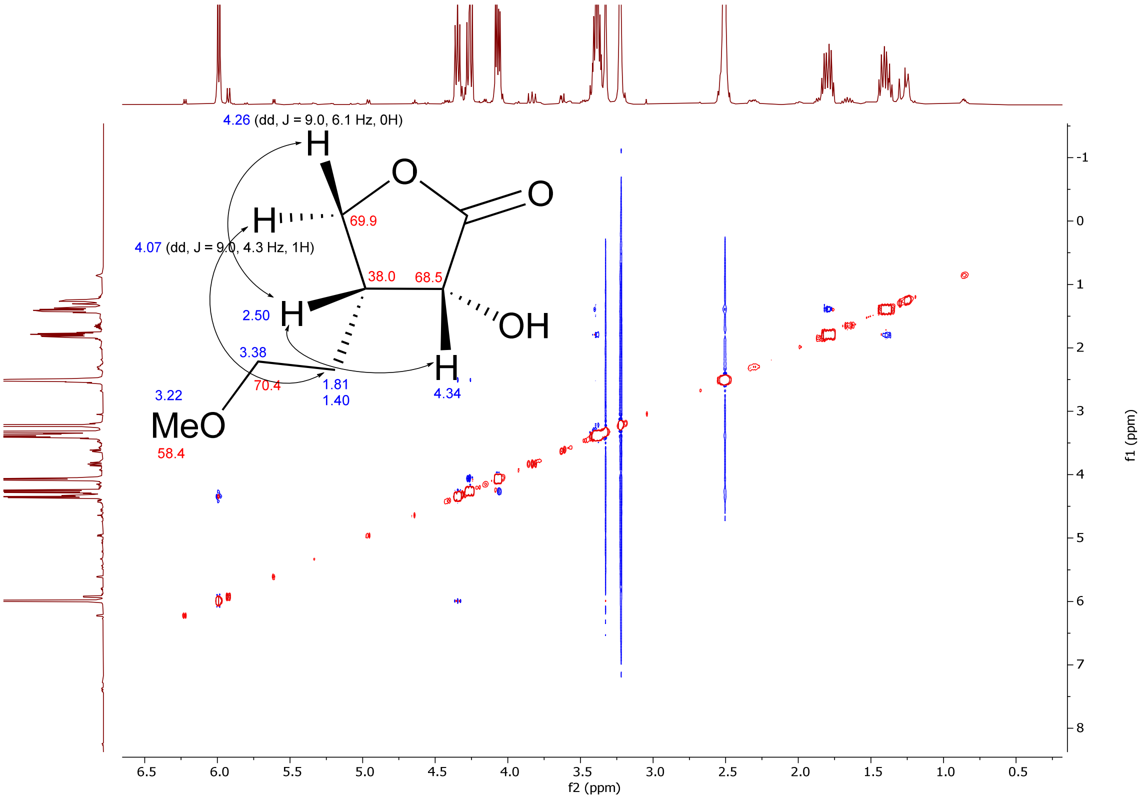

Figure S46. NMR spectra (DMSO) of 2*R*,3*R*-**5e**: a) ^{1}H, b) ^{13}C, c) COSY, d) HSQC, e) HMBC and f) NOESY.

a)

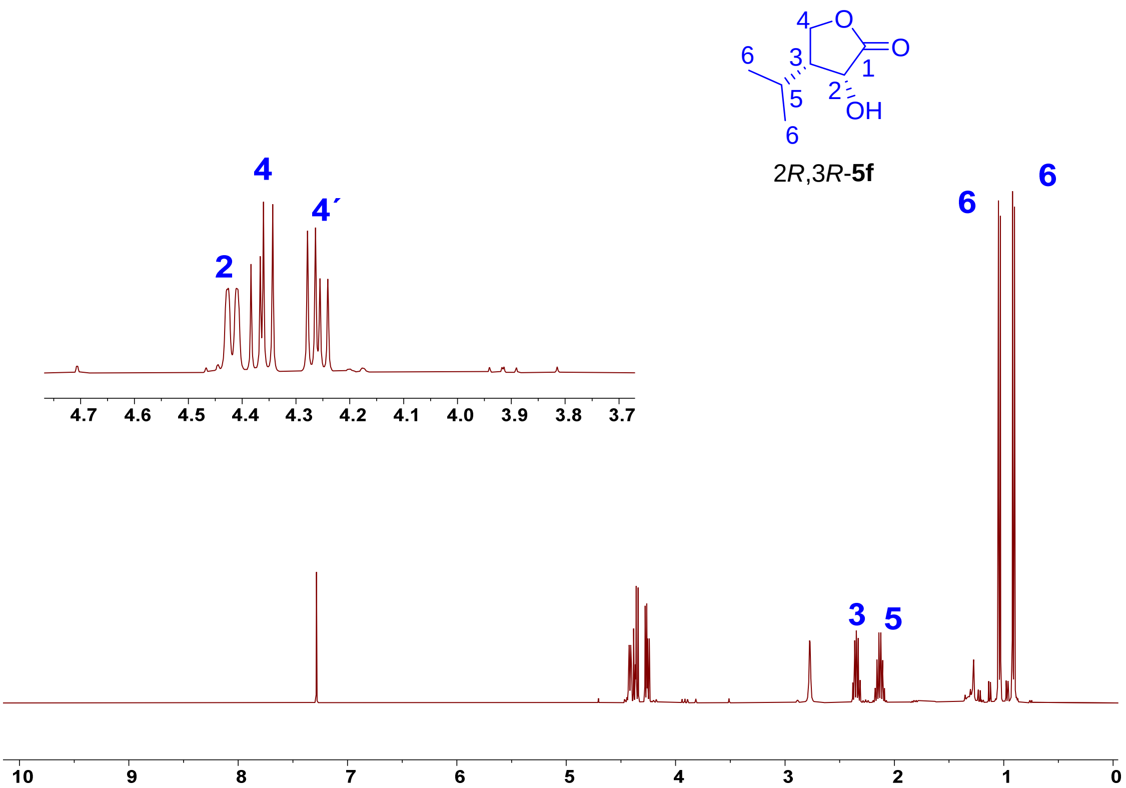

b)

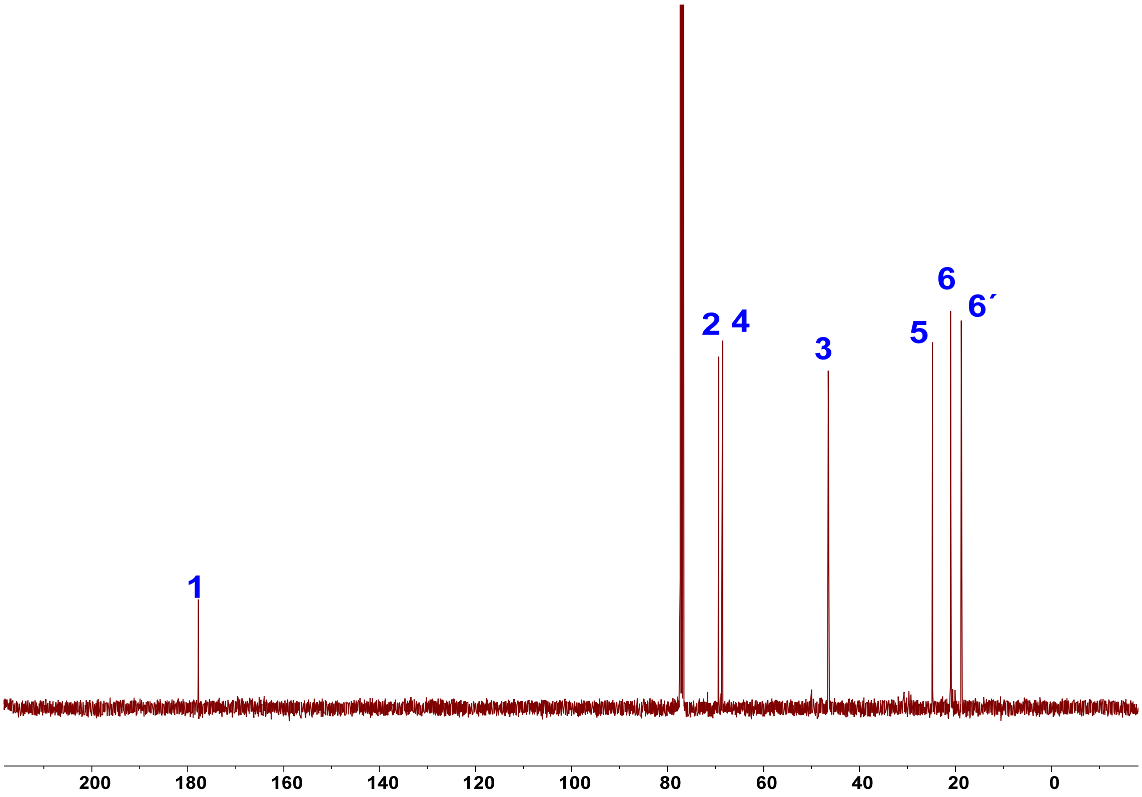

1
2
4
3
5
6
6´
200
180
160
140
120
100
80
60
40
20
0

c)

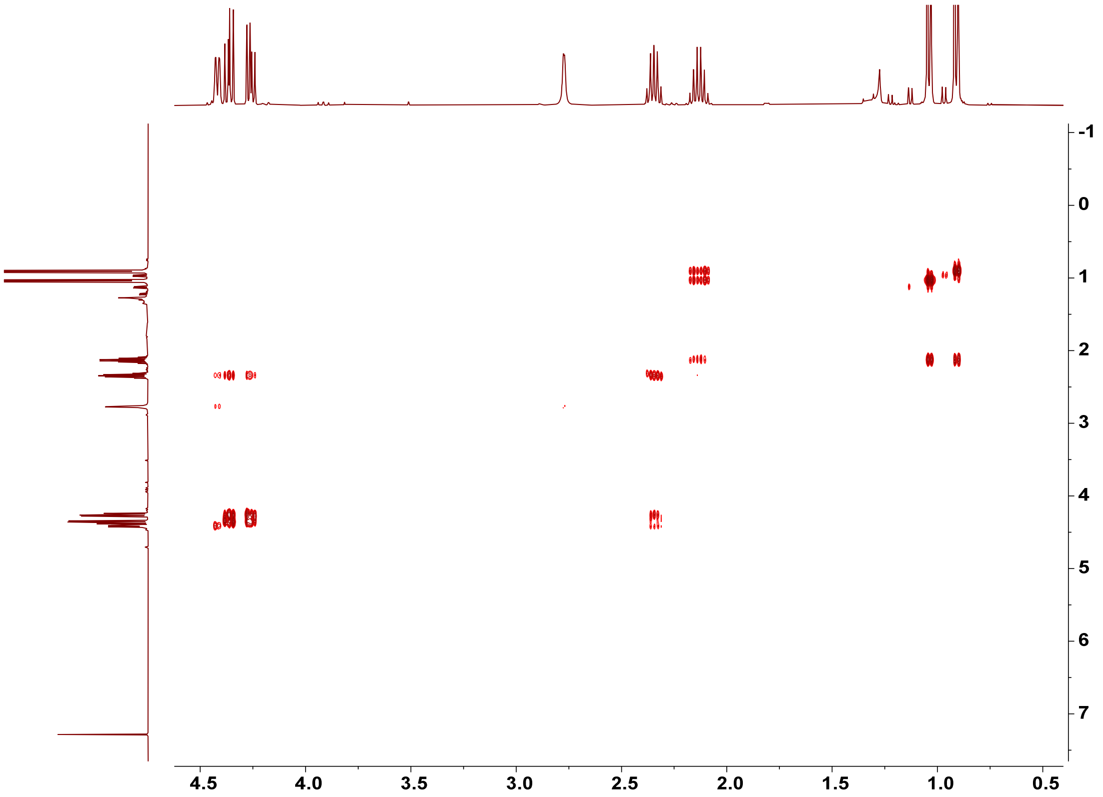

4.5
4.0
3.5
3.0
2.5
2.0
1.5
1.0
0.5
-1
0
1
2
3
4
5
6
7

d)

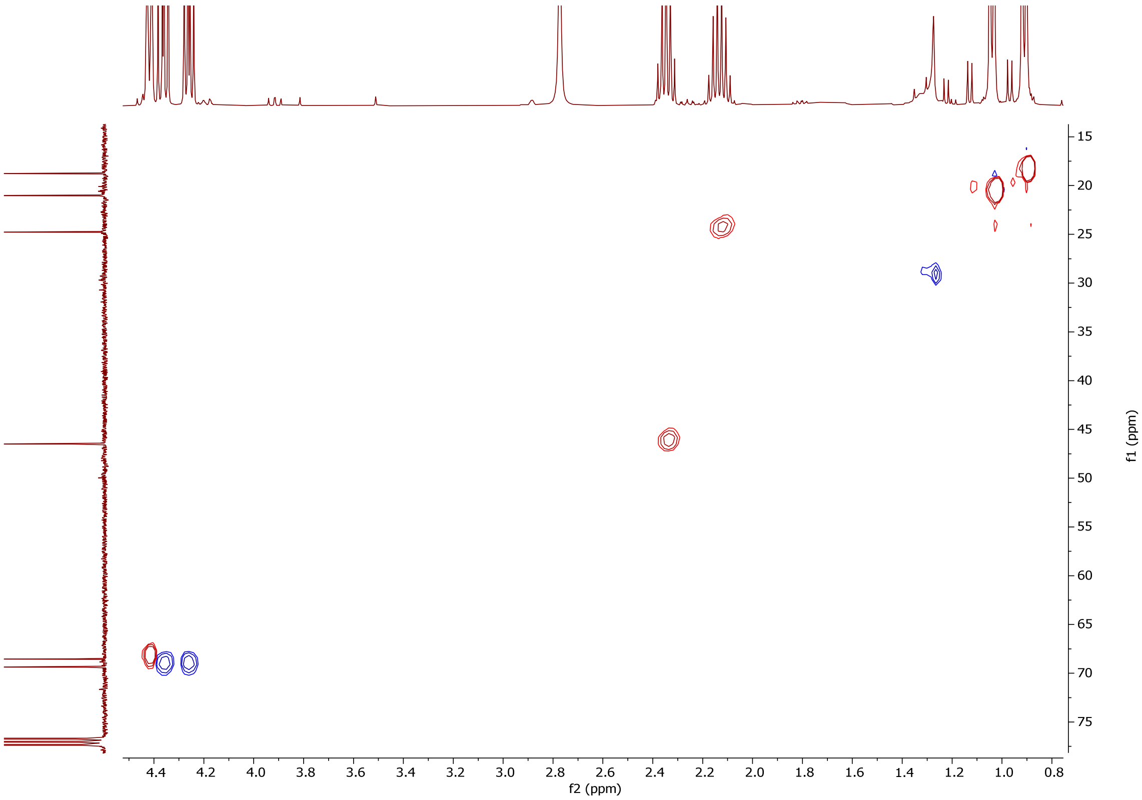

4.4
4.2
4.0
3.8
3.6
3.4
3.2
3.0
2.8
2.6
2.4
2.2
2.0
1.8
1.6
1.4
1.2
1.0
0.8
f2 (ppm)
15
20
25
30
35
40
45
50
55
60
65
70
75
f1 (ppm)

e)

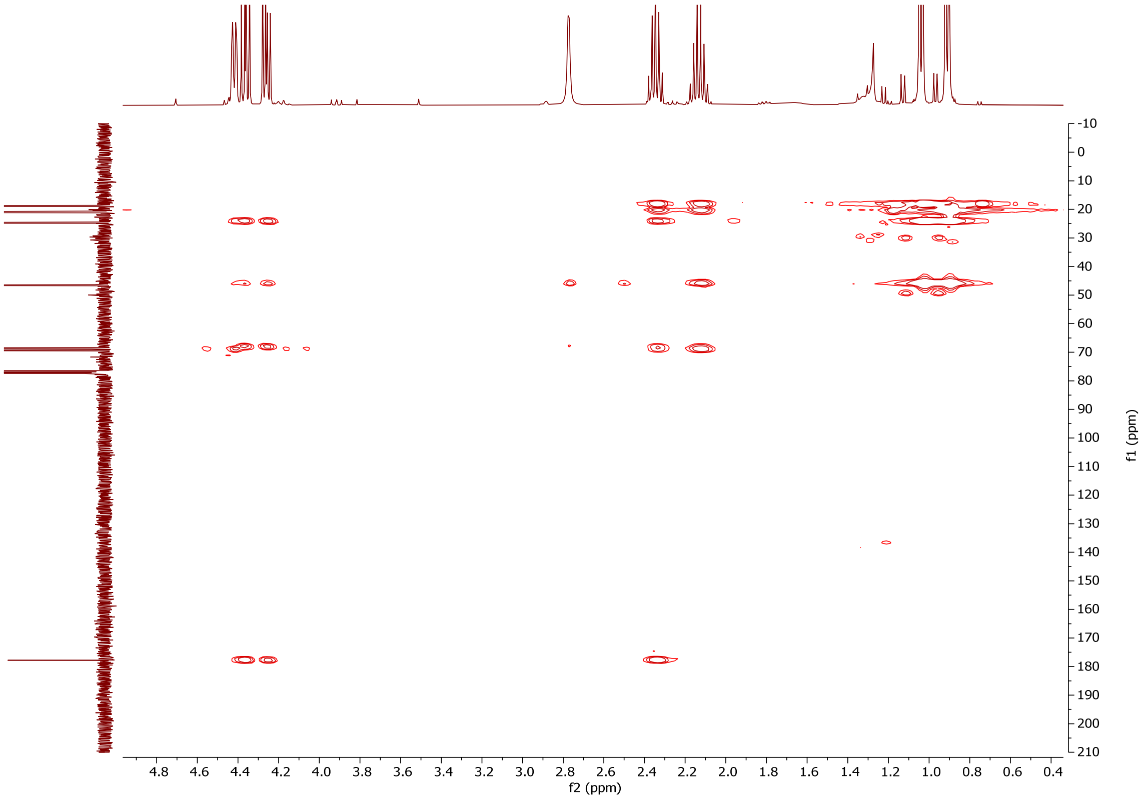

4.8
4.6
4.4
4.2
4.0
3.8
3.6
3.4
3.2
3.0
2.8
2.6
2.4
2.2
2.0
1.8
1.6
1.4
1.2
1.0
0.8
0.6
0.4
f2 (ppm)
-10
0
10
20
30
40
50
60
70
80
90
100
110
120
130
140
150
160
170
180
190
200
210
f1 (ppm)

f)

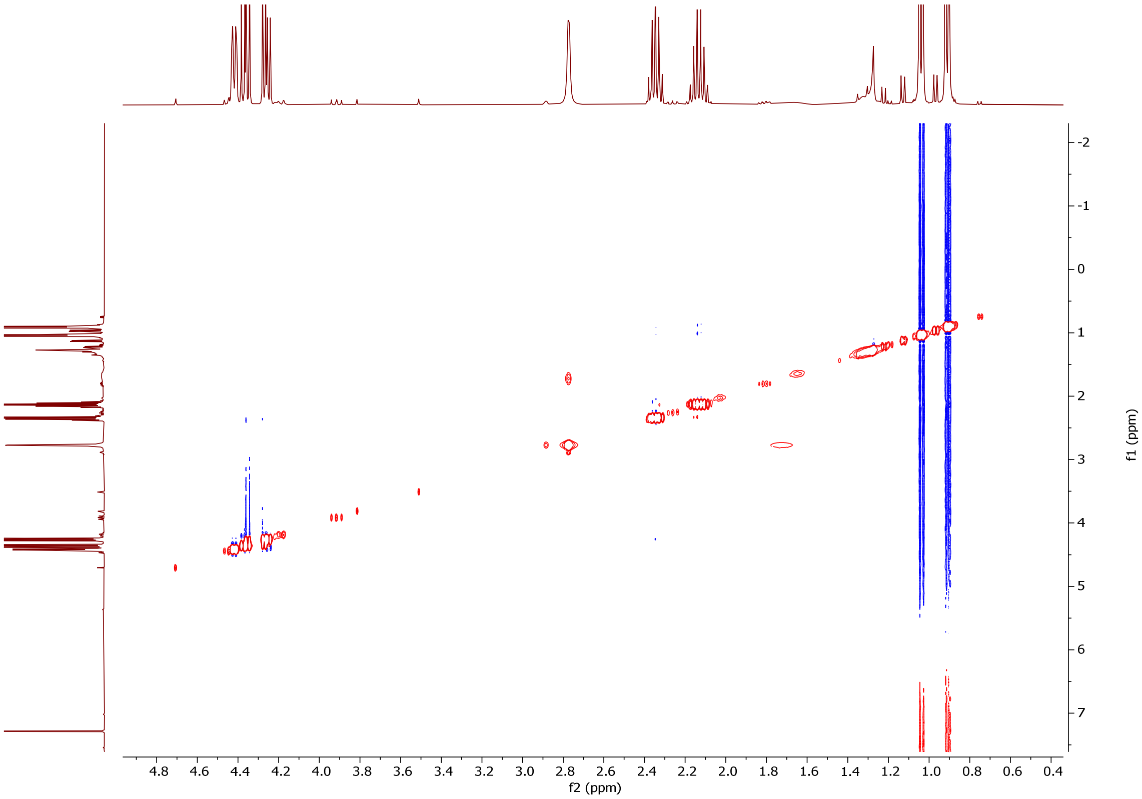

Figure S47. NMR spectra ($CDCl_3$) of 2*R*,3*R*-**5f**: a) ^1H, b) ^{13}C, c) COSY, d) HSQC, e) HMBC and f) NOESY.

a)

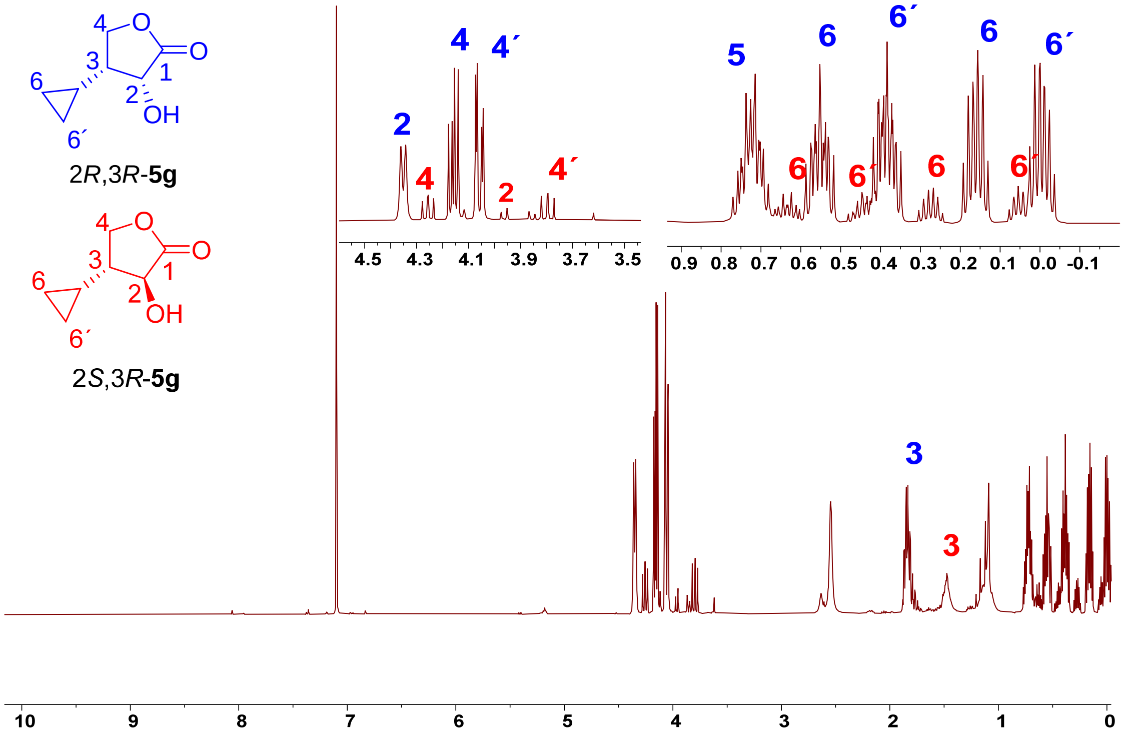

b)

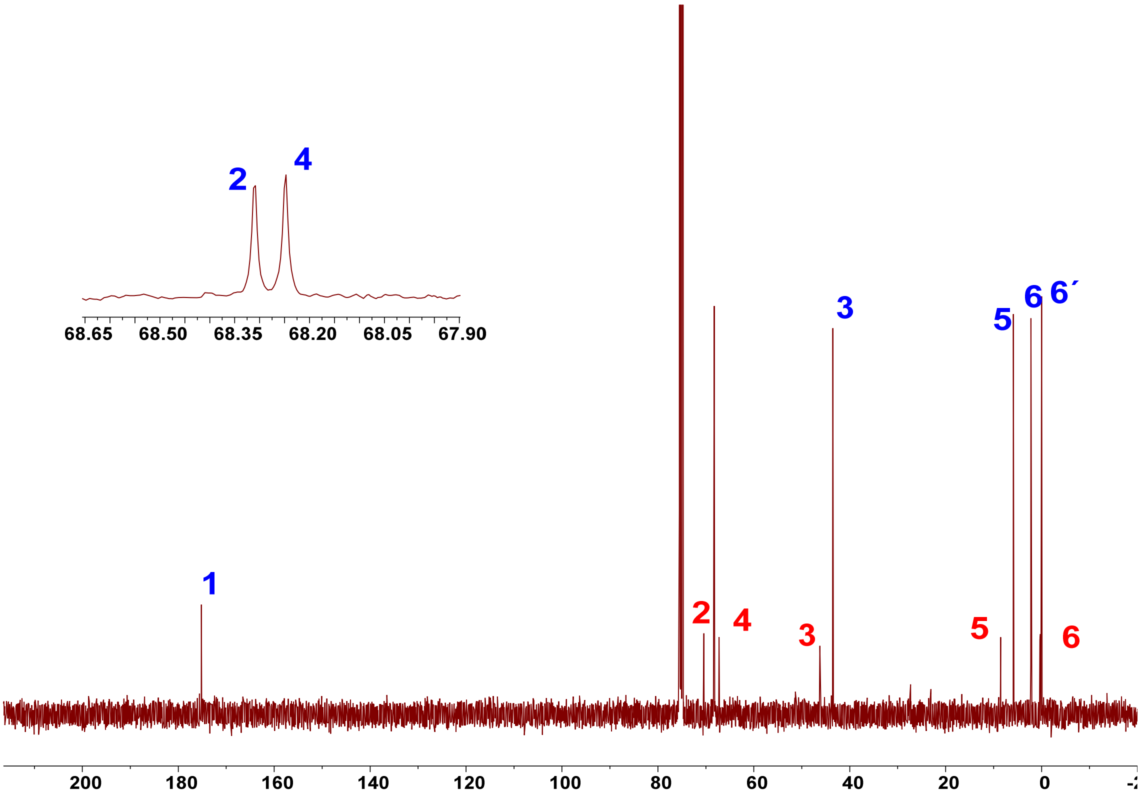
2
4
68.65
68.50
68.35
68.20
68.05
67.90
1
2
4
3
3
5
6
6´
5
6
200
180
160
140
120
100
80
60
40
20
0

c)

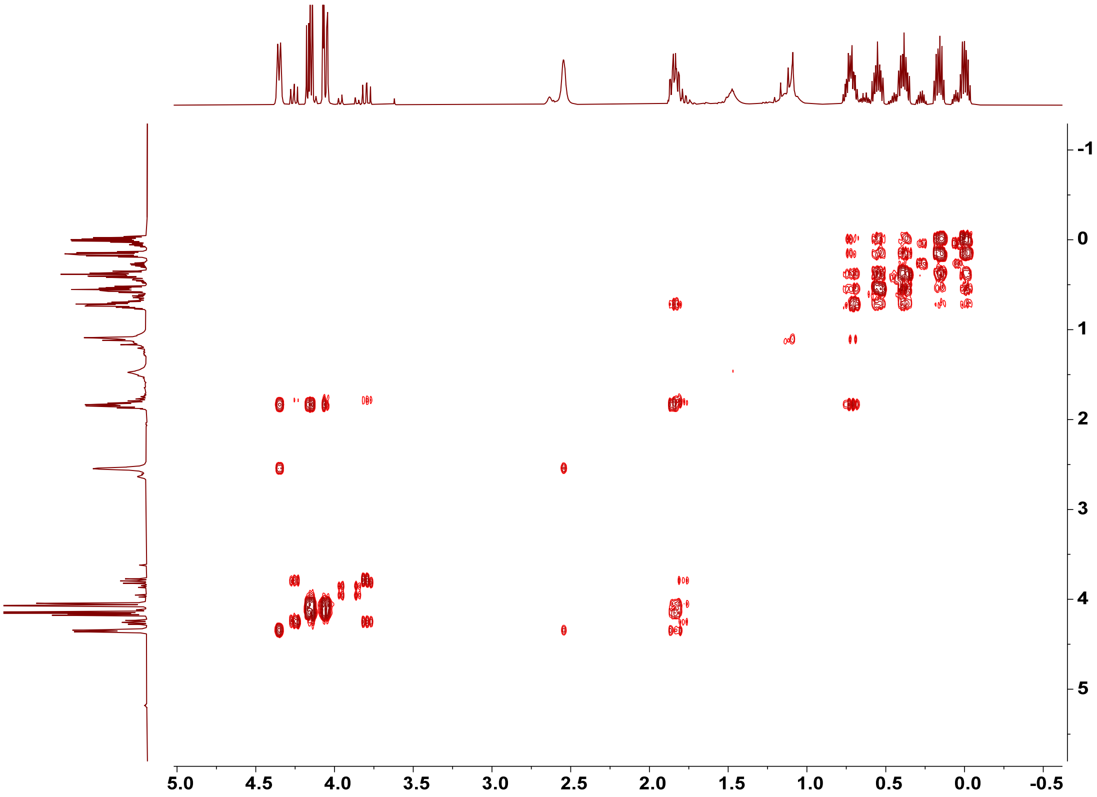
-1
0
1
2
3
4
5
5.0
4.5
4.0
3.5
3.0
2.5
2.0
1.5
1.0
0.5
0.0
-0.5

d)

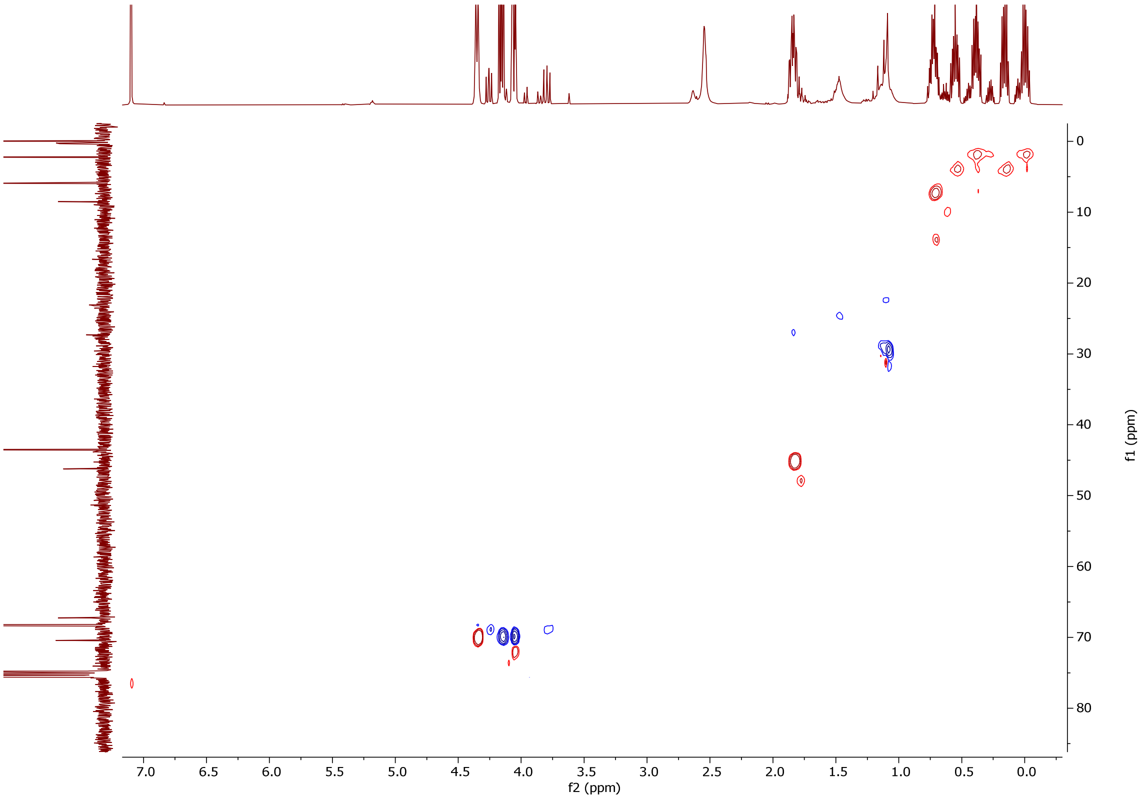

7.0
6.5
6.0
5.5
5.0
4.5
4.0
3.5
3.0
2.5
2.0
1.5
1.0
0.5
0.0
f2 (ppm)
0
10
20
30
40
50
60
70
80
f1 (ppm)

e)

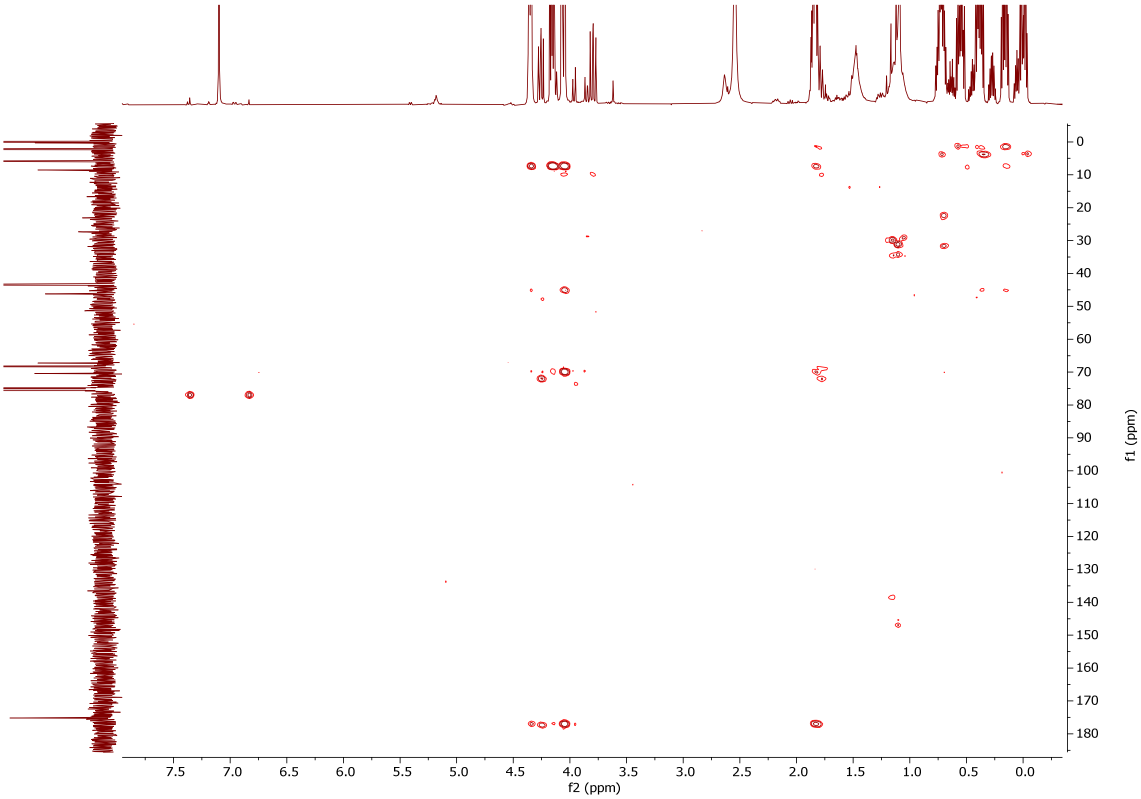

7.5
7.0
6.5
6.0
5.5
5.0
4.5
4.0
3.5
3.0
2.5
2.0
1.5
1.0
0.5
0.0
f2 (ppm)
0
10
20
30
40
50
60
70
80
90
100
110
120
130
140
150
160
170
180
f1 (ppm)

f)

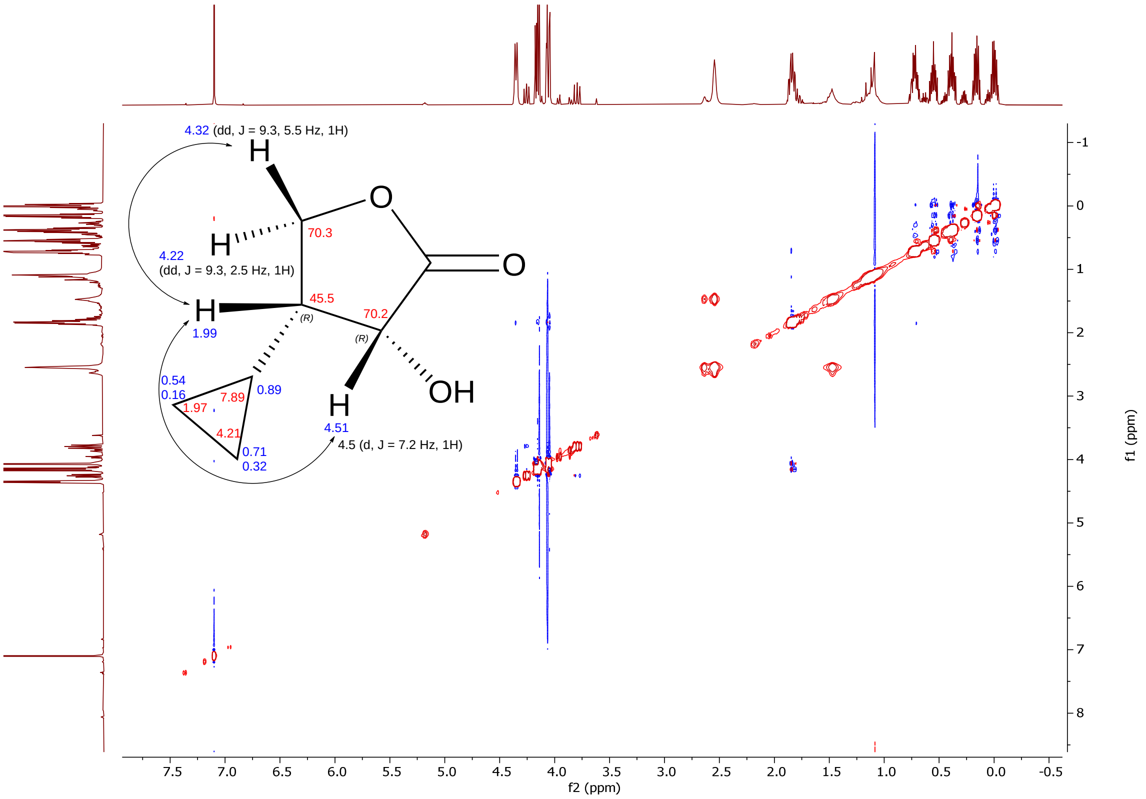

Figure S48. NMR spectra ($CDCl_3$) of 2*R*,3*R*-**5g**: a) ^1H, b) ^{13}C, c) COSY, d) HSQC, e) HMBC and f) NOESY.

a)

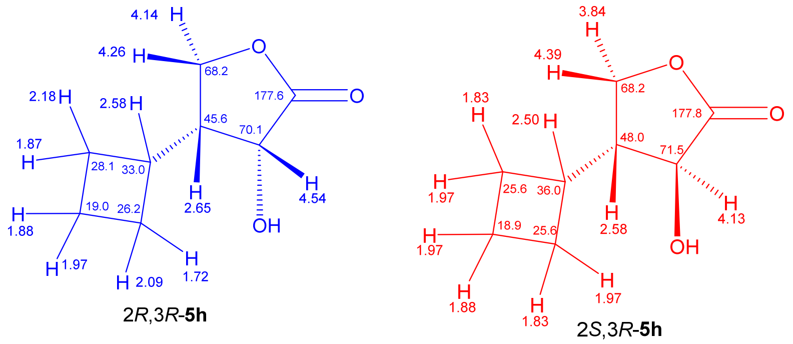

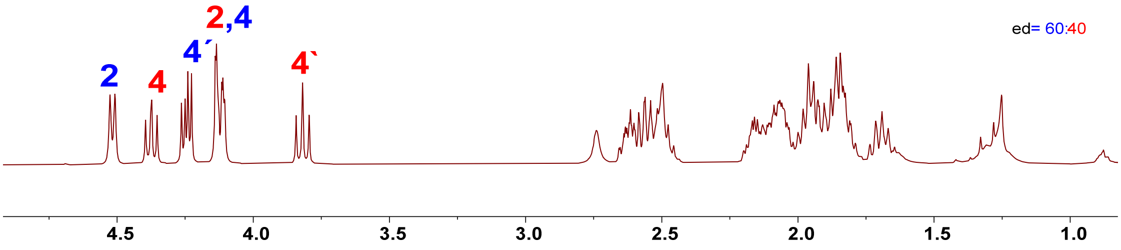

b)

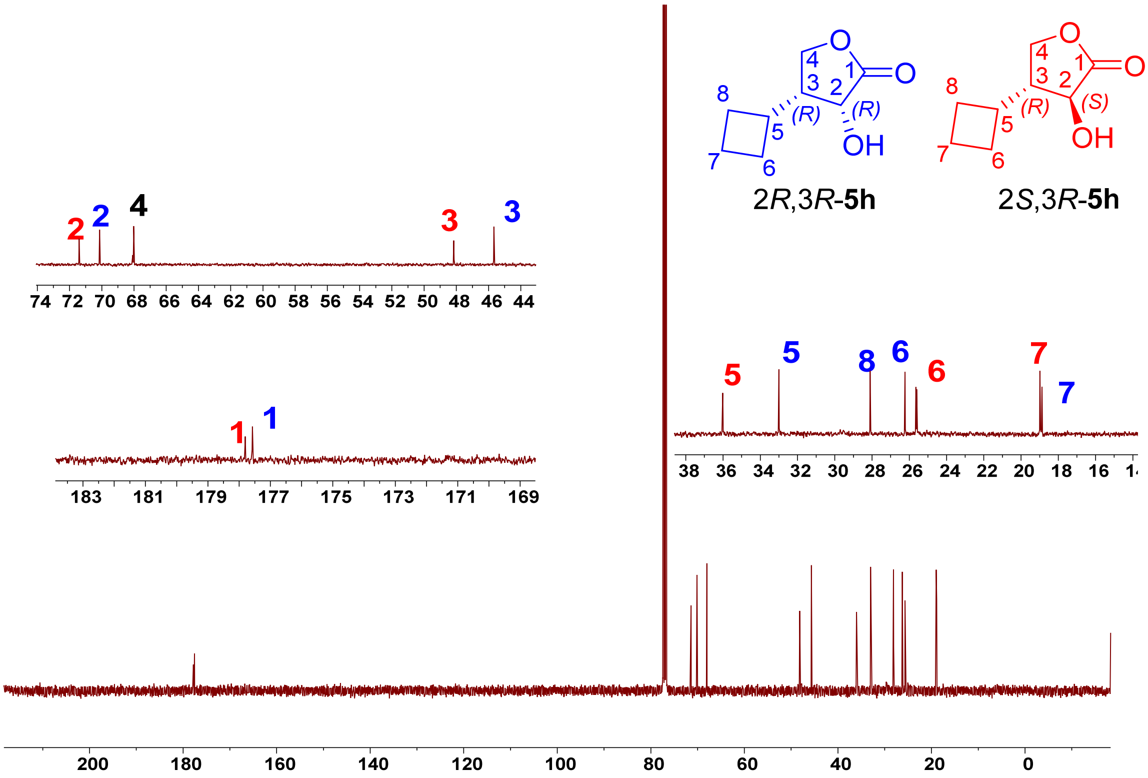

c)

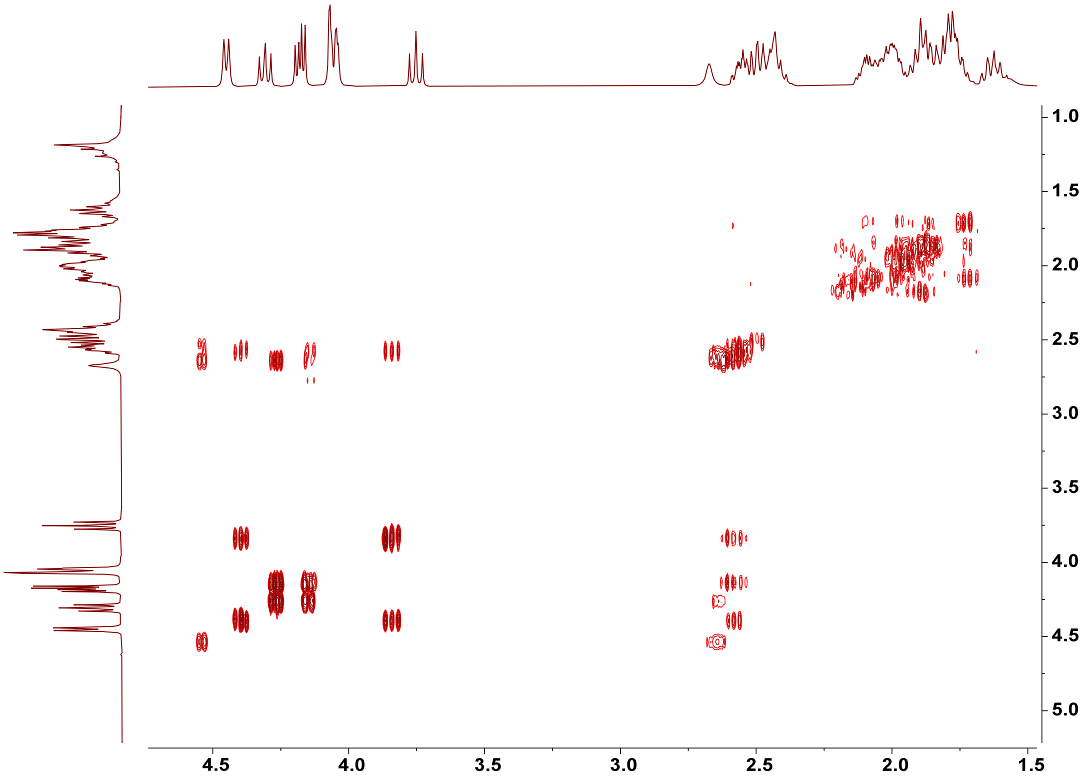

d)

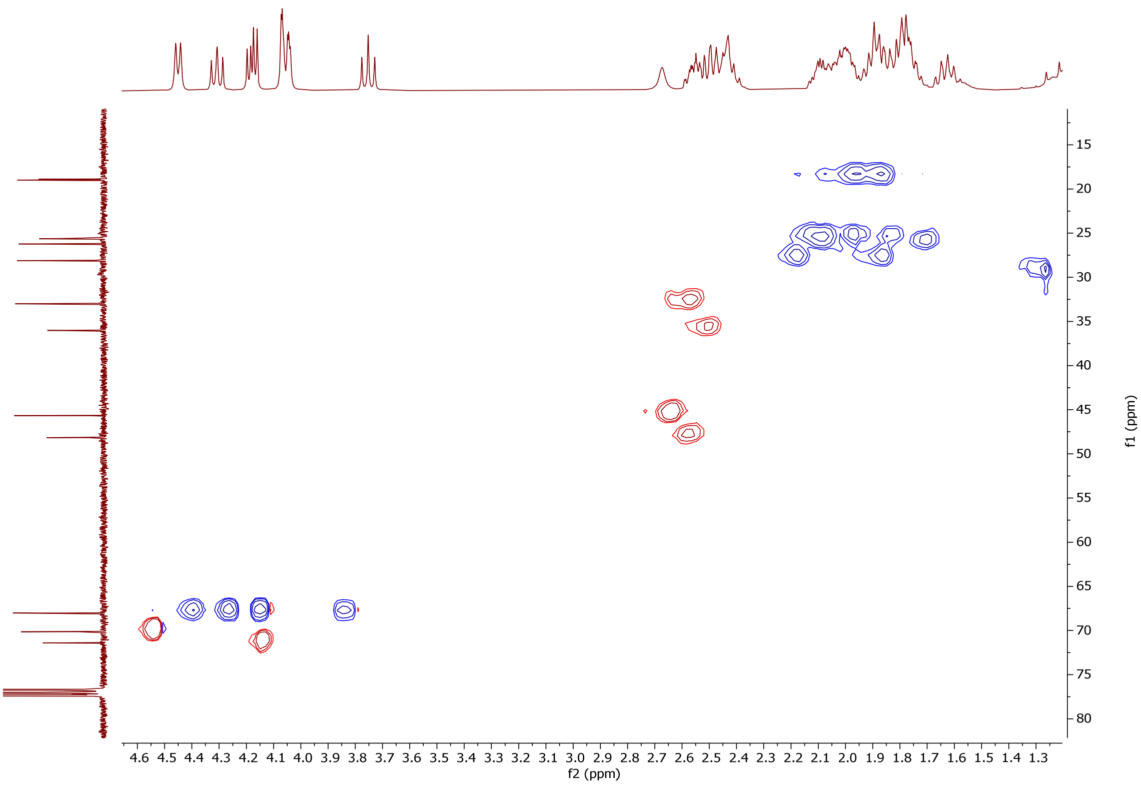

e)

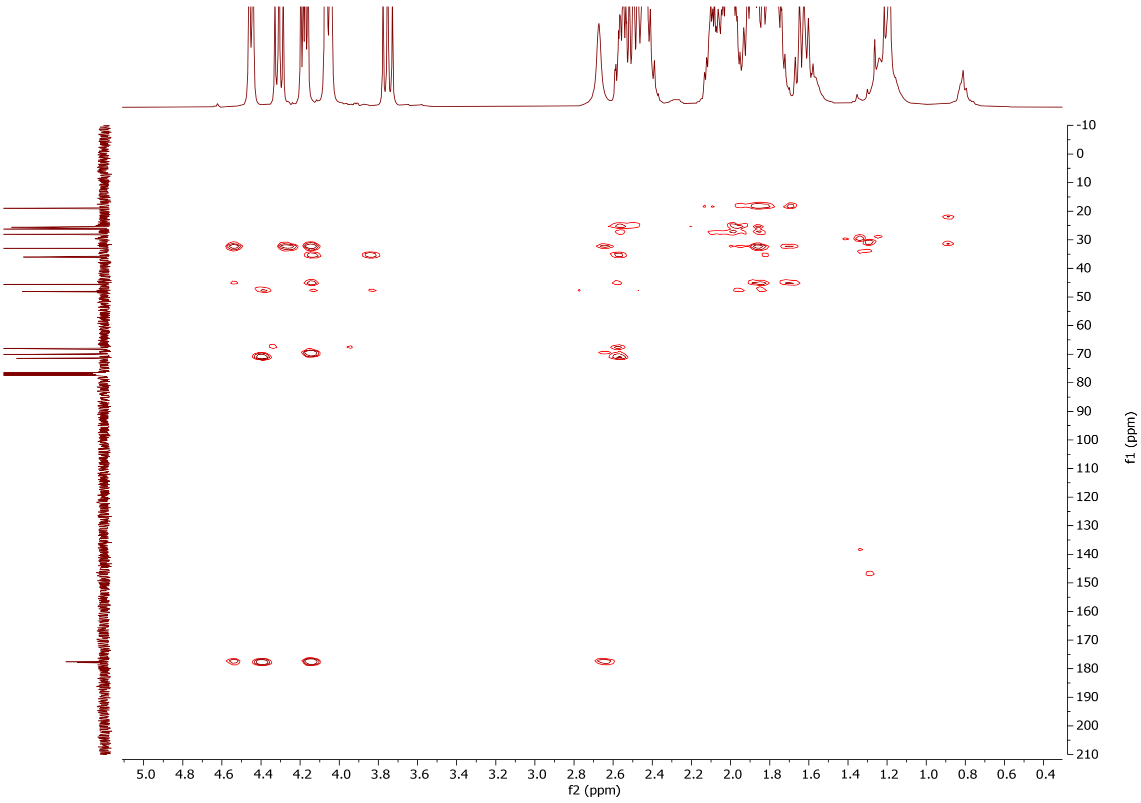

f)

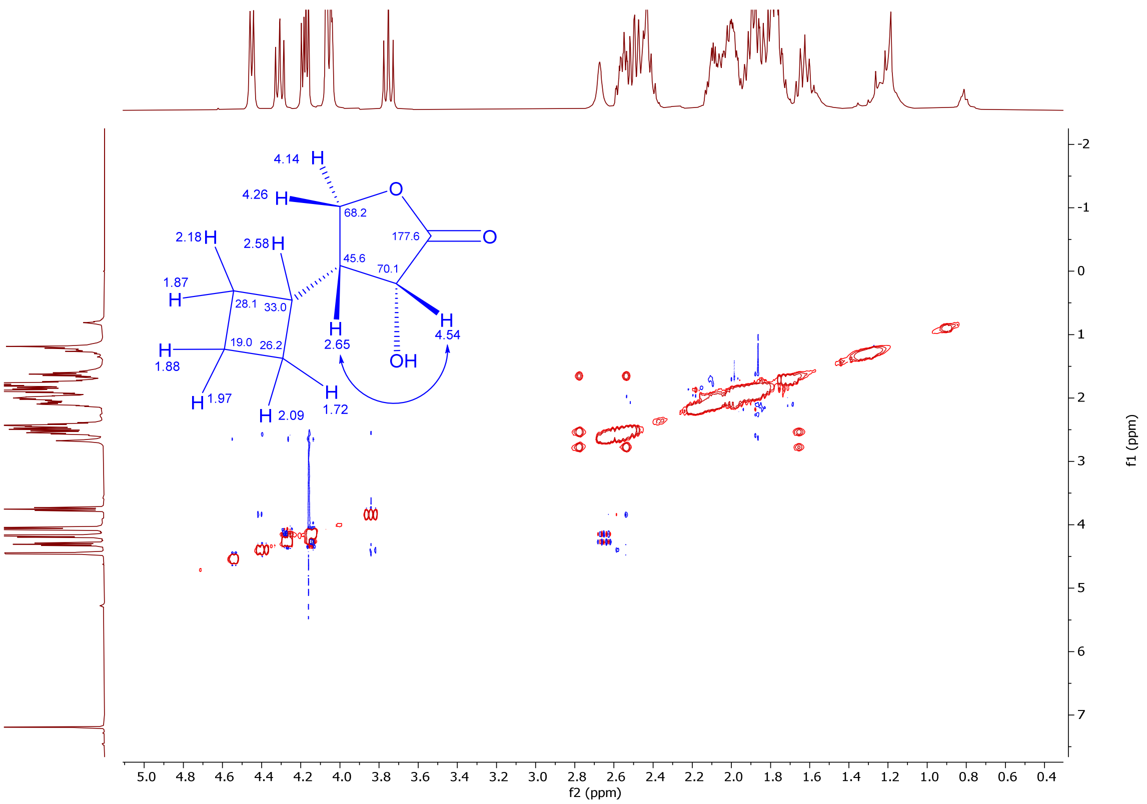

Figure S49. NMR spectra ($CDCl_3$) of 2*R*,3*R*-**5h**: a) ^{1}H, b) ^{13}C, c) COSY, d) HSQC, e) HMBC and f) NOESY.

a)

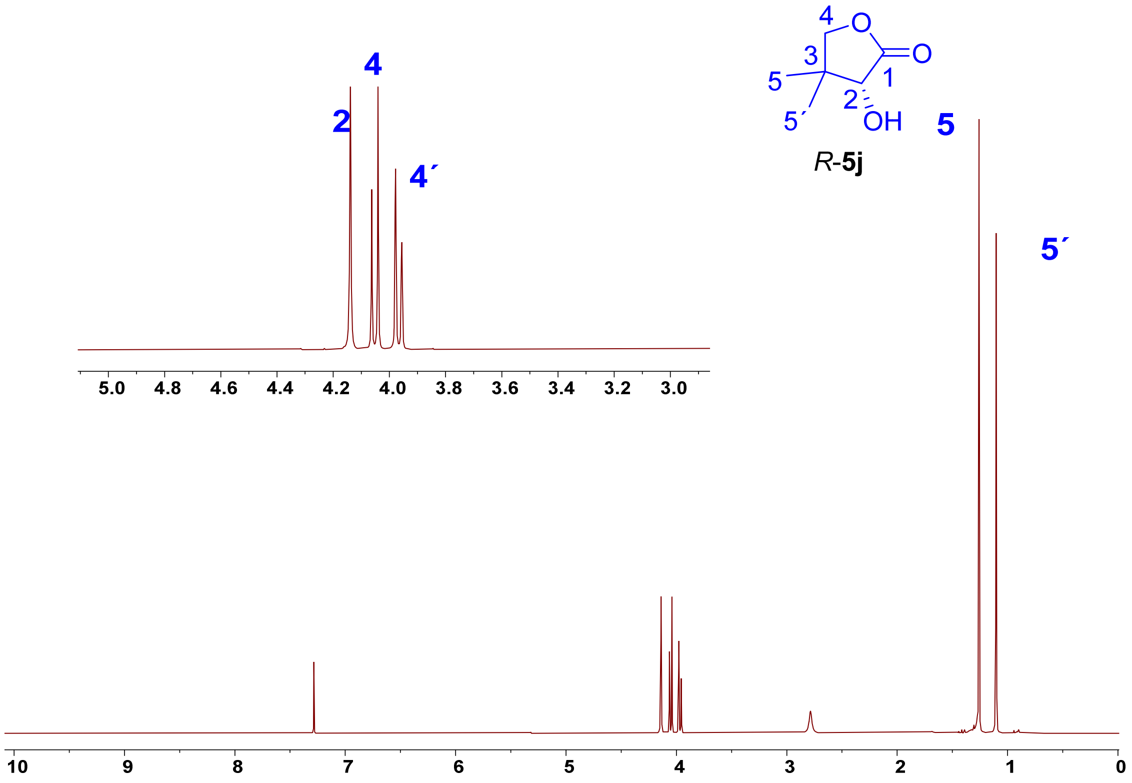

b)

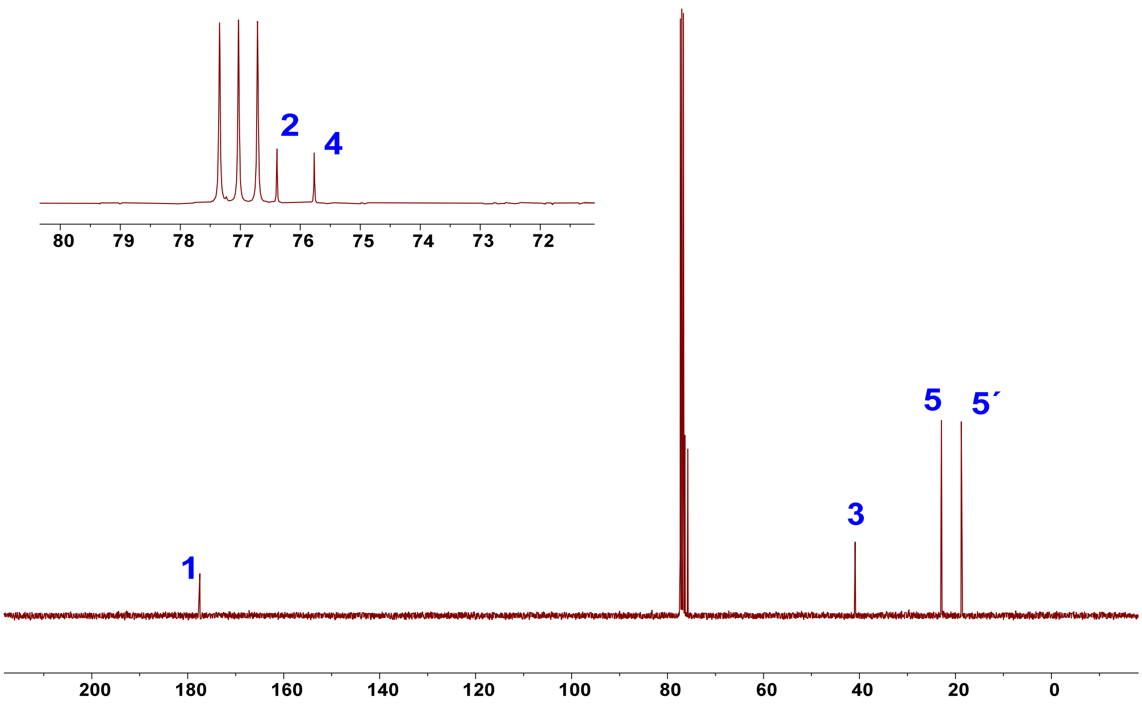

2
4
80
79
78
77
76
75
74
73
72
1
3
5
5´
200
180
160
140
120
100
80
60
40
20
0

c)

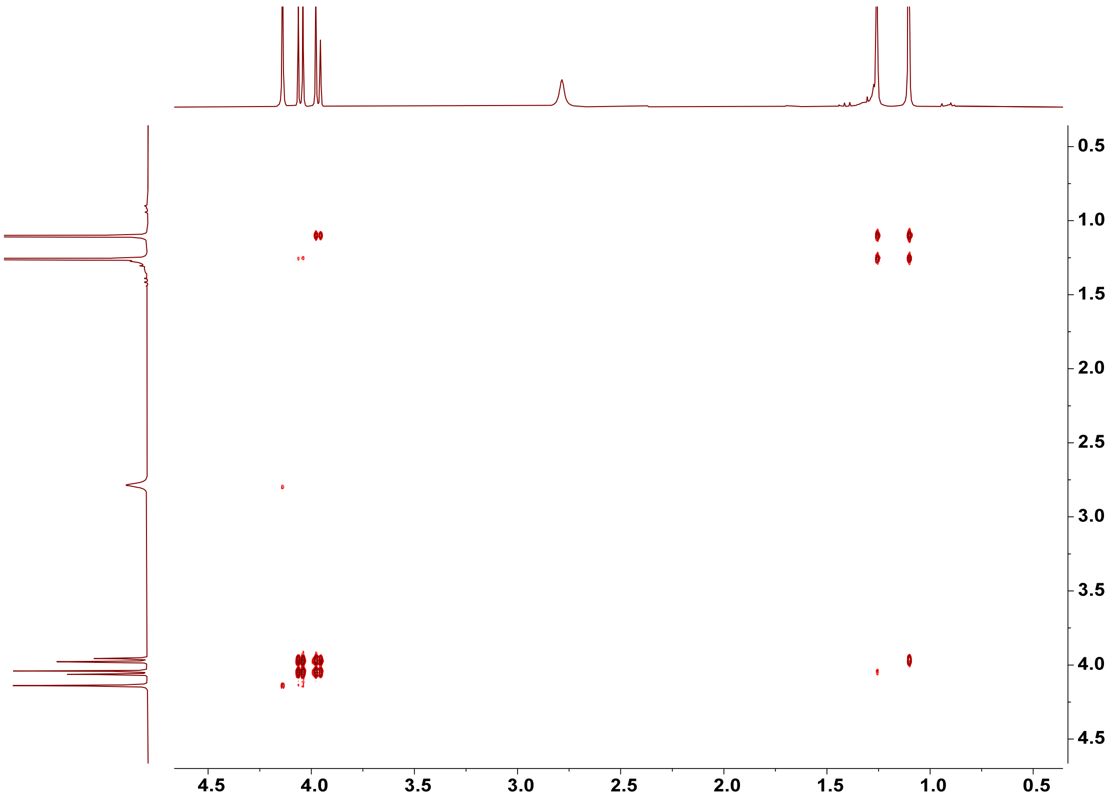

0.5
1.0
1.5
2.0
2.5
3.0
3.5
4.0
4.5
4.5
4.0
3.5
3.0
2.5
2.0
1.5
1.0
0.5

d)

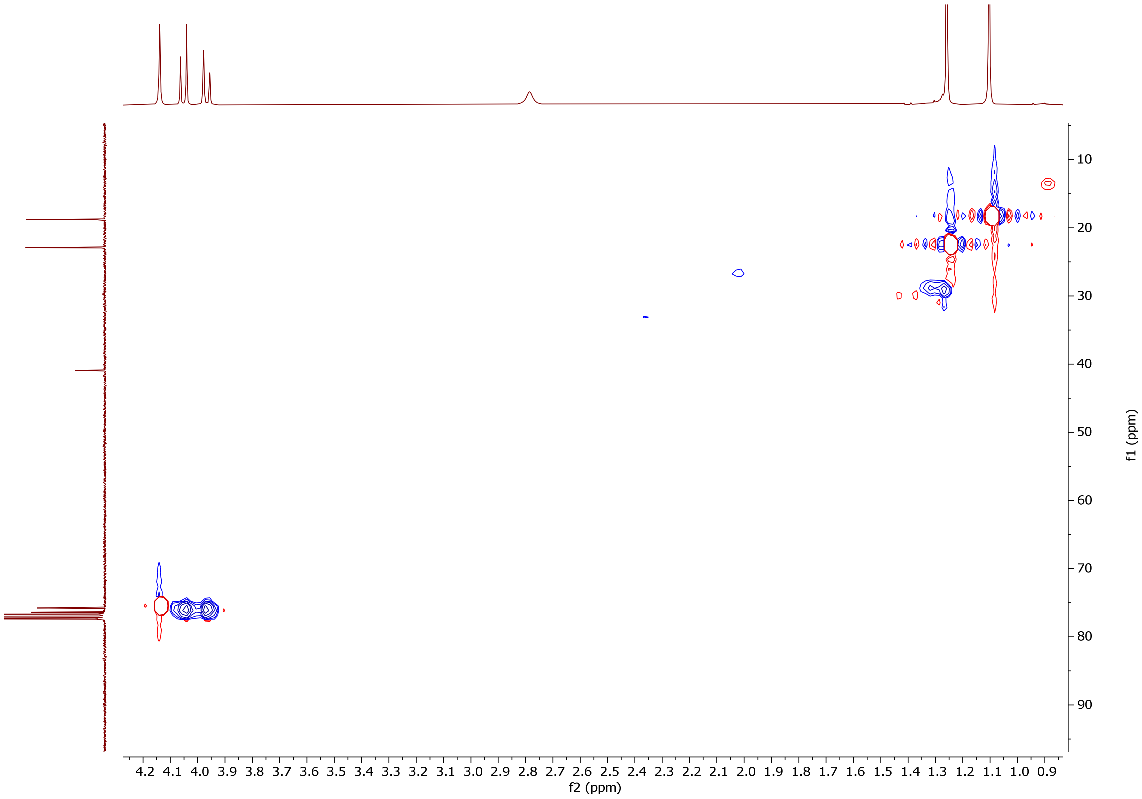

e)

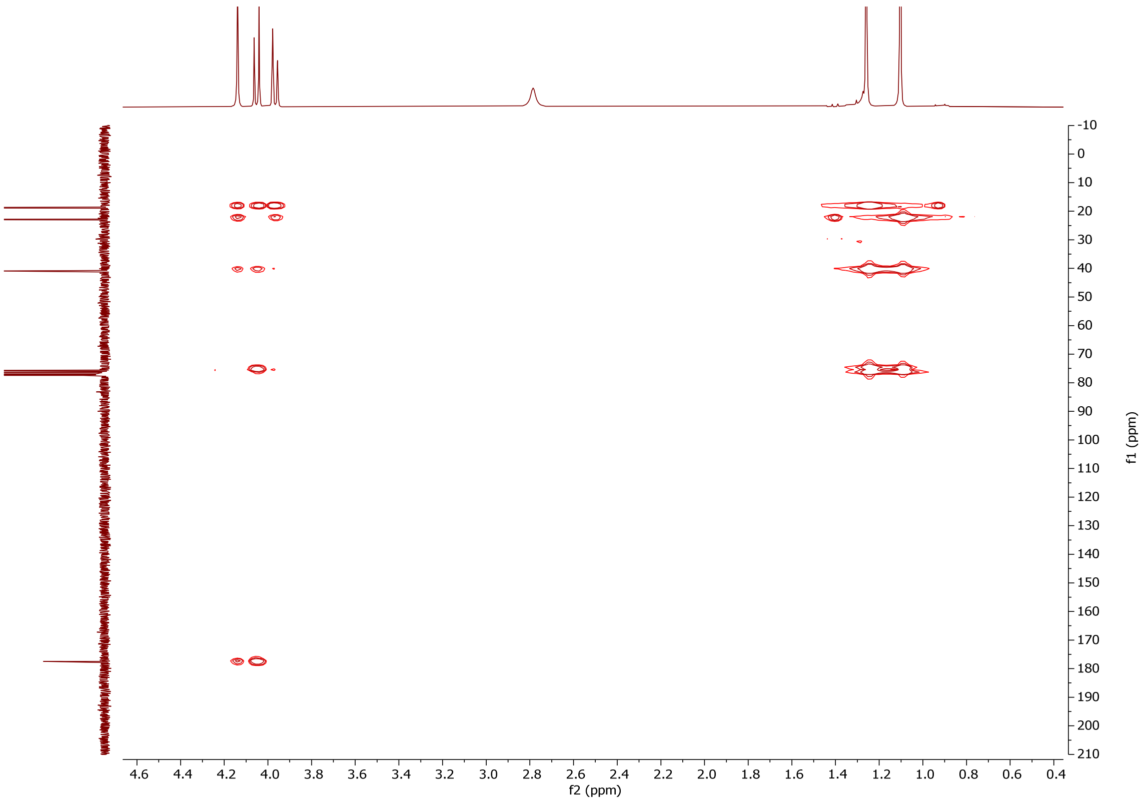

Figure S50. NMR spectra ($CDCl_3$) of *R*-**5j**: a) ^{1}H, b) ^{13}C, c) COSY, d) HSQC and HMBC.

a)

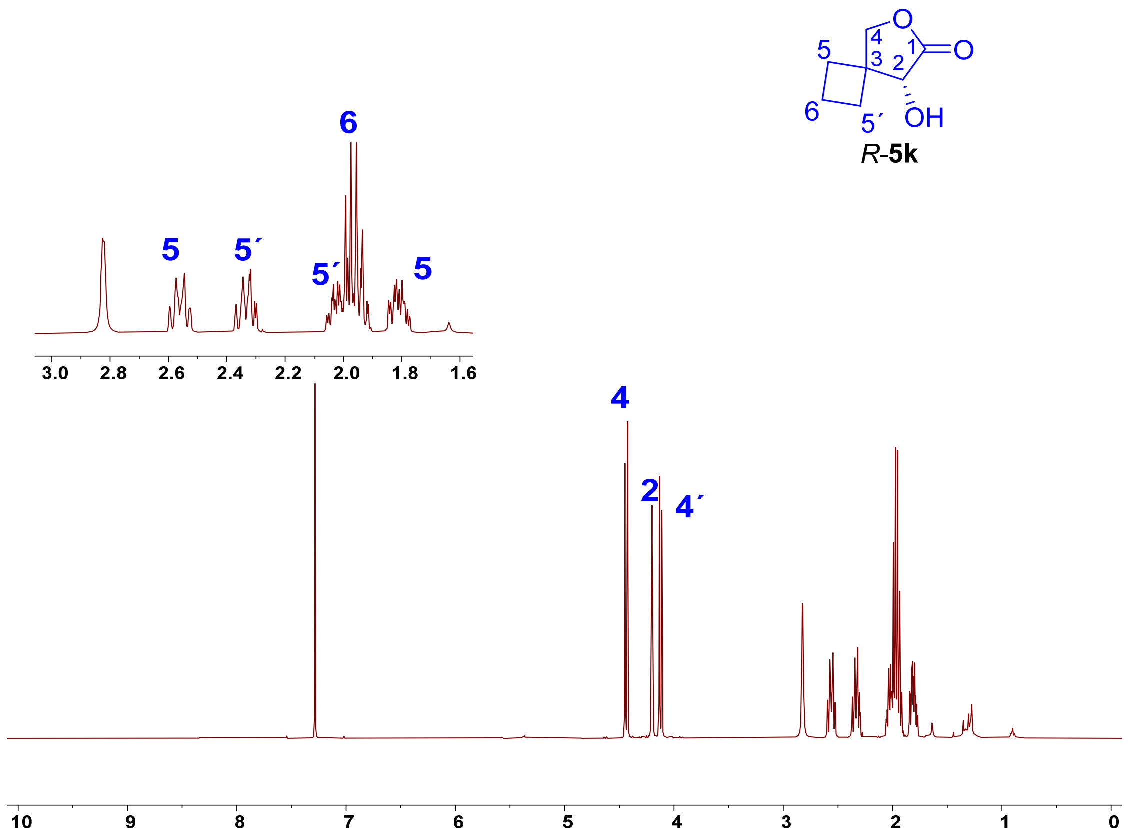

b)

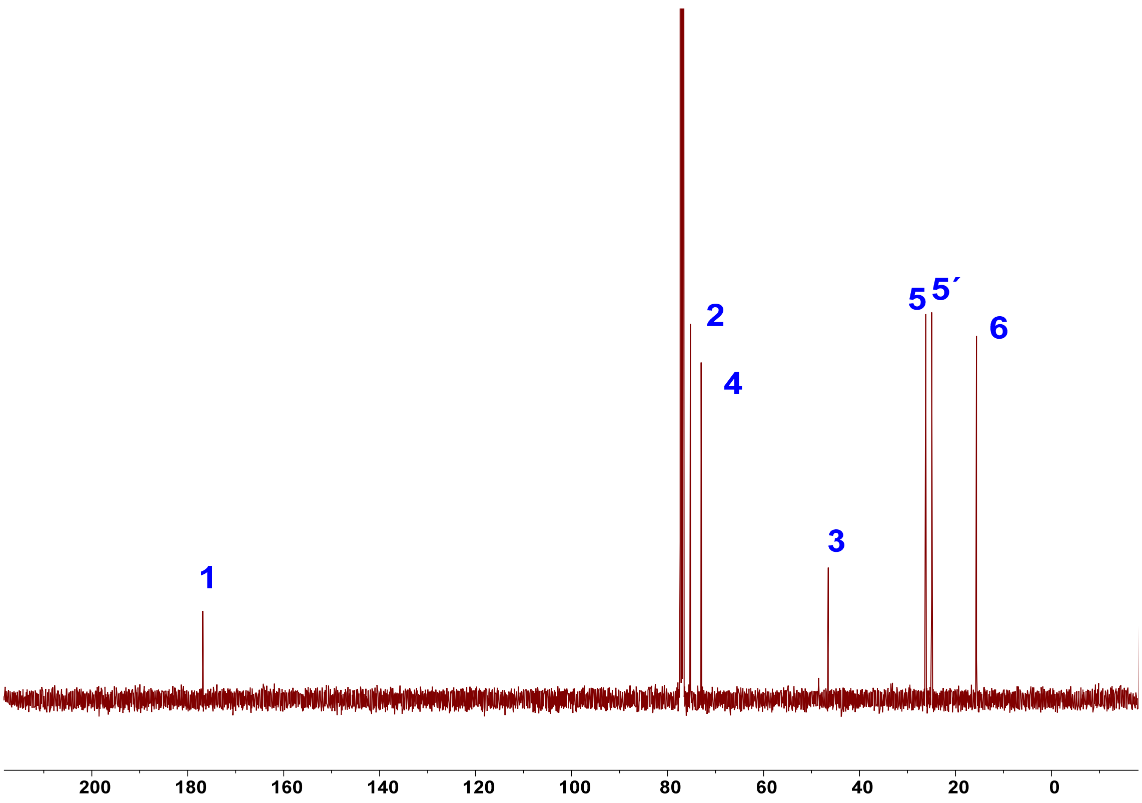

1
2
4
3
5
5´
6
200 180 160 140 120 100 80 60 40 20 0

c)

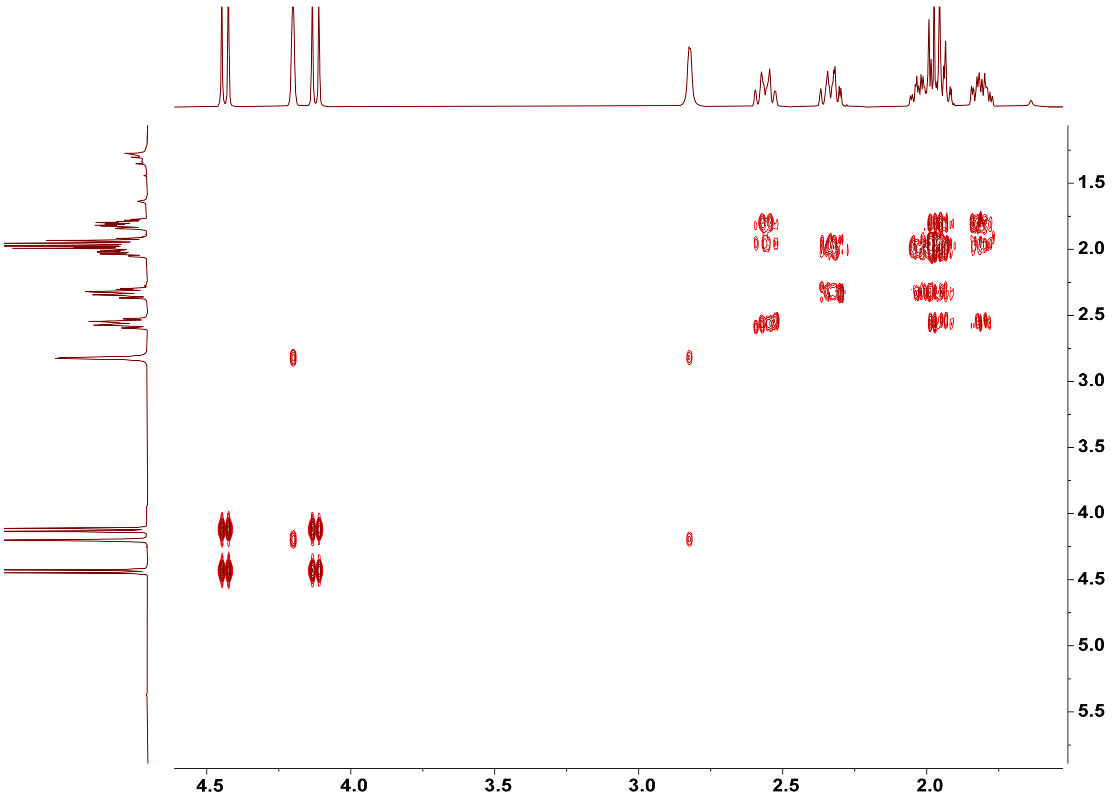

1.5
2.0
2.5
3.0
3.5
4.0
4.5
5.0
5.5
4.5 4.0 3.5 3.0 2.5 2.0

d)

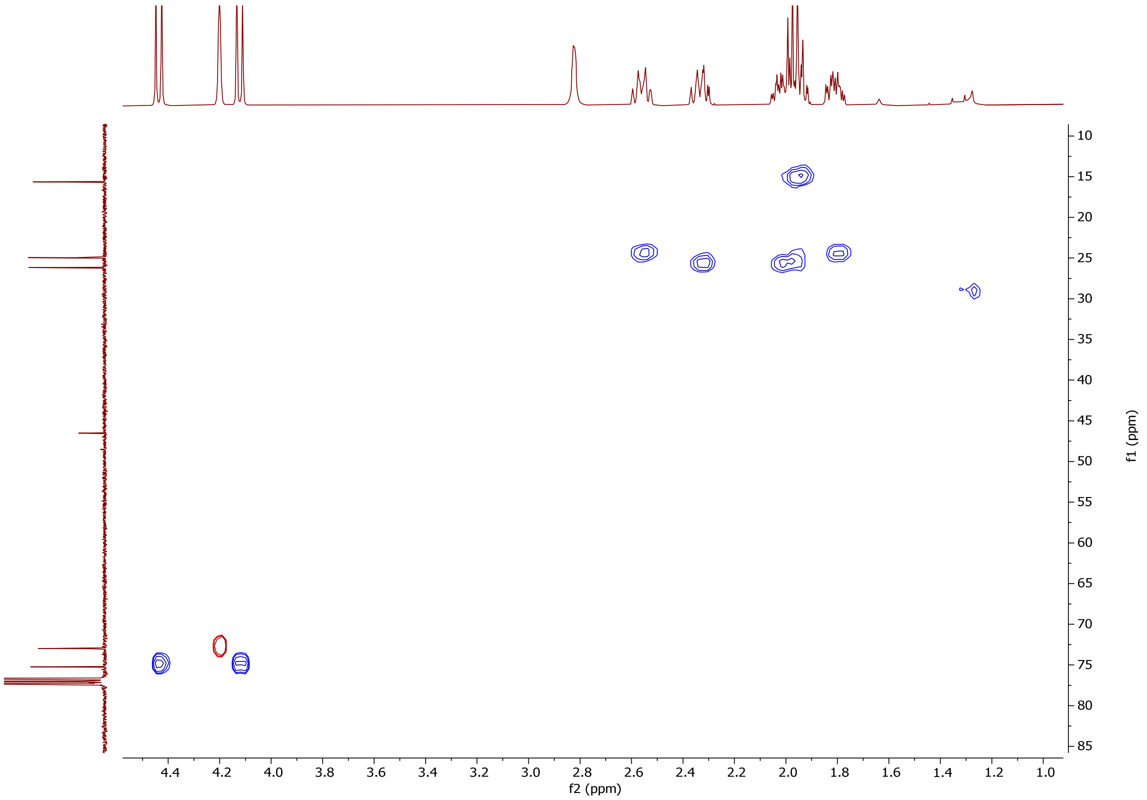

e)

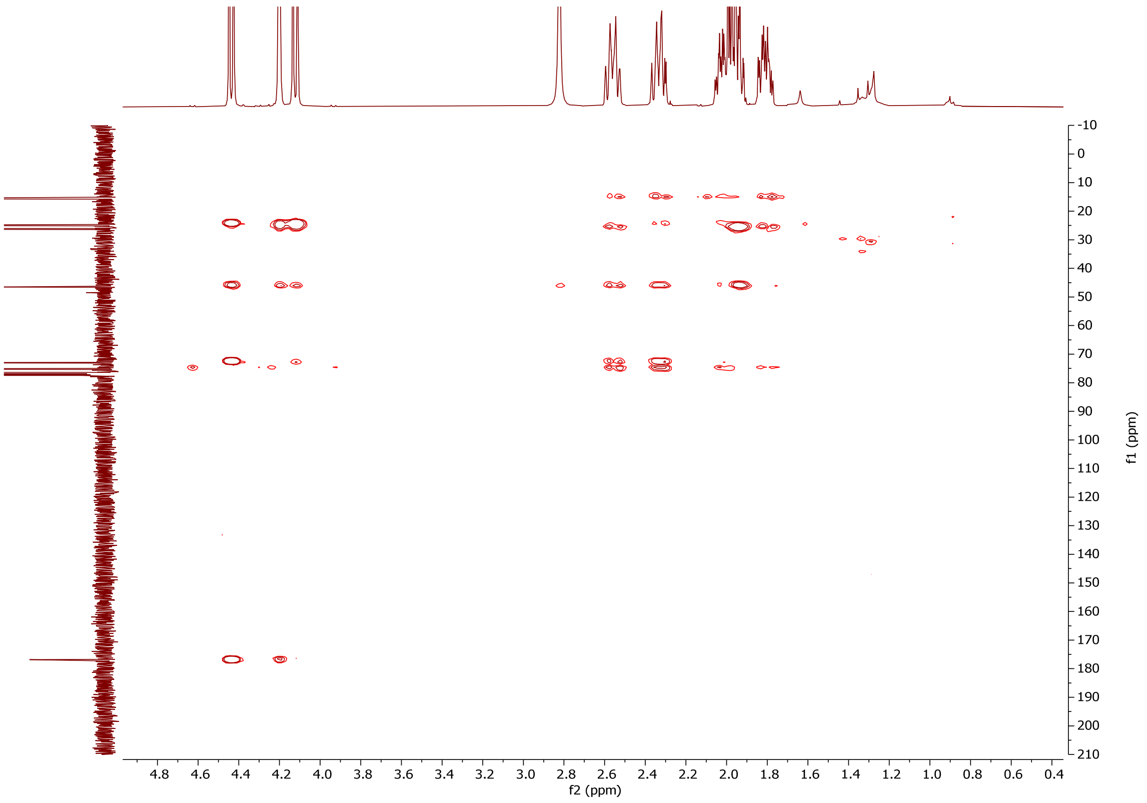

Figure S51. NMR spectra ($CDCl_3$) of *R*-**5k**: a) ^{1}H, b) ^{13}C, c) COSY, d) HSQC, and HMBC.

a)

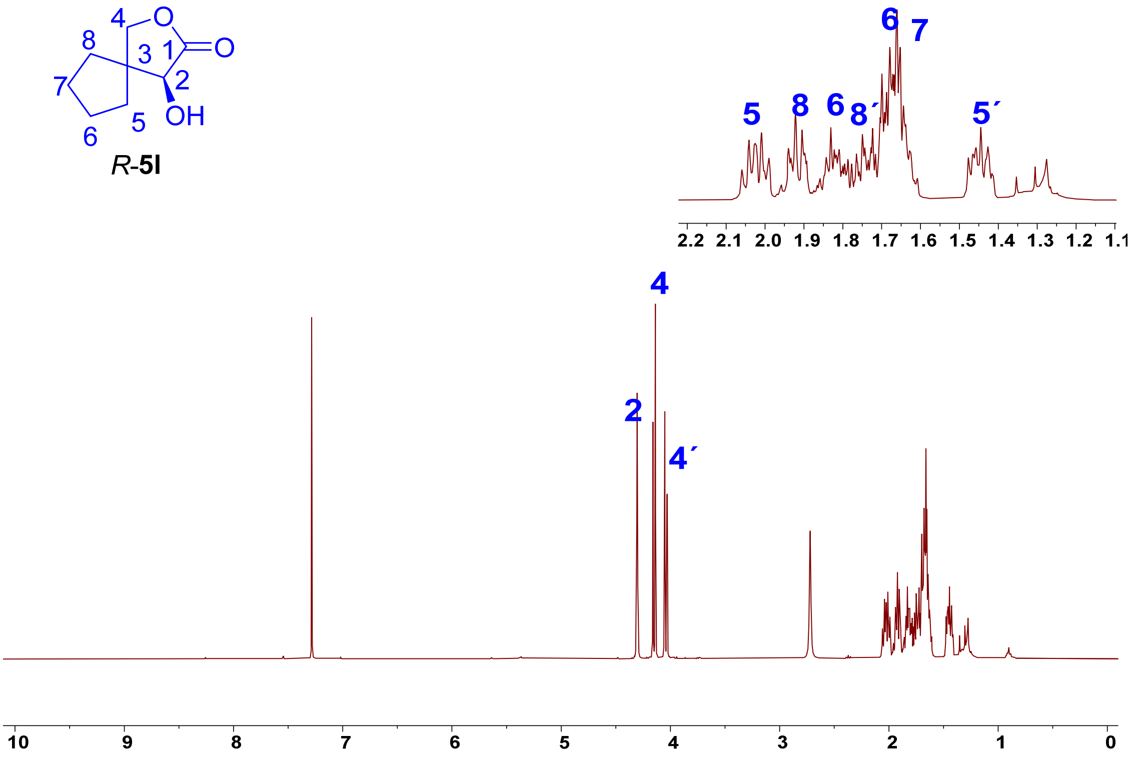

b)

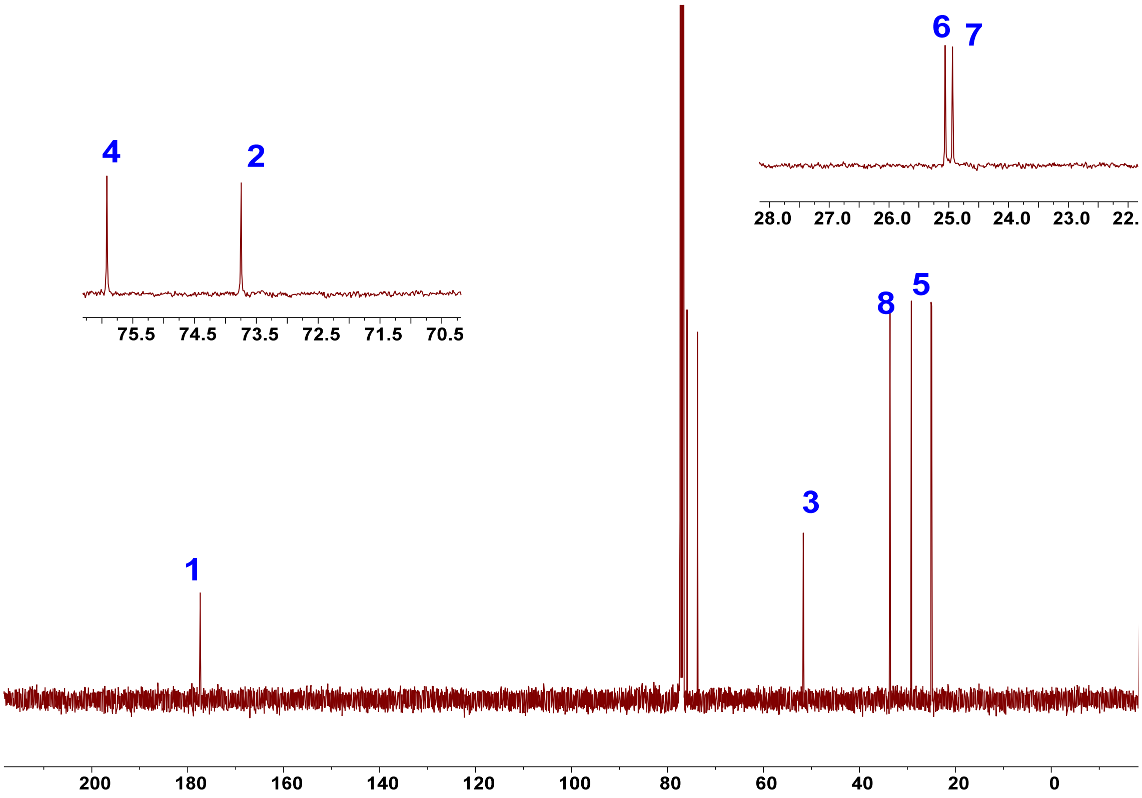

c)

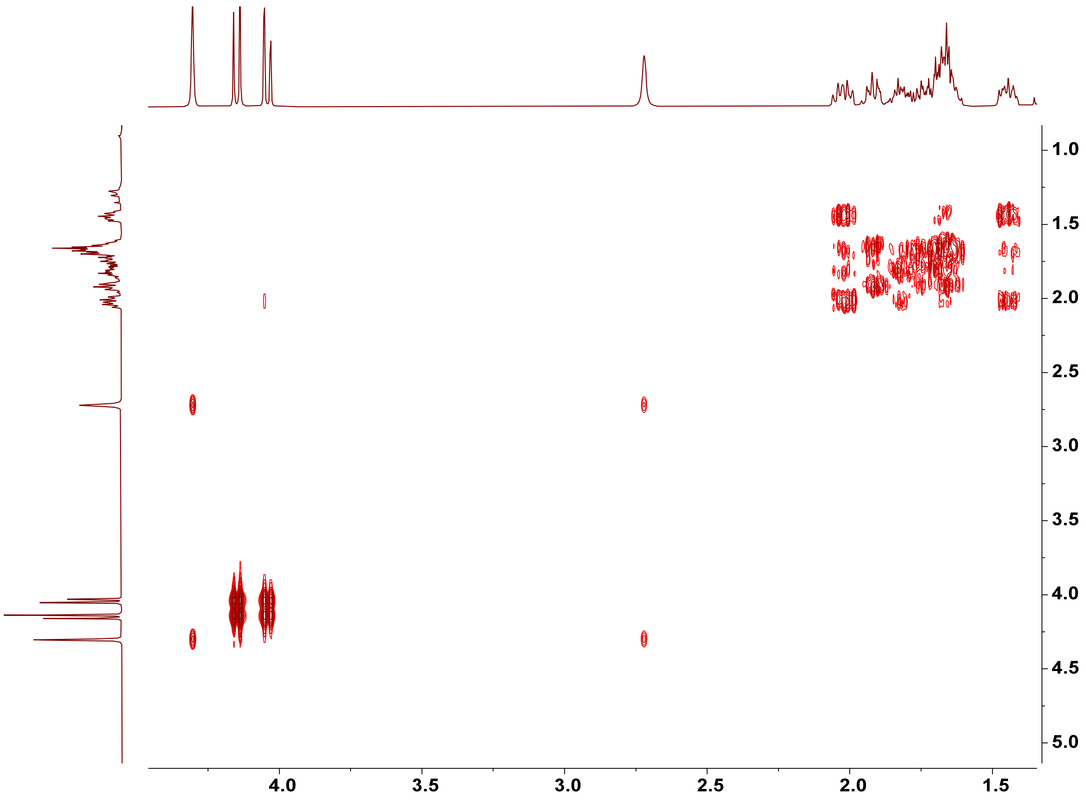

1.0
1.5
2.0
2.5
3.0
3.5
4.0
4.5
5.0
4.0
3.5
3.0
2.5
2.0
1.5

d)

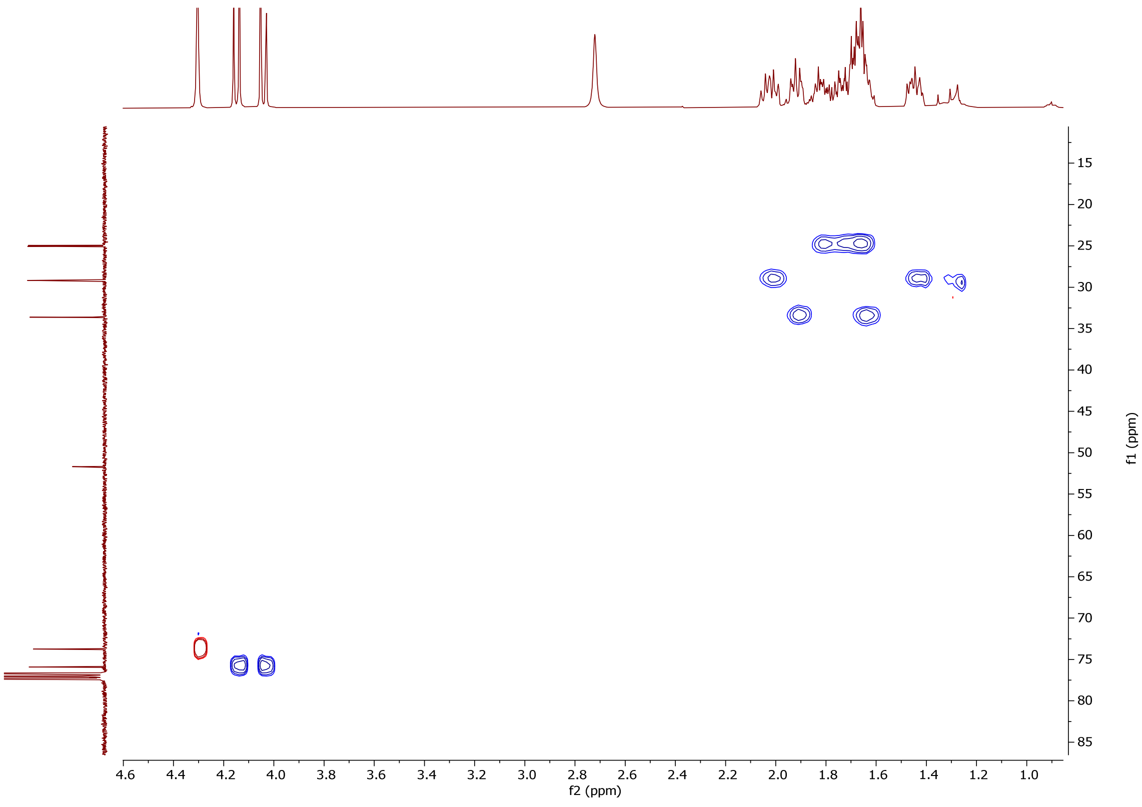

15
20
25
30
35
40
45
50
55
60
65
70
75
80
85
f1 (ppm)
4.6
4.4
4.2
4.0
3.8
3.6
3.4
3.2
3.0
2.8
2.6
2.4
2.2
2.0
1.8
1.6
1.4
1.2
1.0
f2 (ppm)

e)

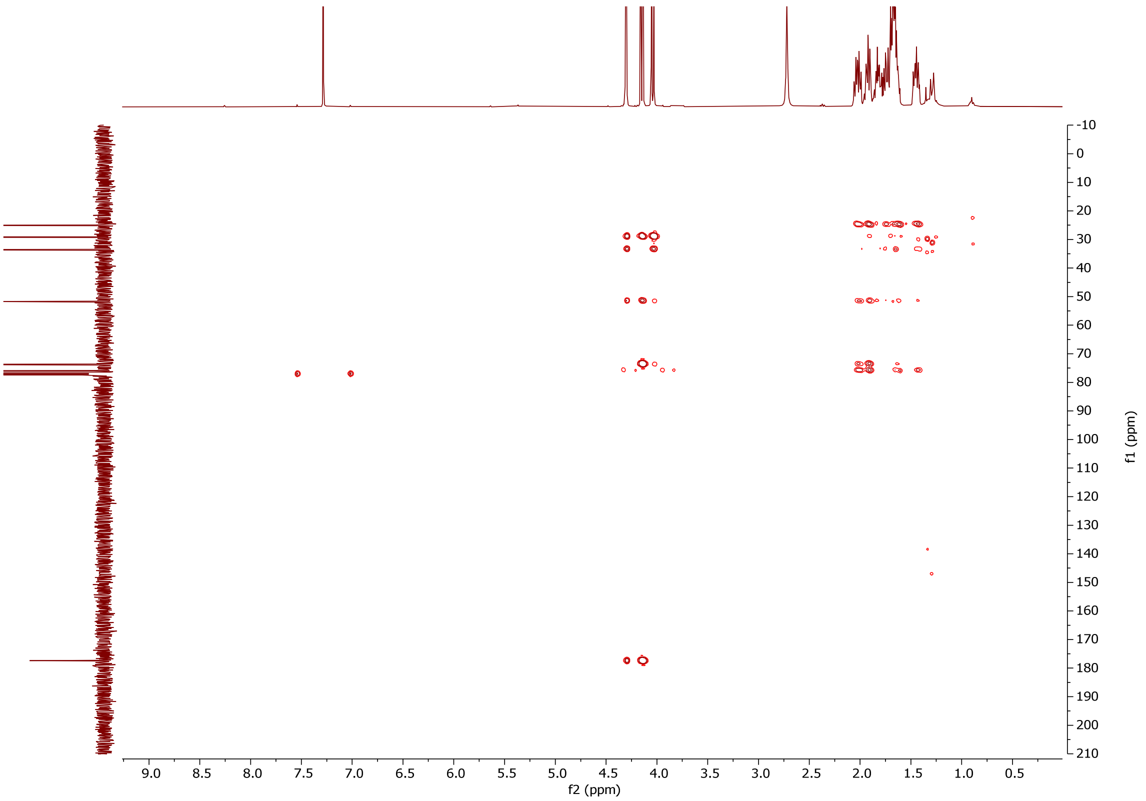

Figure S52. NMR spectra ($CDCl_3$) of *R*-**5l**: a) ^{1}H, b) ^{13}C, c) COSY, d) HSQC and e) HMBC.

a)

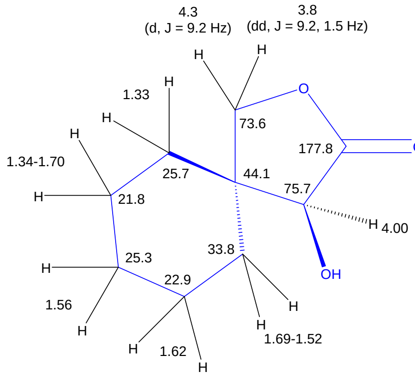

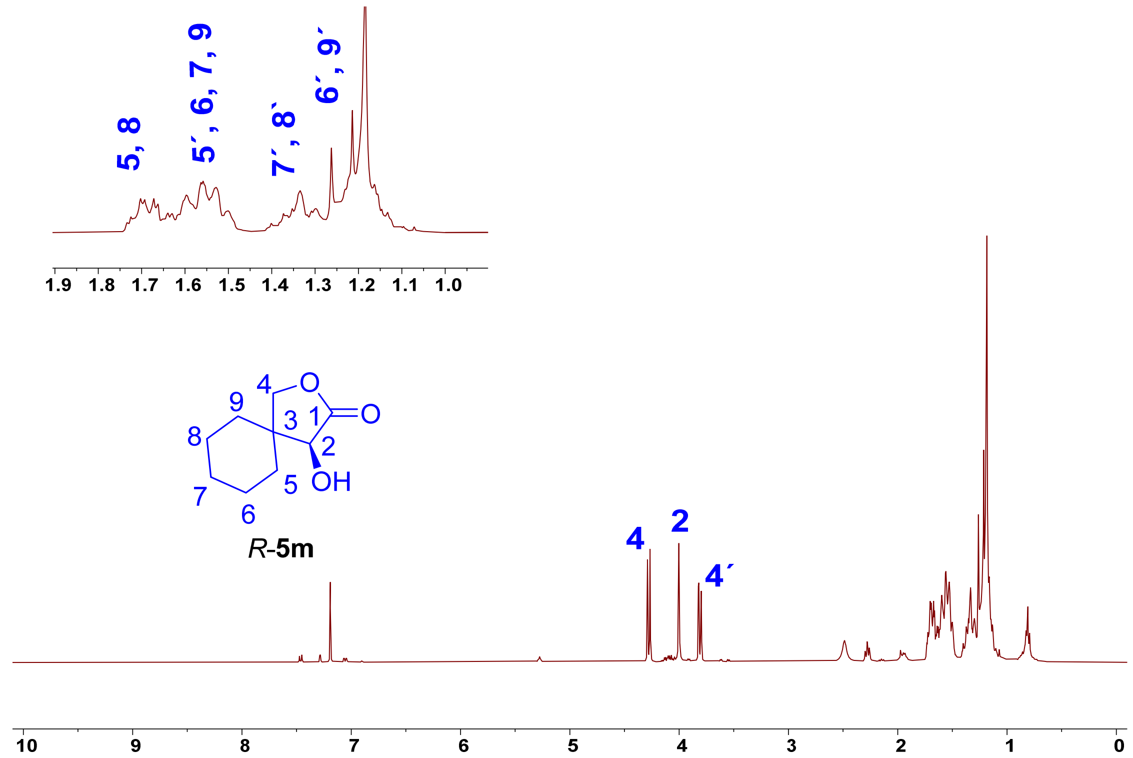
5, 8
5´, 6, 7, 9
7´, 8´
6´, 9´
1.9 1.8 1.7 1.6 1.5 1.4 1.3 1.2 1.1 1.0
R-**5m**
4
2
4´
10 9 8 7 6 5 4 3 2 1 0

b)

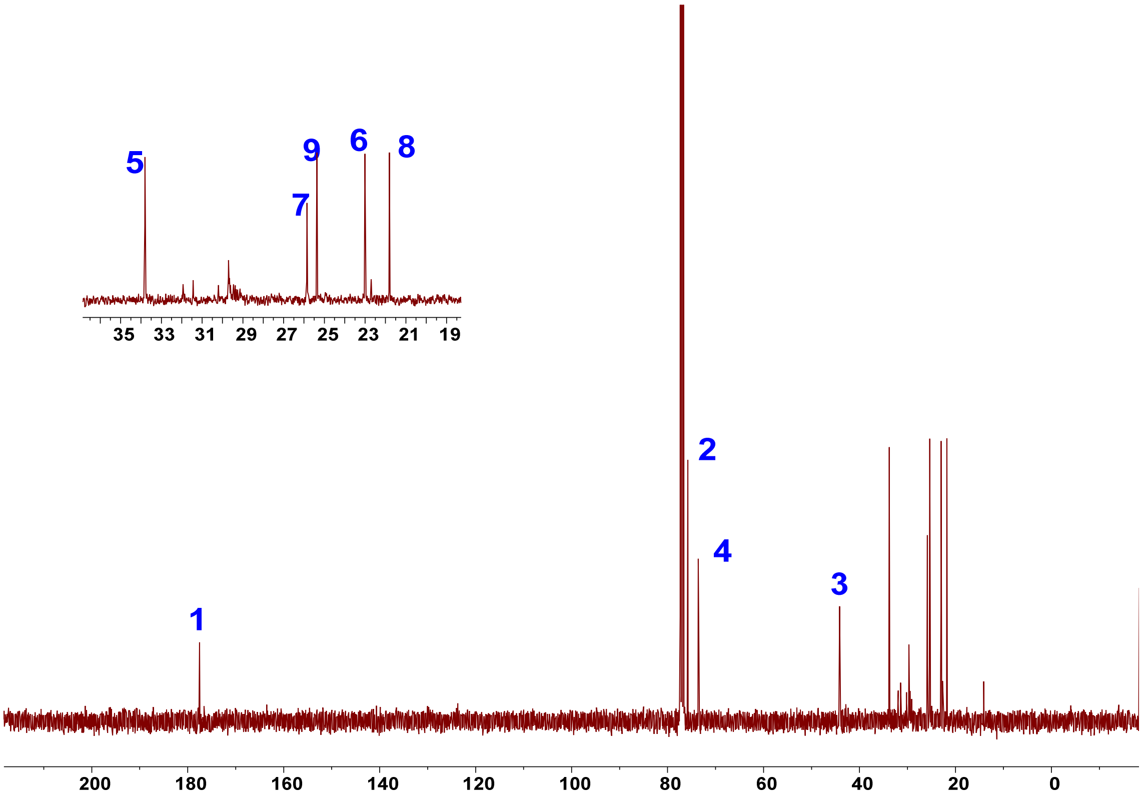
5
9
6
8
7
35 33 31 29 27 25 23 21 19
2
4
3
1
200 180 160 140 120 100 80 60 40 20 0

c)

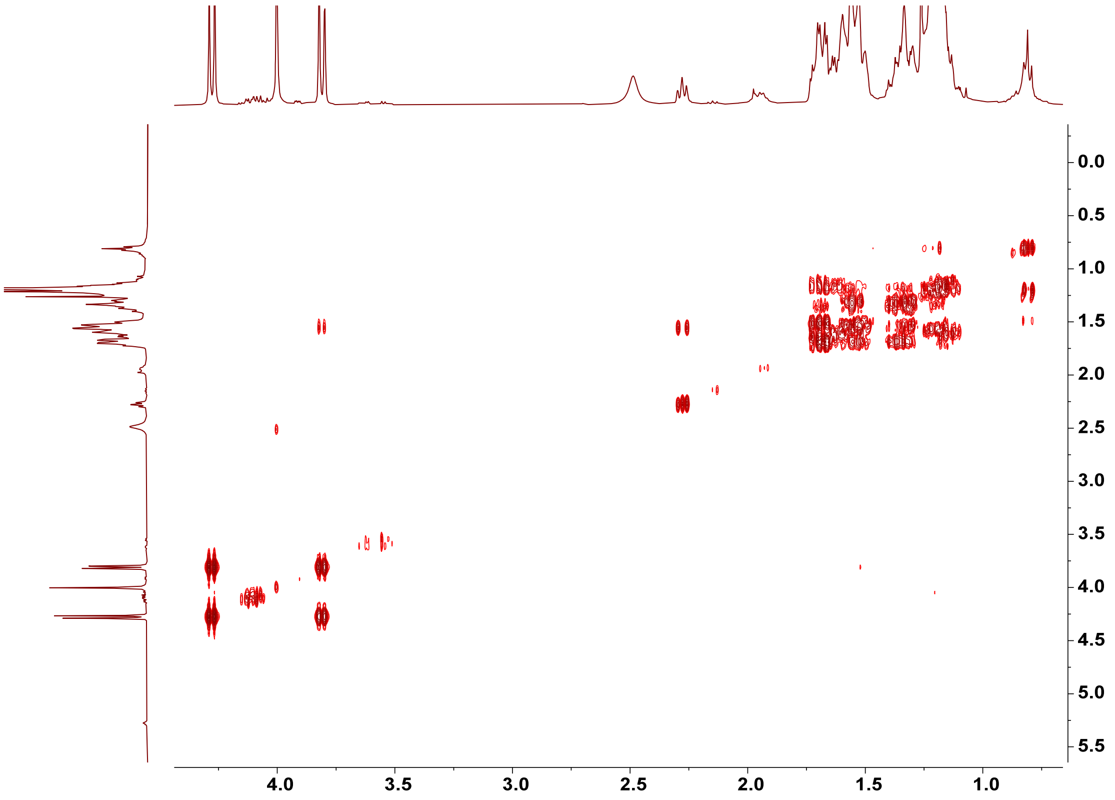
0.0
0.5
1.0
1.5
2.0
2.5
3.0
3.5
4.0
4.5
5.0
5.5
4.0 3.5 3.0 2.5 2.0 1.5 1.0

d)

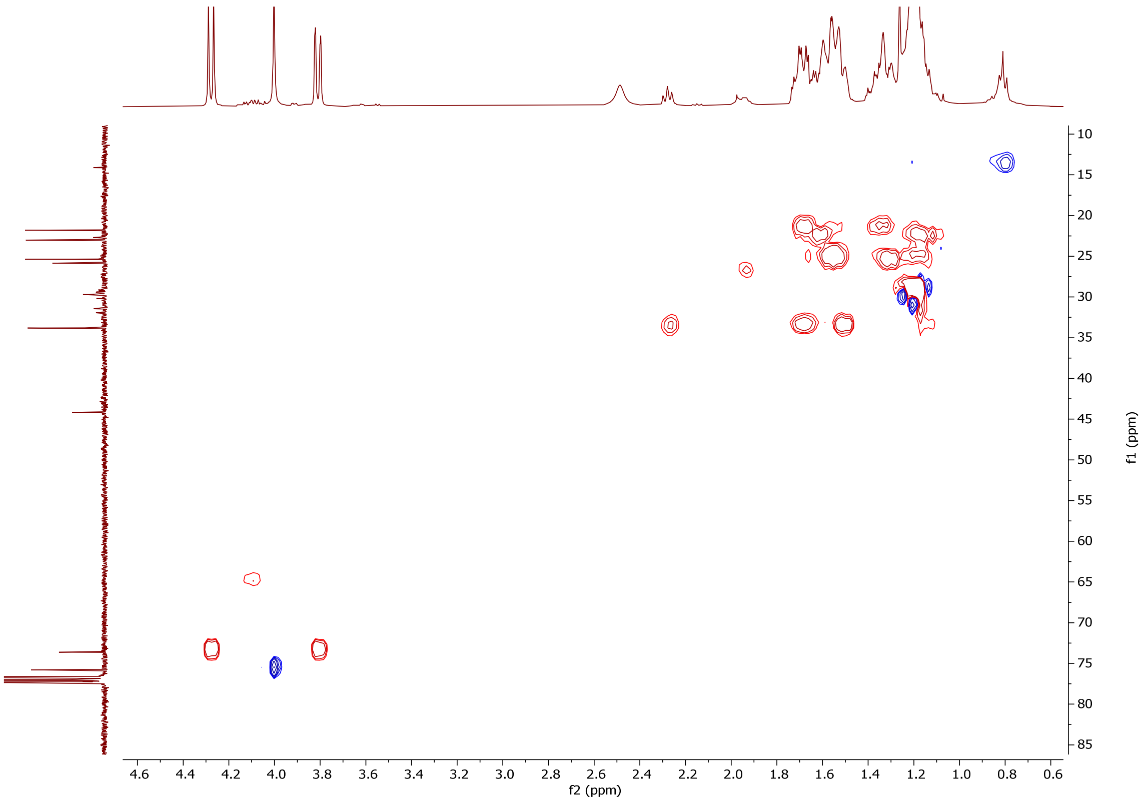
10
15
20
25
30
35
40
45
50
55
60
65
70
75
80
85
f1 (ppm)
4.6 4.4 4.2 4.0 3.8 3.6 3.4 3.2 3.0 2.8 2.6 2.4 2.2 2.0 1.8 1.6 1.4 1.2 1.0 0.8 0.6
f2 (ppm)

e)

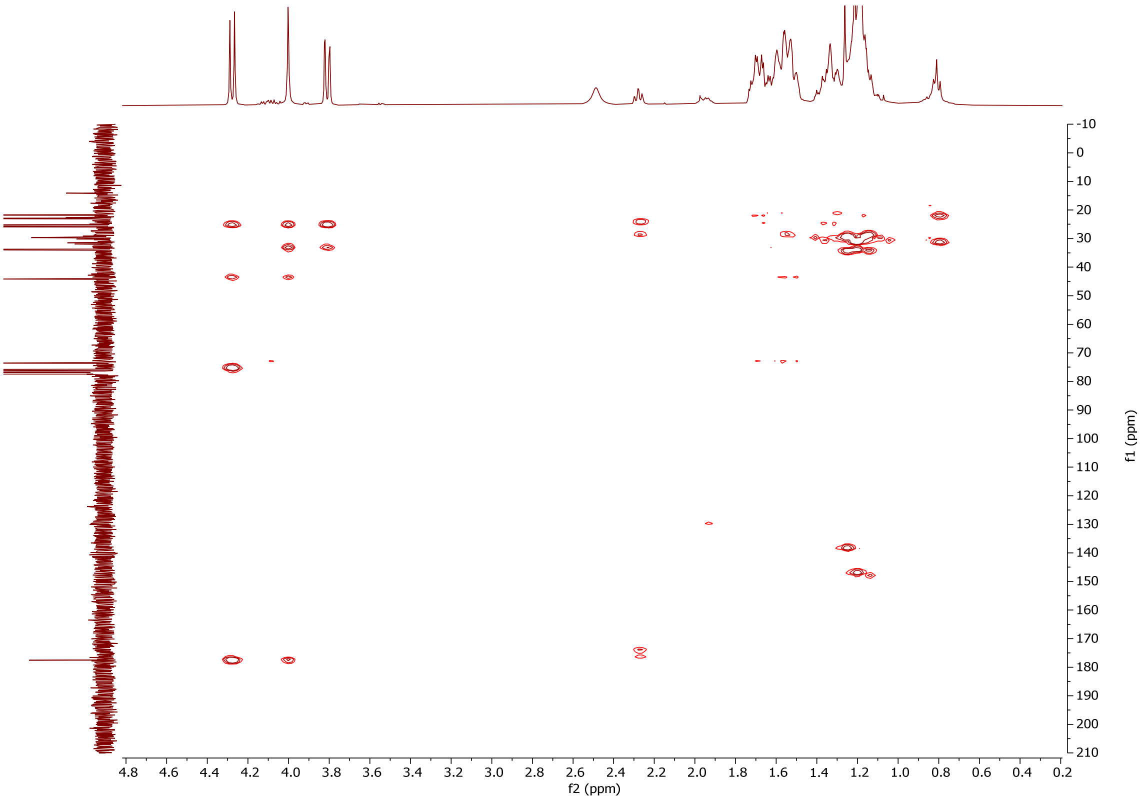

Figure S53. NMR spectra ($CDCl_3$) of *R*-**5m**: a) ^1H, b) ^{13}C, c) COSY, d) HSQC and HMBC.

a)

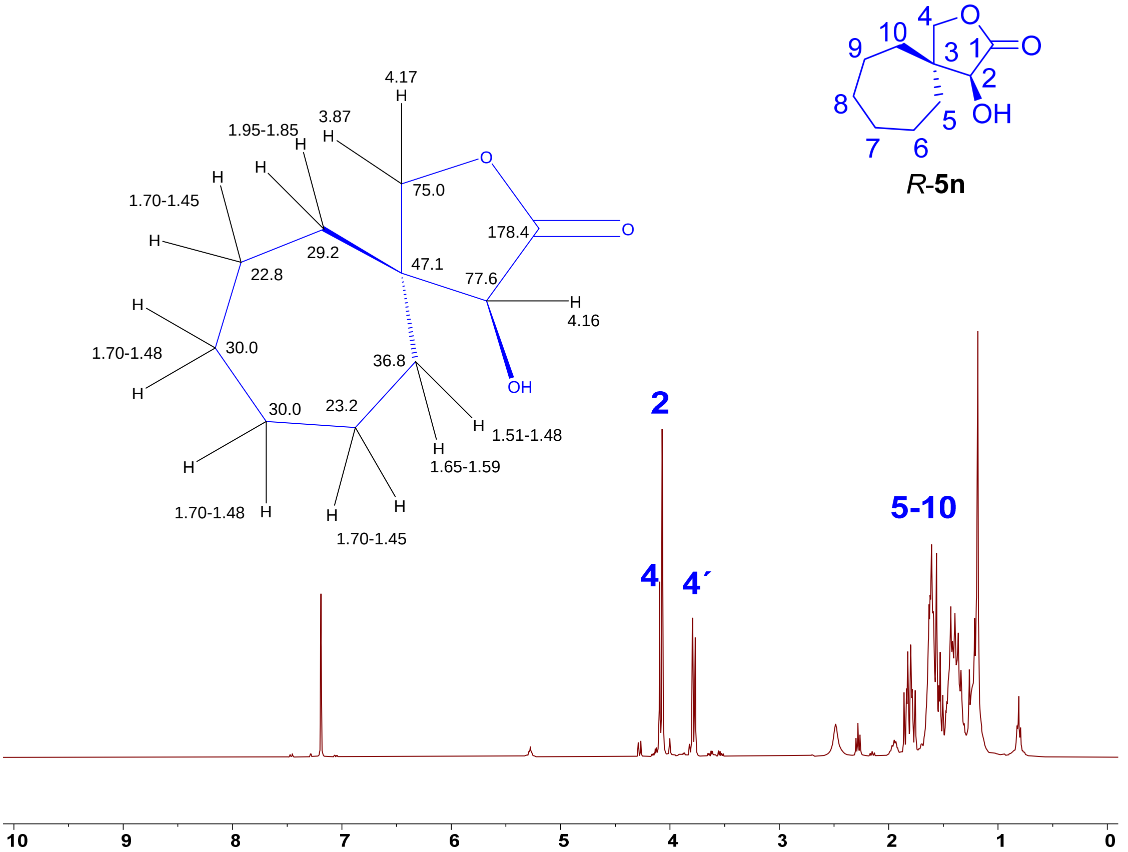

b)

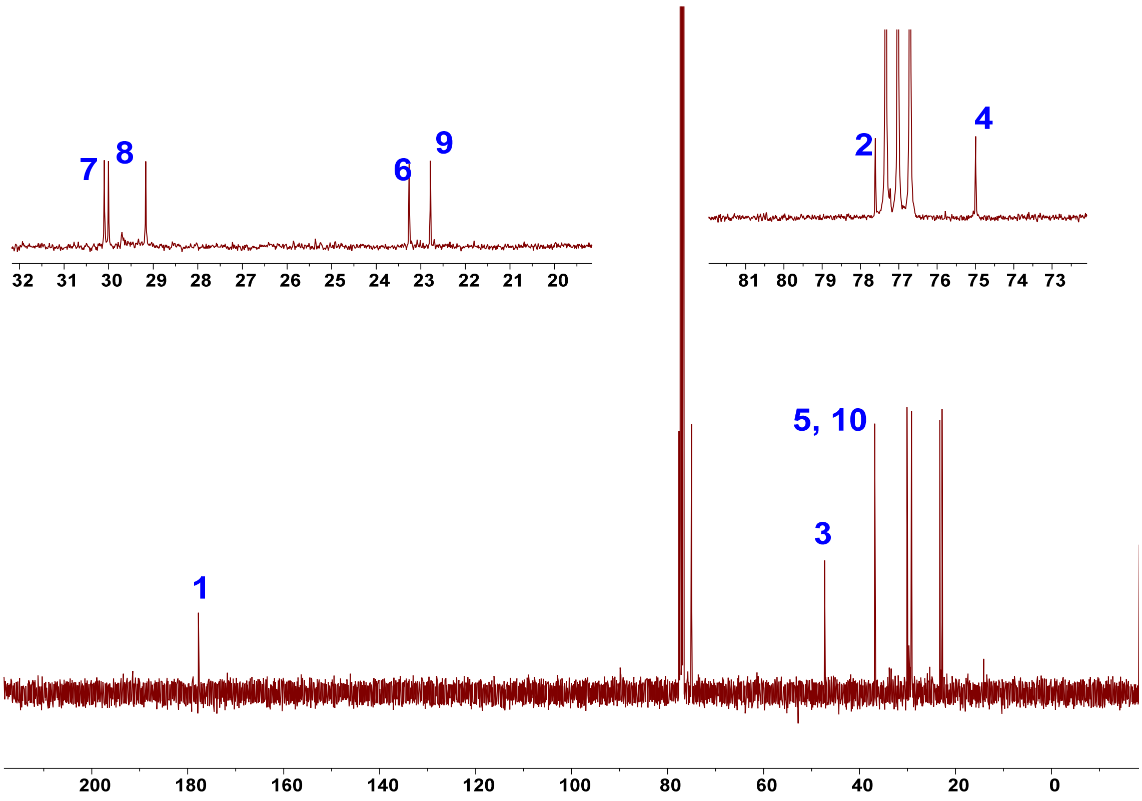

7
8
6
9
32 31 30 29 28 27 26 25 24 23 22 21 20
2
4
81 80 79 78 77 76 75 74 73
1
3
5, 10
200 180 160 140 120 100 80 60 40 20 0

c)

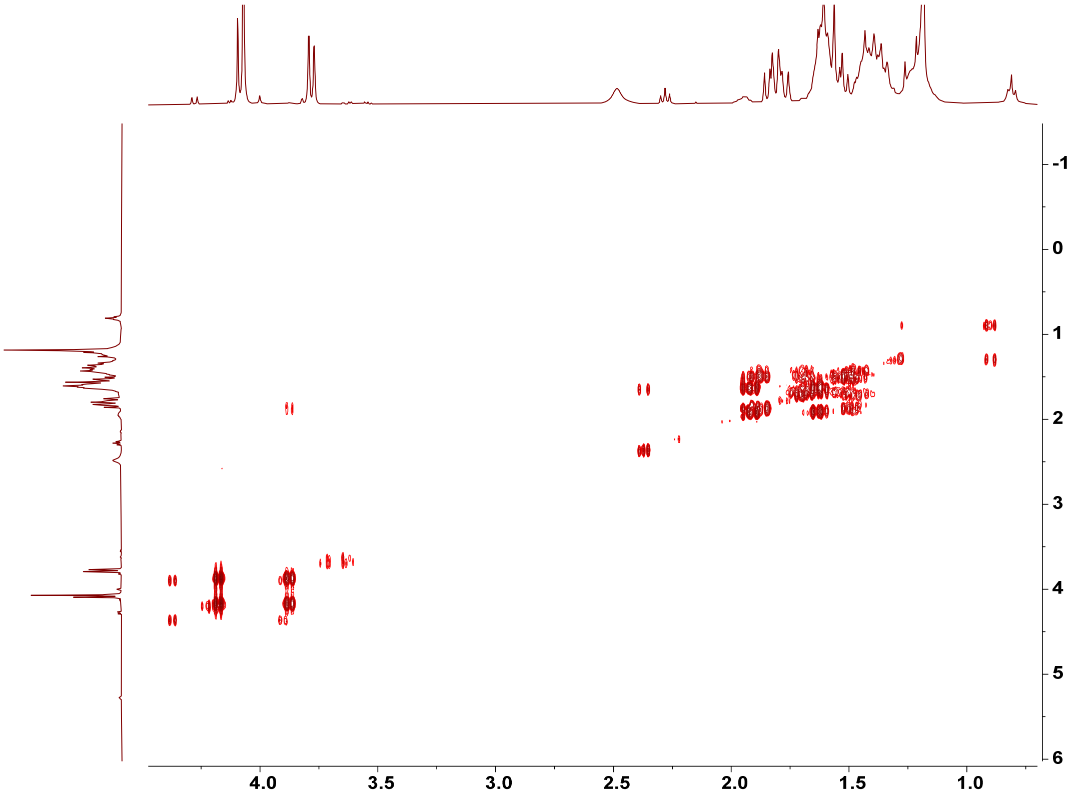

4.0 3.5 3.0 2.5 2.0 1.5 1.0
-1 0 1 2 3 4 5 6

d)

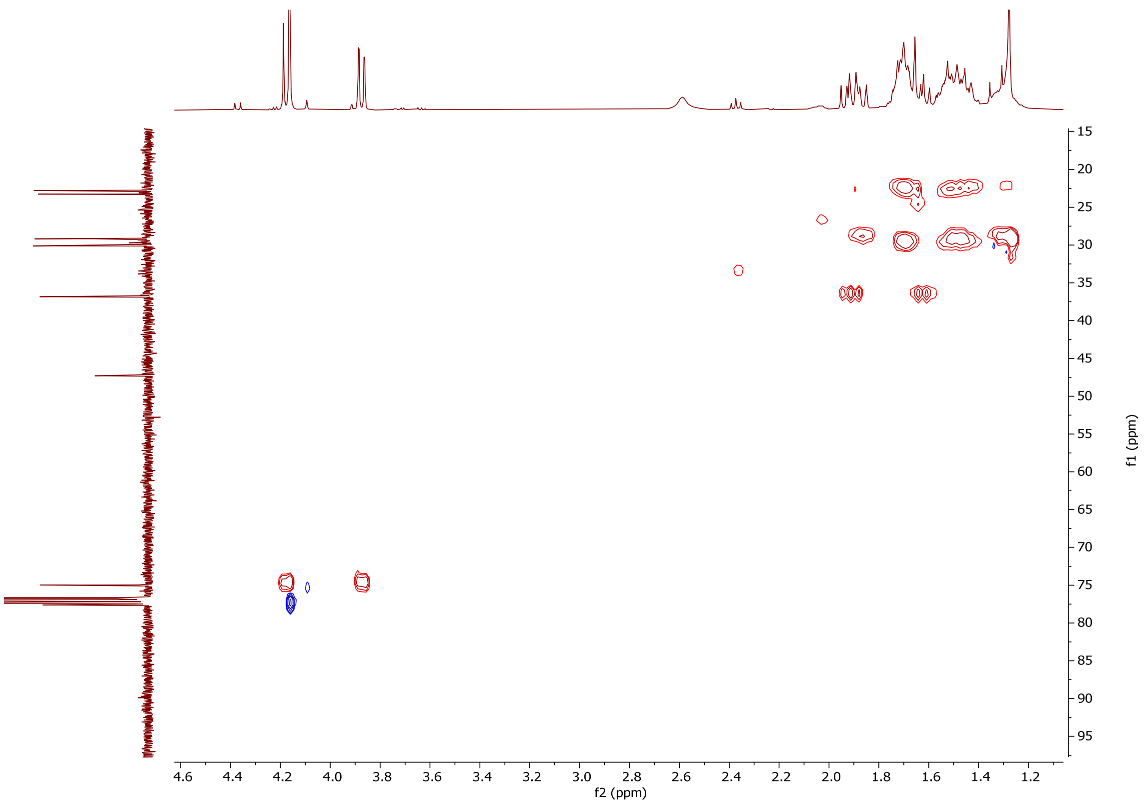

e)

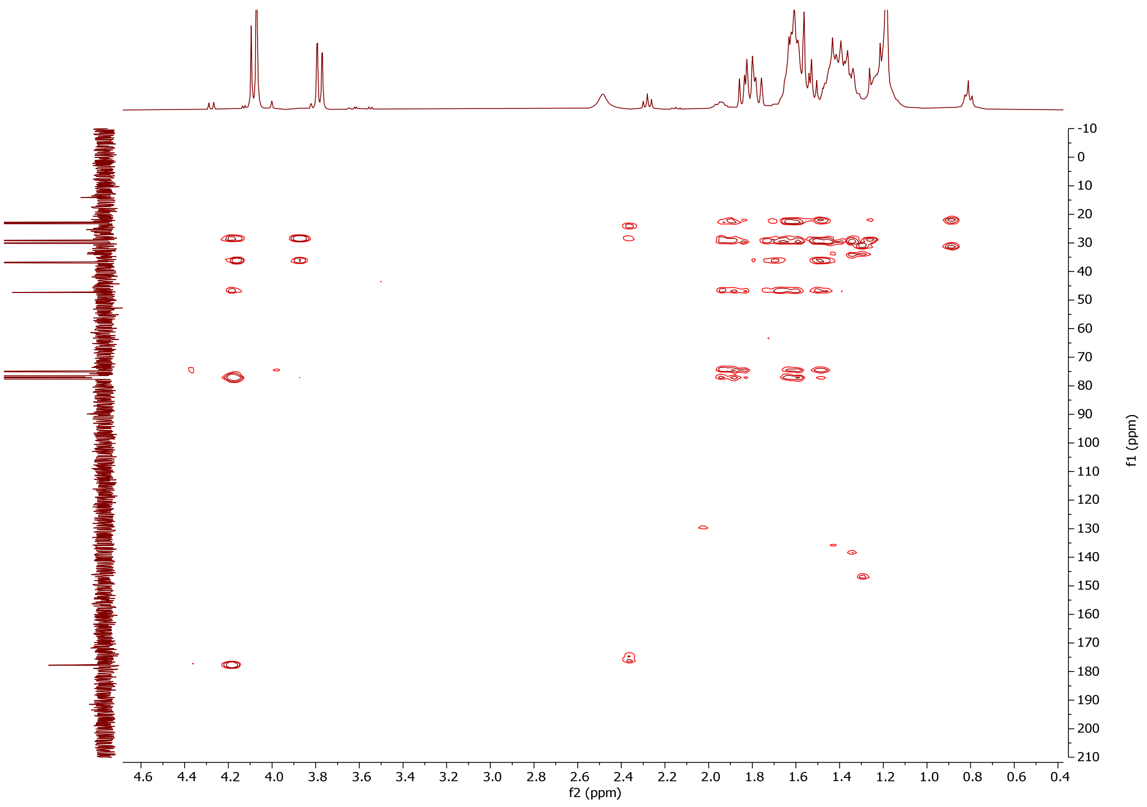

Figure S54. NMR spectra ($CDCl_3$) of *R*-**5n**: a) ^1H, b) ^{13}C, c) COSY, d) HSQC, and HMBC.

a)

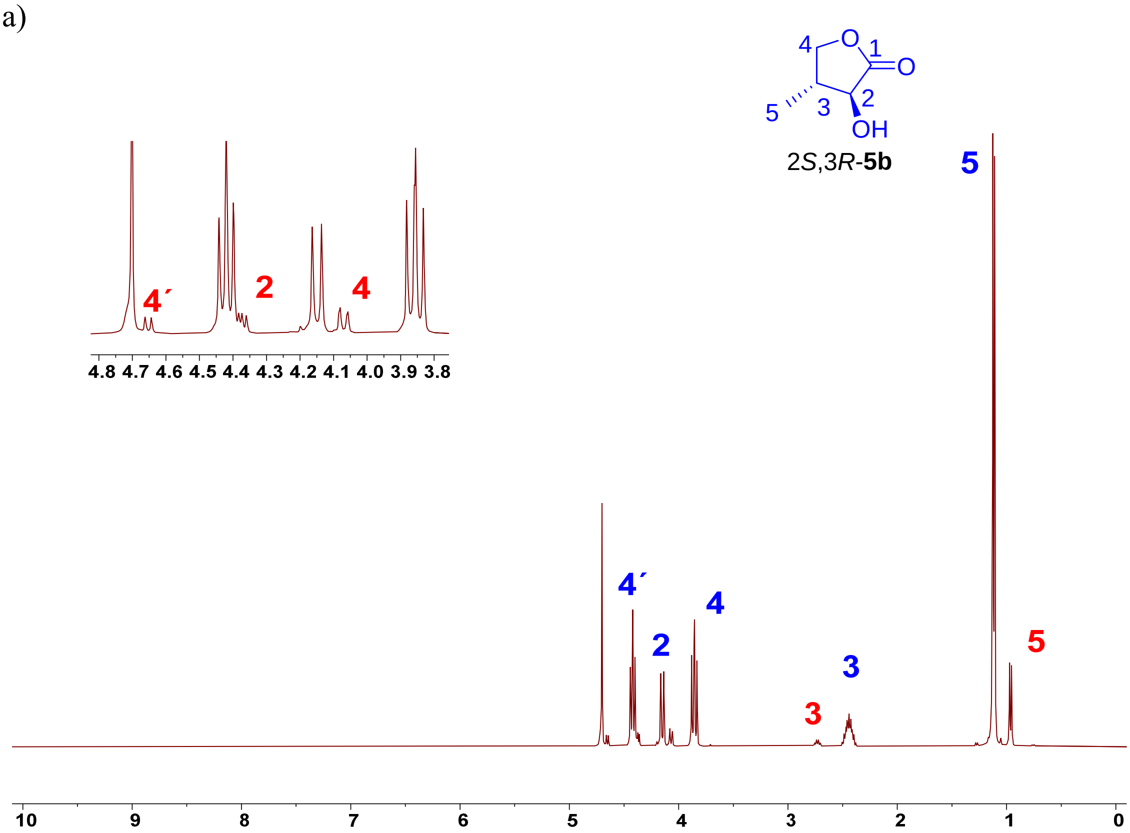
2*S*,3*R*-**5b**
4´
2
4
3
5
4.8 4.7 4.6 4.5 4.4 4.3 4.2 4.1 4.0 3.9 3.8
10 9 8 7 6 5 4 3 2 1 0

b)

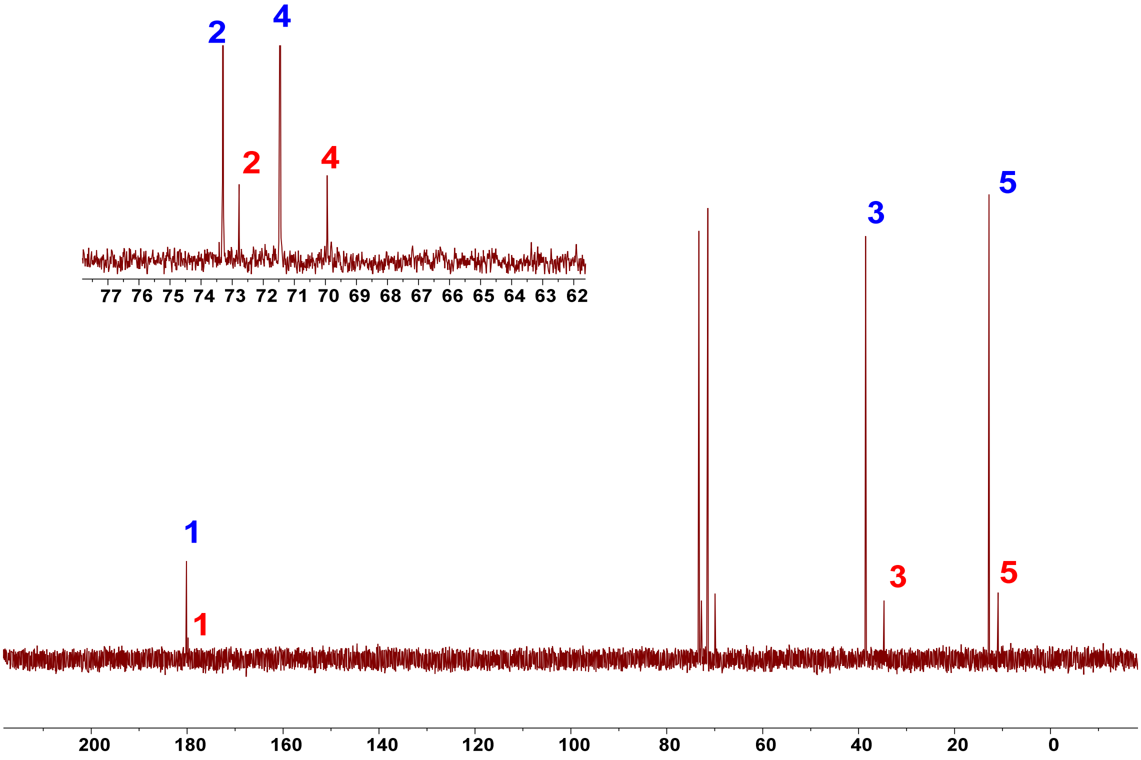
1
2
3
4
5
77 76 75 74 73 72 71 70 69 68 67 66 65 64 63 62
200 180 160 140 120 100 80 60 40 20 0

c)

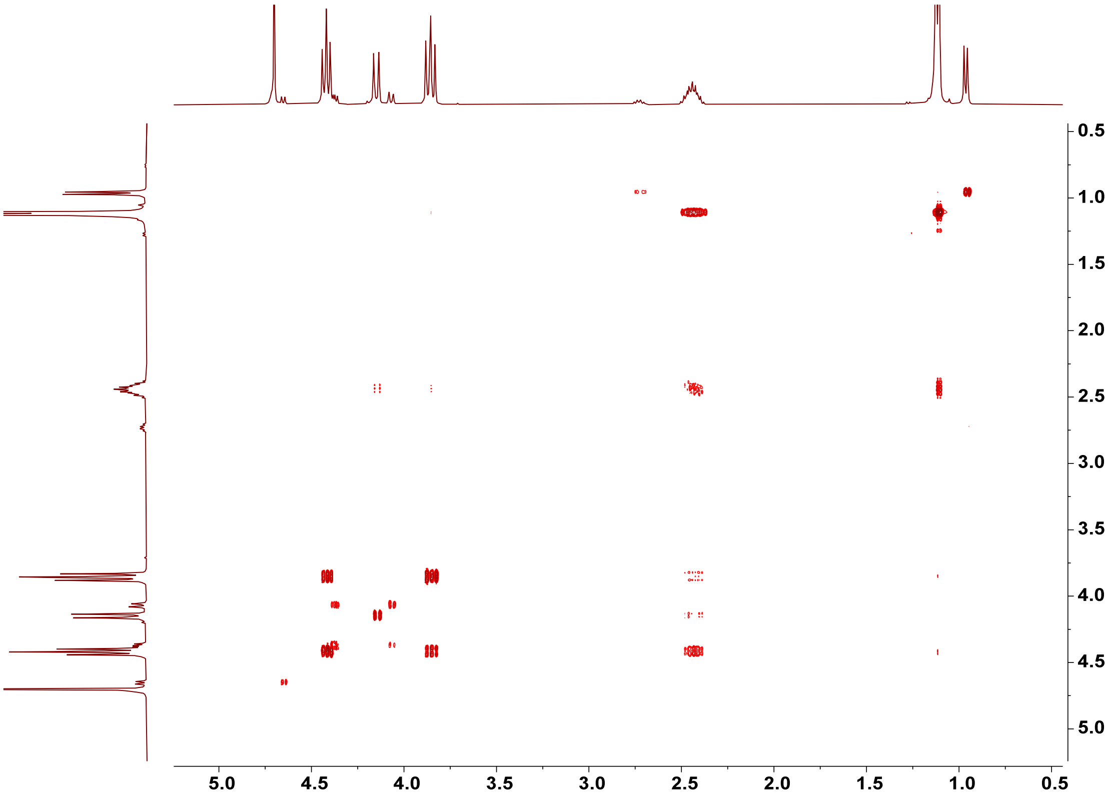
0.5
1.0
1.5
2.0
2.5
3.0
3.5
4.0
4.5
5.0
5.0
4.5
4.0
3.5
3.0
2.5
2.0
1.5
1.0
0.5

d)

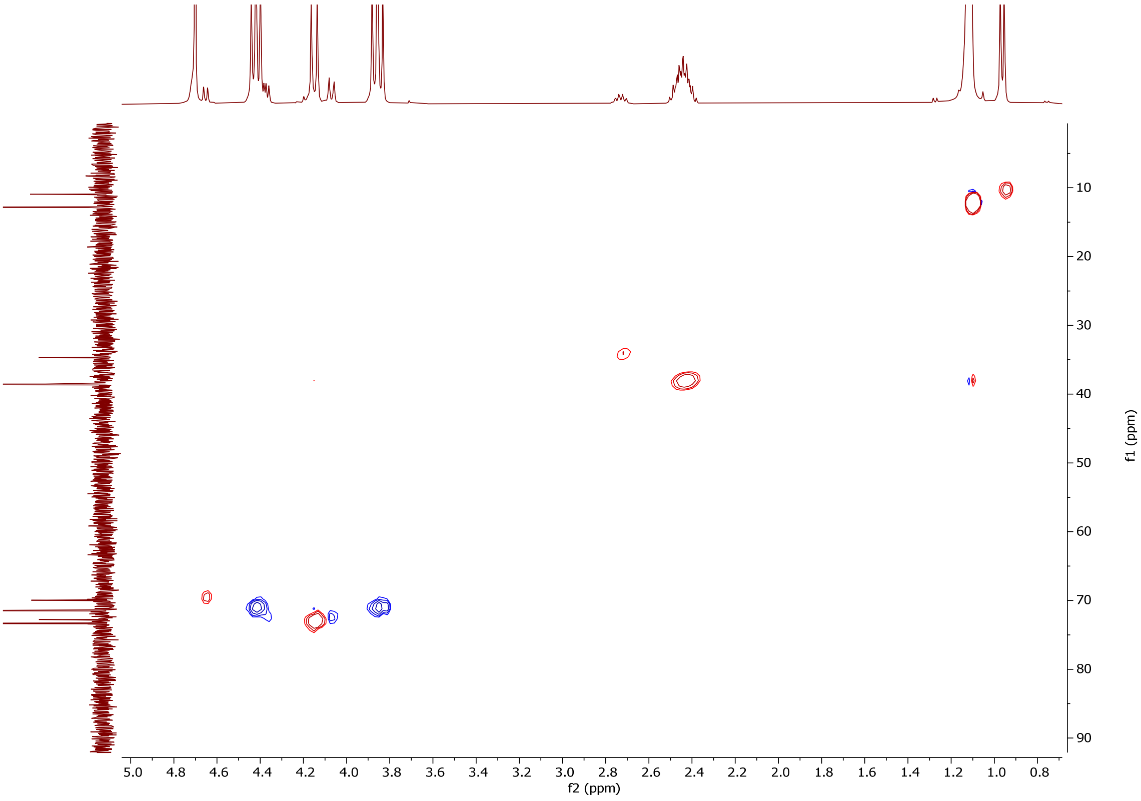
10
20
30
40
50
60
70
80
90
f1 (ppm)
5.0 4.8 4.6 4.4 4.2 4.0 3.8 3.6 3.4 3.2 3.0 2.8 2.6 2.4 2.2 2.0 1.8 1.6 1.4 1.2 1.0 0.8
f2 (ppm)

e)

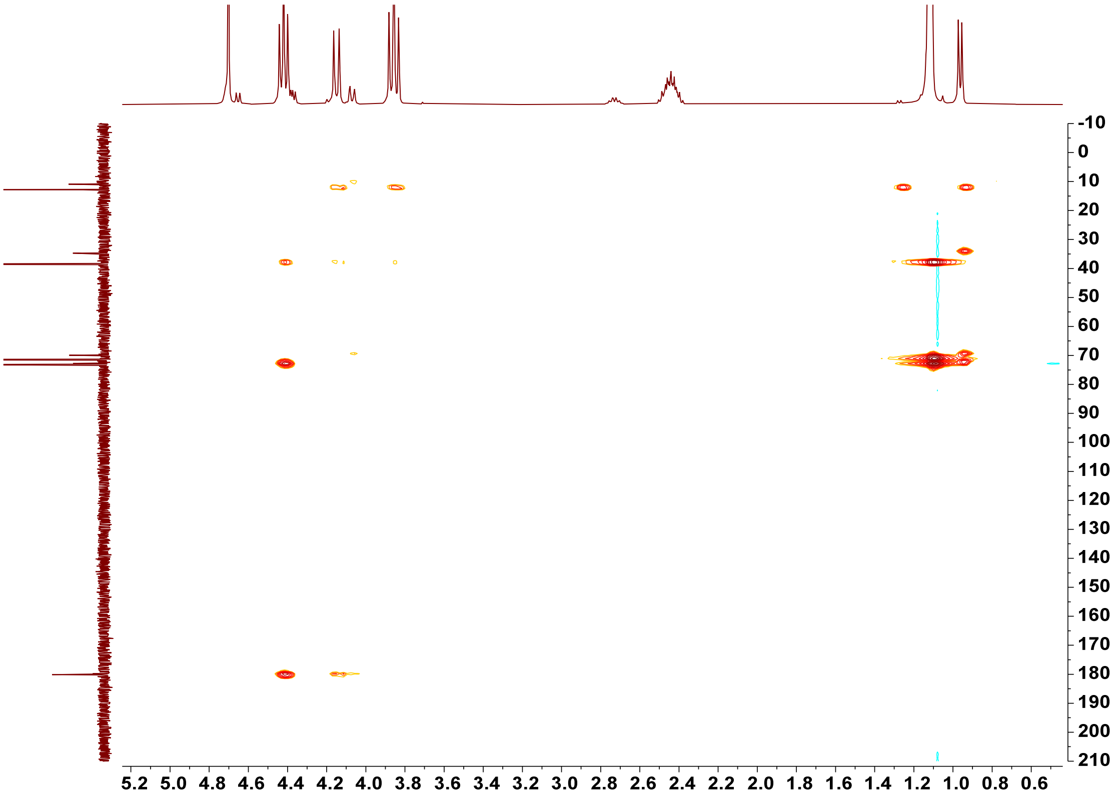

Figure S55. NMR spectra (D_2O) of 2*S*,3*R*-**5b**: a) ^{1}H, b) ^{13}C, c) COSY, d) HSQC and e) HMBC.

a)

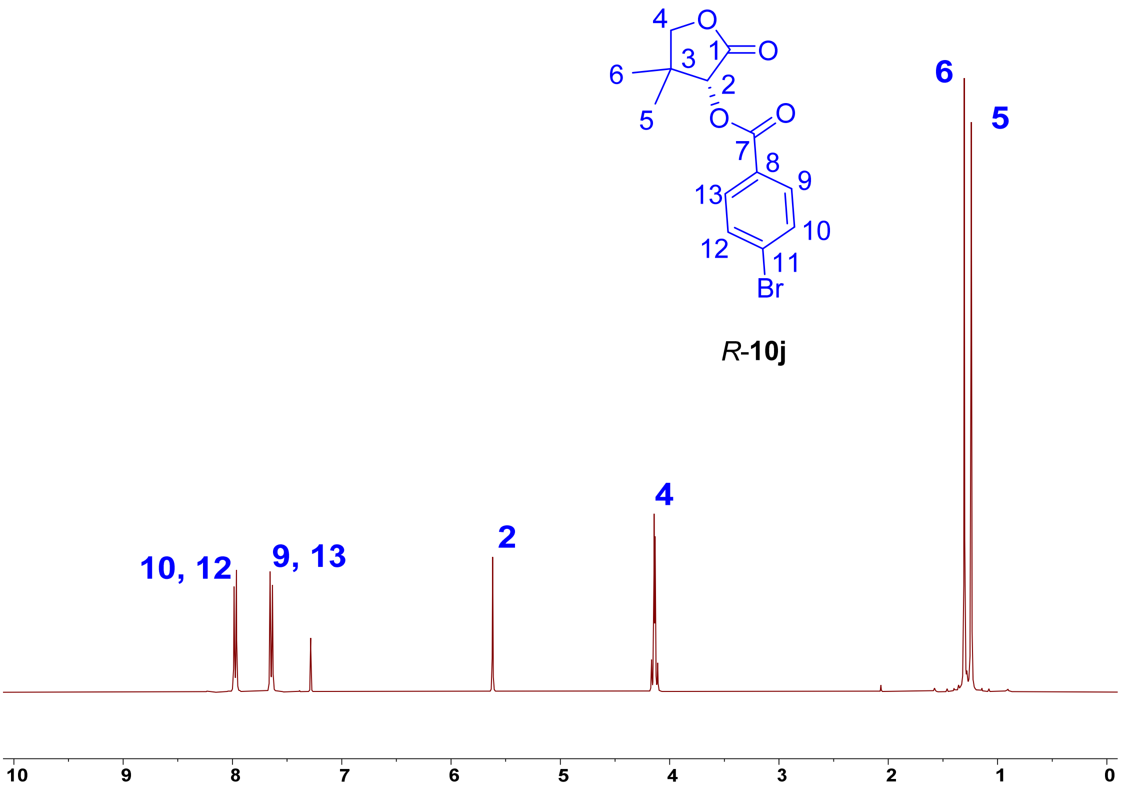

b)

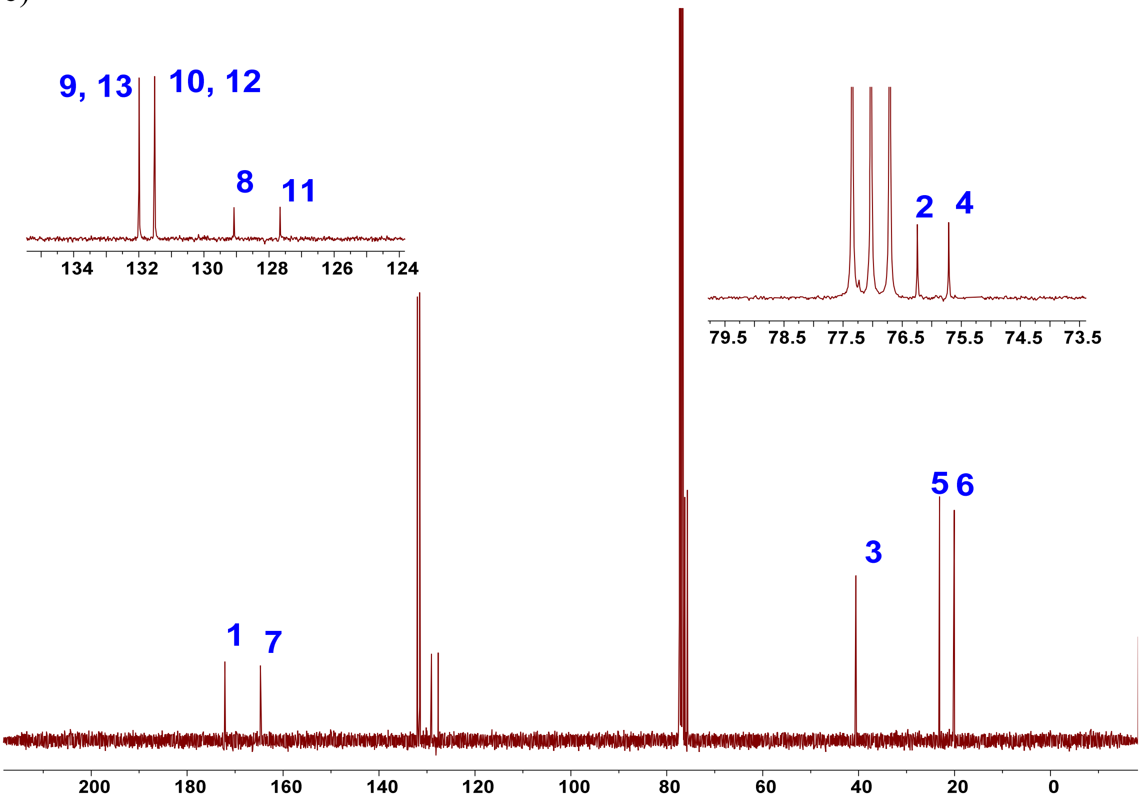

c)

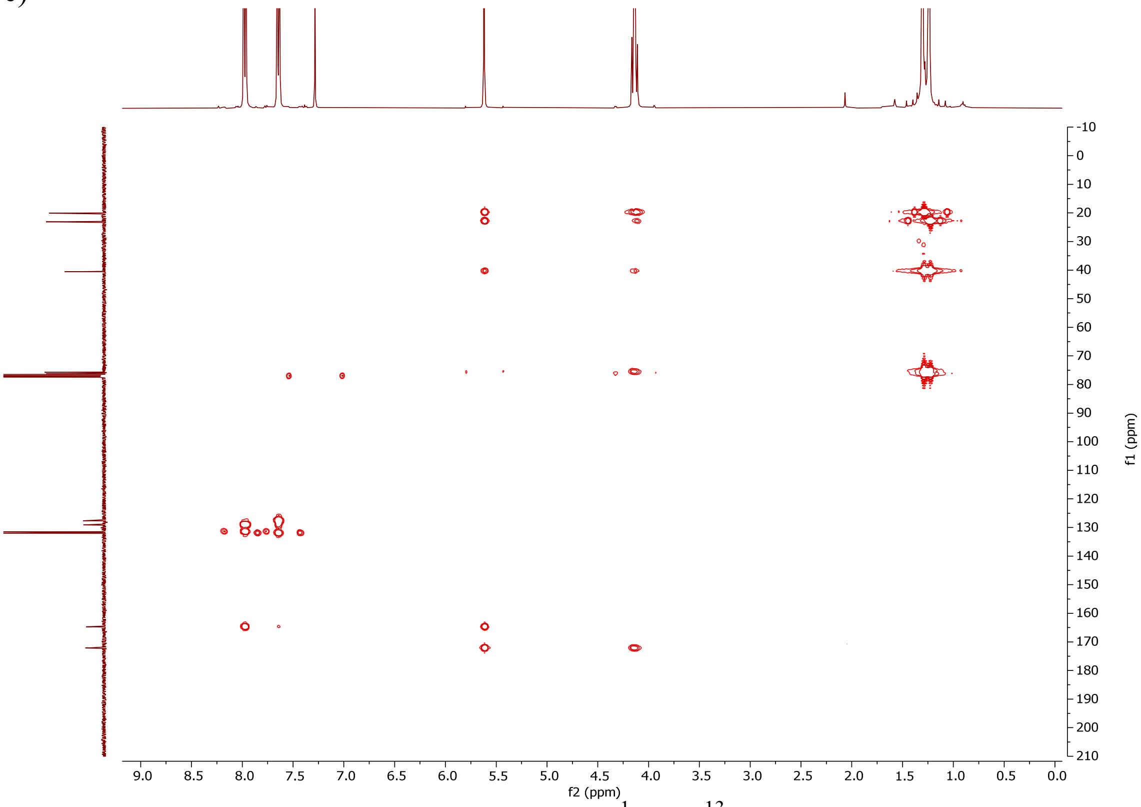

Figure S56. NMR spectra ($CDCl_3$) of *R*-**10j**: a) ^{1}H, b) ^{13}C and COSY.

a)

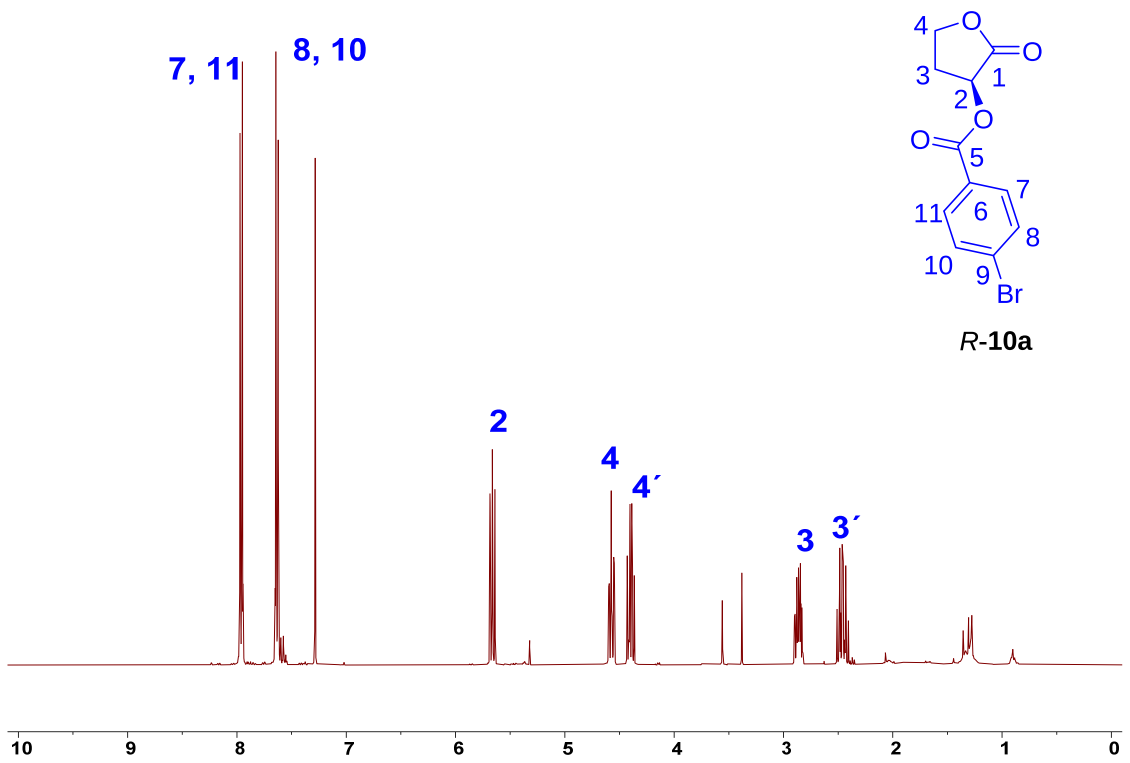
7, 11
8, 10
2
4
4´
3
3´
4
O
O
3
1
2
O
O
5
7
11
6
8
10
9
Br
R-**10a**
10 9 8 7 6 5 4 3 2 1 0

b)

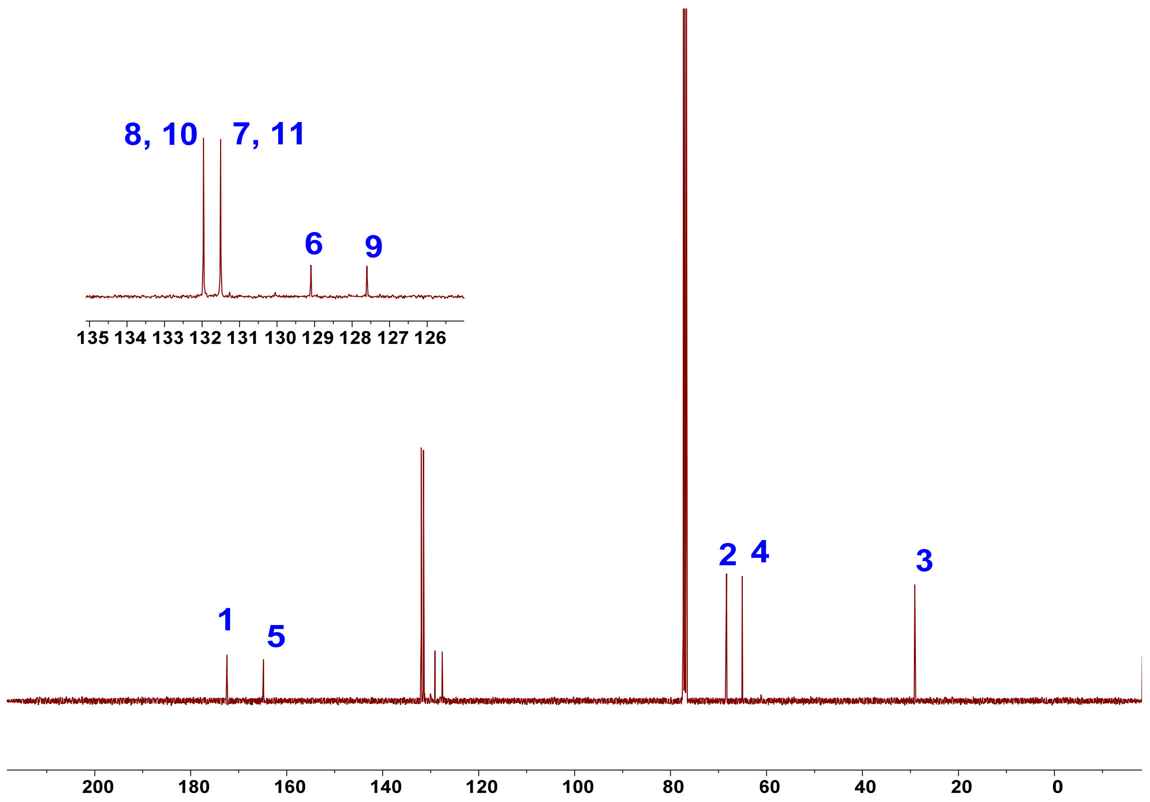
8, 10
7, 11
6
9
135 134 133 132 131 130 129 128 127 126
1
5
2 4
3
200 180 160 140 120 100 80 60 40 20 0

c)

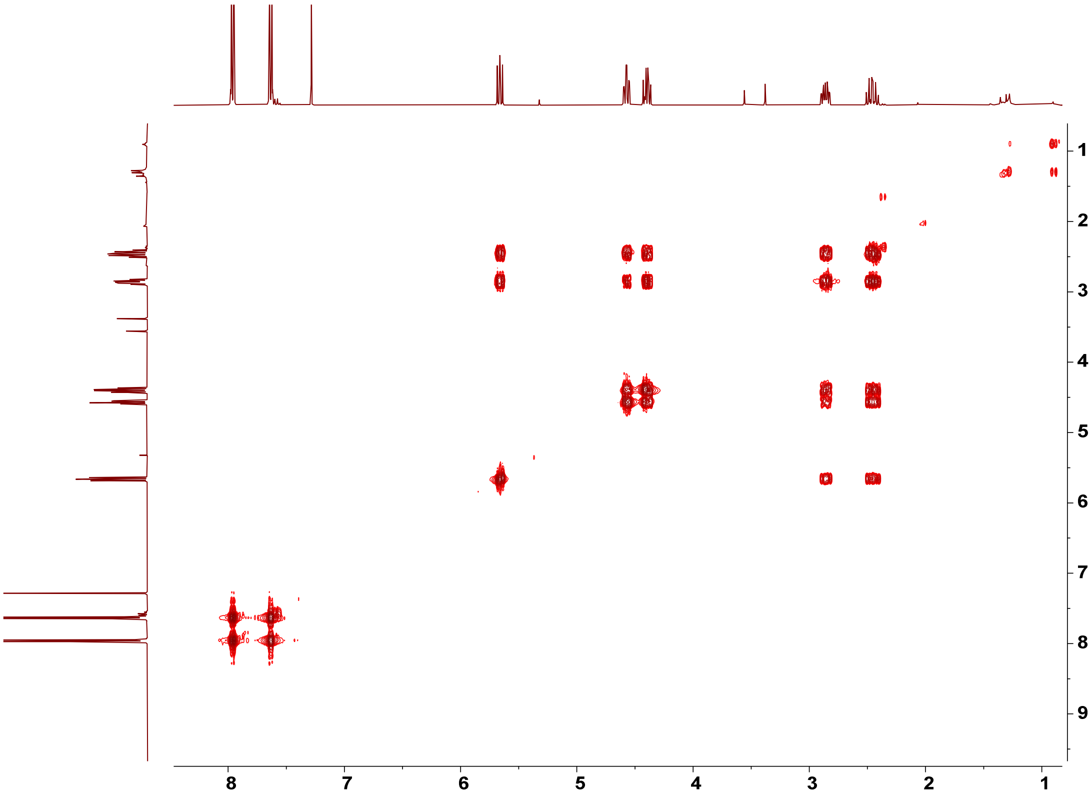

d)

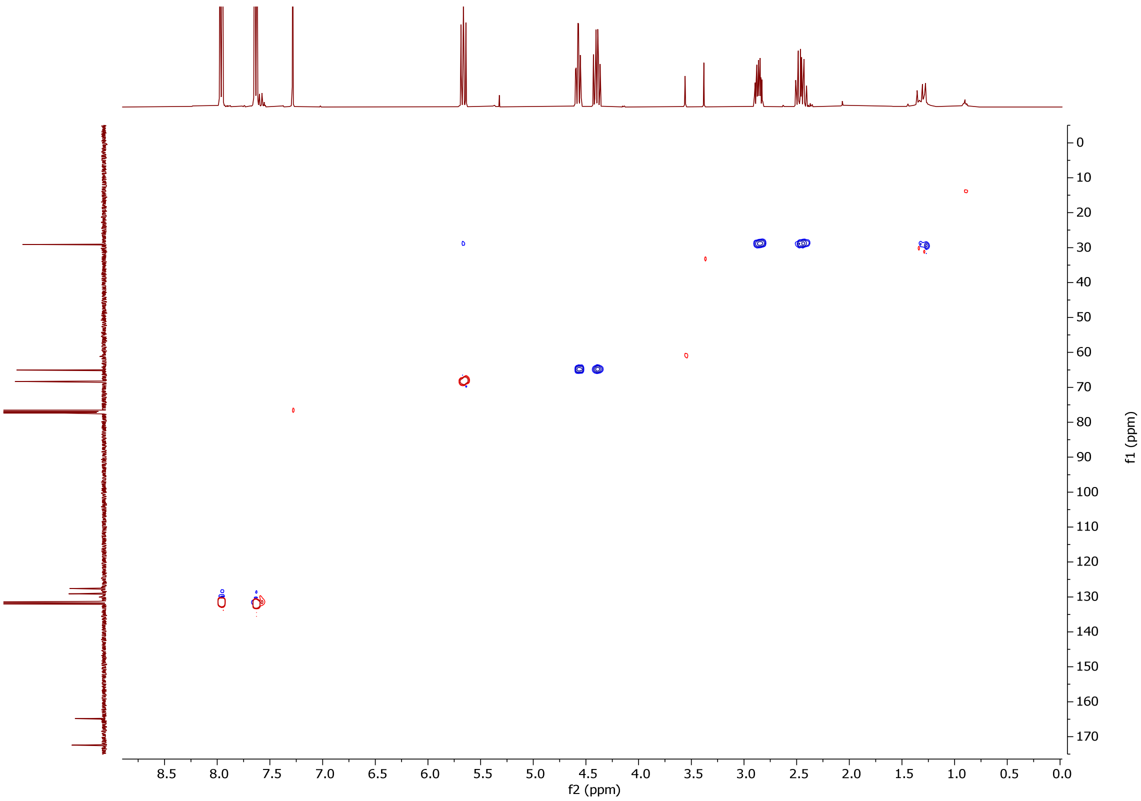

Figure S57. NMR spectra ($CDCl_3$) of *R*-**10a**: a) ^{1}H, b) ^{13}C, c) COSY and HSQC.

a)

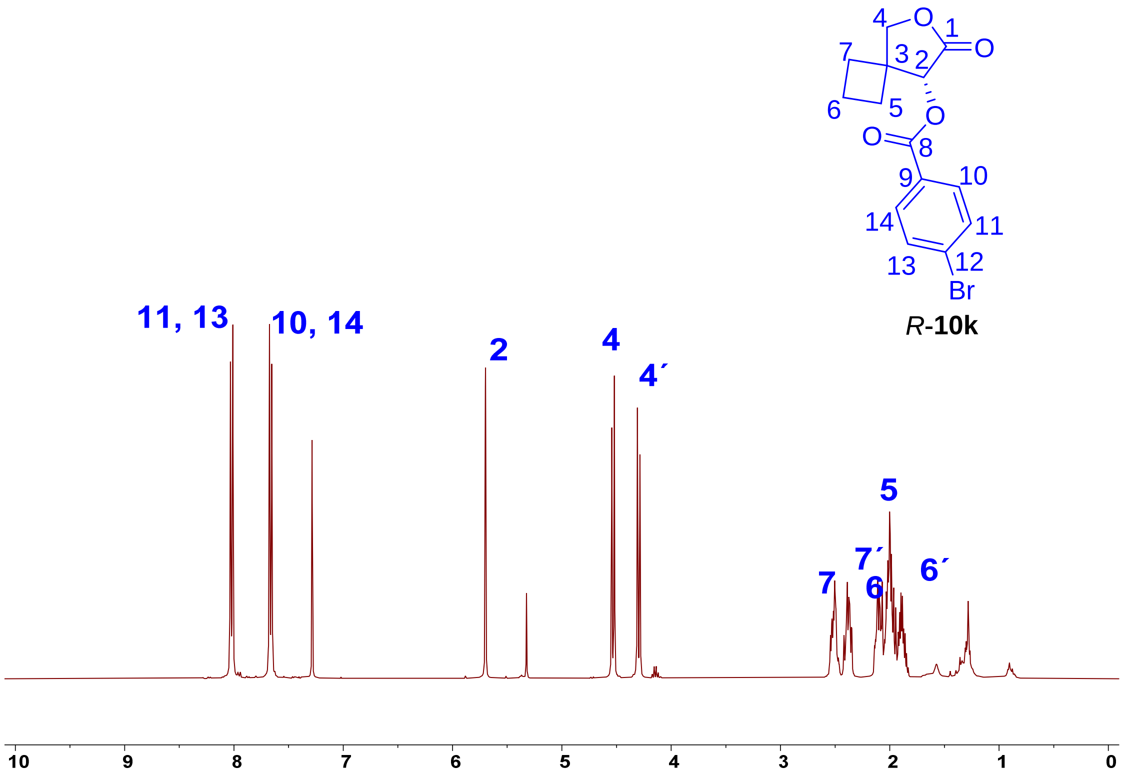
11, 13
10, 14
2
4
4´
5
7´
7
6
6´
R-10k
10
9
8
7
6
5
4
3
2
1
0

b)

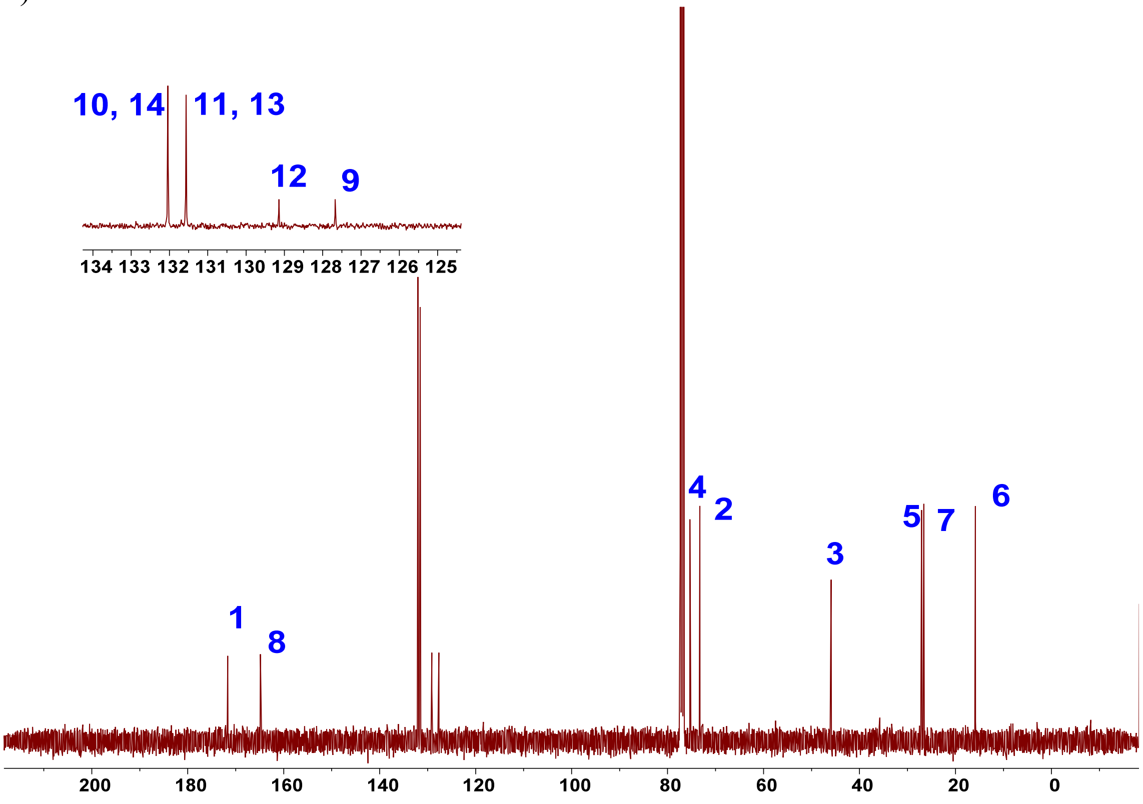
10, 14
11, 13
12
9
134 133 132 131 130 129 128 127 126 125
1
8
4
2
3
5
7
6
200
180
160
140
120
100
80
60
40
20
0

c)

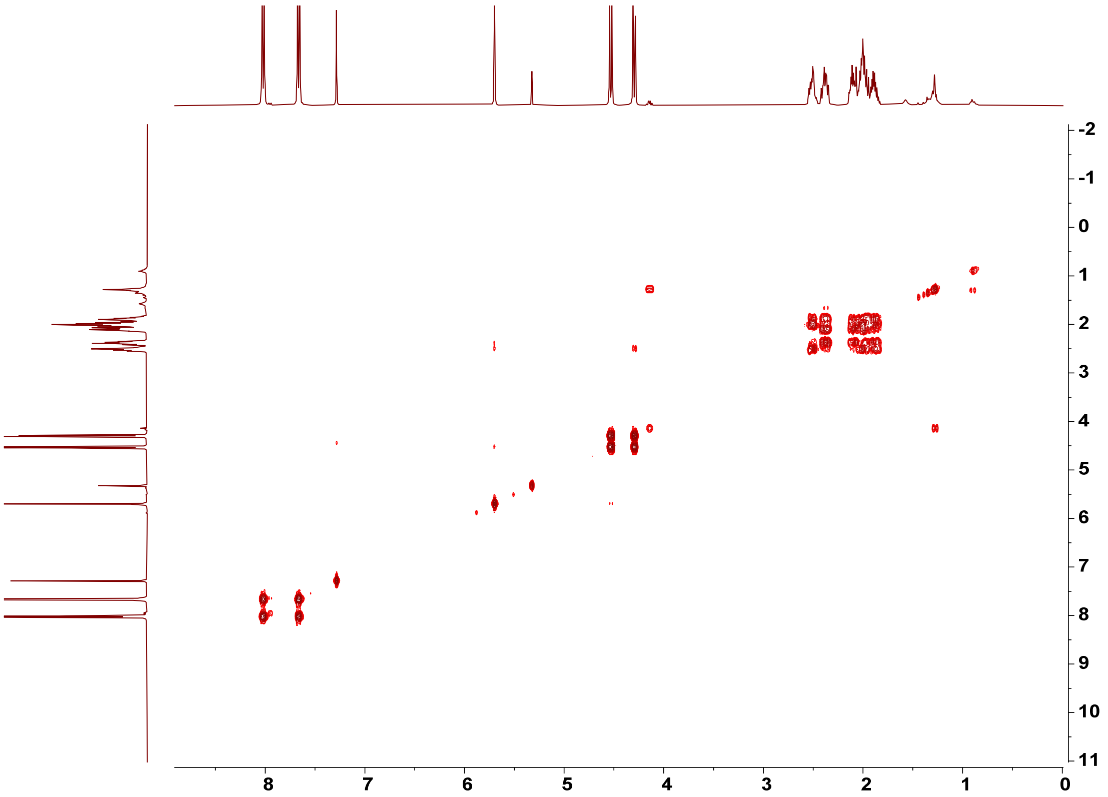

d)

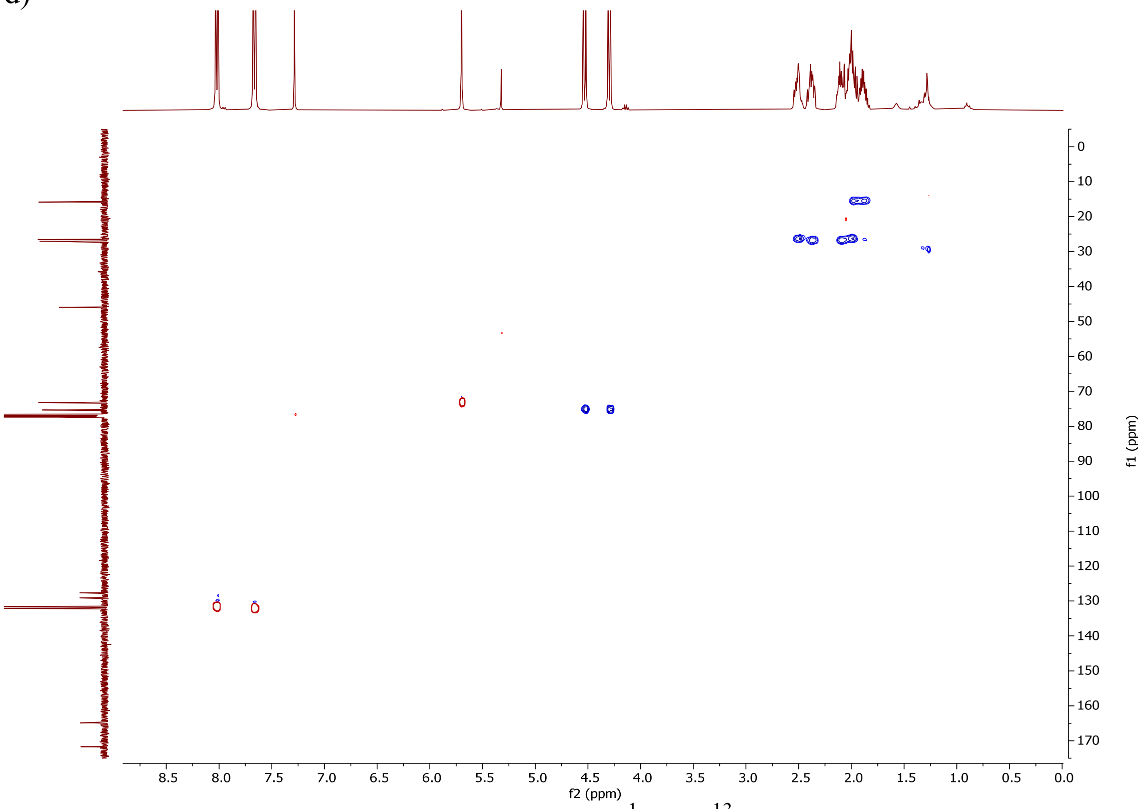

Figure S58. NMR spectra ($CDCl_3$) of *R*-**10k**: a) ^{1}H, b) ^{13}C, c) COSY and d) HSQC.

a)

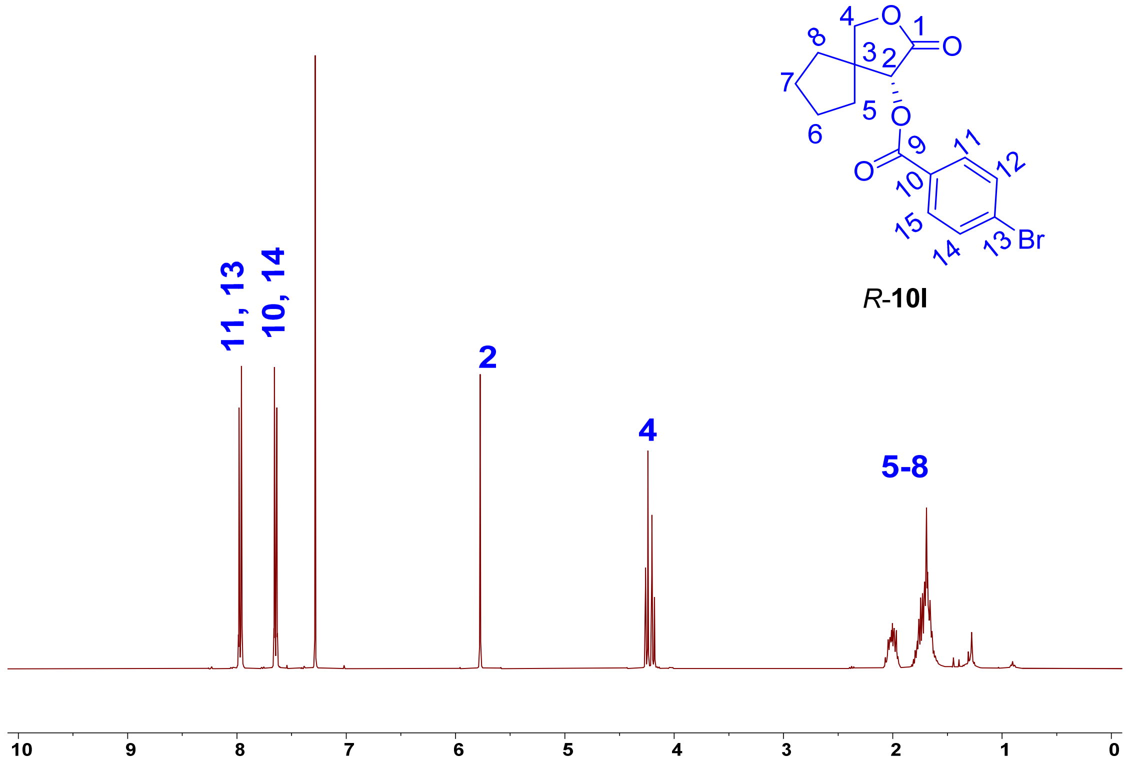
R-10l
11, 13
10, 14
2
4
5-8
10
9
8
7
6
5
4
3
2
1
0

b)

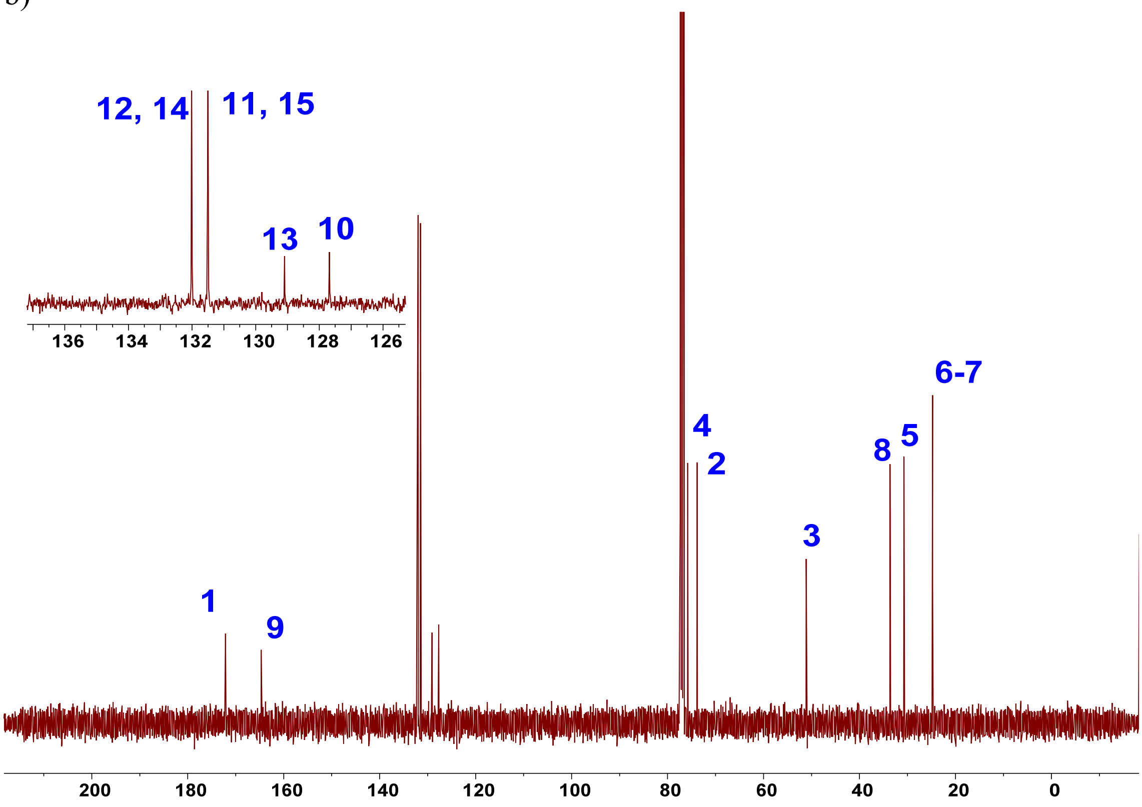
12, 14
11, 15
13
10
136
134
132
130
128
126
6-7
4
2
8
5
3
1
9
200
180
160
140
120
100
80
60
40
20
0

c)

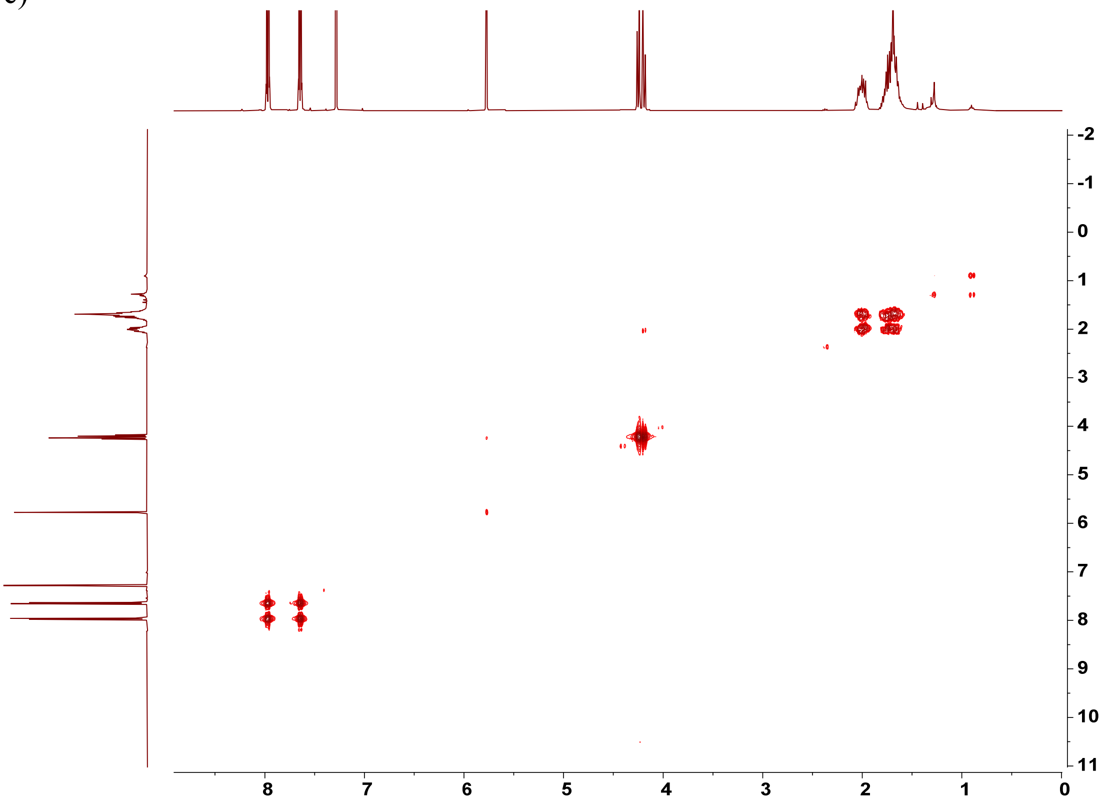

d)

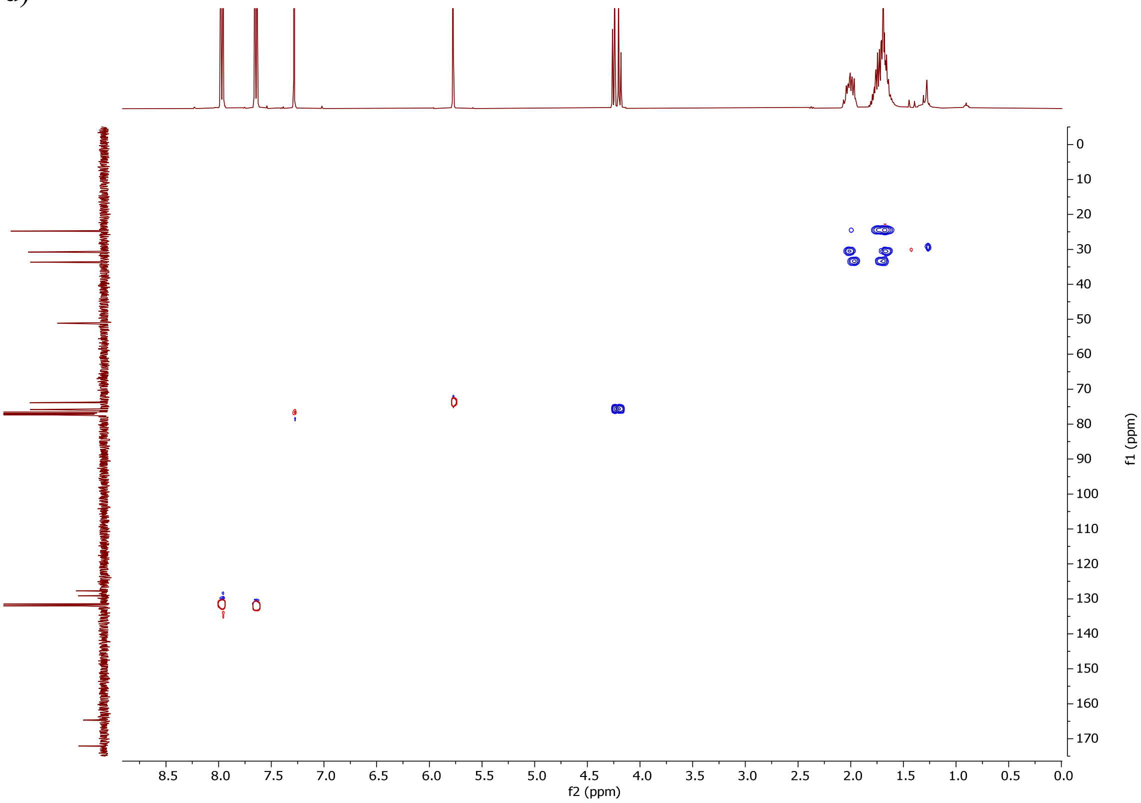

Figure S59. NMR spectra ($CDCl_3$) of *R*-**10l**: a) ^{1}H, b) ^{13}C, c) COSY and d) HSQC.

a)

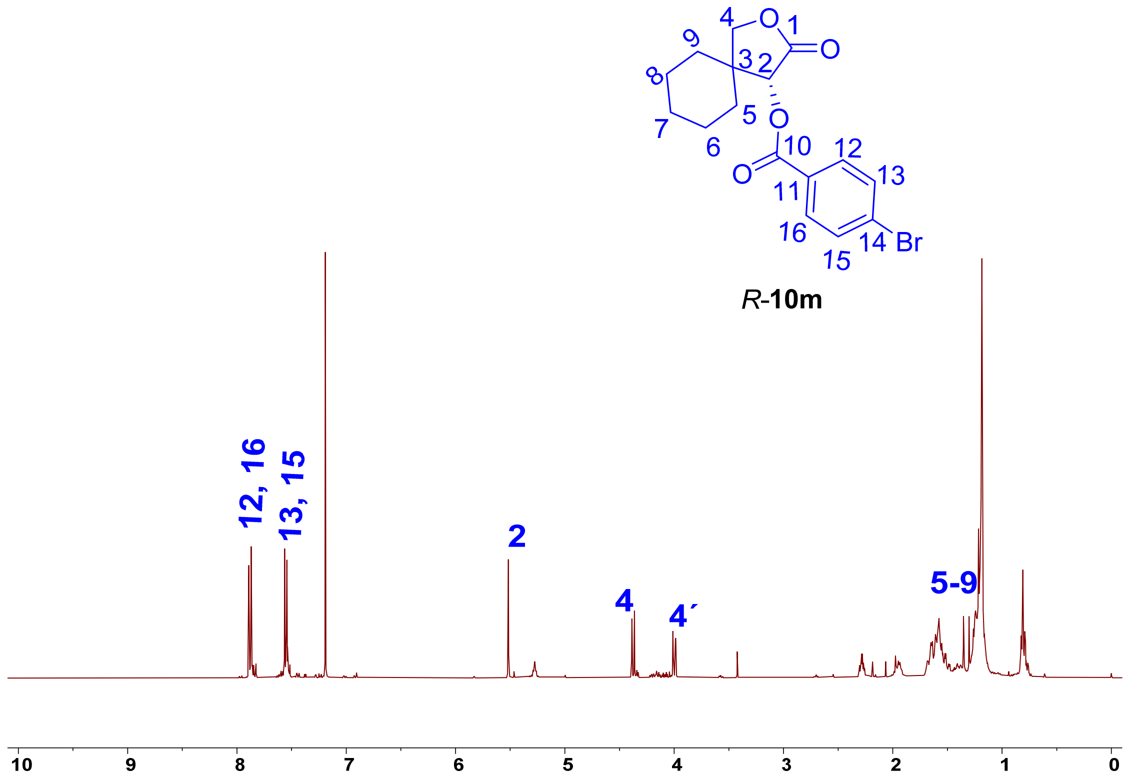

R-10m
12, 16
13, 15
2
4
4´
5-9
10
9
8
7
6
5
4
3
2
1
0

b)

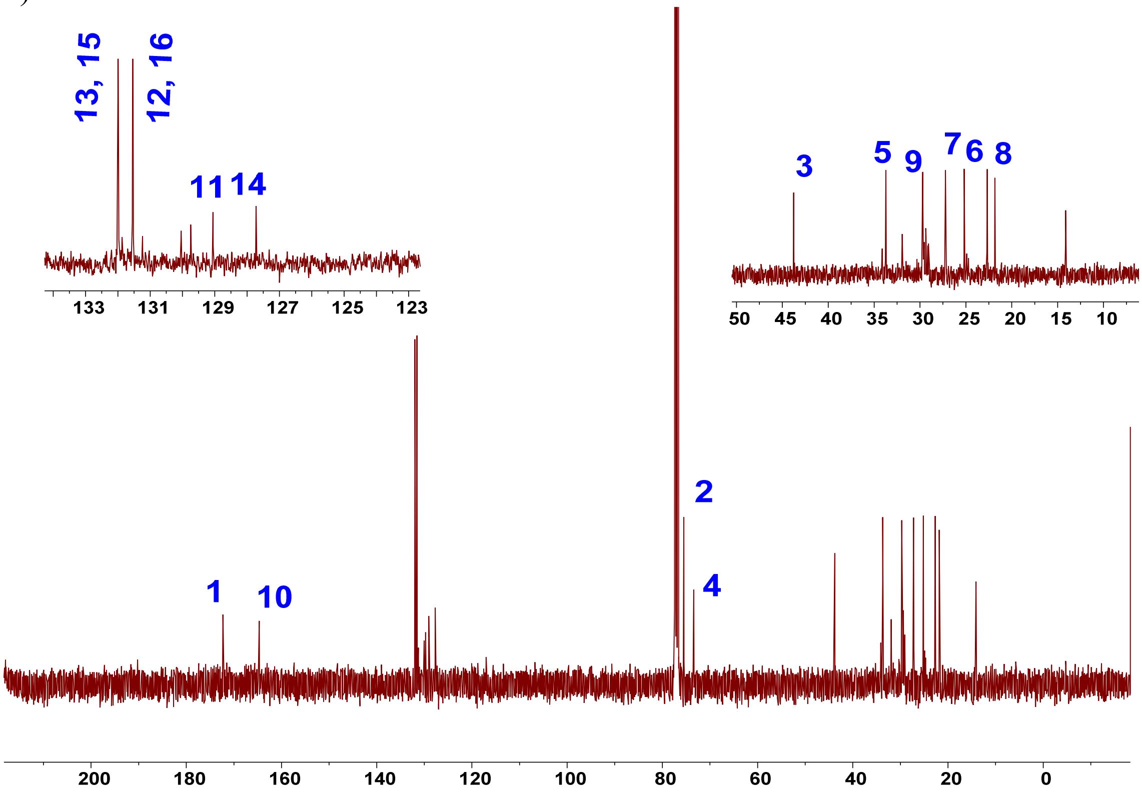

13, 15
12, 16
11
14
133
131
129
127
125
123
3
5
9
7
6
8
50
45
40
35
30
25
20
15
10
1
10
2
4
200
180
160
140
120
100
80
60
40
20
0

c)

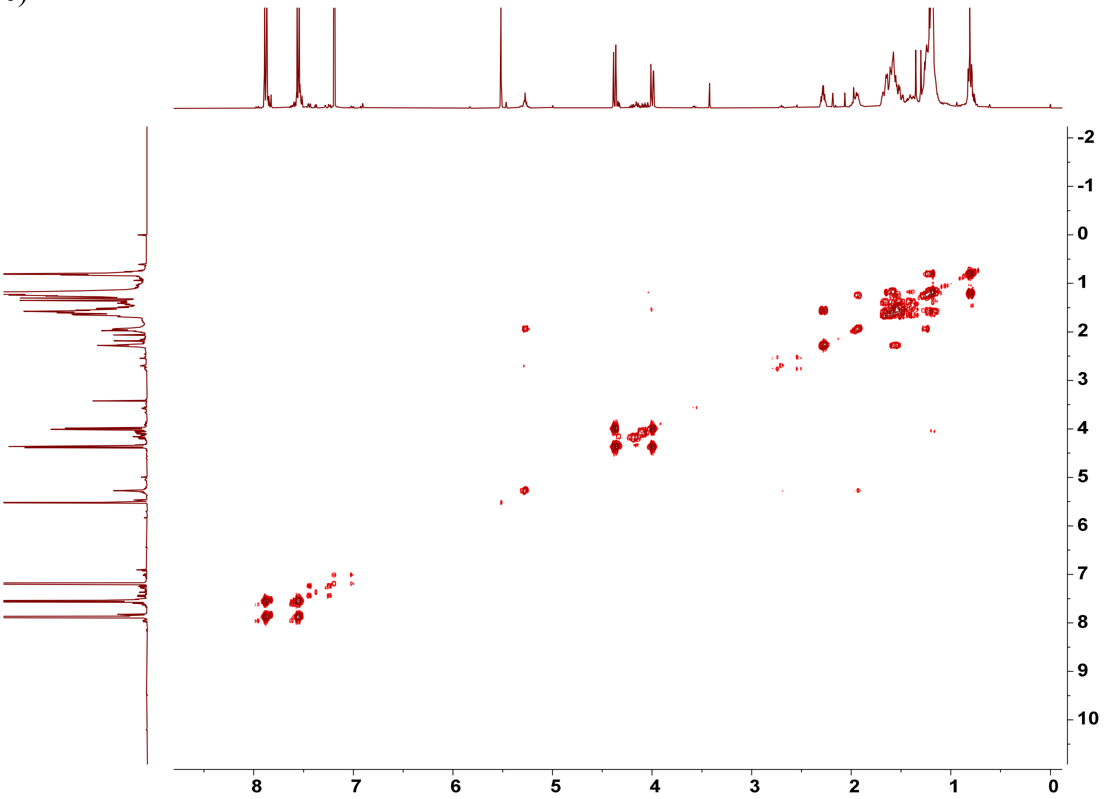

d)

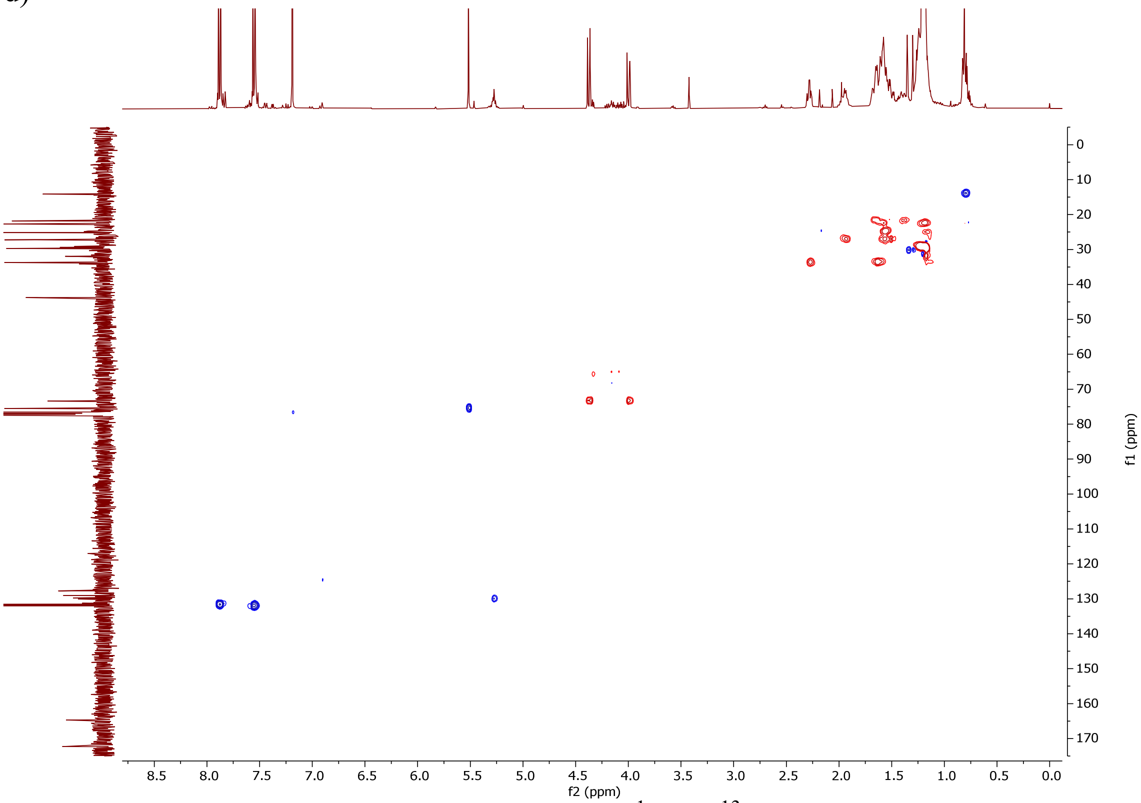

Figure S60. NMR spectra ($CDCl_3$) of *R*-**10m**: a) ^{1}H, b) ^{13}C, c) COSY and d) HSQC.

a)

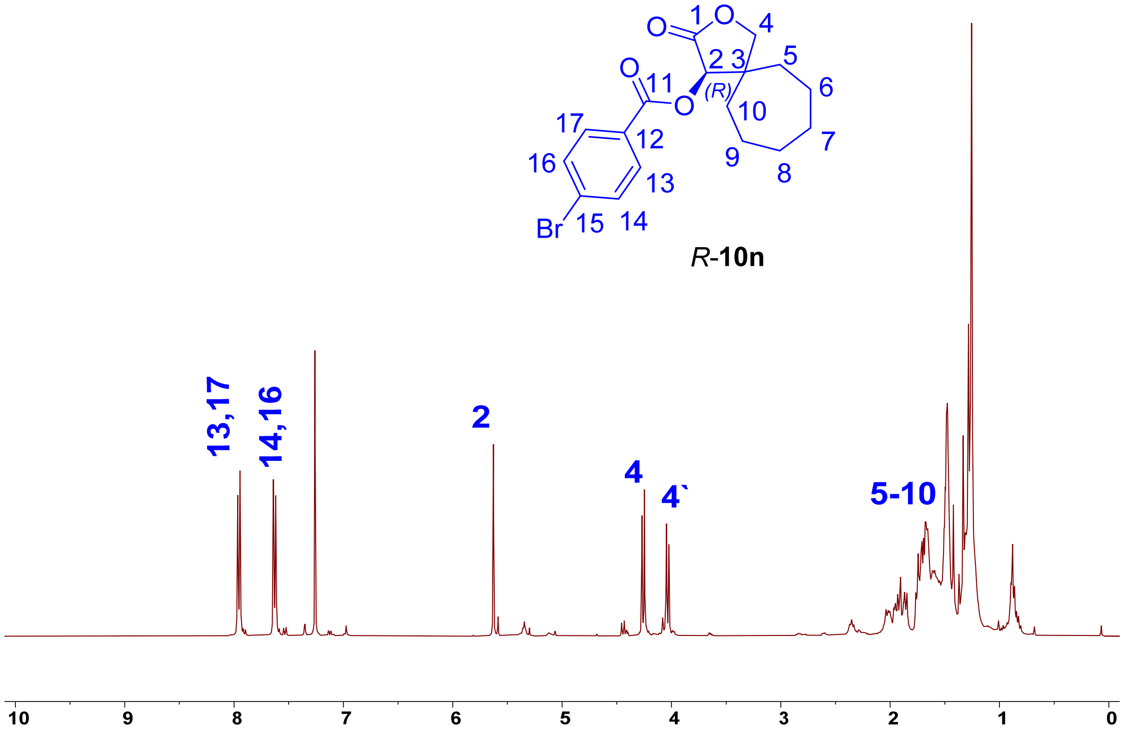

R-10n
13,17
14,16
2
4
4`
5-10
10 9 8 7 6 5 4 3 2 1 0

b)

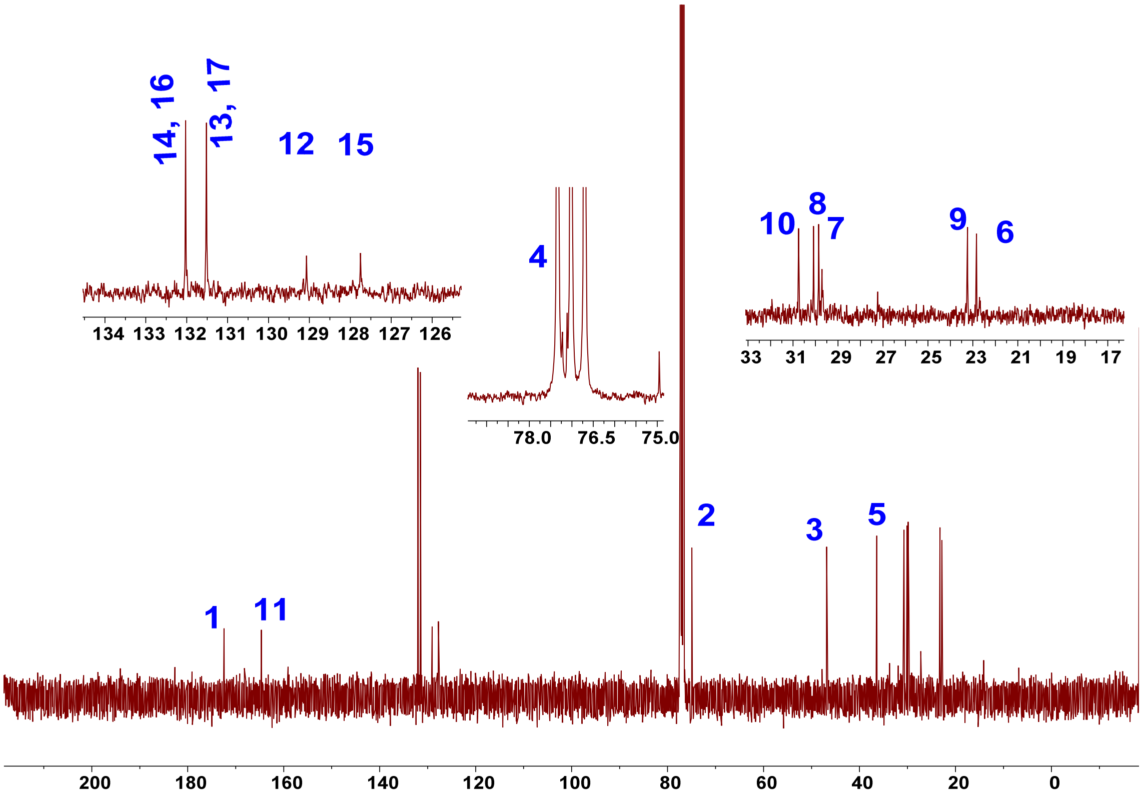

14, 16
13, 17
12
15
134 133 132 131 130 129 128 127 126
4
78.0 76.5 75.0
10
8
7
9
6
33 31 29 27 25 23 21 19 17
1
11
2
3
5
200 180 160 140 120 100 80 60 40 20 0

c)

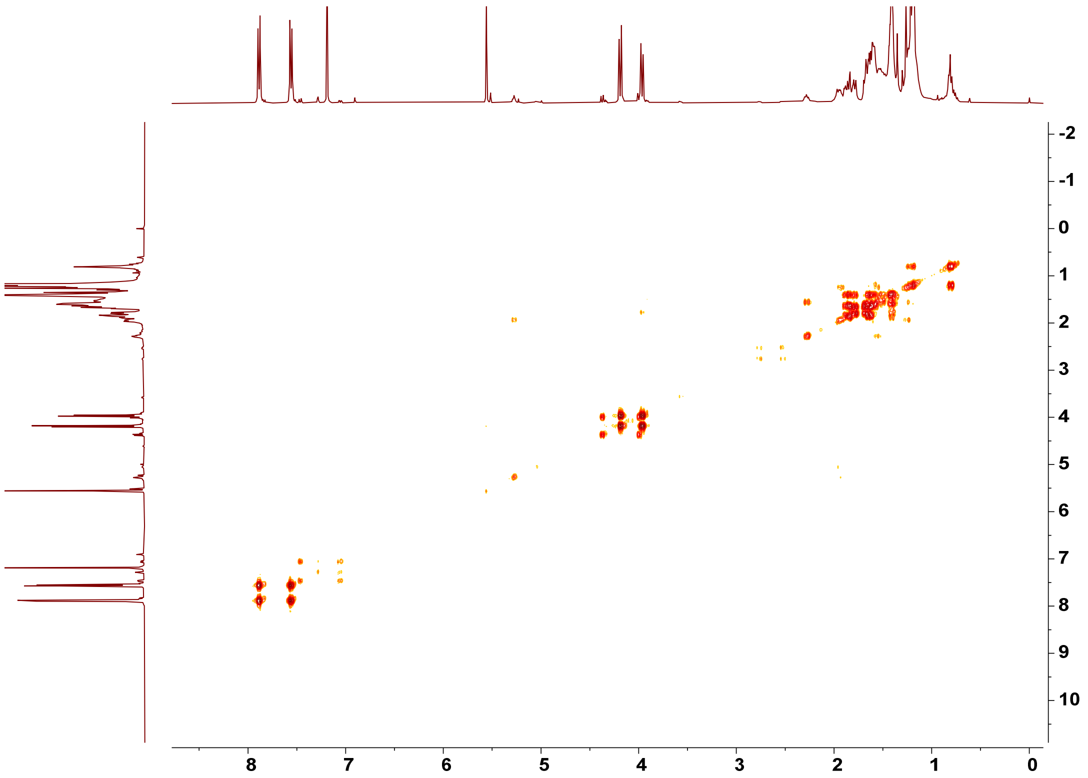

d)

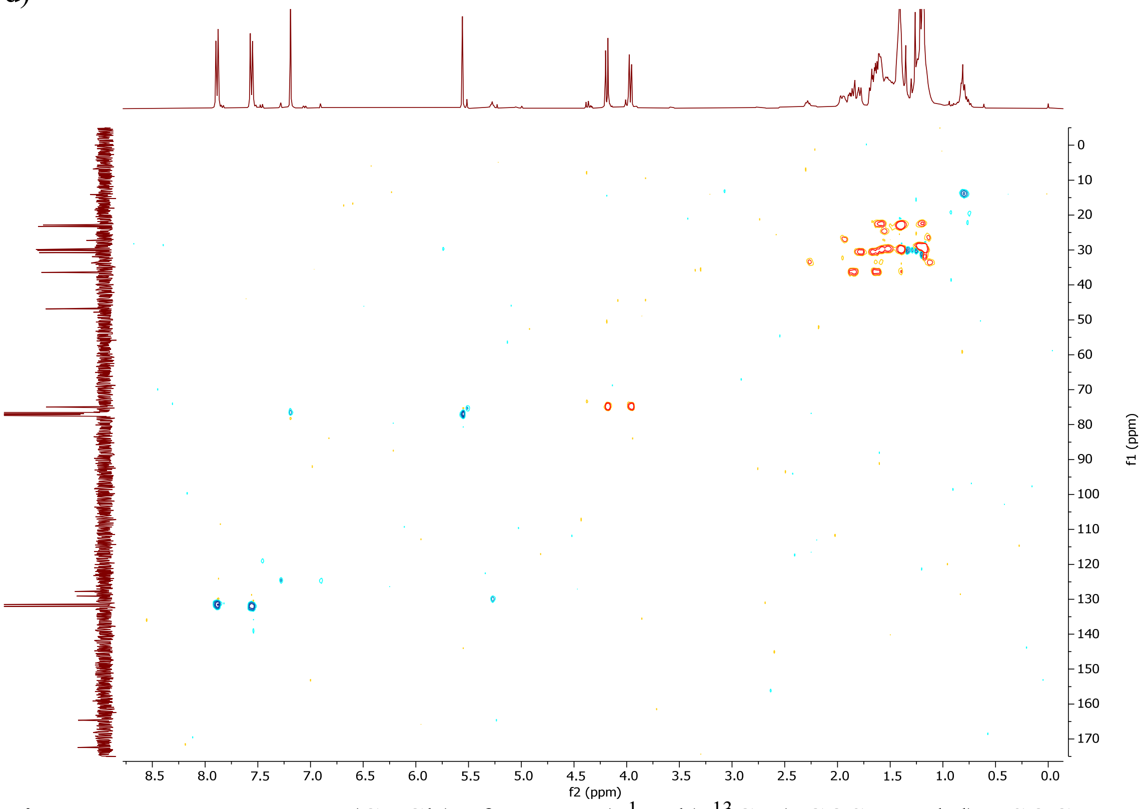

Figure S61. NMR spectra ($CDCl_3$) of *R*-**10n**: a) ^{1}H, b) ^{13}C, c) COSY and d) HSQC.

a)

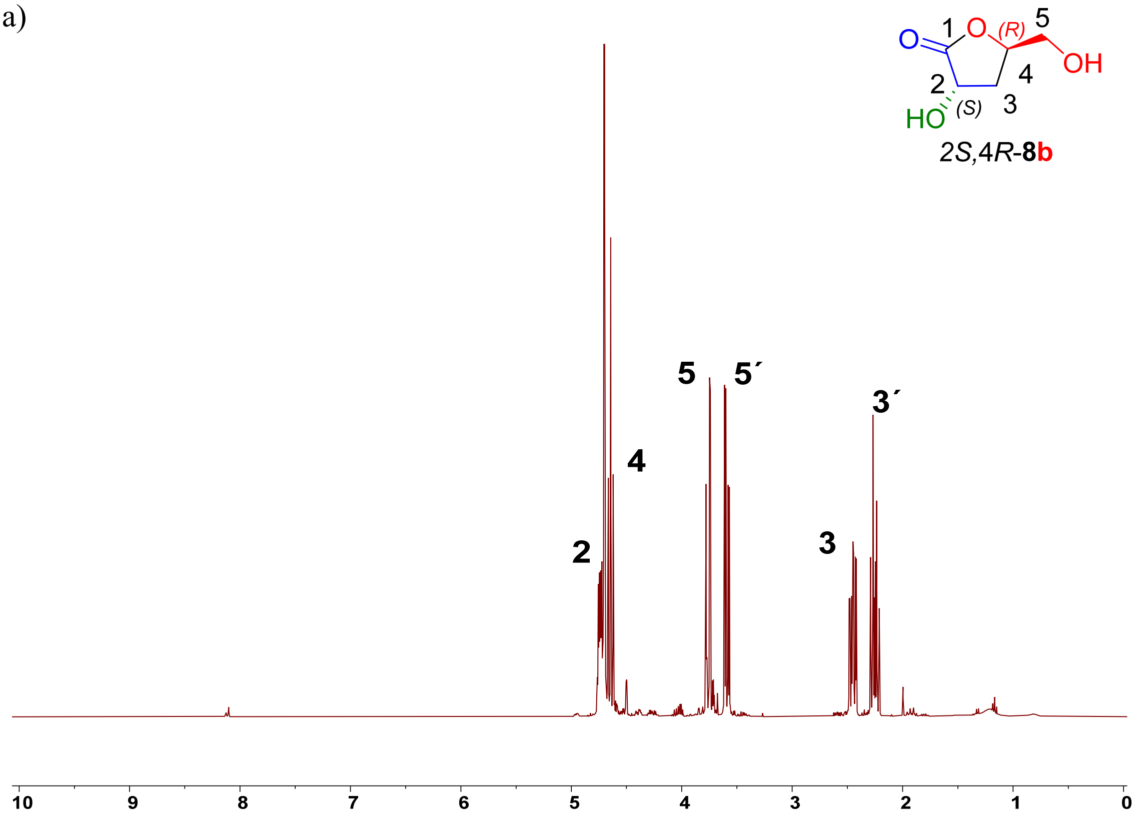
1
O
(R)
5
OH
2
4
HO
(S)
3
2S,4R-8b
5
5´
4
2
3
3´
10
9
8
7
6
5
4
3
2
1
0

b)

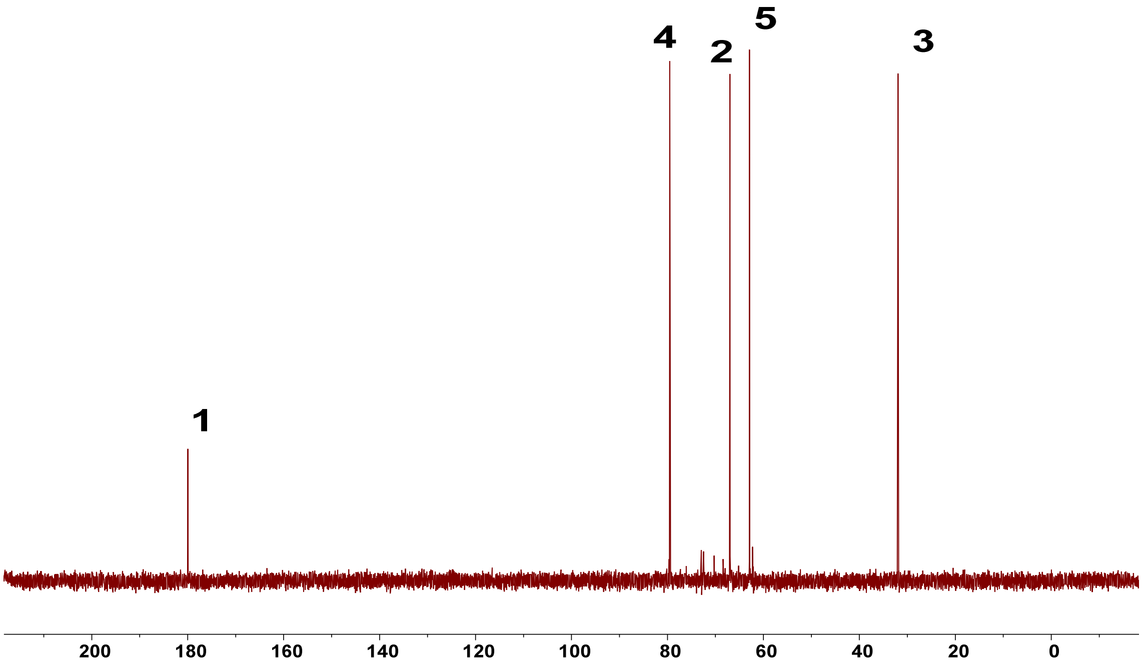
4
2
5
3
1
200
180
160
140
120
100
80
60
40
20
0

c)

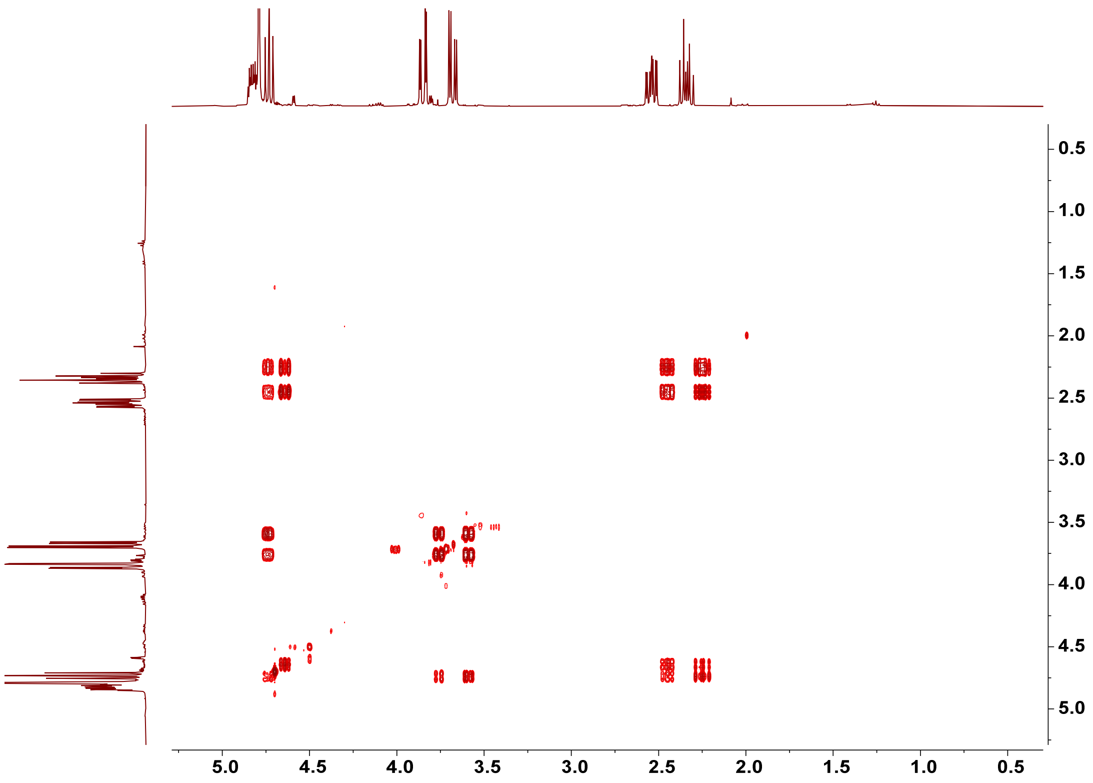

0.5
1.0
1.5
2.0
2.5
3.0
3.5
4.0
4.5
5.0
5.0 4.5 4.0 3.5 3.0 2.5 2.0 1.5 1.0 0.5

d)

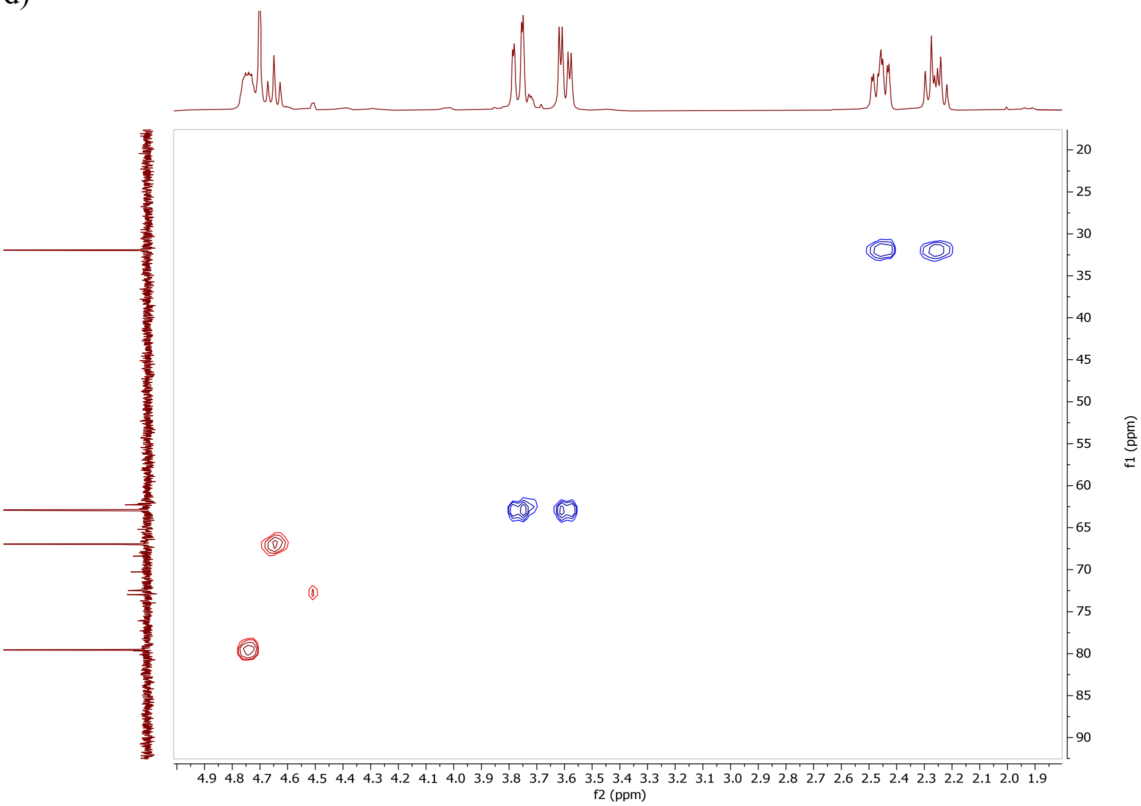

20
25
30
35
40
45
50
55
60
65
70
75
80
85
90
f1 (ppm)
4.9 4.8 4.7 4.6 4.5 4.4 4.3 4.2 4.1 4.0 3.9 3.8 3.7 3.6 3.5 3.4 3.3 3.2 3.1 3.0 2.9 2.8 2.7 2.6 2.5 2.4 2.3 2.2 2.1 2.0 1.9
f2 (ppm)

e)

f)

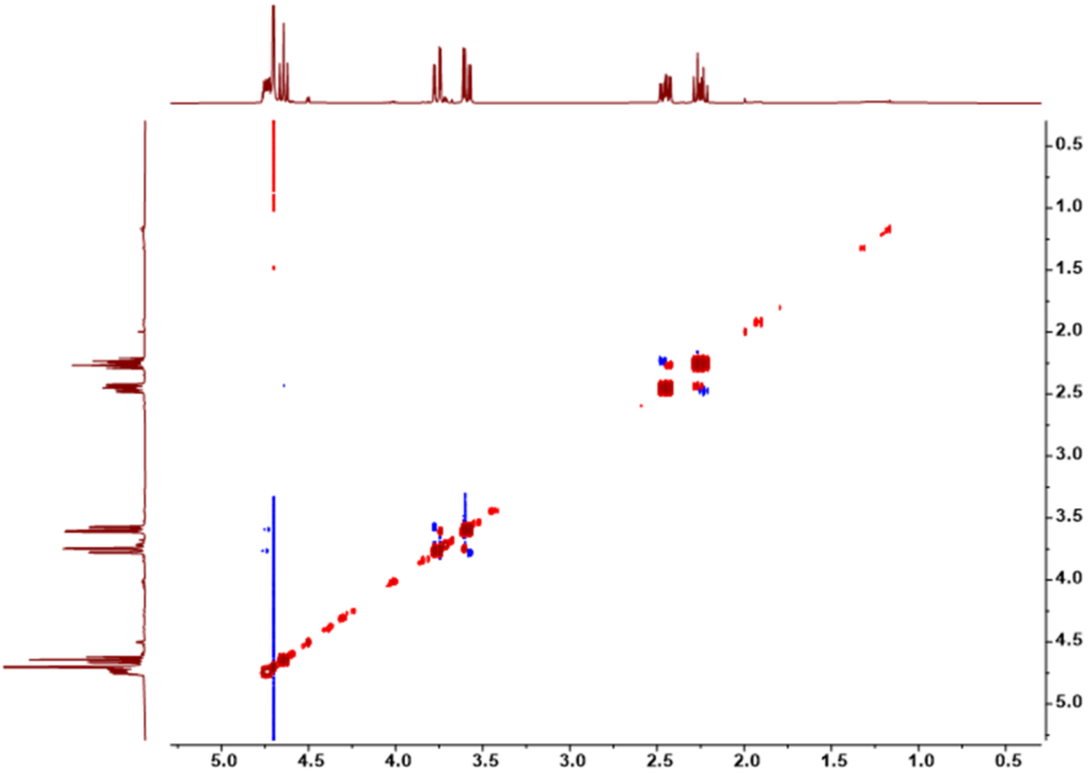

Figure S62. NMR spectra (D_2O) of 2*S*,4*R*-**8b**: a) ^{1}H, b) ^{13}C, c) COSY, d) HSQC, e) HMBC and f) NOESY.

a)

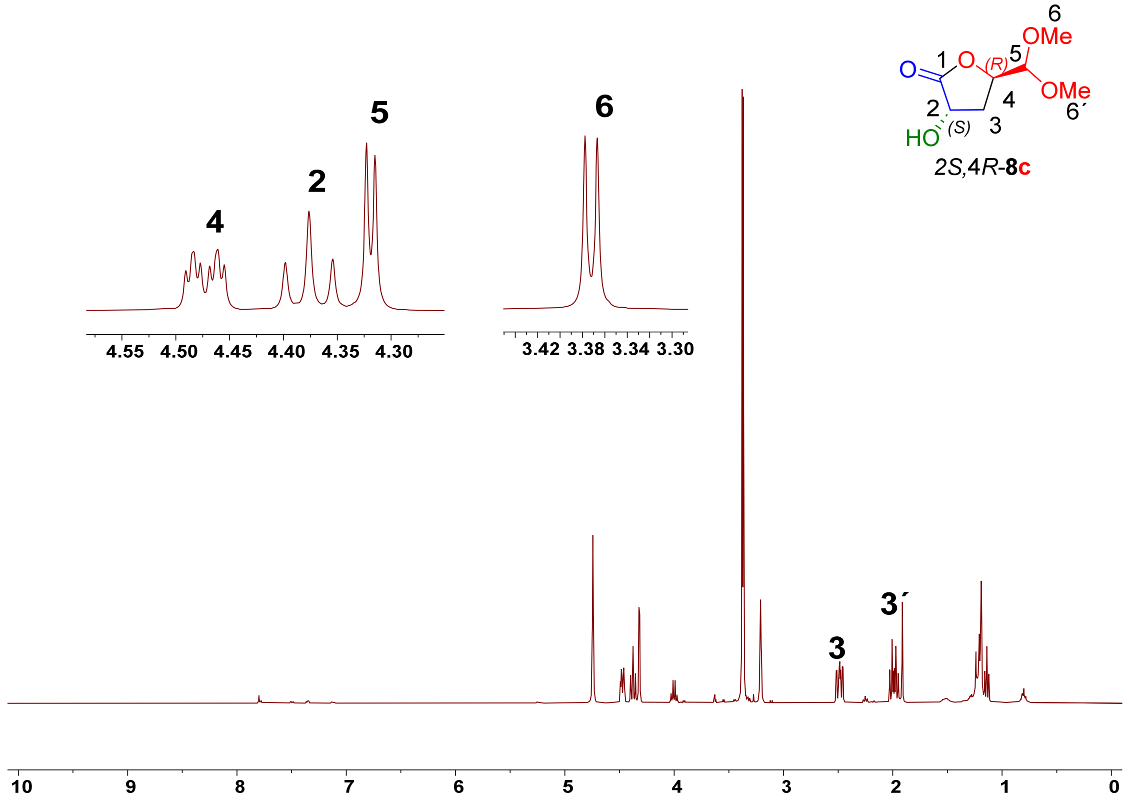

6
OMe
5
1
O
(R)
O
4
OMe
6´
2
(S)
3
HO
2S,4R-8c
4
2
5
6
4.55 4.50 4.45 4.40 4.35 4.30
3.42 3.38 3.34 3.30
3
3´
10 9 8 7 6 5 4 3 2 1 0

b)

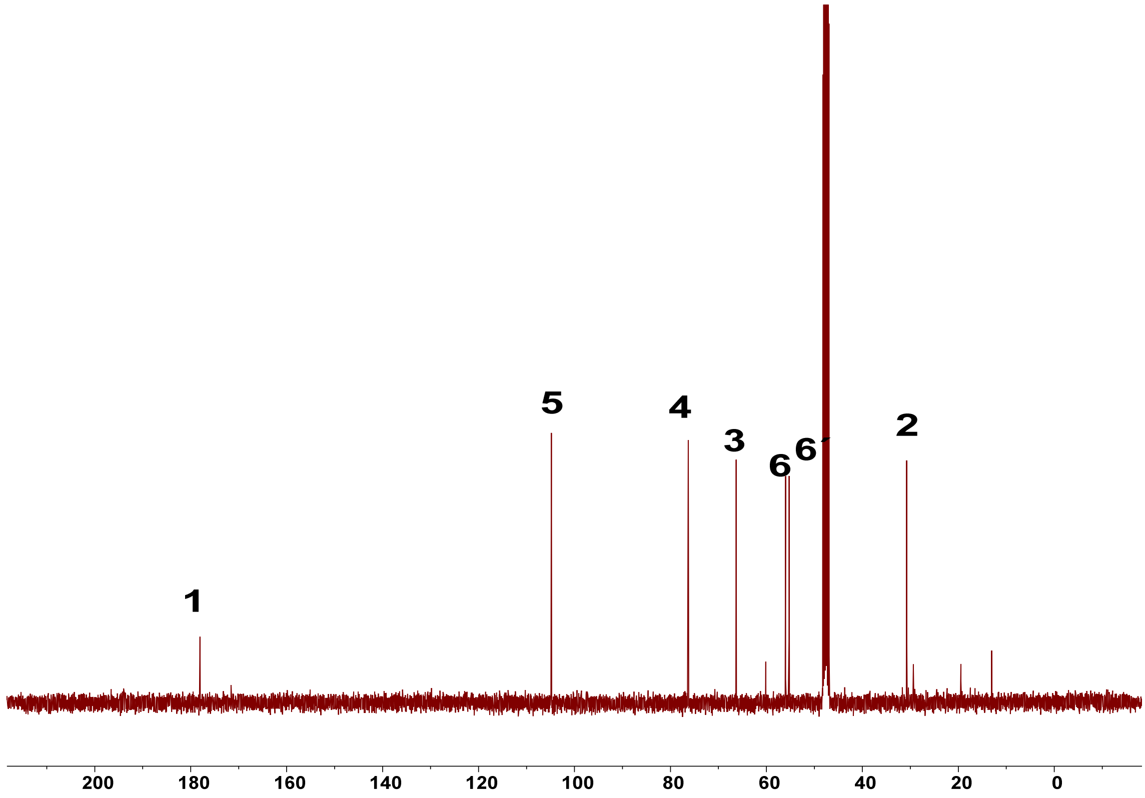

5
4
3
6
6´
2
1
200 180 160 140 120 100 80 60 40 20 0

c)

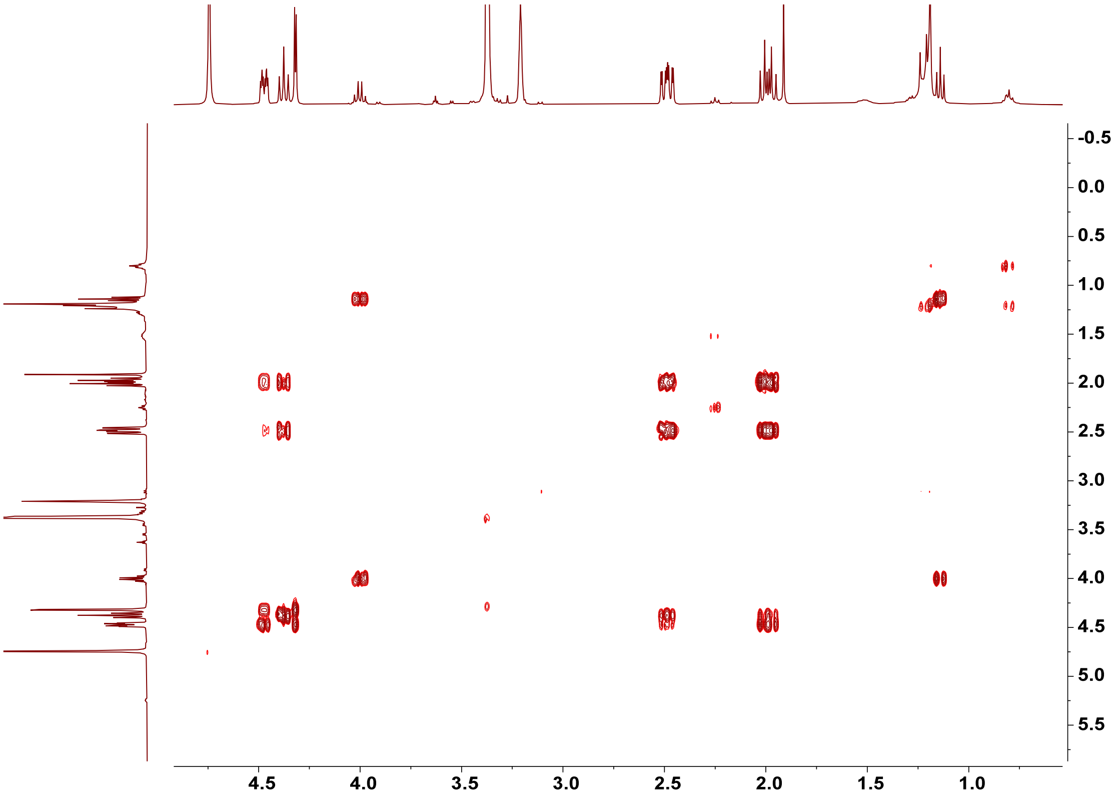
-0.5
0.0
0.5
1.0
1.5
2.0
2.5
3.0
3.5
4.0
4.5
5.0
5.5
4.5
4.0
3.5
3.0
2.5
2.0
1.5
1.0

d)

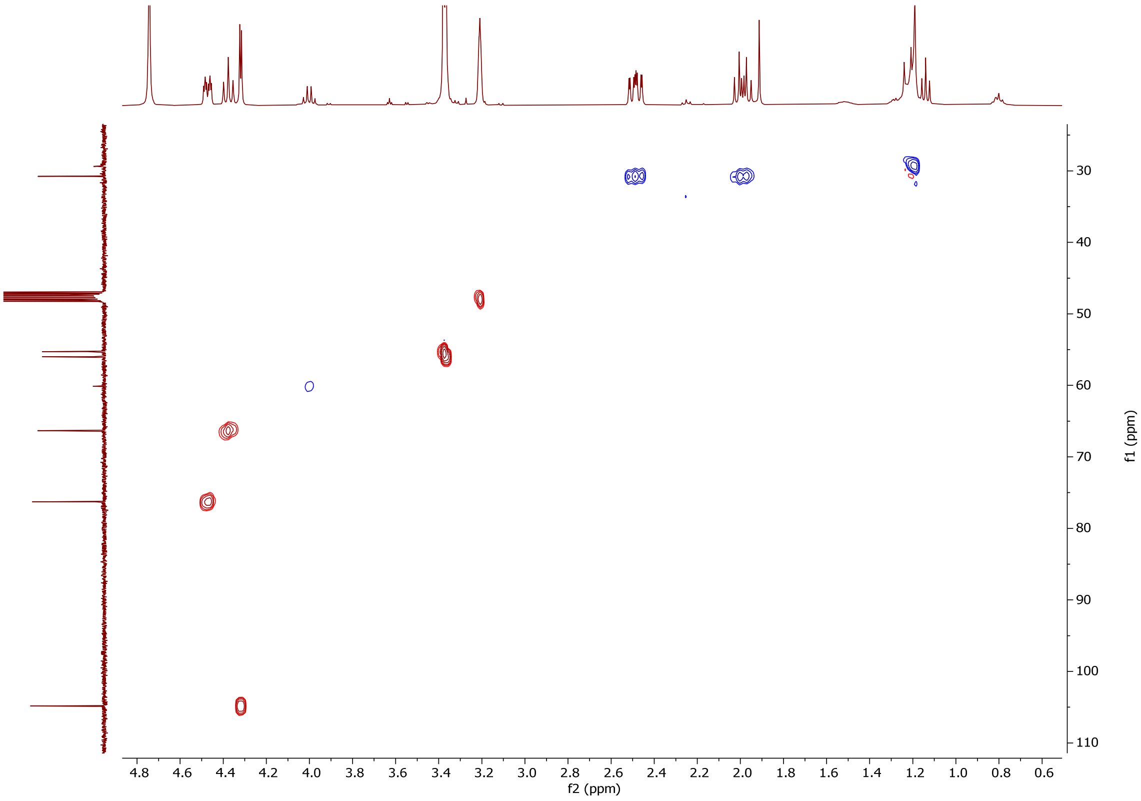
30
40
50
60
70
80
90
100
110
f1 (ppm)
4.8
4.6
4.4
4.2
4.0
3.8
3.6
3.4
3.2
3.0
2.8
2.6
2.4
2.2
2.0
1.8
1.6
1.4
1.2
1.0
0.8
0.6
f2 (ppm)

e)

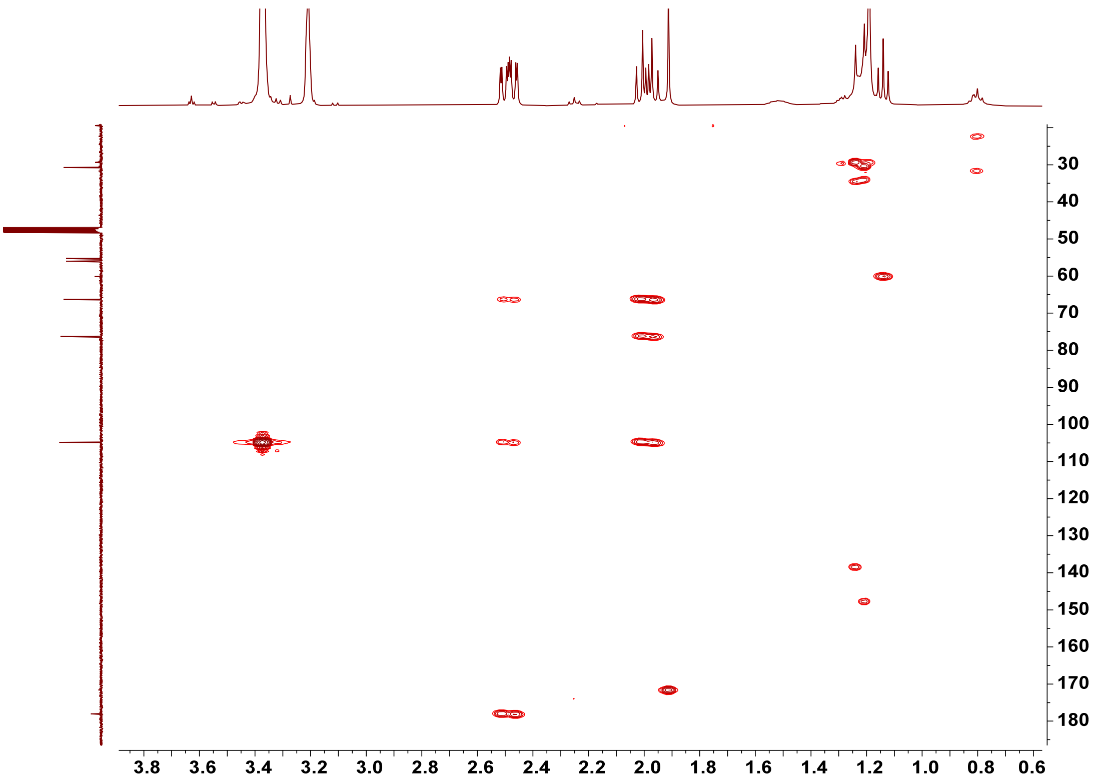

f)

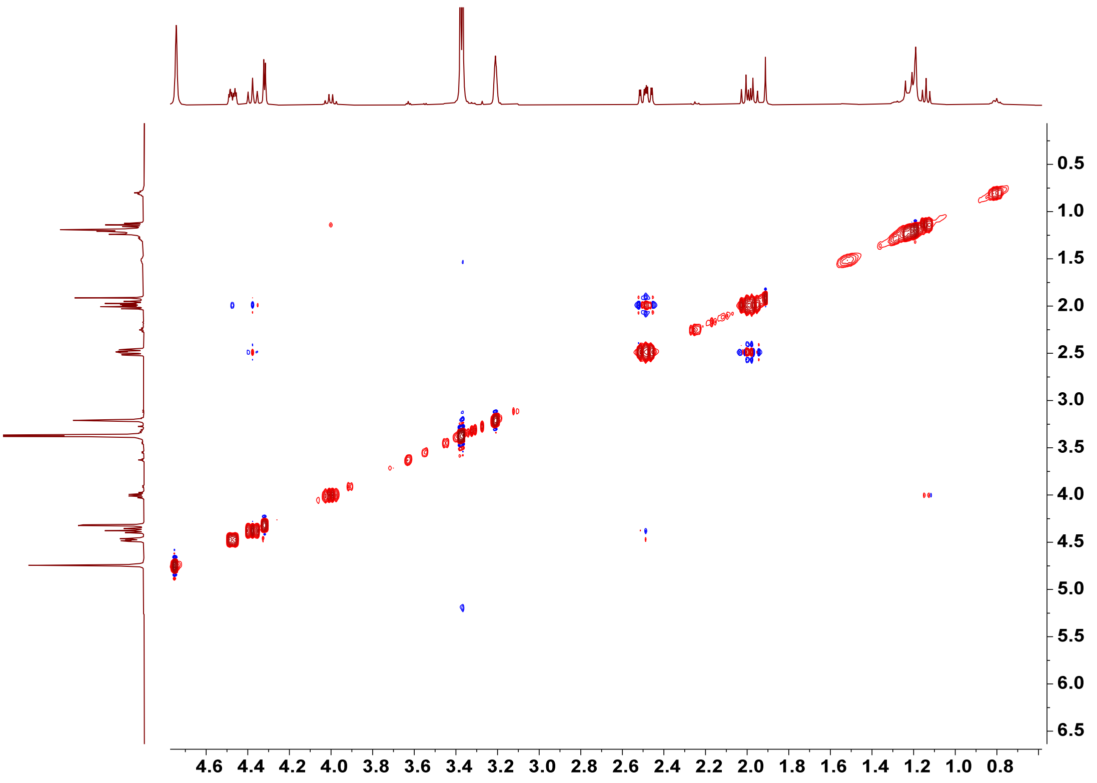

Figure S63. NMR spectra (MeOD) of 2*S*,4*R*-**8c**: a) ^{1}H, b) ^{13}C, c) COSY, d) HSQC, e) HMBC and f) NOESY.

a)

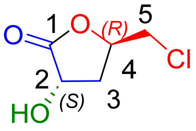

O
1
O
(R)
5
Cl
2
4
HO
(S)
3

2*S*,4*R*-**8d**

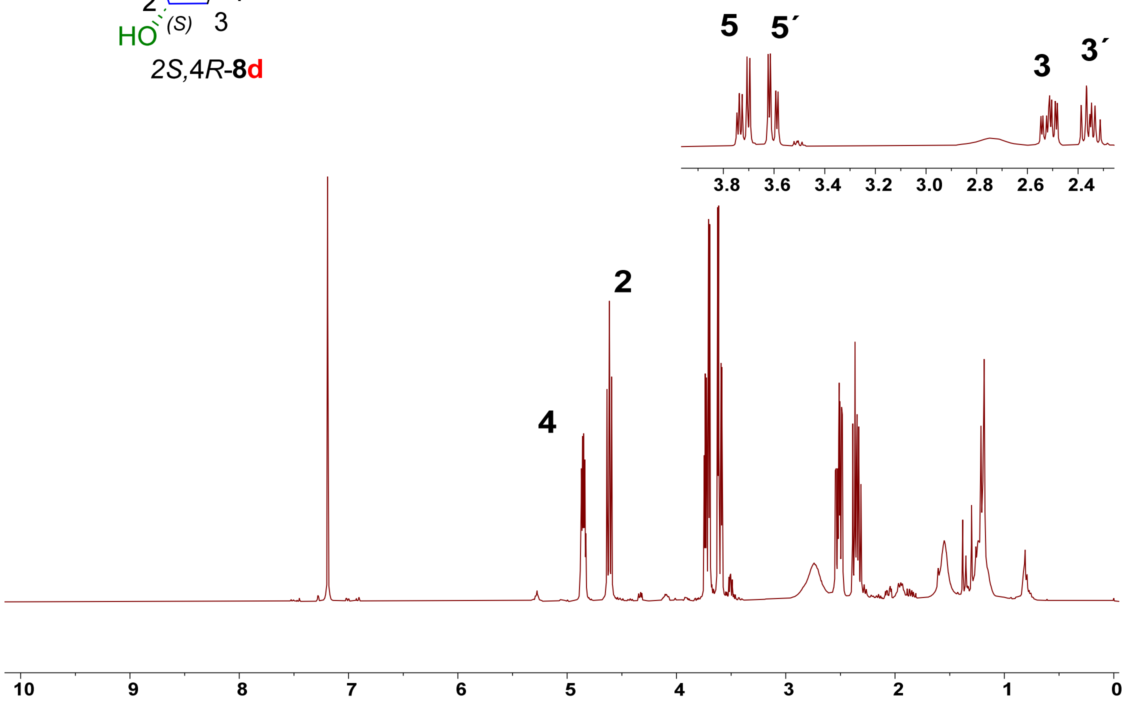

5
5´
3
3´
3.8
3.6
3.4
3.2
3.0
2.8
2.6
2.4
2
4
10
9
8
7
6
5
4
3
2
1
0

b)

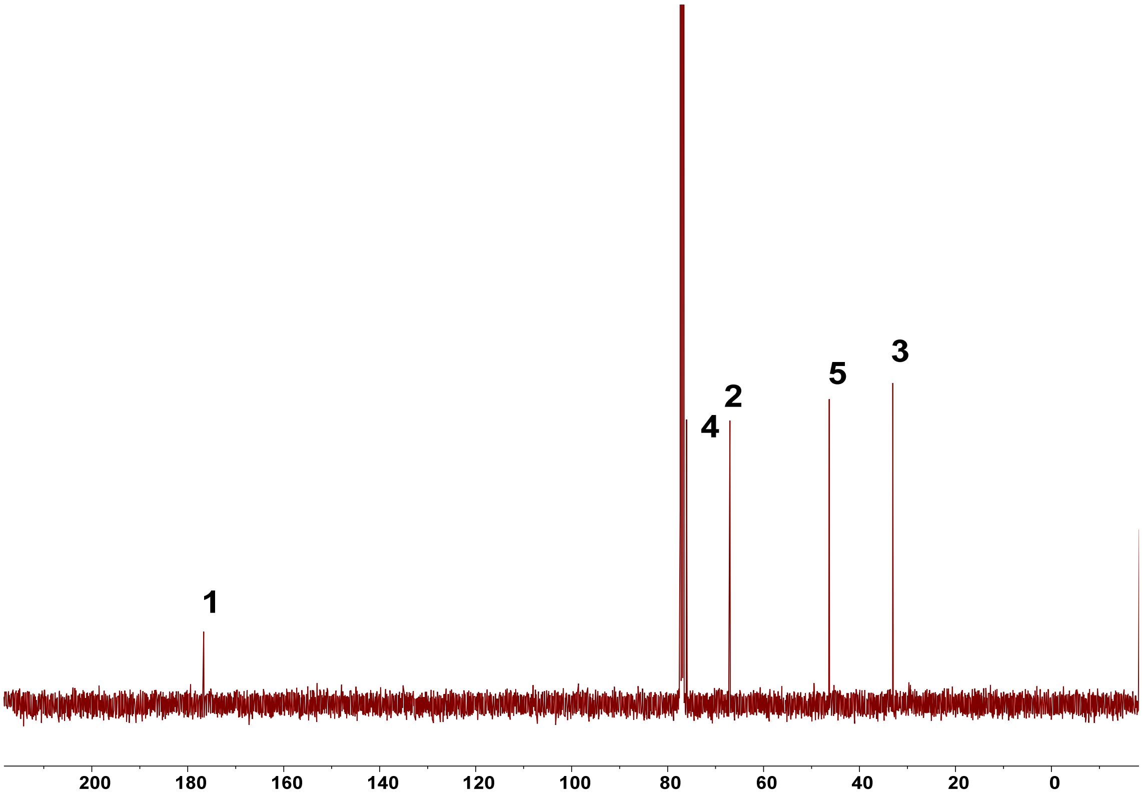

3
5
2
4
1
200
180
160
140
120
100
80
60
40
20
0

c)

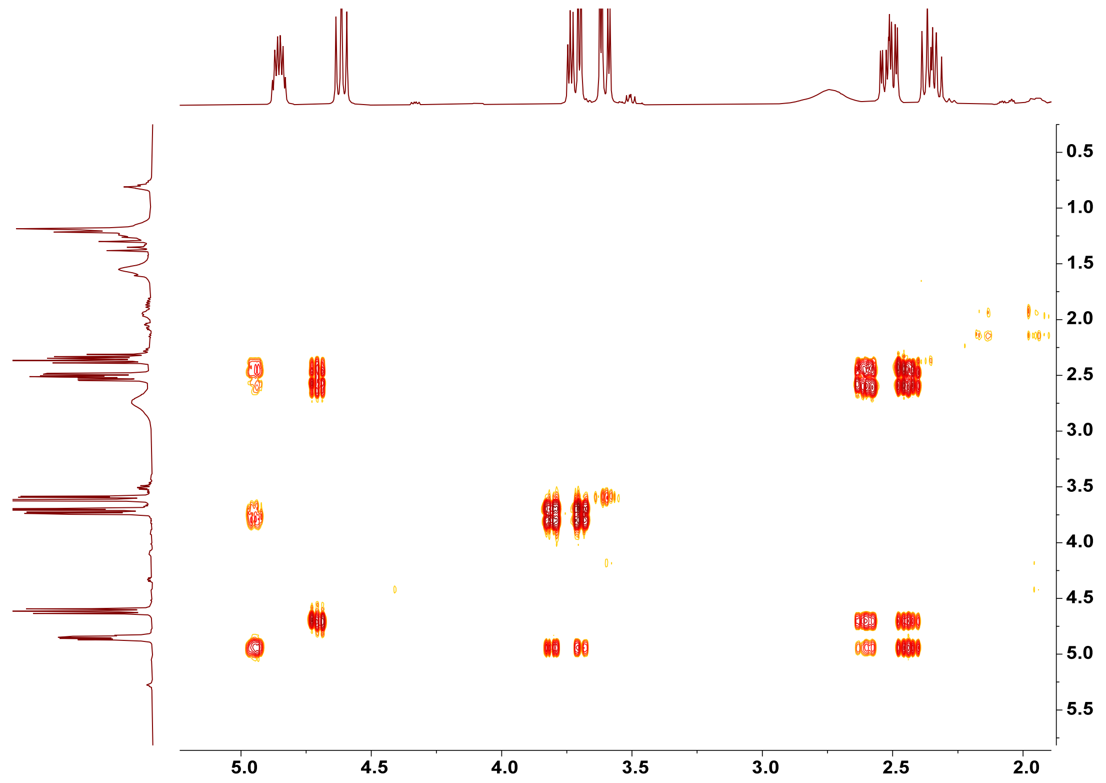
0.5
1.0
1.5
2.0
2.5
3.0
3.5
4.0
4.5
5.0
5.5
5.0 4.5 4.0 3.5 3.0 2.5 2.0

d)

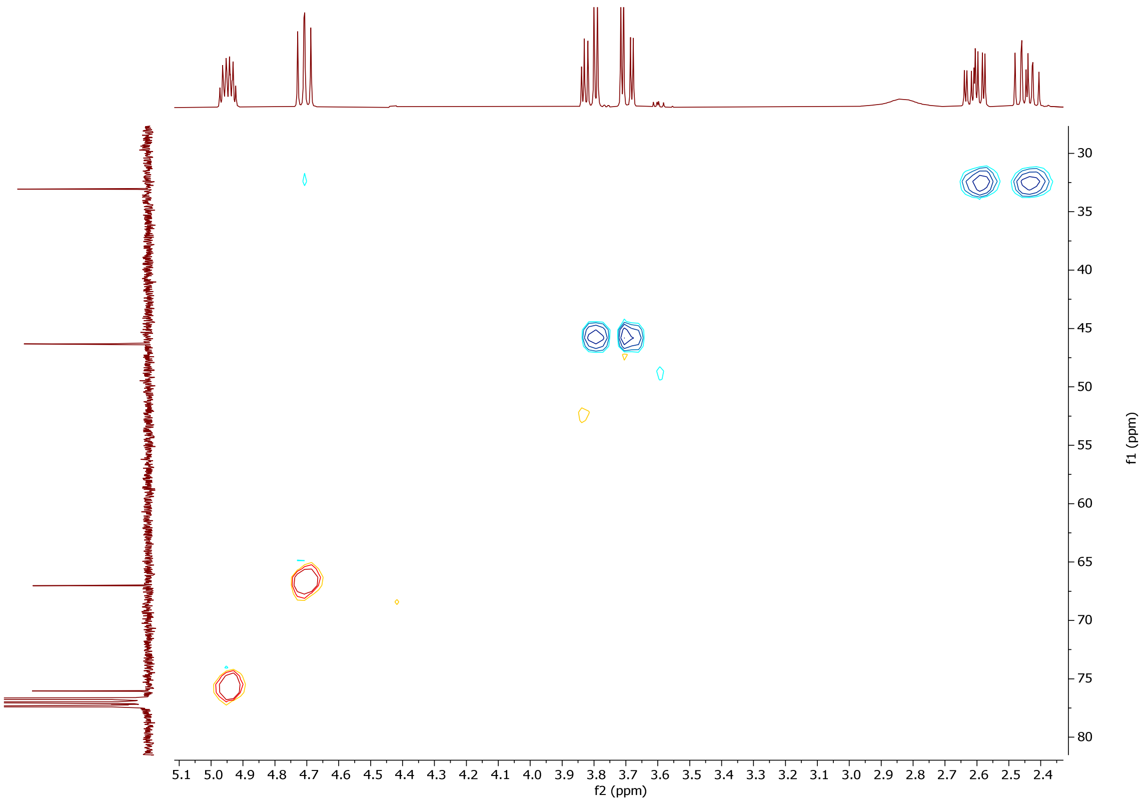
30
35
40
45
50
55
60
65
70
75
80
f1 (ppm)
5.1 5.0 4.9 4.8 4.7 4.6 4.5 4.4 4.3 4.2 4.1 4.0 3.9 3.8 3.7 3.6 3.5 3.4 3.3 3.2 3.1 3.0 2.9 2.8 2.7 2.6 2.5 2.4
f2 (ppm)

e)

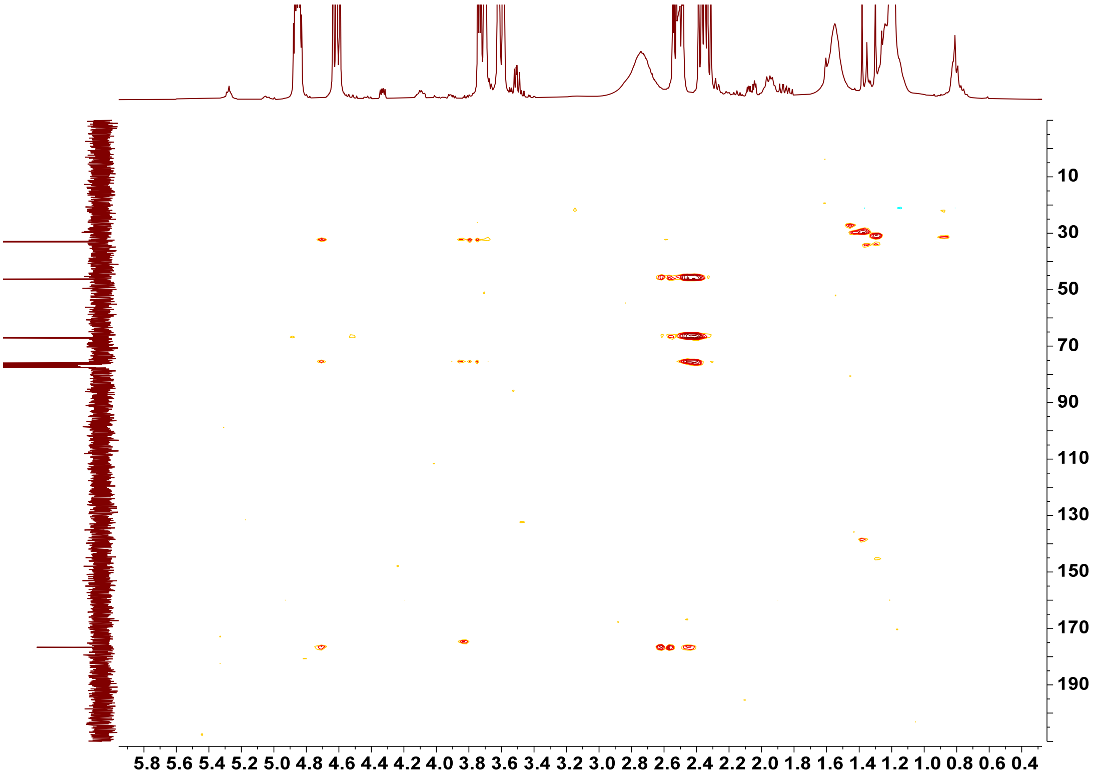

f)

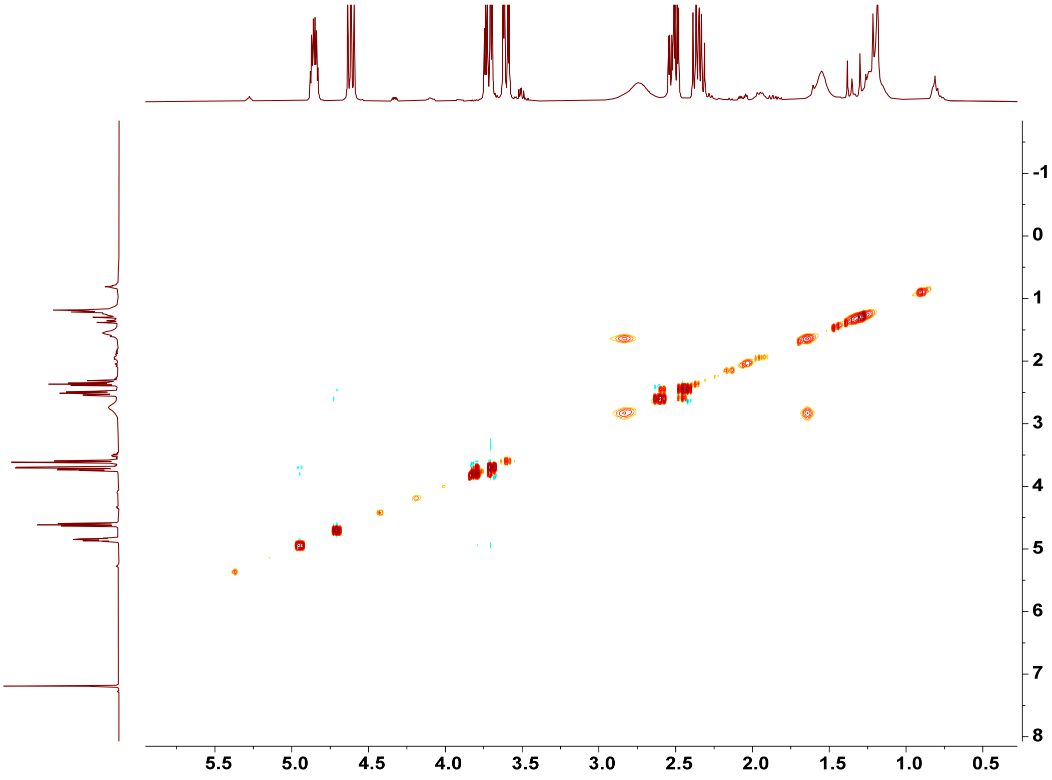

Figure S64. NMR spectra ($CDCl_3$) of 2*S*,4*R*-**8d**: a) ^{1}H, b) ^{13}C, c) COSY, d) HSQC, e) HMBC and f) NOESY.

a)

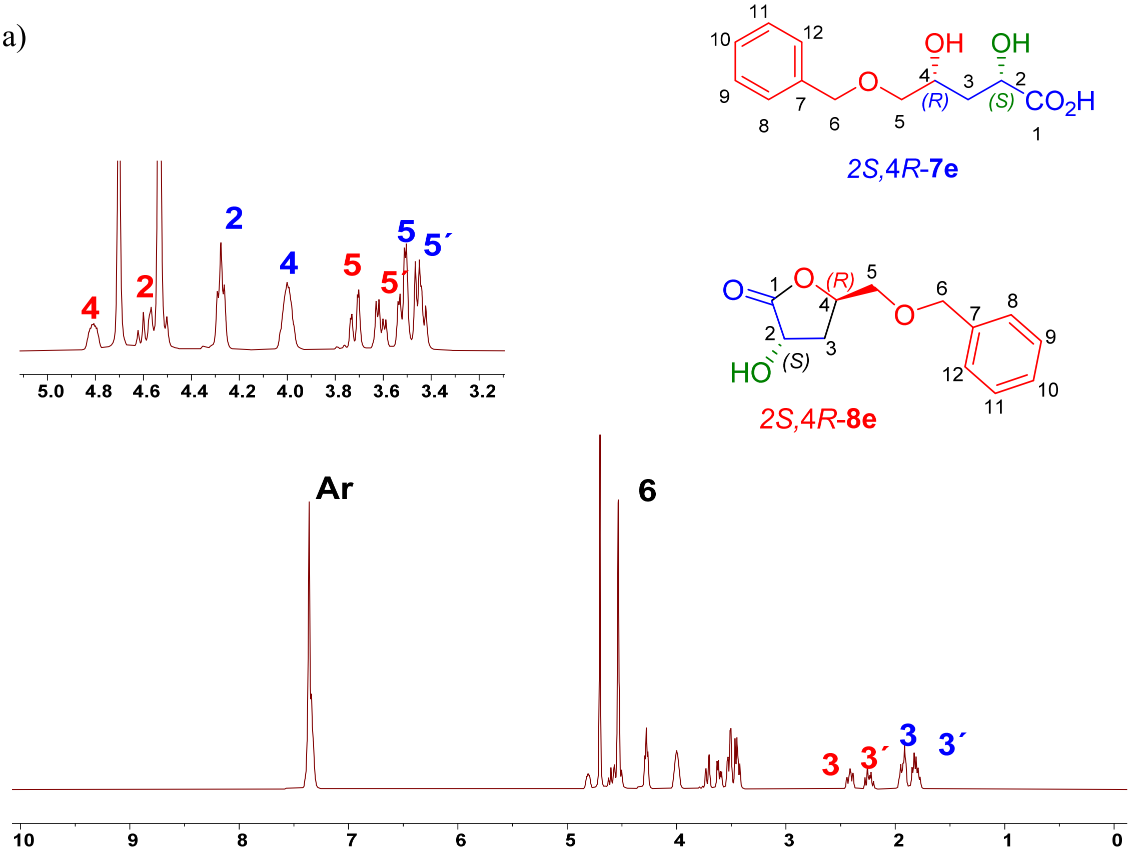

OH
OH
CO2H
(R)
(S)
2S,4R-7e
O
(R)
(S)
HO
2S,4R-8e
Ar
6
4
2
4
5
5
5
5´
3
3´
3
3´

b)

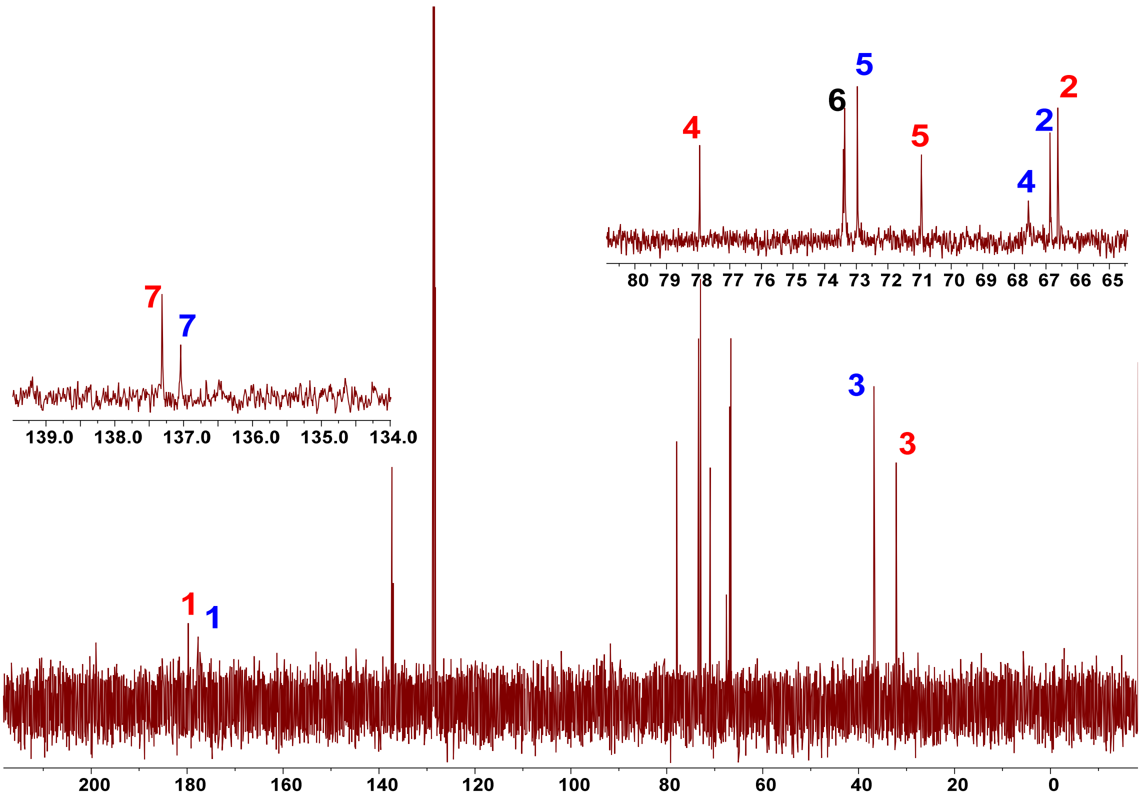

4
6
5
5
2
2
4
7
7
3
3
1
1

c)

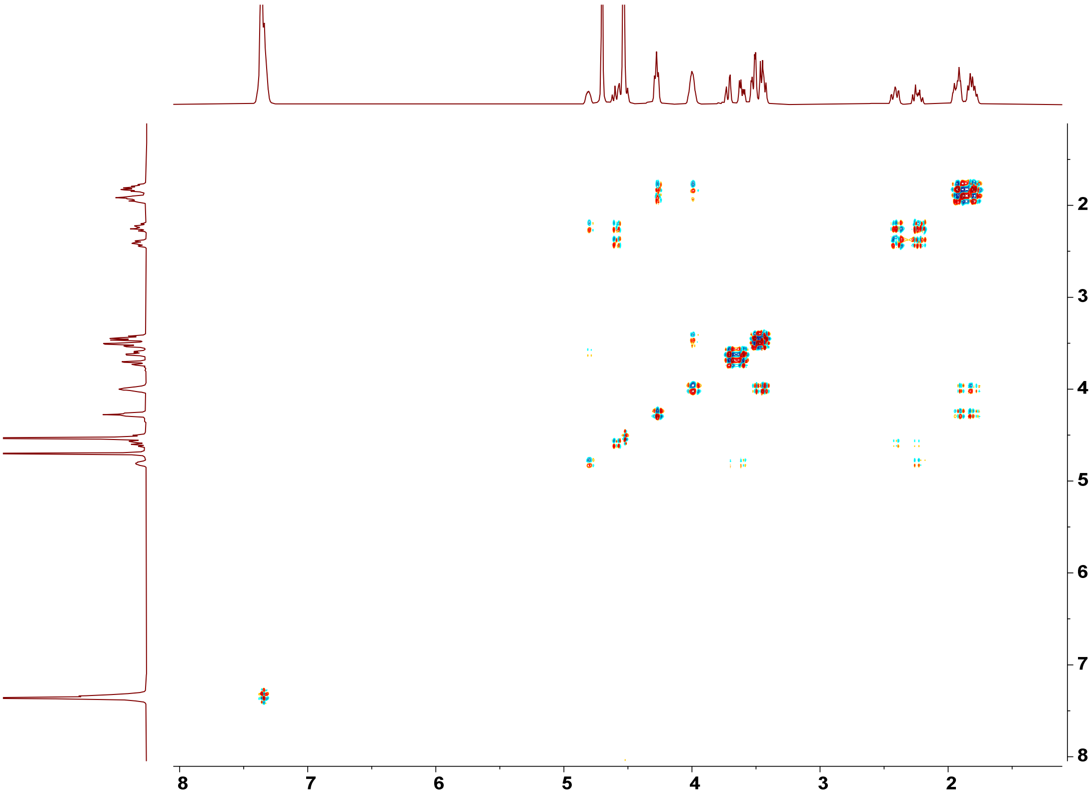
8
7
6
5
4
3
2
2
3
4
5
6
7
8

d)

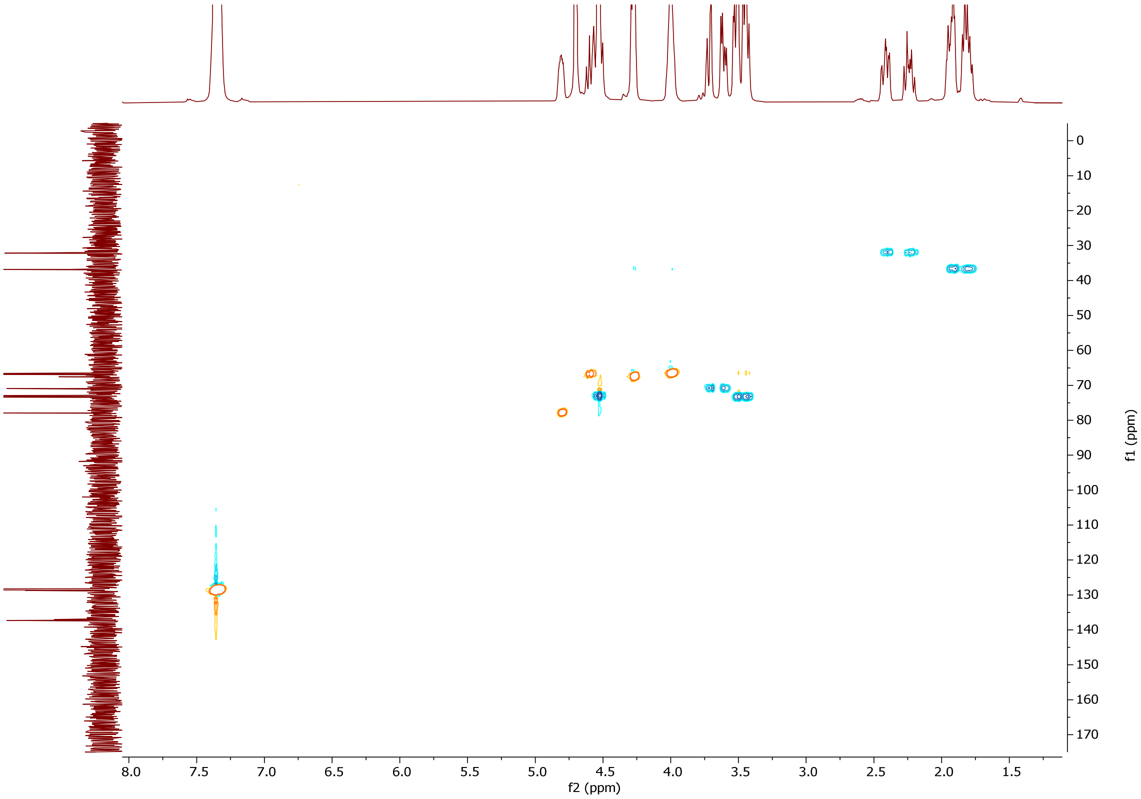
f2 (ppm)
f1 (ppm)
8.0
7.5
7.0
6.5
6.0
5.5
5.0
4.5
4.0
3.5
3.0
2.5
2.0
1.5
0
10
20
30
40
50
60
70
80
90
100
110
120
130
140
150
160
170

e)

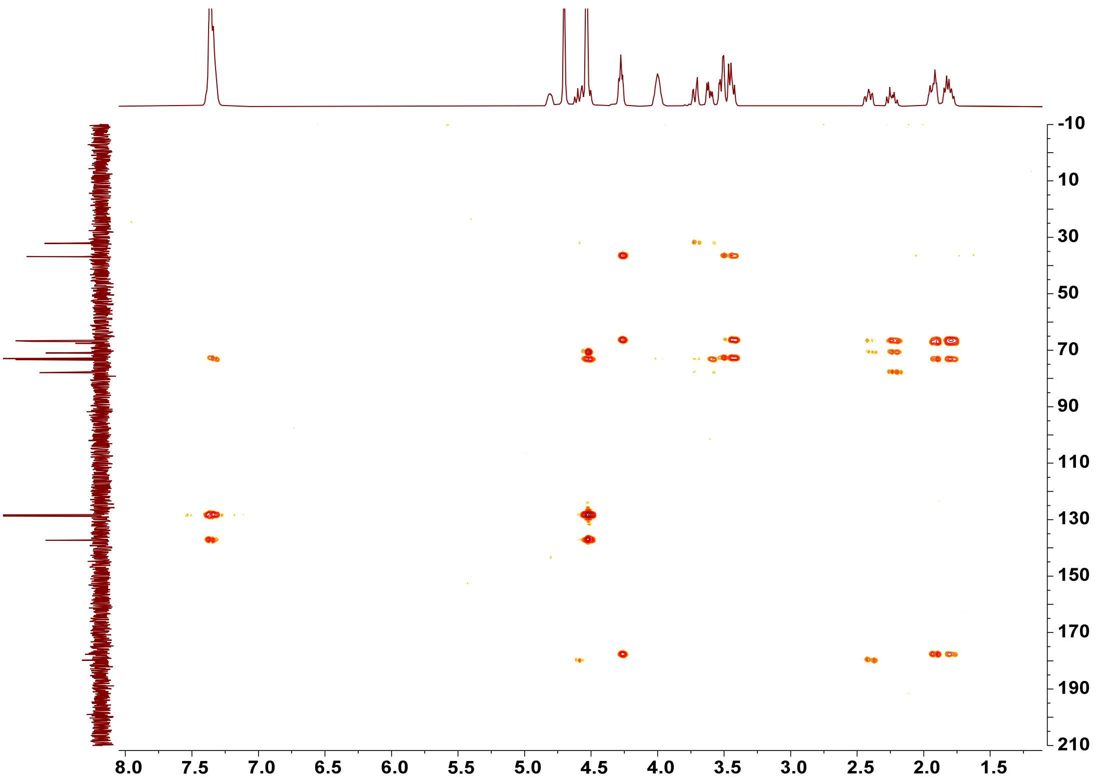

f)

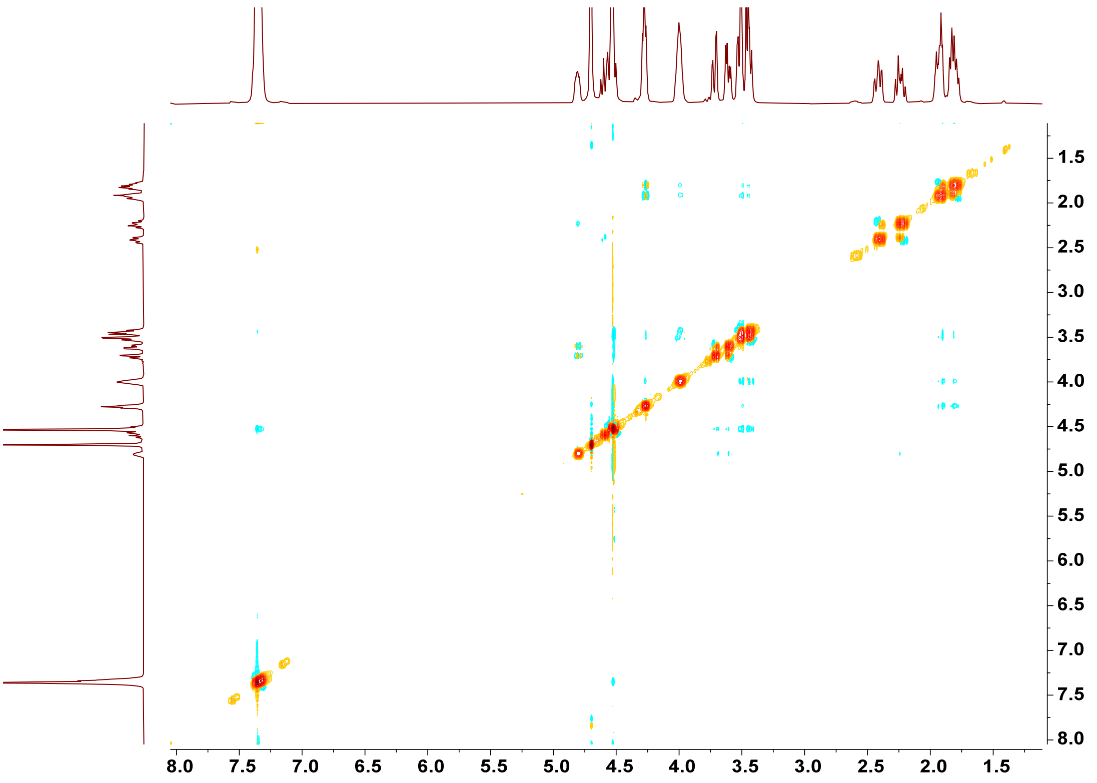

Figure S65. NMR spectra (D_2O) of 2*S*,4*R*-**8e**: a) ^{1}H, b) ^{13}C, c) COSY, d) HSQC, e) HMBC and f) NOESY.

a)

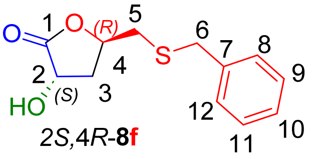

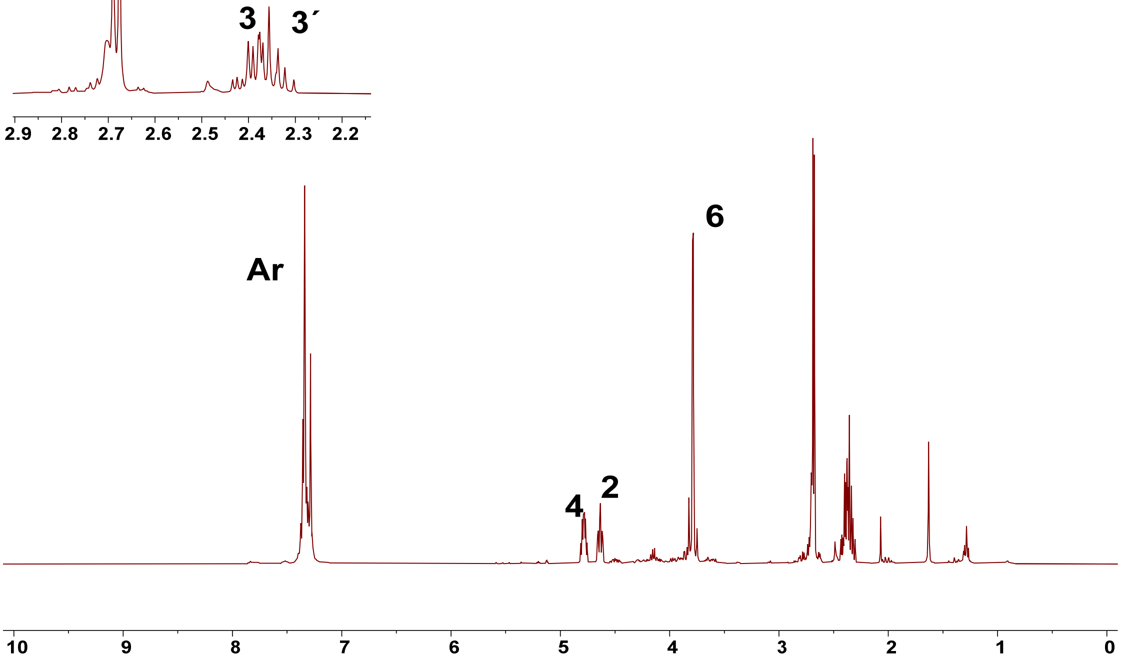

b)

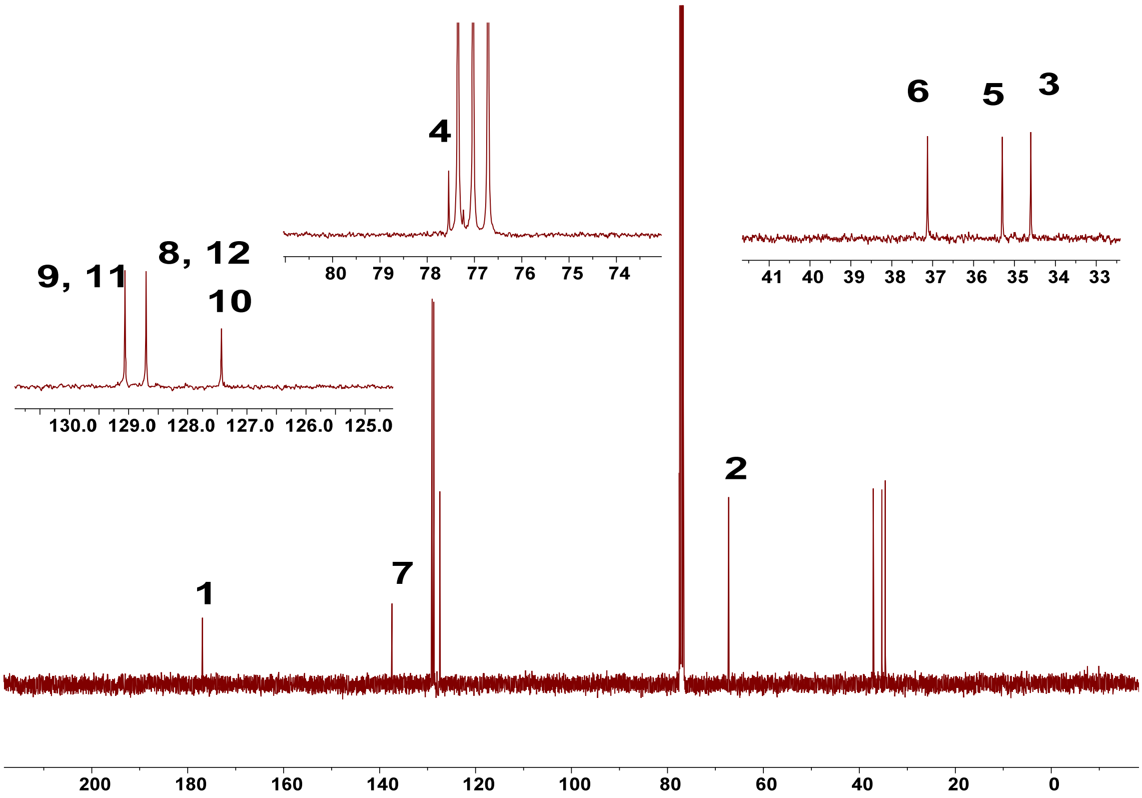

c)

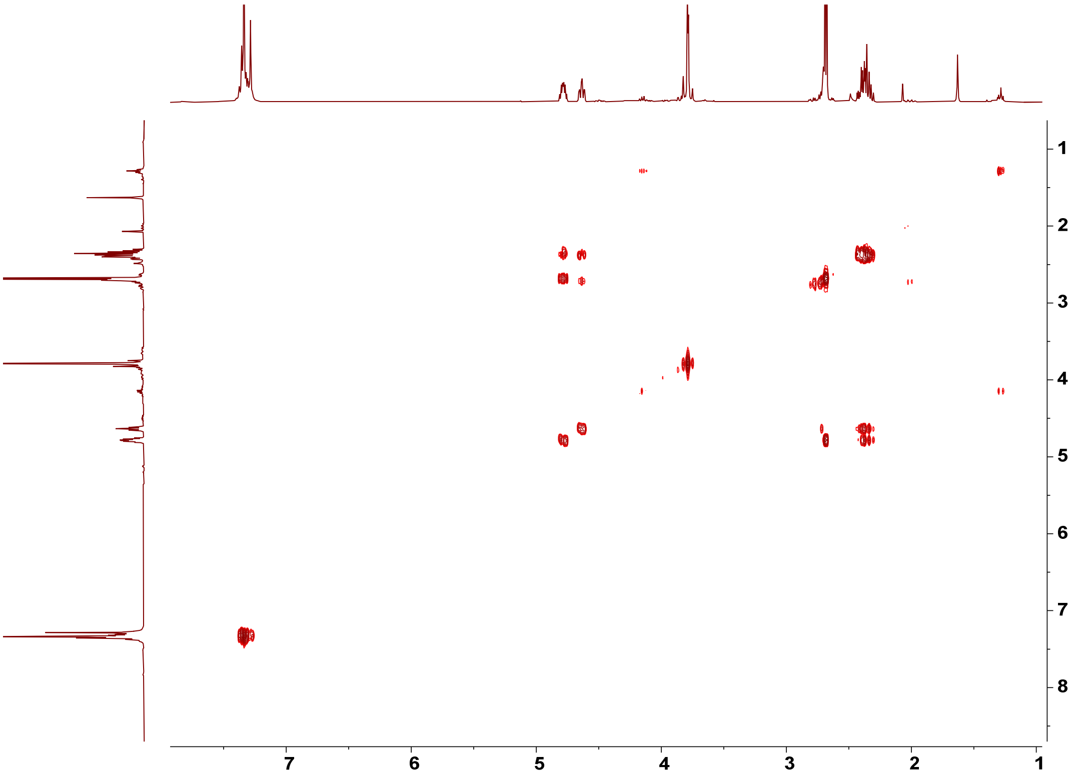
1
2
3
4
5
6
7
8
7
6
5
4
3
2
1

d)

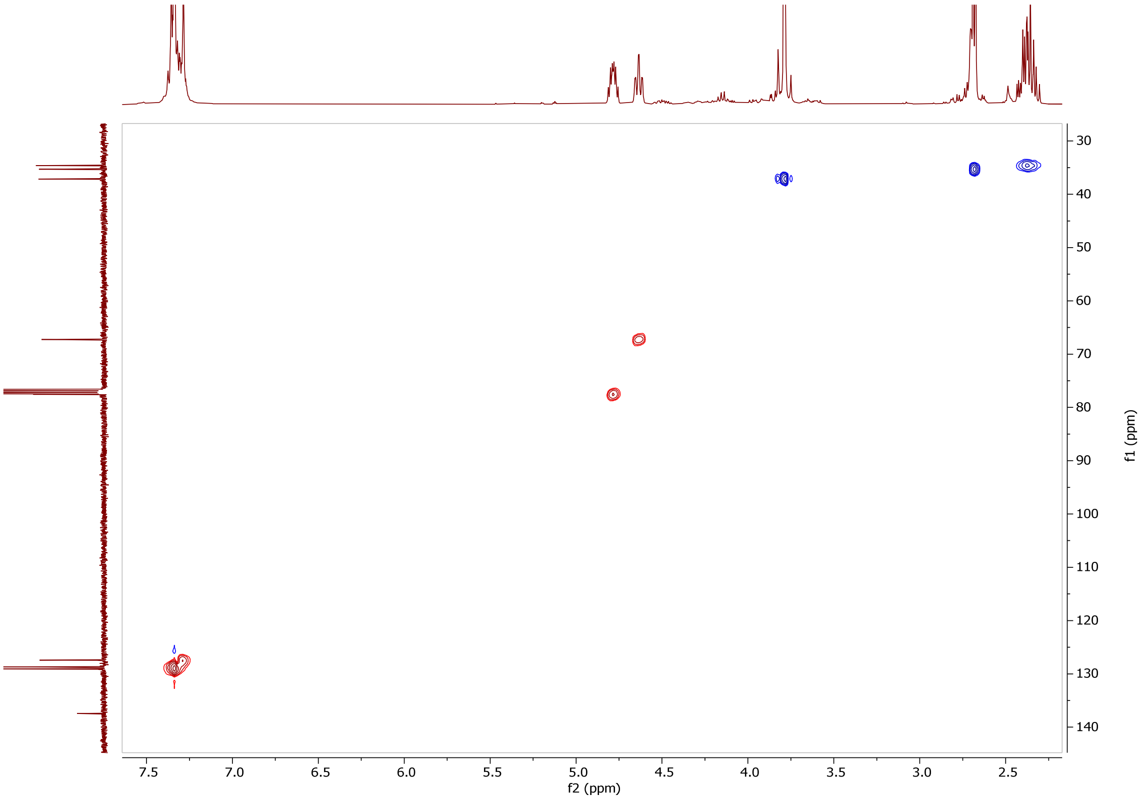
30
40
50
60
70
80
90
100
110
120
130
140
f1 (ppm)
7.5
7.0
6.5
6.0
5.5
5.0
4.5
4.0
3.5
3.0
2.5
f2 (ppm)

e)

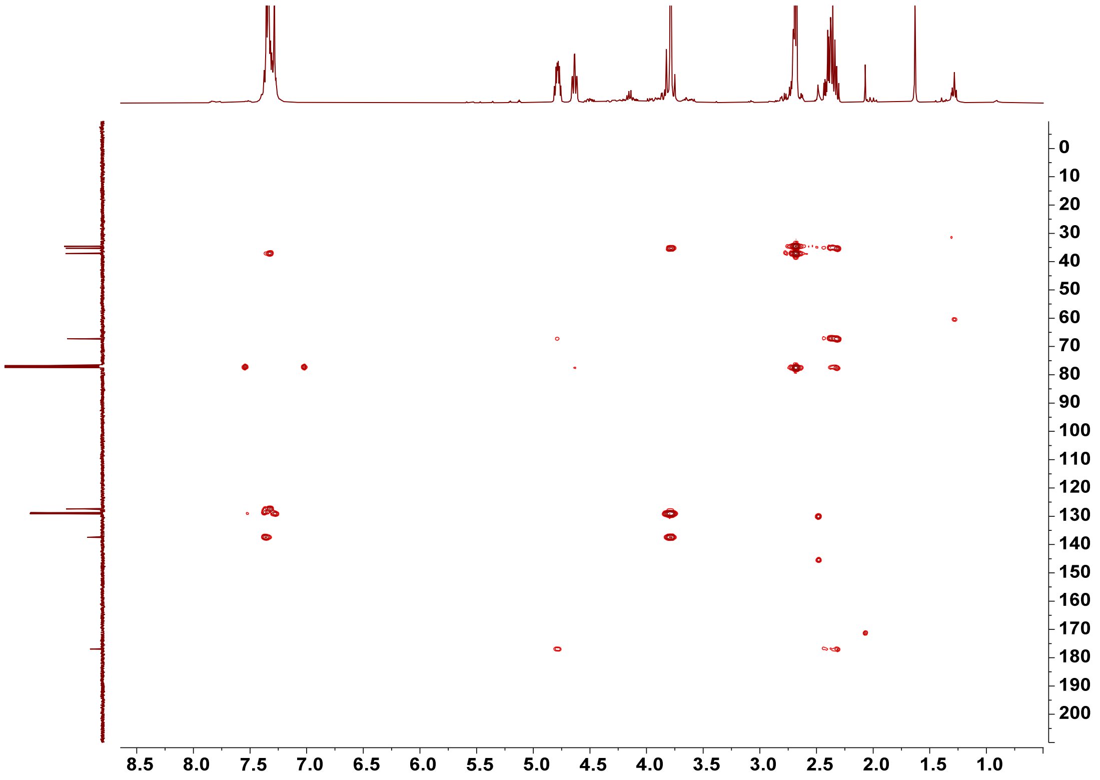

f)

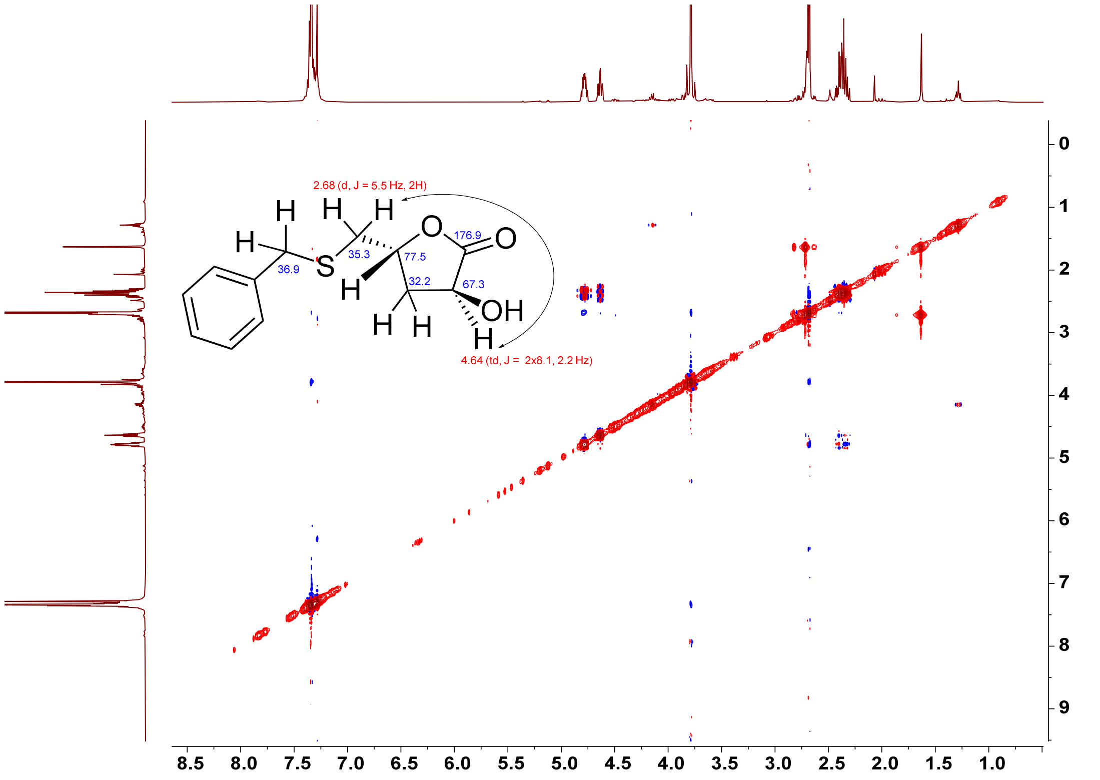

Figure S66. NMR spectra ($CDCl_3$) of 2*S*,4*R*-**8f**: a) ^{1}H, b) ^{13}C, c) COSY, d) HSQC, e) HMBC and f) NOESY.

a)

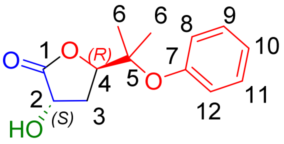

O
1
O
(R)
4
5
O
6
6
7
8
9
10
11
12
2
HO
(S)
3

2S,4R-**8g**

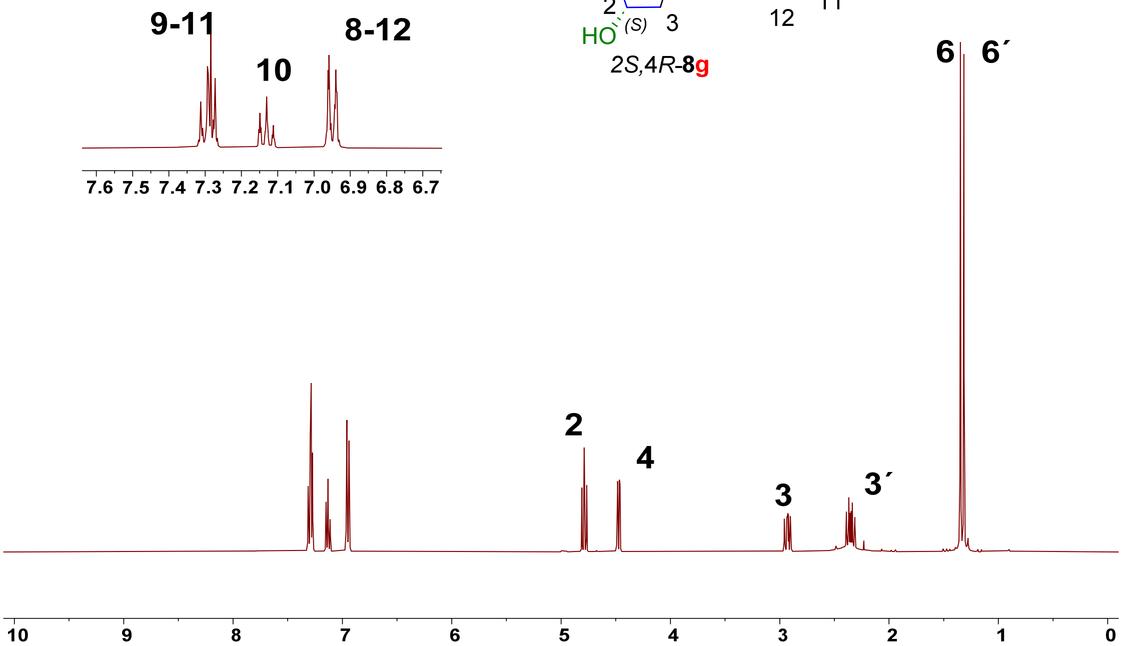

9-11
10
8-12
7.6 7.5 7.4 7.3 7.2 7.1 7.0 6.9 6.8 6.7
6
6´
2
4
3
3´
10
9
8
7
6
5
4
3
2
1
0

b)

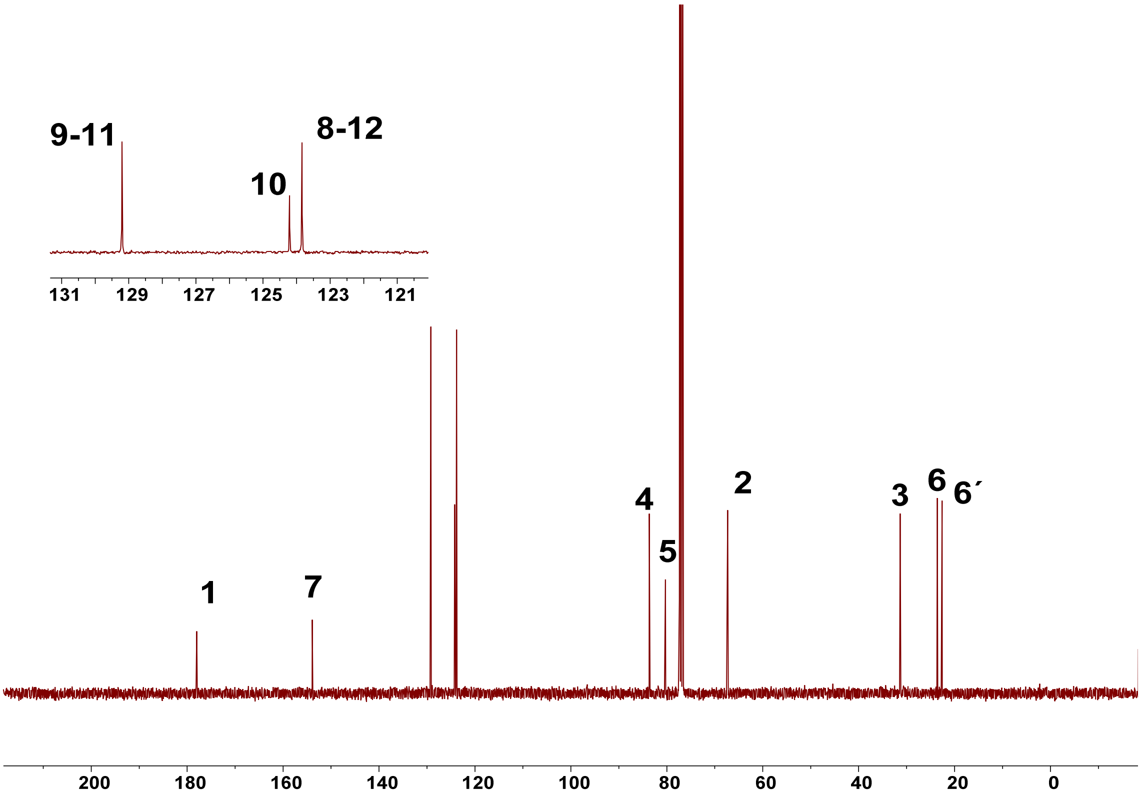

9-11
10
8-12
131
129
127
125
123
121
1
7
4
5
2
3
6
6´
200
180
160
140
120
100
80
60
40
20
0

c)

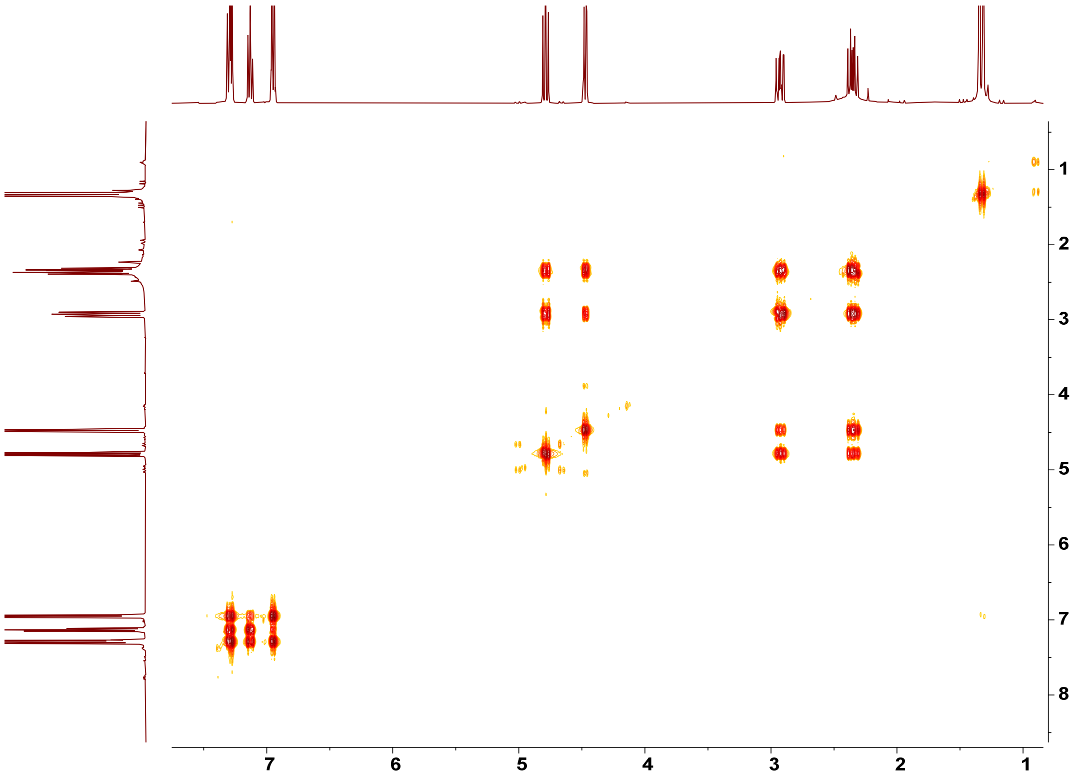
7
6
5
4
3
2
1
8

d)

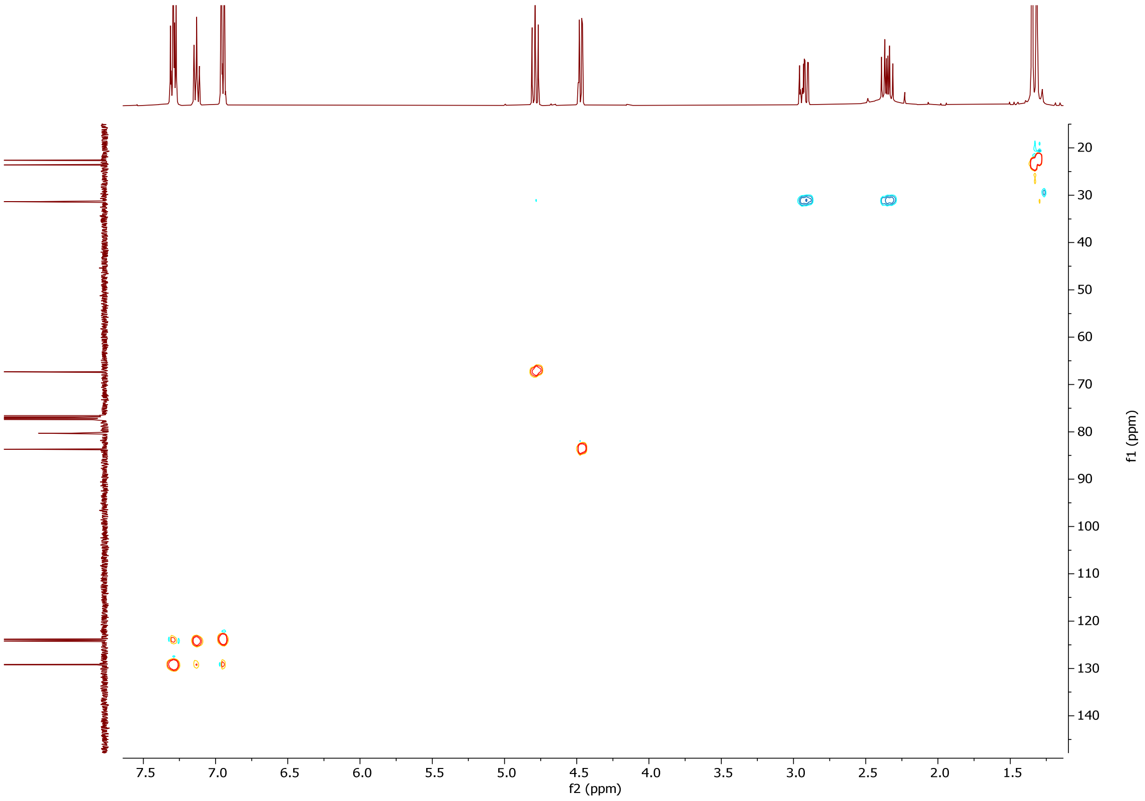
f2 (ppm)
f1 (ppm)
7.5
7.0
6.5
6.0
5.5
5.0
4.5
4.0
3.5
3.0
2.5
2.0
1.5
20
30
40
50
60
70
80
90
100
110
120
130
140

e)

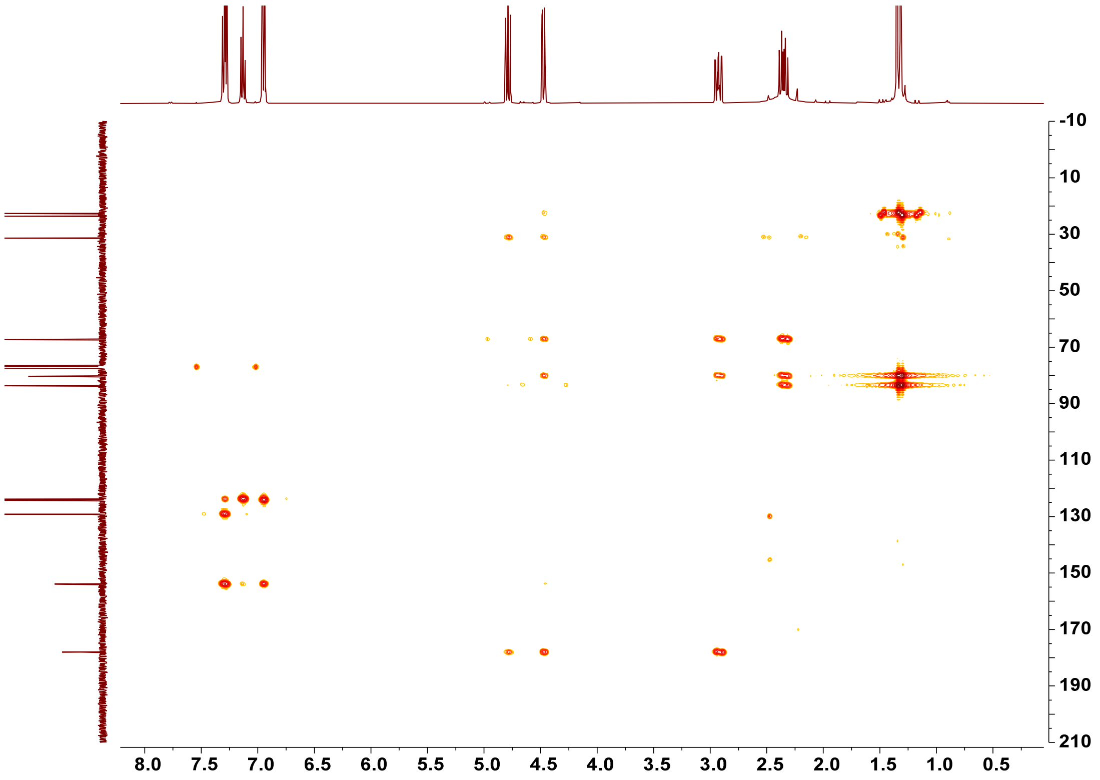

f)

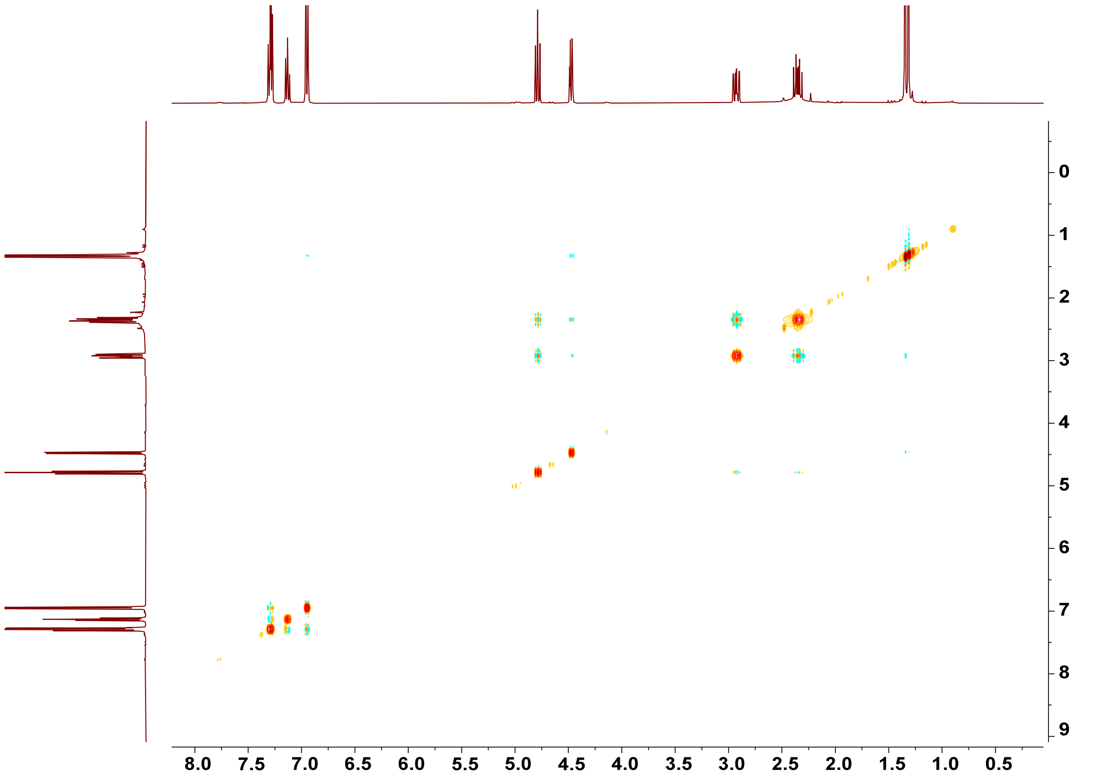

Figure S67. NMR spectra ($CDCl_3$) of 2*S*,4*R*-**8g**: a) ^{1}H, b) ^{13}C, c) COSY, d) HSQC, e) HMBC and f) NOESY.

Computational Methods.

Protein complexes were modeled with the package Schrödinger Suite 2022-2,[18] through its graphical interface Maestro.[19] The Protein Preparation Wizard[20] included in Maestro was used to prepare the protein structure by removing solvent molecules and ions, adding hydrogens, setting protonation states[21] and running a restrained minimization using the OPLS4 force-field.[22] The program MacroModel[23] with the same force field and GB/SA water solvation conditions[24] was used for further molecular mechanics calculations. The program QSite[19, 25-26] was used for the QM/MM calculations.

Molecular models of the pre-reactive 4-hydroxy-2-oxoacids bound into the active sites of KPR_{Ecoli} and $DpkA_{Psyrin}$ were generated from the reported crystal structures of both enzymes.[27-28] In particular, KPR_{Ecoli} was modeled from chain B of PDB structure 2OFP,[27] which is in the closed form and includes the NADP cofactor and a molecule of pantoate. On the other hand, all the modeling with $DpkA_{Psyrin}$ was performed on the active site of chain A of PDB structure 2CWH,[28] although both chains A and B, which show quite similar overall structures, were taken into account for the calculations since the enzyme naturally functions as a dimer and the active site cavity on each subunit also involves residues from the neighboring one. Substrates **3** were built within Maestro, based on the structure of the ligands bound in each case. The structures of the complexes were minimized with QSite at the DFT B3LYP/6-31G** level of theory. For KPR_{Ecoli} the QM/MM boundary was defined by placement of hydrogen caps between the Cα and Cβ atoms of residues Lys176, Asn184, Asn194 and Asn241, as well as between the C4' and C5' atoms of the ribose ring bound to the nicotinamide moiety of the NADPH cofactor (Figure S68A). For $DpkA_{Psyrin}$ the boundary was established through hydrogen caps between the Cα and Cβ atoms of residues His54, Arg58 and Thr166, and the C4' and C5' atoms of the same ribose ring of NADPH (Figure S68B). In both cases the substrate molecule was also part of the QM region. All residues with atoms within 6 Å of the substrate and the NADPH molecules were simultaneously optimized using the default OPLS2005 force-field,[29] while residues which were further away were kept frozen. Furthermore, to find the best bound conformations for the more flexible substrates, a conformational search was performed using the mixed MCMM/LMCS method[30] implemented in MacroModel to find the best poses for the C-3 substituents (R^1 and R^2 substituents in Scheme 2 of the main text) , while the rest of the system was kept frozen. Then, the best conformers detected by this search were

QM/MM reoptimized as above. All complexes were characterized as minima by running frequency analysis calculations and confirming that they had no imaginary frequencies.

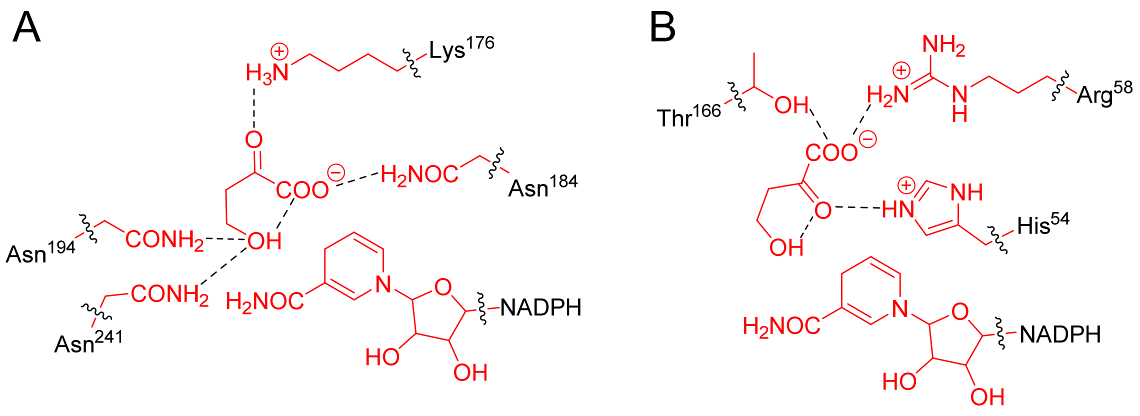

Figure S68. Active sites of KPR$_{Ecoli}$ (A) and DpkA$_{Psyrin}$ (B) with bound **3a**, as example substrate, and atoms that constitute the QM region (red atoms) in each case. Wavy lines denote where the hydrogen caps were placed; dashed lines represent hydrogen bonds.

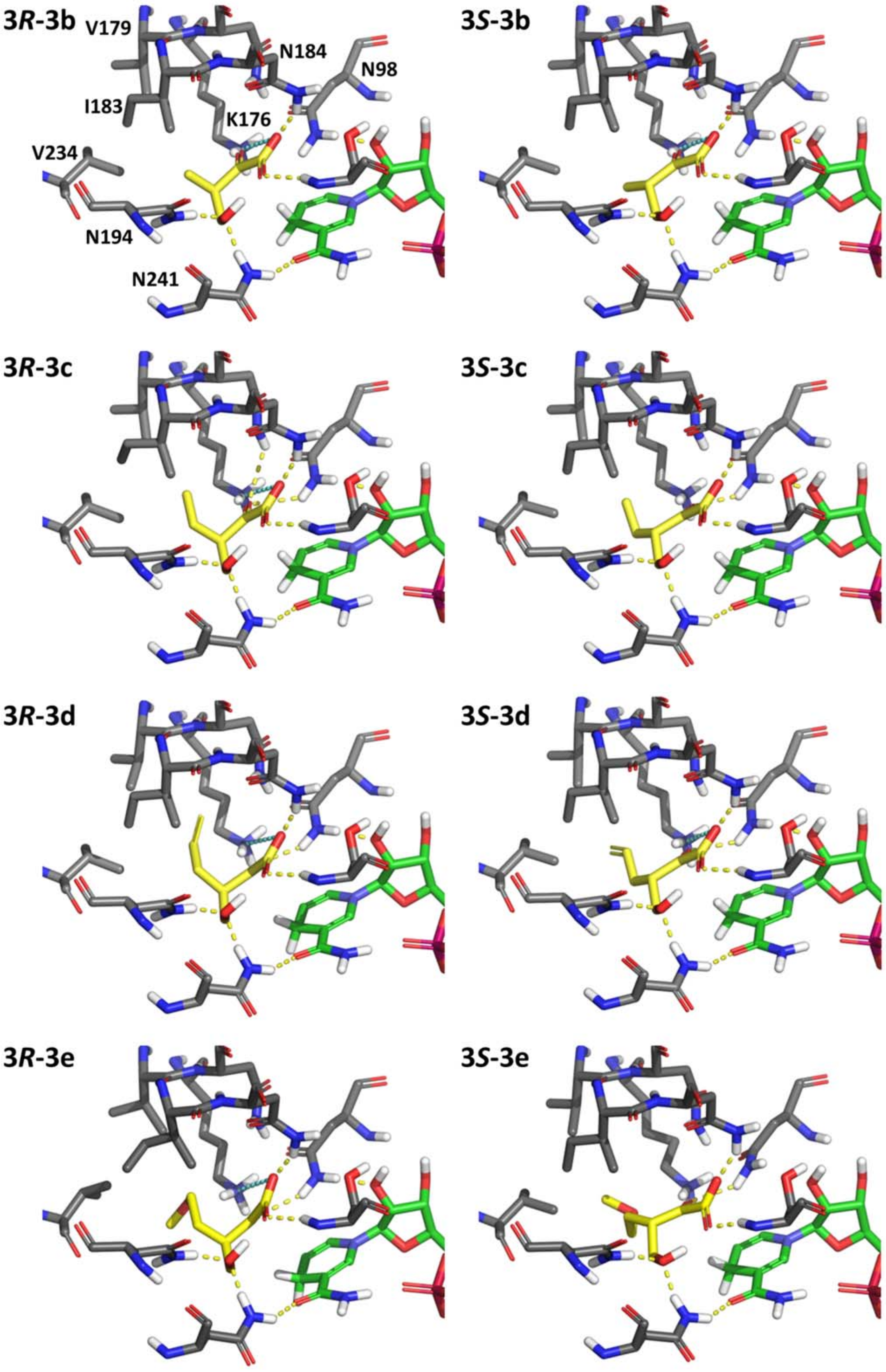
3R-3b
V179
N184
N98
I183
K176
V234
N194
N241
3S-3b
3R-3c
3S-3c
3R-3d
3S-3d
3R-3e
3S-3e

3*R*-3f

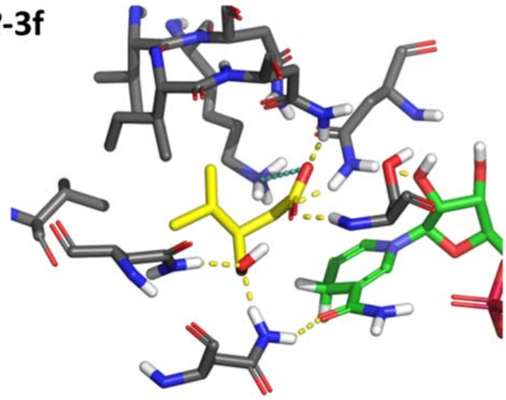

3*S*-3f

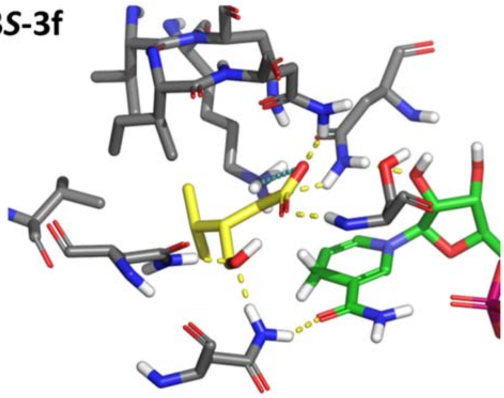

3*R*-3g

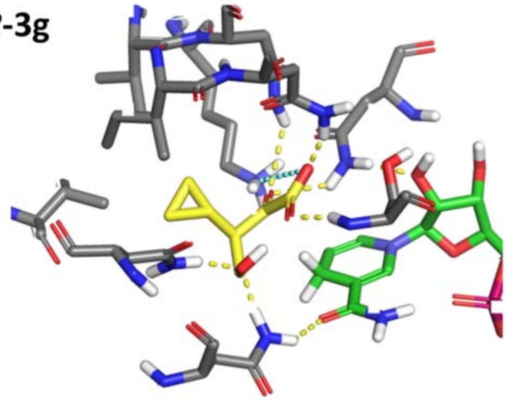

3*S*-3g

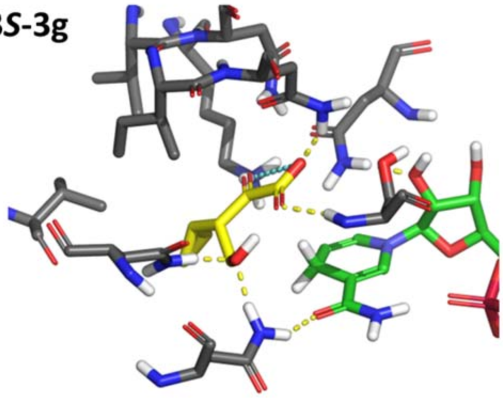

3*R*-3h

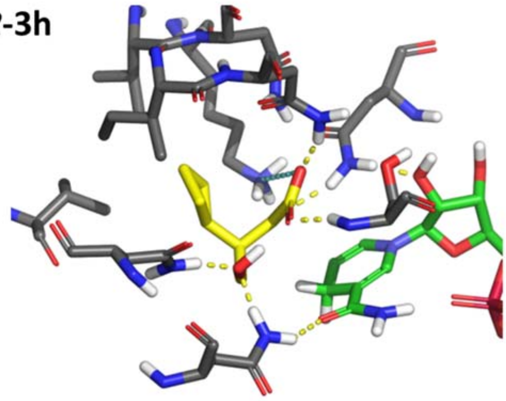

3*S*-3h

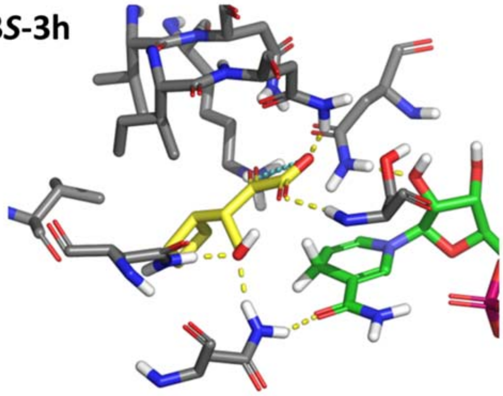

3j

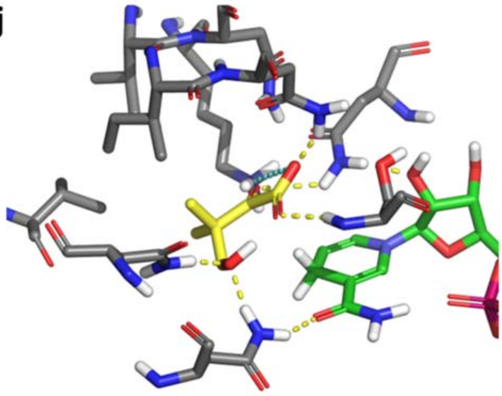

3k

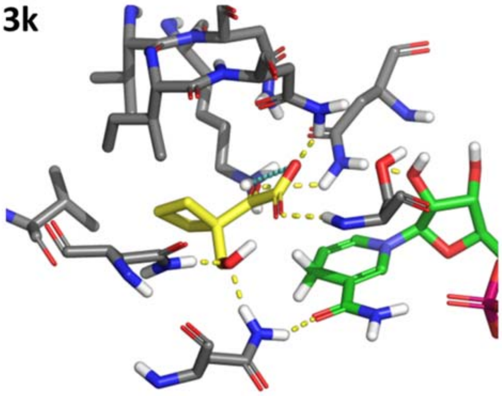

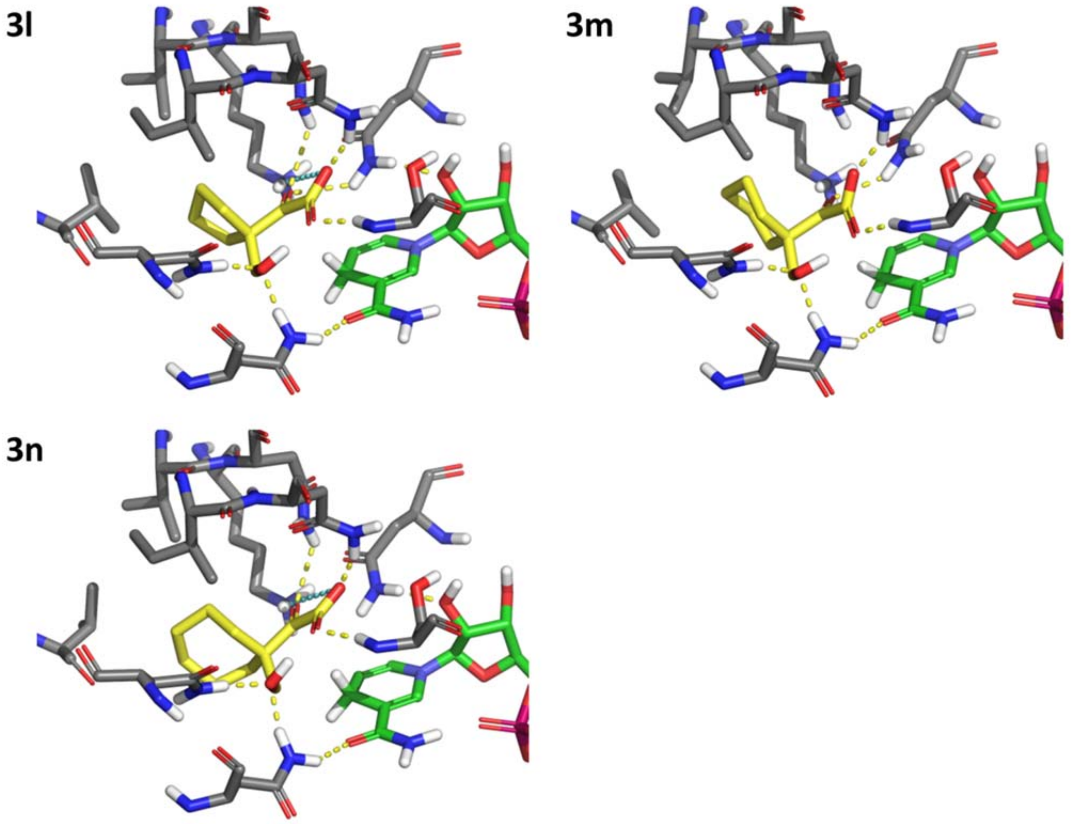

Figure S69. Molecular models of substrates **3** bound into the active site of KPR_{Ecoli}. These models were built starting from PDB structure 2OFP and they were optimized by QM/MM methods as described. The substrate, NADPH and close protein residues are shown with yellow, green and gray C-atoms; H-bonds and salt bridges are shown with yellow and cyan dashed lines.

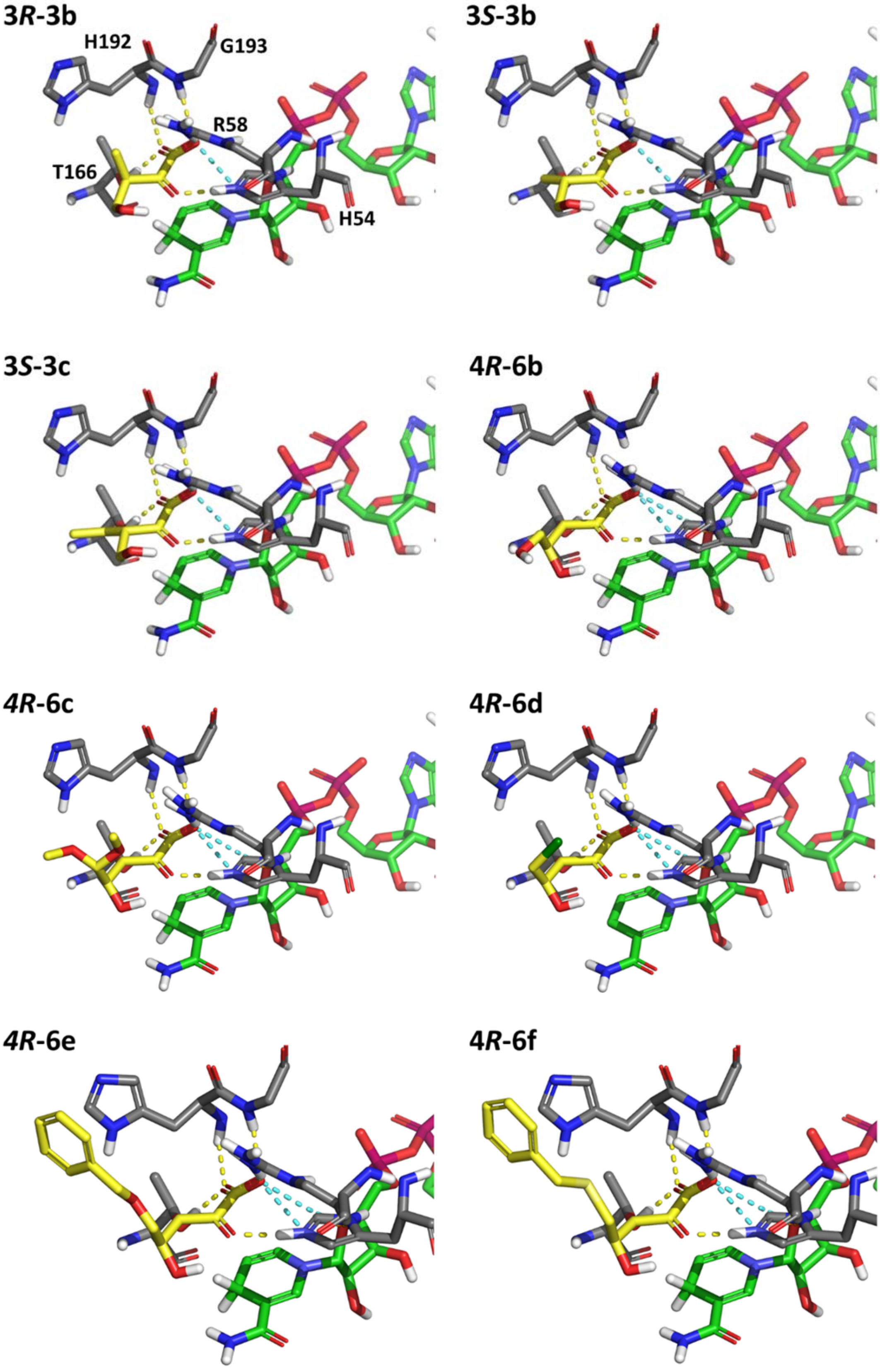
3R-3b
H192
G193
R58
T166
H54
3S-3b
3S-3c
4R-6b
4R-6c
4R-6d
4R-6e
4R-6f

4R-6g

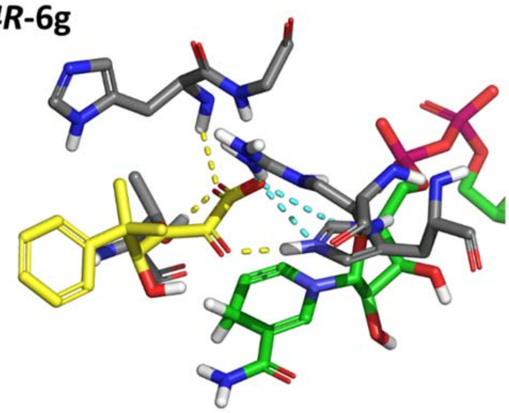

Figure S70. Molecular models of substrates **3** and **6** bound into the active site of DpkA$_{Psyrin}$. These models were built starting from PDB structure 2CWH and they were optimized by QM/MM methods as described. The substrate, NADPH and close protein residues are shown with yellow, green and gray C-atoms; H-bonds and salt bridges are shown with yellow and cyan dashed lines.

References

1. Marín-Valls, R.; Hernandez, K.; Bolte, M.; Joglar, J. s.; Bujons, J.; Clapes, P., Chemoenzymatic hydroxymethylation of carboxylic acids by tandem stereodivergent biocatalytic aldol reaction and chemical decarboxylation. *ACS Catalysis* **2019,** *9* (8), 7568-7577.
2. Marín-Valls, R.; Hernández, K.; Bolte, M.; Parella, T.; Joglar, J. s.; Bujons, J.; Clapés, P., Biocatalytic construction of quaternary centers by aldol addition of 3, 3-disubstituted 2-oxoacid derivatives to aldehydes. *Journal of the American Chemical Society* **2020,** *142* (46), 19754-19762.
3. Moreno, C. J.; Hernández, K.; Charnok, S. J.; Gittings, S.; Bolte, M.; Joglar, J.; Bujons, J.; Parella, T.; Clapés, P., Synthesis of γ-hydroxy-α-amino acid derivatives by enzymatic tandem aldol addition–transamination reactions. *ACS catalysis* **2021,** *11* (8), 4660-4669.
4. QIAexpressionist, A., A handbook for high-level expression and purification of 6xhis-tagged proteins. *Qiagen. p1-125* **2002**.
5. Hernandez, K.; Bujons, J.; Joglar, J. s.; Charnock, S. J.; Dominguez de Maria, P.; Fessner, W. D.; Clapés, P., Combining aldolases and transaminases for the synthesis of 2-amino-4-hydroxybutanoic acid. *ACS Catalysis* **2017,** *7* (3), 1707-1711.
6. Hernández, K.; Gómez, A.; Joglar, J.; Bujons, J.; Parella, T.; Clapés, P., 2-Keto-3-Deoxy-L-Rhamnonate Aldolase (YfaU) as Catalyst in Aldol Additions of Pyruvate to Amino Aldehyde Derivatives. *Adv. Synth. Catal.* **2017,** *359* (12), 2090-2100.
7. Hernández, K.; Joglar, J.; Bujons, J.; Parella, T.; Clapés, P., Nucleophile promiscuity of engineered class II pyruvate aldolase YfaU from E. coli. *Angewandte Chemie International Edition* **2018,** *57* (14), 3583-3587.
8. Green, M. R.; Sambrook, J., The Hanahan method for preparation and transformation of competent Escherichia coli: high-efficiency transformation. *Cold Spring Harbor Protocols* **2018,** *2018* (3), pdb. prot101188.
9. Marín-Valls, R.; Hernández, K.; Bolte, M.; Joglar, J.; Bujons, J.; Clapés, P., Chemoenzymatic Hydroxymethylation of Carboxylic Acids by Tandem Stereodivergent

Biocatalytic Aldol Reaction and Chemical Decarboxylation. *ACS Catal.* **2019,** *9* (8), 7568-7577.
10. Marín-Valls, R.; Hernández, K.; Bolte, M.; Parella, T.; Joglar, J.; Bujons, J.; Clapés, P., Biocatalytic Construction of Quaternary Centers by Aldol Addition of 3,3-Disubstituted 2-Oxoacid Derivatives to Aldehydes. *J. Am. Chem. Soc.* **2020,** *142* (46), 19754-19762.
11. Yamaguchi, S.; Motokura, K.; Sakamoto, Y.; Miyaji, A.; Baba, T., Tin-catalyzed conversion of biomass-derived triose sugar and formaldehyde to α-hydroxy-γ-butyrolactone. *Chem. Commun.* **2014,** *50* (35), 4600-4602.
12. Magnani, J. L.; Peterson, J. M.; Zierke, M.; Smiesko, M.; Ernst, B., E-Selectin Antagonists Modified by Macrocycle Formation to the Galactose. Google Patents: 2016.
13. Scheffler, U.; Mahrwald, R., Histidine-catalyzed asymmetric aldol addition of enolizable aldehydes: Insights into its mechanism. *The Journal of organic chemistry* **2012,** *77* (5), 2310-2330.
14. Nakata, K.; Gotoh, K.; Ono, K.; Futami, K.; Shiina, I., Kinetic Resolution of Racemic 2-Hydroxy-γ-butyrolactones by Asymmetric Esterification Using Diphenylacetic Acid with Pivalic Anhydride and a Chiral Acyl-Transfer Catalyst. *Org. Lett.* **2013,** *15* (6), 1170-1173.
15. Bourgeois, F.; Medlock, J. A.; Bonrath, W.; Sparr, C., Catalyst repurposing sequential catalysis by harnessing regenerated prolinamide organocatalysts as transfer hydrogenation ligands. *Organic letters* **2019,** *22* (1), 110-115.
16. Vugts, D. J.; Aktas, H.; Al-Mafraji, K.; De Kanter, F. J.; Ruijter, E.; Groen, M. B.; Orru, R. V., Synthesis of 3′-Deoxyribolactones using a Hydrolysis-Induced Lactonization Cascade Reaction of Epoxy Cyanohydrins. Wiley Online Library: 2008.
17. Sheldrick, G., A short history of SHELX. *Acta Crystallogr. Sect. A* **2008,** *64* (1), 112-122.
18. Schrödinger *Release 2022-2*, Schrödinger, LLC: New York, NY, 2022.
19. Philipp, D. M.; Friesner, R. A., Mixed ab initio QM/MM modeling using frozen orbitals and tests with alanine dipeptide and tetrapeptide. *J. Comp. Chem.* **1999,** *20*, 1468-1494.
20. Sastry, G. M.; Adzhigirey, M.; Day, T.; Annabhimoju, R.; Sherman, W., Protein and ligand preparation: parameters, protocols, and influence on virtual screening enrichments. *J. Comput. Aided Mol. Des.* **2013,** *27* (3), 221-34.
21. Olsson, M. H. M.; Søndergard, C. R.; Rostkowski, M.; Jensen, J. H., PROPKA3: Consistent Treatent of Internal and Surface Residues in Empirical pKa predictions. *J. Chem. Theor. Comput.* **2011,** *7*, 525-537.
22. Lu, C.; Wu, C.; Ghoreishi, D.; Chen, W.; Wang, L.; Damm, W.; Ross, G. A.; Dahlgren, M. K.; Russell, E.; Von Bargen, C. D.; Abel, R.; Friesner, R. A.; Harder, E. D., OPLS4: Improving Force Field Accuracy on Challenging Regimes of Chemical Space. *J Chem Theory Comput* **2021**.
23. Schrödinger *Release 2022-2: Macromodel*, Schrödinger, LLC: New York, NY, 2022.
24. Still, W. C.; Tempczyk, A.; Hawley, R. C.; Hendrickson, T., Semianalytical Treatment of Solvation for Molecular Mechanics and Dynamics. *J. Am. Chem. Soc.* **1990,** *112* (16), 6127-6129.
25. Schrödinger *Release 2022-2: QSite*, Schrödinger, LLC: New York, NY, 2022.
26. Murphy, R. B.; Philipp, D. M.; Friesner, R. A., A mixed quantum mechanics/molecular mechanics (QM/MM) method for large-scale modeling of chemistry in protein environments. *J. Comp. Chem.* **2000,** *21*, 1442-1457.

27. Ciulli, A.; Chirgadze, D. Y.; Smith, A. G.; Blundell, T. L.; Abell, C., Crystal Structure of *Escherichia coli* Ketopantoate Reductase in a Ternary Complex with NADP+ and Pantoate Bound: substrate recognition, conformational change, and cooperativity. *J. Biol. Chem.* **2007,** *282* (11), 8487-8497.
28. Goto, M.; Muramatsu, H.; Mihara, H.; Kurihara, T.; Esaki, N.; Omi, R.; Miyahara, I.; Hirotsu, K., Crystal Structures of Δ1-Piperideine-2-carboxylate/Δ1-Pyrroline-2-carboxylate Reductase Belonging to a New Family of NAD(P)H-dependent Oxidoreductases: Conformational Change, Substrate Recognition, and Stereochemistry of the Reaction. *J. Biol. Chem.* **2005,** *280* (49), 40875-40884.
29. Harder, E.; Damm, W.; Maple, J.; Wu, C.; Reboul, M.; Xiang, J. Y.; Wang, L.; Lupyan, D.; Dahlgren, M. K.; Knight, J. L.; Kaus, J. W.; Cerutti, D. S.; Krilov, G.; Jorgensen, W. L.; Abel, R.; Friesner, R. A., OPLS3: A Force Field Providing Broad Coverage of Drug-like Small Molecules and Proteins. *J Chem Theory Comput* **2016,** *12* (1), 281-96.
30. Kolossvary, I.; Guida, W. C., Low-mode conformational search elucidated: application to $C_{39}H_{80}$ and flexible docking of 9-deazaguanine inhibitors into PNP. *J. Comput. Chem.* **1999,** *20* (Copyright (C) 2013 American Chemical Society (ACS). All Rights Reserved.), 1671-1684.
